# 10th Anniversary of the European Association for Predictive, Preventive and Personalised (3P) Medicine - EPMA World Congress Supplement 2020

**DOI:** 10.1007/s13167-020-00206-1

**Published:** 2020-08-19

**Authors:** Olga Golubnitschaja, Ondrej Topolcan, Radek Kucera, Vincenzo Costigliola, EPMA, M. Akopyan, S. N. Akulov, O Alexandrova, Angel Alonso, Russell J. Andrews, A Assuane Duarte, O. Azarova, Maximilian Babel, L. Babenko, A. Barilo, V. A. Baturin, Joanna Bauer, Jan Baxa, Karl-Friedrich Becker, Zuzana Bečková, I. Belenov, I. Belenova, Sergi Beltrán, Peter Berek, Toya Bernad, Jaroslav Betka, R. O. Boshyan, Nadiya Boyko, Paul Brennan, Christoph Brochhausen, Pavel Broz, Rostyslav Bubnov, Rosario Carmona, A. Cibisev, Renata Cifkova, Joaquín Dopazo, V. Drobnych, Viktória Ďurajová, Petr Duras, M. Eremin, I. Esaulenko, Gonzalo Etayo, Matthias Evert, M. Evsevyeva, Jiri Ferda, Eva Ferdova, A. FilinaL, Jan Filipovsky, V. A. Frolov, Radka Fuchsova, J. S. Fuentes Jiménez, E. Fursova, Bretislav Gal, A. V. Gavrish, Julius Gelzinsky, Elisha Goldstein, Olga Golubnitschaja, David Gómez-Cabrero, Luis Angel González, E. E. Grinenko, Ivo Gut, Lukas Hauer, Zdenka Hertelyova, M. Hofmann-Apitius, M. Holubova, Petr Hosek, Karel Houdek, M. W. Huellner, N. A. Iamanidze, Yu. A. Ippolitov, Marko Kapalla, Marie Karlikova, O. Khrypunova, Judita Kinkorova, Romana Koberova-Ivancakova, Іvan Kopolovets, Eva Korcakova, I. Koretskaya, P. V. Koroy, V. Koshel, M. A. Kovalevskaia, Gabriela Krakorova, Juraj Kubáň, Radek Kucera, E. D. Kuchumova, V. Kudryavtseva, A. A. Kunin, D. A. Kunin, T. Kupets, N. I. Kurysheva, V. Lage-Rupprecht, Miriam Lapuníková, Iñigo Lasa, Gorka Lasheras, L. Lazarenko, Na Li, Haihai Liang, V. Lim, V. Liska, E. Loboda, Dmytro Lotnyk, P. A. Lukuanovich, Alberto Maillo, V. Malakhovskiy, L. Manni, Stepan Mares, Petr Martinek, Leslie Matalonga, Martin Matejovic, S. Matelo, Marketa Materankova, Otto Mayer, T. Meleshko, Vlasta Merglova, A. Mikhailova, María Miranda, Hynek Mirka, N. S. Moiseeva, E. Mokin, N. Mokina, Jiri Molacek, L. Monasipova, M. Mozaffari, Rastislav Mucha, Petr Mukensnabl, M. V. Naprienko, C. Nardini, Tanja Niedermair, V. A. Nikolaev, LYu Orekhova, Pavel Ostasov, M. Ovchinnikova, M. Pachkoria, R. Palek, Sara Pasalodos, Friedemann Paul, Javier Pérez-Florido, Martin Pesta, Iñaki Pinillos, Halina Podbielska, Jiri Polivka, Jiri Polivka, Nikola Ptakova, V. Pyatin, Daria Radchenko, A. Raj Dahal, Hana Rezackova, Vladimir Rohan, J. Rosendorf, R. Rukavchuk, M. Rusidi, S. Sadikario, O. Safonicheva, Josefa Salgado, Nafiseh Sargheini, V. J. Sarithala, Jitka Seidlerova, Pavel Seredin, O. Sergeeva, R. Shabanov, Niva Shapira, I. P. Shurygina, Vladimir Sihotsky, Vaclav Simanek, E. V. Sitkina, Ivana Sklenková, David Slouka, L. V. Smekalkina, N. Smirnova, I. Smokovski, A. Sokolovskiy, M. Spivak, Drahomira Springer, E. Stchetinin, Peter Stefanic, Janek Stela, Kamila Stibrana, David Suchy, Marian Svajdler, Tomas Svoboda, Veronika Svobodova, V. V. Tachalov, Ondrej Topolcan, Stefan Toth, N. Tsygankova, V. Tsygankova, A. I. Veremeenko, Cees Vermeer, Y. Vinnichenko, Y. V. Vladimirova, Josef Vodicka, O. Vycital, Wilko Weichert, Jindra Windrichova, A. V. Yagoda, L. A. Yakunina, Xianquan Zhan, V. G. Zilov

**Affiliations:** 1 Predictive, Preventive and Personalised (3P) Medicine, Department of Radiation Oncology, University Hospital Bonn, Rheinische Friedrich-Wilhelms-Universität Bonn, Venusberg-Campus 1, 53127 Bonn, Germany; 2 University Hospital in Pilsen, Medical Faculty in Pilsen, Charles University, Prague, Czech Republic; 3 European Medical Association, Brussels, Belgium; 4 European Association for Predictive, Preventive and Personalised Medicine, EPMA, Brussels, Belgium

## Abstract

In 2019, the EPMA celebrated its 10th anniversary at the 5th World Congress in Pilsen, Czech Republic. The history of the International Professional Network dedicated to Predictive, Preventive and Personalised Medicine (PPPM / 3PM) is rich in achievements. Facing the coronavirus COVID-19 pandemic it is getting evident globally that the predictive approach, targeted prevention and personalisation of medical services is the optimal paradigm in healthcare demonstrating the high potential to save lives and to benefit the society as a whole. The EPMA World Congress Supplement 2020 highlights advances in 3P medicine.


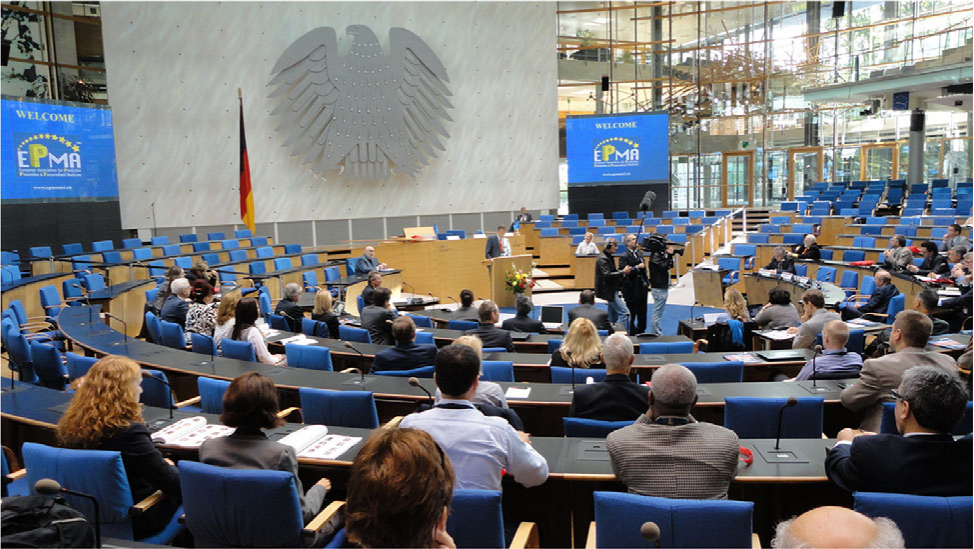
The historical 1st EPMA World Congress in former Bundestag, Bonn, Germany, September 2011

## Introduction

European Association for Predictive, Preventive and Personalised Medicine has been created in 2009. In 2011 the historical 1st EPMA World Congress took place in Bonn, Germany.

In 2019, the EPMA celebrated its 10th anniversary at the 5th World Congress in Pilsen, Czech Republic. The decade-old professional history of the EPMA is rich in achievements. Herewith we briefly highlight some of them.

**Geographic distribution** of the 3PM-relevant expertise under the EPMA-umbrella started with approximately 20 countries in 2009; currently, the EPMA is represented in 54 countries worldwide who actively promote 3PM concepts in bio/medical sciences and practical medicine strongly benefiting patients and healthcare systems.

The first issue of the **EPMA Journal**, ***Springer Nature***, was released in March 2010. In 2018, the journal received its first IF 3.9; in 2020, it reached 4.901. Nowadays the EPMA J. is a highly recognised international forum for 3P medicine operating in a hybrid subscription/open access modus. Scopus CiteScore 2019 of the EPMA J. is 7.7, https://www.scopus.com/sourceid/19700201201?origin=sourceInfo&zone=refpointrank#tabs=1, thereby Scopus ranks the EPMA J. amongst the top 3% in the category “Health Policy”, due to highly requested and well-cited strategic papers created by multi-professional groups of EPMA experts such as

- *General report & recommendations in predictive, preventive and personalised medicine 2012: white paper of the European association for predictive, preventive and personalised medicine.* 10.1186/1878-5085-3-14.
- *Medicine in the early twenty-first century: paradigm and anticipation – EPMA position paper 2016.* 10.1186/s13167-016-0072-4.

***SCImago*** top-ranks the EPMA J. in all three categories, namely “*Health policy*”, “*Medical Biochemistry*” and “*Drug discovery*”:

https://www.scimagojr.com/journalsearch.php?q=19700201201&tip=sid

In 2018, Springer Nature awarded the below-mentioned article the status of an “article with a potential to change the world” in the category “*Medicine and Public Health*”, https://www.springernature.com/gp/researchers/campaigns/change-the-world/medicine-public-health

- *Pregnancy Associated Breast Cancer: The Risky Status Quo and New Concepts of Predictive Medicine.* EPMA J. 2018, 10.1007/s13167-018-0129-7.

**“Advances in Predictive, Preventive and Personalised Medicine”** is a very successful EPMA/Springer Nature book series which educates both professionals and the general population in 3P medicine. Since 2012, 12 book volumes have been released dedicated to a whole spectrum of PPPM related aspects such as digital health, information technology framework, application of artificial intelligence in healthcare, drug delivery systems, liquid biopsy and multi-level diagnostics, amongst others.

**“Horizon 2020”** is the main European Scientific Programme which EPMA experts have contributed to with 3PM-related protocols as well as with the top-expertise provided by Representatives and Members of the association involved in the evaluation panels.

**EPMA AWARD for EXCELLENCE in BIOMEDICAL SCIENCES** was created in 2017, and the 1st EPMA award was given to Prof. Dr. Josef Flammer, University of Basel, for phenotyping of the “Flammer Syndrome”, which the EPMA international jury panel valued as being of great clinical utility.

“**Young professionals in PPPM” Award** was created by the EPMA in 2015. At the international workshops linked to the biannual EPMA World Congresses, the presentations made by young professionals get evaluated by an international jury panel. The best presentations and smart 3PM concepts receive awards that effectively promote the careers of young professionals in innovative bio/medical fields.

**EPMA World Congress 2019 in Pilsen, Czech Republic** attracted 3PM experts from 35 countries. The congress was dedicated to innovation in a broad spectrum of bio/medical fields with a specific focus on the concepts of ***predictive diagnostics, targeted prevention and personalisation of medical services*** in “Cancer”, “Metabolic Disorders”, “Cardiovascular Disease”, “Neurological, Neurodegenerative and Neuropsychiatric Disorders”, “Inflammatory Disorders”, “Dentistry”, “Biobanking and Screening Programmes”, “Multi-omics”, “Microbiome, Immune-, Pre- and Probiotics”, and “Innovative Technologies”, among others. Further, there were several new topics presented at the congress: among others these were “Implementation of 3PM Concepts in Plastic Surgery”, “Application of Artificial Intelligence in Medicine – 3PM strategies” and “Medical Use of Cannabis”. The latter topic was discussed in the EU Parliament in 2019, and the EPMA position has been elucidated by the EPMA Representatives; for more information see the below link:

http://www.epmanet.eu/latest/events/2019/epma-position-on-medical-use-of-cannabis-presented-at-the-eu-parliament.

Oral and poster presentations provided valuable information regarding pilot projects towards personalised healthcare (e.g. awarded by ICPerMed), individualised patient profiles, multi-level biomarker panels, patient stratification, creation and application of innovative IT-tools, ethical issues, doctor-patient collaboration, optimal structure and organisation of the modern hospital ambitioned to practically implement the paradigm change from reactive to predictive, preventive and personalised medicine.

**World First 3P Medical Unit**

In March 2020, the historically first worldwide unit dedicated to Predictive, Preventive and Personalised (3P) Medicine led by Secretary-General of the EPMA, Prof. Dr. Olga Golubnitschaja, was created in Germany at the Department of Radiation Oncology, University Hospital, Rheinische Friedrich-Wilhelms-Universität Bonn.

## 3PM vision and strategies

**PPPM for Twenty-first Century Biosensing: Painless, Personalised, Point-of-Care Monitoring with Wearable and Implantable Devices**

Andrews RA*

*Corresponding author: Nanotechnology & Smart Systems, 121 NASA Ames Research Center, 122 Moffett Field, CA, USA; e.mail: rja@russelljandrews.org

**Keywords:** Artificial intelligence, Biosensors, Blood pressure monitoring, Brain-computer/Brain-machine interface, Continuous monitoring, Diabetes, Electrocardiogram, Electroencephalogram, Epilepsy, Fall detection, Gait disorders, Glucose monitoring, Implantable sensors, Ingestible sensors, Internet of things, Iontophoresis, Interstitial fluid, Nanosensors, Neurotechnology, Pressure monitoring, Saliva monitoring, Seizure detection, Smart contact lenses, Smart mouthguards, Smart patches, Smart skin, Smart watches, Smartphone apps, Skin patches, Sweat monitoring, Tear monitoring, Temperature monitoring, Tissue-device interface, Wearable sensors, Wireless monitoring

**Introduction**

Many people do not realize they already have adopted wearable devices for medical monitoring—smartwatches. Typical stories of smartwatches providing life-saving diagnostic information include the following: (1) A smartwatch alarming all night regarding abnormal heartrate alerted the wearer to seek medical attention for what proved to be atrial fibrillation [1]; (2) A hiker—lost as nightfall approached—stumbled and fell on difficult terrain. Unbeknownst to the hiker, the fall triggered his smartwatch to automatically call the emergency phone number (911 in the USA), thereby avoiding what could have been a tragic outcome. Smartphones, with accelerometer and GPS capabilities, have apps for people with epilepsy who may require emergency medical assistance [2].

Medical monitoring has not always been so painless, persistent, and unobtrusive. Atrial fibrillation required attaching electrodes to the skin with a conductive gel, in turn connected to a device—possibly portable, but certainly obtrusive. Monitoring of blood glucose by diabetic patients required repeated finger-sticks—painful, intermittent, and obtrusive.

**Phases of Biofluid Monitoring**

Diagnostic techniques for biofluids, e.g., blood, urine, saliva, and cerebrospinal fluid (CSF), have evolved over the past several decades (Fig. 1) [3]. The first phase—extending from the twentieth century to the present—entails obtaining a sample from the patient (an invasive procedure for blood and CSF) and sending it to a laboratory for analysis. Results are not available for hours to days for samples obtained from outpatients, and minutes to hours for inpatients.
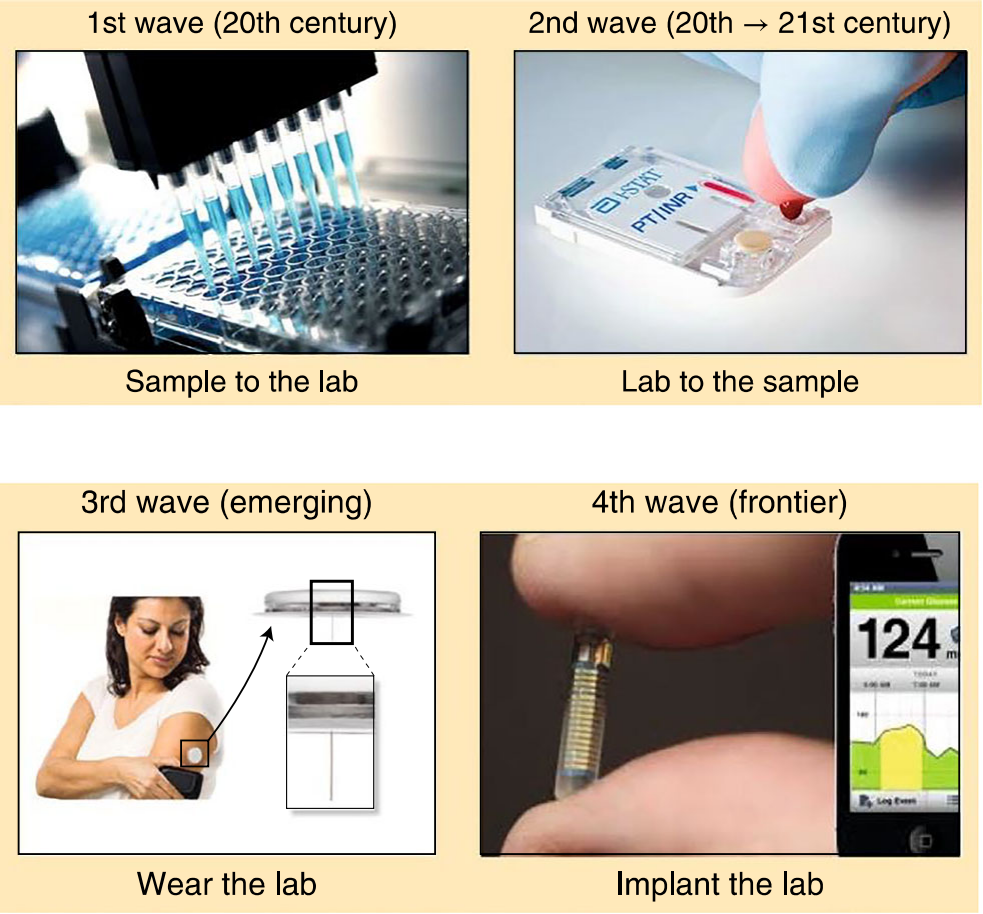


**Fig. 1** The four technological waves of biochemical monitoring. (reference [3], with permission)

The second phase began about two decades ago with point-of-care (POC) monitoring, where the laboratory comes to the patient (i.e., to the recently obtained sample) rather than transporting the sample to the laboratory.

The third phase, more recently available, consists of wearable devices. This is the epitome of POC monitoring since the patient and the device are inseparable. Smartwatches can do this for pulse and blood pressure, patches applied to the skin for continuous blood glucose monitoring. The patches, e.g., for glucose monitoring, typically monitor the analyte concentration in interstitial fluid (ISF), which closely parallels blood glucose [3–7].

The line between the third and fourth phases—wearable and implantable devices—is blurred. Part of this is due to expansion of the fluids monitoring from blood (or ISF) to sweat, saliva, and tears. Most would call a mouthguard to monitor saliva a wearable device—but what about a “smart” contact lens to monitor tears? Truly implantable devices (e.g., inserted subcutaneously by a minor surgical procedure) can monitor analytes such as glucose for months (potentially longer) rather than the days to a week or so of most patches [8].

**Power to the Patient—Digitizing Biofluid Monitoring**

During the sample-to-lab and POC phases, urine was the ideal biofluid—non-invasively obtained and relatively easily transported. Blood required an invasive procedure, a needlestick. Sweat and tears were not easily obtained in a manner guaranteeing uniformity, and saliva could vary greatly depending on time of sampling (e.g., after a drink or a meal).

Wearable devices have transformed those problems into one consisting of a tissue-device interface (TDI) challenge. Continuous biofluid monitoring is a reality: sampling urine, blood, or other biofluids continuously was not practical previously outside a hospital setting (with an indwelling catheter for urine or blood or even CSF). A second problem in phases one and two was obtaining continuous diagnostic information from the biofluid.

The smartphone and smartwatches plus machine learning and artificial intelligence (AI) have allowed not only continuous biofluid monitoring but also continuous, real-time interpretation of that monitoring information in a precise and personalized manner—“digital biomarkers” [9]. This can answer the question:

“What does this biofluid monitoring value mean for this particular patient at this precise moment?”

Once answered, that information can guide real-time, precise, personalized treatment, e.g., continuous feedback-guided (or closed-loop) insulin release in diabetes. The patient, if desired, can have control over when the biofluid monitoring information is gathered or processed or transmitted, e.g., to a databank. The patient can remove the patch or the smartwatch, or turn off the smartphone containing the app transferring the data.

**Blood, Sweat, Tears, and Saliva**

Although the primary target has been a wearable monitor of blood glucose for diabetic patients, other biological signals that can be measured through the skin include chemicals (beyond glucose—potassium, chloride, lactate), electrical (electrocardiogram (ECG), electroencephalogram (EEG), electromyogram (EMG)), and physical (temperature, pressure, light, sound) [3–7]. Additionally, non- or minimally invasive monitoring has included measures ranging from respiratory rate to joint movement to gait [5, 9–11]. This review is primarily limited to the TDI for biofluids.

Wearable skin patches depend on knowledge of the structure of human skin [3, 6, 7]. “Smart” skin exhibits many technological advances, as illustrated in Fig. 2 [7]. Skin patches usually monitor ISF concentrations of the chemical of interest, relatively straightforward for ISF glucose (as a surrogate for blood glucose). Sweat, however, poses a different problem, since sweat is not continuously available for monitoring. In the typical skin patch for sweat, the patch incorporates an electrode to deliver a cholinergic agent such as carbachol into the skin for stimulation of sweat (iontophoresis) [3, 6].
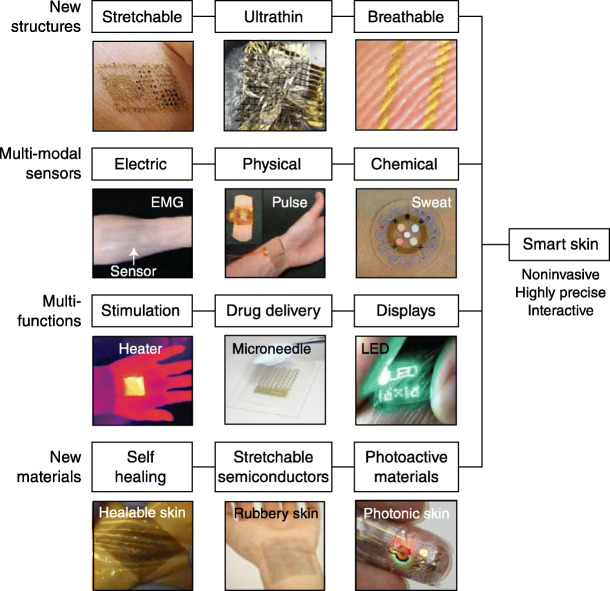


**Fig. 2** Recent research trends in smart skin from four viewpoints. First, the structures of smart skins are advancing from stretchable to ultra-thin to breathable sensors, resulting in enhancement of biocompatibility and reduced burden of sensor attachment. Second, multi-modality is expanding from electrical to physical to chemical sensors. Third, more advanced functions such as stimulation, drug delivery, and displays are being incorporated, in addition to sensing functions. Fourth, novel materials such as self-healing conductors, intrinsically stretchable semiconductors, and photoactive materials are being developed. (reference [7], with permission)

Monitoring tears is challenging: (1) the rate of tearing is not uniform; (2) the device must be acceptable to the patient. Tear-based biofluid sensors include smart contact lenses and devices placed in the lower eyelid (Fig. 3A) [4, 6].
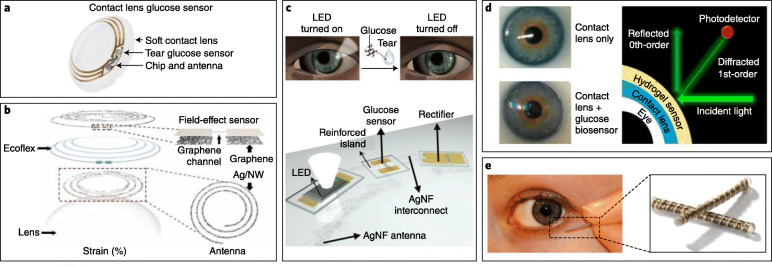


**Fig. 3A** Tear-based biosensors. **a** Contact lens sensor previously under development by Google and Novartis to measure tear glucose concentration. Prototype platform contained integrated electronics for sensor response processing and wireless transmission. **b** Multifunctional wearable smart sensor system incorporated onto a contact lens for monitoring both glucose in tears and intraocular pressure using enzyme-functionalized graphene-silver nanowire hybrid nanostructures. **c** A wireless glucose sensor incorporated into a contact lens platform with wireless power transfer circuitry and display pixels for a fully integrated and transparent platform that does not hinder vision. **d** Wearable contact lens tear glucose biosensor applied to an artificial eye, with schematic representation of smartphone-based quantification of glucose levels through reflection of incident light by the photonic microstructure within the lens. The smart contact lens system integrated with a glucose sensitive hydrogel monitors changing glucose concentrations in vitro without complicated fabrication procedures and allows rapid response time for continuous measurements. **e** NovioSense electrochemical tear glucose sensor. A small spring-like sensing device is designed to be placed within the conjunctival fornix for continuous access to tear glucose. (reference [6], with permission)

Saliva is readily available but suffers from analyte variability (e.g., temperature and concentration) resulting from the presence of liquids of varying temperatures over time in the oral cavity (hot vs cold drinks) [6]. Patient acceptance of a device in the oral cavity—given that some saliva biofluid sensors are mouthguards or otherwise bulky/obtrusive—is another issue (Fig. 3B) [6].
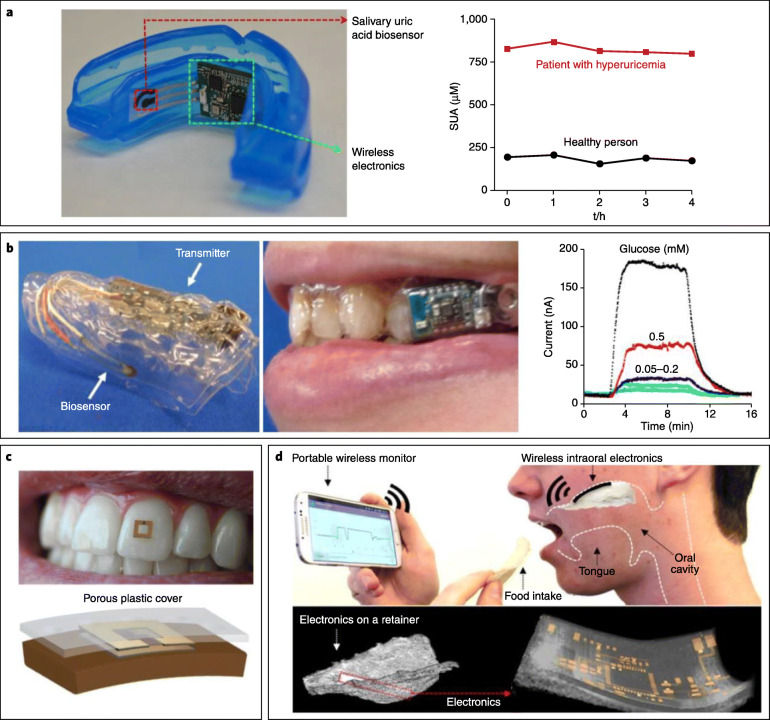


**Fig. 3B** Saliva-based biosensors. **a** Mouthguard-based wearable salivary uric acid biosensing platform with integrated wireless electronics and analysis of salivary uric acid concentrations. **b** Mouthguard-based sensor for glucose monitoring in saliva with on-body application and analysis of increasing glucose concentrations. **c** On-body depiction and cross-sectional configuration of radio frequency trilayer tooth-mounted sensor for wireless monitoring of food consumption. This dielectric sensor fabricated with biocompatible materials is capable of being mounted onto tooth enamel to detect foods and fluids during ingestion when functionalized with analyte-sensitive layers. Projected uses were for detection of sugars, alcohol, salinity, pH, and temperature. **d** Operational principles and electronics configuration of a wireless, user-comfortable sensing platform for long-range oral monitoring of sodium intake during hypertension management. (reference [6], with permission)

**Sometimes It Takes Guts to Monitor**

Confirmation of ingestion of prescribed medications, particularly in unreliable patients (e.g., dementia), is another biosensing challenge. One solution is the “smart pill”—a capsule containing a microsensor that is swallowed, monitoring whether the medication is present in the stomach [4]. The “smart pill” communicates with a skin patch, which not only documents that the pill was swallowed (and when) but also (if desired) blood pressure, pH, and temperature.

For continuous monitoring, a sensor can be stationed in the gut (most likely the stomach). Such monitoring could include medication ingestion, pH, controlled drug delivery, and imaging of the gut lining. An ingestible sensor that is self-powered by stomach acid in contact with zinc and copper electrodes on the sensor surface is being developed [12]. Another ingestible capsule under development uses a microneedle that inserts into the stomach wall to deliver a drug (e.g., insulin) [13].

**Wear Your Heart on Your Sleeve; Wear Your Brain on Your Hat**

The topic of brain biomonitoring—from EEG to next-generation brain-machine interfaces (BMIs)—is beyond the scope of this article but has been recently reviewed [14]. An area of concern regarding brain biomonitoring is direct-to-consumer (DTC) marketing of devices that are of undocumented value or possible risk [15–17]. Brain biomonitoring information obtained through DTC marketing raises questions of both personal privacy and ultimate use of such data by marketers. Increasing DTC availability of brain electrical stimulation (e.g., via a skullcap), notably transcranial direct current, alternating current, and random noise stimulation techniques, raises questions of safety [15]. Ethical considerations regarding DTC brain biomonitoring and biostimulating remain unresolved [15–17].

**Conclusions and Expert Recommendations**

The field of wearable and implantable biosensors is evolving so rapidly that no review truly reflects the “state-of-the-art.” Advances in the TDI and AI promise that such devices will not only enhance diagnostic capabilities but also provide a wealth of information for improved treatments.

Specific recommendations:

1. Incorporating the latest technology into biosensors—from nanotechniques to microfluidics—is essential. A smartphone from ten years ago would be unacceptable in the consumer marketplace; outdated diagnostic techniques in medicine are similarly unwarranted.
2. Similarly, the latest AI is necessary to analyze the huge amounts of data that wearable and implantable biosensors provide.
3. The consumer/patient must be involved in device development from the outset. What may appear wonderful in the lab or the boardroom may prove a failure in the marketplace and social media. Consumer/patient acceptance (CPA) is crucial for widespread adoption.
4. Flexibility is key. Some patients may prefer a patch for continuous glucose monitoring, others a smart contact lens, and others an implanted device (requiring a minor procedure for implantation but not frequent replacement). What works in a high-income country such as Belgium may not work in a low-income country such as Burkina Faso.
5. Legislation and safeguards regarding the huge amounts of personal medical data generated by wearable and implantable biosensors is essential, since data collection and storage systems can be hacked. This is especially crucial with regard to biomodulating devices, e.g., cardiac pacemakers, brain stimulation, and controlled drug delivery systems.
6. Given the vulnerability to hacking, wearable and implantable biosensors require the same caution as other widespread threats to population health, e.g., toxins (both liquid and aerosol), biological warfare agents, and radiation.

**References**

1. Weichert W. ‘My watch kept on alarming all night about my heart rate’. Oxford Med Case Rep. 2019;3:124–126.
2. Seizario: detecting seizures and falls. Available: https://seizario.healthhappy.com [Accessed 27 Apr 2019].
3. Heikenfeld J, Jajack A, Feldman B, Granger SW, Gaitonde S, Begtrup G, et al. Accessing analytes in biofluids for peripheral biochemical monitoring. Nat Biotechnol. 2019;37:407–419.
4. Guk K, Han G, Lim J, Jeong K, Kang T, Lim EK, et al. Evolution of wearable devices with real-time disease monitoring for personalized healthcare. Nanomaterials. 2019;9:813.
5. Khan S, Ali S, Bermak A. Recent developments in printable flexible and wearable sensing electronics for healthcare applications. Sensors. 2019;19:1230.
6. Kim J, Campbell AS, Esteban-Fernandez de Avila B, Wang J. Wearable biosensors for healthcare monitoring. Nat Biotechnol. 2019;37:389–406.
7. Someya T, Amagai M. Toward a new generation of smart skins. Nat Biotechnol. 2019;37:382–388.
8. Waltz E. Sweet sensation. Nat Biotechnol. 2019;37:340–344.
9. McCarthy A. The biomarker future is digital. Clinical OMICS. 2020(Jan/Feb):24–28.
10. Massaroni C, Nicolo A, Lo Presti D, Sacchetti M, Silvestri S, Schena E. Contact-based methods for measuring respiratory rate. Sensors. 2019;19:908.
11. Faisal AI, Majumder S, Mondal T, Cowan D, Naseh S, Deen MJ. Monitoring methods of human body joints: state-of-the-art and research challenges. Sensors. 2019;19:2629.
12. McDonnell S. Ingestible sensors powered by stomach acid. Tech Briefs. 2018(Aug):45–46.
13. Jarchum I. To the stomach and beyond. Nat Biotechnol. 2019;37:377–381.
14. Frank JA, Antonini MJ, Anikeeva P. Next-generation interfaces for studying neural function. Nat Biotechnol. 2019;37:1013–1023.
15. Ienca M, Haselager P, Emanuel EJ. Brain leaks and consumer neurotechnology. Nat Biotechnol. 2018;36:805–810.
16. Wexler A. Separating neuroethics from neurohype. Nat Biotechnol. 2019;37:988–992.
17. Jarchum I. The ethics of neurotechnology. Nat Biotechnol. 2019;37:993–996.

**The Navarra 1000 Genomes Project (NAGEN 1000): Benefits for Predictive, Preventive and Personalized Medicine**

Pasalodos S^1^, Salgado J^1^, Miranda M^1^, Maillo A^1^, Matalonga L^2^, Beltrán S^2^, Carmona R^3^, Pérez-Florido J^3^, Etayo G^4^, Lasheras G^4^, Bernad T^4^, Gómez-Cabrero D^1^, Angel González L^5^, Brennan P^6^, Gut I^2^, Dopazo J^3^, Pinillos I^4^, Lasa I^1^, Alonso A*^1^

^1^Navarrabiomed, Complejo Hospitalario de Navarra. Universidad Pública de Navarra (UPNA), IdiSNA, Pamplona, Spain.

^2^Centro Nacional de Análisis Genómico (CNAG-CRG), Center for Genomic Regulation, Barcelona Institute of Science and Technology (BIST), Barcelona, Spain.

^3^Área de Bioinformática, Fundación Progreso y Salud, Nodo de Genómica Funcional, (INB-ELIXIR-es), Bioinformática de ER (BiER-CIBERER), CDCA, Hospital Virgen del Rocío, Sevilla, Spain

^4^Navarra de Servicios y Tecnología NASERTIC. Spain

^5^AVANTIA 400+, Pyramide Asesores. Spain

^6^NENC NHS Genomic Medicine Centre. Newcastle upon Tyne, UK.

*Corresponding author: Dr. Angel Alonso. Genomic Medicine Unit. Navarrabiomed, Complejo Hospitalario de Navarra. Universidad Pública de Navarra (UPNA), IdiSNA. C/Irunlarrea, 3, 31008 Pamplona, Spain; e.mail: angel.alonso.sanchez@navarra.es

**Keywords:** predictive preventive personalized medicine, genomics, next generation sequencing, NGS, whole genome sequencing, WGS, rare diseases, eHealth, bioinformatics, big data, ICPerMed, multi-omics

**Background**

In the past few years, extraordinary developments in the field of next generation sequencing (NGS) technologies, such as whole genome sequencing (WGS), have made it possible for clinicians to have access to a huge amount of biological information which could potentially explain complex genetic diagnoses, genetic predisposition to severe diseases, reproductive risks and inappropriate responses to certain medications. These advances herald a new era of predictive preventive personalized medicine (PPPM), although incorporation into clinical practice has proved to be challenging [1]. “NAGEN 1000” is a Spanish regional pilot study to implement recent advances of cutting edge genomic research technology into real clinical practice.

**Goal, materials and methods**

NAGEN 1000’s main goal is the implementation of the whole genome sequencing (WGS) derived information as a clinical tool for the development of PPPM in the Public Health Service.

A scientific implementation approach was used to identify and categorize both the local barriers and facilitators to accelerate the incorporation of translational genomics into healthcare (see Fig. 1).
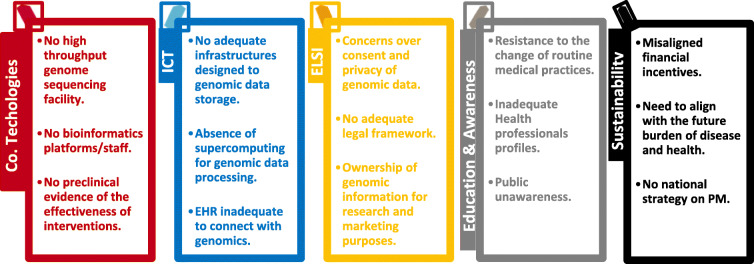


Fig. 1. Local barriers for genomic medicine implementation in Navarra (NAGEN 1000 project)

Key Actions for this implementation:

1. Subjects: NAGEN 1000 is recruiting 1000 patients, and their relatives, affected with one condition from a list of nearly 200 rare diseases (RD). Albeit rare, joint RD’s prevalence is high (2–7%), with a very high social impact, wide multidisciplinary medical coverage, and a high rate of identifiable genetic causes. These features make it possible to involve the medical community, raise population awareness and offer good support to evidence-based medicine practice. The rate of 1 genome per 500 inhabitants facilitates a wide participation from patients and health professionals.
2. Results and incidental findings: Pertinent findings explaining the referral condition, secondary findings on personal and reproductive risks of severe inherited diseases, and pharmacogenomic variants determining drugs dose and toxicity are reported, based on patient’s choice, providing the necessary evidence of the effectiveness of medical interventions based on genomic medicine. New genetic counselling interventions, variant validation and reporting pathways have been put in place for the best provision of services.
3. Electronic health record (EHR) adaptation: The existing EHR has been modified to host a newly designed recruitment tool which enables and guides the identification and immediate referral of patients from any point in the Navarre health system network. An additional development also makes it possible that clinically actionable genomic results are available for participants’ doctors with all other clinical information across the system.
4. Clinical research: A number of new exciting genomic results, potentially providing new insights into the genetic basis of RDs, and additional information on population genomics, are being produced by NAGEN 1000, offering exceptional material to support new research. It is a main goal of the Project to ensure an adequate data harmonization, which enables data sharing for research under an appropriate regulatory and legal framework.
5. Optimized use of pre-existing public infrastructures: In order to overcome the lack of local facilities, NAGEN 1000 externalizes WGS sequencing services to CNAG-CRG, the Spanish world leader public centre for genomic analysis. Bioinformatic analysis also relies primarily on CNAG-CRG through the RD-Connect Genome-Phenome Analysis Platform which was deployed for the project to store, analyse and interpret the genomic, data making use of the phenotypes encoded with the Human Phenotype Ontology (HPO), and the experts from the Bioinformatics Platform of the Rare Diseases Spanish network (CIBERer) through the Interactive Variant Analysis (IVA) tool based on the genome browser Genome Maps, but expertise in this field has gradually been transferred to the newly created local Translational Bioinformatics Unit during the course of the Project.
6. ICT: New ICT solutions have been adopted for NAGEN 1000 allowing the storage and high performance managing of massive genomic data, through an innovative partnership with NASERTIC, a local company providing data analysis infrastructures such as the new IBM POWER 9 processor, which build on cross-disciplinary collaboration in research and development with the local industry.
7. ELSI: While genetic data protection is widely regulated for clinical and research purposes, within the NAGEN 1000 project, the local Health Research Authority has specifically resolved that the massive genomic information resulting from WGS will also be part of the patient’s medical record, and it will accordingly be protected and stored. In order to enable the use of genomic data for research, the constitution of a “Genomic Library” has been proposed, which would accept specific research enquiries on anonymized genomic sequences upon pertinent EC approval. This scenario requires a new regulatory legal framework, which has also been explored through a specific partnership with Avantia 400+ from the Pyramide group, a local consulting company with wide experience in data protection.

**Results**

Key results to date:

1. Clinical and pre-clinical results: Around 700 patients have to date followed through the above-outlined pathway, and 33% of the families have now found the long-awaited genetic cause for their previously unexplained condition and now have hope of an improvement of their clinical care based on these findings. Remarkably, 10% of these diagnoses were attributed to genes previously unknown to cause a human disease, or causing different phenotypes than those previously described (Fig. 2). Additionally, 2% of participants carried genetic predispositions to severe diseases, 4% had reproductive risks and 100% had pharmacogenomic actionable variants influencing prescription (Table 1). Further, candidate genomic variants potentially explaining patients’ diseases have been identified in an additional 24% of participating families, which provides an extended base for new collaborative research projects. Interestingly, about 20 different medical specialities have referred patients to NAGEN 1000, indicating a desirable multidisciplinary involvement in this implementation initiative.
2. Healthcare workforce education and public empowerment: Monographic NAGEN 1000 symposiums, hospital briefings, clinical sessions and face to face meetings have been organized, opening the participation to all medical professionals in the region. Moreover, the 18 designated specialities “physician champions”, especially commissioned to facilitate recruitment, help with the clinical interpretation of genomic variants, and to spread the word, received 50 category 1 and 2 CME credits from a NAGEN 1000 tailored genomics education programme. Public involvement has also been possible through a press conference, which was widely covered by national general and medical press and social media, open conferences at the “Science Week” and “Rare Diseases Day”, a specific website www.nagen1000navarra.es, and communications to national and international congresses.
3. Sustainability: After deducting marginal costs due to the Translational Bioinformatics Unit establishment and ICT infrastructures, the cost-effectiveness analysis (CEA) recognized a full running costs of €12,776 per RD diagnosis (prior to familial cascade genetic testing and including duo and trio studies costs when necessary) compared with €18,300 average cost per diagnosis estimated for the standard of care pathway [2]. Considering that cost-benefit analysis (CBA) outperforms CEA for RD, we conducted a survey of all participants which showed that more than 50% of them would be willing to pay more than €10,000 for the genomic information they received after their participation in NAGEN 1000, regardless of whether their diagnosis was ultimately achieved or not.
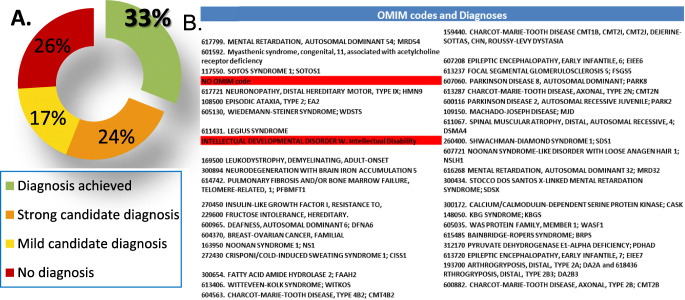

**Fig. 2** Pertinent clinical findings. **a** Pie-chart showing the performance of 33% genomic diagnoses achieved by NAGEN 1000 and 24% of strong and 17% mild candidates genomic variants. **b** Table listing OMIM codes and diagnoses (in red cases with no OMIM codification)

**Table 1.** Clinical Actionable Incidental Findings

**Table:** 

Clinical Actionable Incidental Findings			
Type	Consent	N of patients	% of cases
Disease Predisposition	352	8	2.27%
Reproductive Risk	362	15	4.14%
Pharmacogenomic	388	388	100%

**Conclusions and expected impacts**

Genomics has become a major contributor to multiomics and PPPM related approaches in management of major and fatal pathologies such as cancer, diabetes and stroke [3–5]. NAGEN 1000 illustrates how translational research and innovation in the field of genomics and PPPM is already delivering real benefits to patients, and it was awarded as the Best Practice in Personalised Medicine by ICPerMed in 2018. Significantly, it resulted in setting the new Genomic Medicine Unit of Navarrabiomed and its NAGEN strategy, which has now raised €6 million for 3 R&D projects on PM over the past 4 years. NAGEN is an exemplar practice for the Spanish Senate Initiative for a National Strategy on Genomics and PM (2018), and has given rise to the launch of the Navarra Government Strategy on Personalised Medicine announced in November 2019.

**Acknowledgements**

- This study will always be in debt to all participating patients, their families, and to the referring doctors.
- The designated speciality “physician champions” contributing to this project were: Ana Guerra Lacunza, Angel Alonso Sanchez, Carolina Purroy Irurzun, Emma Anda Apiñaniz, Eva Bandres Ellizalde, Iñaki Molinuevo, Jose Andonegui Navarro, José Zubicaray Ugarteche, Juan Jose Beloqui Lizaso, Lucia Sanchez. Eugenia Yoldi Petri, María Sagaseta De Ilurdoz Uranga, Maite Basurte Elorz, Maite Mendioroz Iriarte, Manuel Cuesta, Marta Montes Díaz, Merce Artigas Lopez, Patricia Fanlo Mateo, Pilar Cebollero Rivas, Raquel Santesteban Muruzabal, Rosario Ibañez Bosch, Sergio Curi, and Sira Moreno Laguna.
- The Navarra Government (Departamento de Desarrollo Económico - Dirección General de Industria, Energía e Innovación) supported this study through the GEMA challenge for the Strategic R&D Projects call 2017–2019, under the Regional Smart Specialization Strategy S3.

**References**

1. Golubnitschaja O, Baban B, Boniolo G, Wang W, Bubnov R, Kapalla M, Krapfenbauer K, Mozaffari M, Costigliola V. Medicine in the early twenty-first century: paradigm and anticipation – EPMA position paper 2016. EPMA J. 2016;7:23. 10.1186/s13167-016-0072-4.
2. Stark Z, Schofield D, Alam K, Wilson W, Mupfeki N, Macciocca I, Shrestha R, White SM, Gaff C. Prospective comparison of the cost-effectiveness of clinical whole-exome sequencing with that of usual care overwhelmingly supports early use and reimbursement. Genet Med. 2017;19:867–874. 10.1038/gim.2016.221.
3. Lu M, Zhan X. The crucial role of multiomic approach in cancer research and clinically relevant outcomes. EPMA J. 2018;9(1):77–102. 10.1007/s13167-018-0128-8.
4. Polivka J Jr, Polivka J, Pesta M et al. Risks associated with the stroke predisposition at young age: facts and hypotheses in light of individualized predictive and preventive approach. EPMA J. 2019;10(1):81–99. 10.1007/s13167-019-00162-5.
5. Duarte AA, Mohsin S, Golubnitschaja O. Diabetes care in figures: current pitfalls and future scenario. EPMA J. 2018;9(2):125–131. 10.1007/s13167-018-0133-y.

**Objectives and achievements of the specialised dental section created by the European Association for Predictive, Preventive and Personalised Medicine**

Kunin A^1^, Esaulenko I^1^, Moiseeva N^1^, Mozaffari M^2^, Golubnitschaja O*^3^

^1^ Voronezh State Medical University named after N.N. Burdenko

^2^Augusta University, Georgia, USA

^3^Predictive, Preventive and Personalised (3P) Medicine, Department of Radiation Oncology, University Hospital Bonn, Friedrich-Wilhelms-University Bonn, Germany

*Corresponding author: Prof. Dr. Olga Golubnitschaja, Predictive, Preventive and Personalised (3P) Medicine, Department of Radiation Oncology, University Hospital Bonn, Rheinische Friedrich-Wilhelms-Universität Bonn, Venusberg-Campus 1, 53127 Bonn, Germany; e.mail: Olga.Golubnitschaja@ukbonn.de

**Keywords:** predictive preventive personalised medicine, dentistry, European Dentistry Department, European Association for predictive, preventive and personalised medicine EPMA, objectives, multi-professional, international, stem cells, dry mouth syndrome, risk assessment, patient stratification, body fluid, biomarker patterns, multi-level diagnostics, innovative technologies, tailored treatments, saliva, digestive disorders, hyposalivation, eating disorders, periodontitis, dental caries, inflammation, stress

**Motivation to create the specialised dental section focused on 3P Medicine**

Dental disorders can cause a great number of pathophysiological conditions such as acute and chronic infections and inflammatory processes, voice and digestive disorders, otorhinolaryngologic pathologies and cancer. Therefore, risk assessment, early diagnosed suboptimal dental health followed by cost-effective targeted prevention and personalisation of medical services as the concept of predictive, preventive and personalised (3P) medicine is beneficial for the quality of life of the patient, healthcare economy and society at large. Owing to the evident lack of 3PM programmes in the current management of dentistry, the ambition of the EPMA is to promote cross-sectional research and practical implementation in the area. The specialised EPMA dental section was created in 2012 towards the initiative of the Departments of Oral and Maxillofacial surgery and Hospital Dentistry, Voronezh State Medical University named after N.N. Burdenko, Russia supported by the the Dental College of Georgia at Augusta University, USA. Since then, more than 20 national centres and international groups have joined the dental section of the EPMA.

**Multi-professional topics of the section**

The dental EPMA-section has created a number of cross-sectional and multi-lateral projects dedicated to the below-listed topics, among others

- Risk assessment, predictive strategies and personalised prevention [1]
- Stem cells and tooth regeneration [2]
- Psychologic aspects in dental practice [3,4]
- “Dry mouth” syndrome and related pathologies [5]
- Pathology-specific body fluid patterns and multi-level diagnostics [6]
- Innovative materials, technologies and approaches in dental practice [7,8]
- Education [4,9,10]

The dental section under the EPMA leadership follows the innovative concepts of 3P Medicine [11]. Regular updates of scientific projects and practical implementation is performed utilising the platform of biannual EPMA World Congresses (Fig. 1) [12–14].
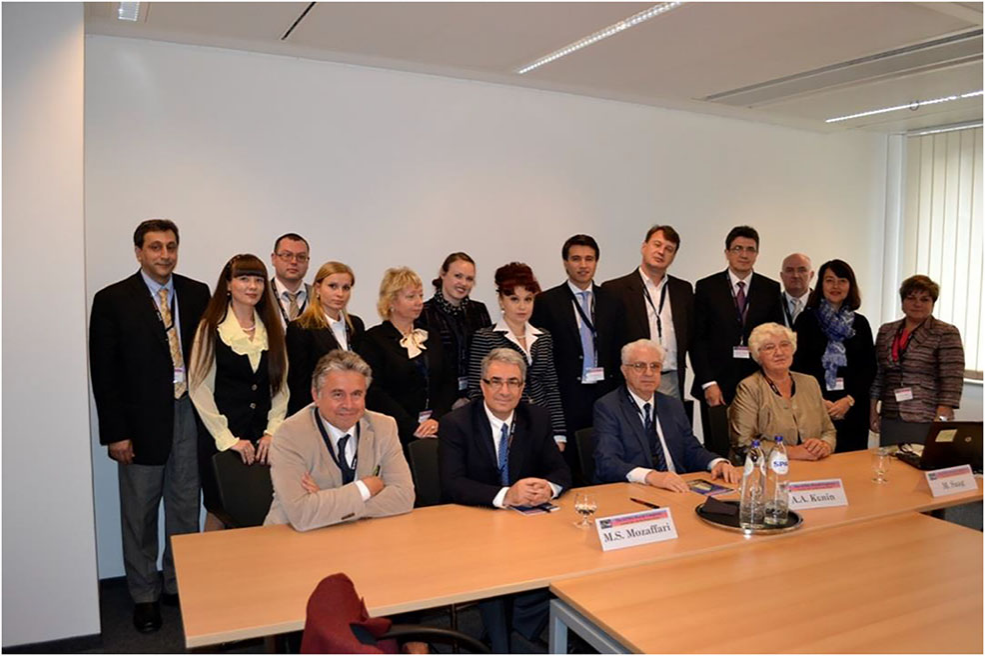


**Fig. 1** EPMA Summit at the EU-Parliament and World Congress, 19–21 September 2013, Brussels, Belgium

**Outlook**

Future projects promoted by the specialised section consider several promising topics such as mild inflammation as the trigger of a cascade of pathologies, microbiome research, application of nanoceria, validation of pathology specific biomarker patterns in body fluids, amongst others.

**References**

1. Kunin AA, Belenova IA, Ippolitov YA, Moiseeva NS, Kunin DA. Predictive research methods of enamel and dentine for initial caries detection. EPMA J. 2013;4(1):19. 10.1186/1878-5085-4-19.
2. Mozaffari MS, Emami G, Khodadadi H, Baban B. Stem cells and tooth regeneration: prospects for personalized dentistry. EPMA J. 2019;10(1):31–42. 10.1007/s13167-018-0156-4.
3. Tachalov VV, Orekhova LY, Isaeva ER, Kudryavtseva TV, Loboda ES, Sitkina EV. Characteristics of dental patients determining their compliance level in dentistry: relevance for predictive, preventive, and personalized medicine. EPMA J. 2018;9(4):379–385. 10.1007/s13167-018-0152-8.
4. Olak J, Nguyen MS, Nguyen TT, Nguyen BBT, Saag M. ‘The influence of mothers’ oral health behaviour and perception thereof on the dental health of their children. EPMA J. 2018;9(2):187–193. 10.1007/s13167-018-0134-x.
5. Kunin A, Polivka J Jr., Moiseeva N, Golubnitschaja O. “Dry mouth” and “Flammer” syndromes-neglected risks in adolescents and new concepts by predictive, preventive and personalised approach. EPMA J. 2018;9(3):307–317. 10.1007/s13167-018-0145-7.
6. Seredin P, Goloshchapov D, Ippolitov Y, Vongsvivut P. Pathology-specific molecular profiles of saliva in patients with multiple dental caries-potential application for predictive, preventive and personalised medical services. EPMA J. 2018;9(2):195–203. 10.1007/s13167-018-0135-9.
7. Beregova TV, Neporada KS, Skrypnyk M, Falalyeyeva TM, Zholobak NM, Shcherbakov OB, Spivak MY, Bubnov RV. Efficacy of nanoceria for periodontal tissues alteration in glutamate-induced obese rats-multidisciplinary considerations for personalized dentistry and prevention. EPMA J. 2017;8(1):43–49. 10.1007/s13167-017-0085-7.
8. Lechner J, Noumbissi S, von Baehr V. Titanium implants and silent inflammation in jawbone-a critical interplay of dissolved titanium particles and cytokines TNF-α and RANTES/CCL5 on overall health? EPMA J. 2018;9(3):331–343. 10.1007/s13167-018-0138-6.
9. Cafiero C, Matarasso S. Predictive, preventive, personalised and participatory periodontology: ‘the 5Ps age’ has already started. EPMA J. 2013;4(1):16. 10.1186/1878-5085-4-16.
10. Golubnitschaja O, Costigliola V and EPMA. General report & recommendations in predictive, preventive and personalised medicine 2012: white paper of the European Association for Predictive, Preventive and Personalised Medicine. EPMA J. 2012;3(1):14. 10.1186/1878-5085-3-14.
11. Golubnitschaja O, Baban B, Boniolo G, Wang W, Bubnov R, Kapalla M, et al. Medicine in the early twenty-first century: paradigm and anticipation – EPMA position paper 2016. EPMA J. 2016;7:23. 10.1186/s13167-016-0072-4.
12. Proceedings of the EPMA World Congress. EPMA J. 2013;5 (Suppl 1):A1–A165.
13. EPMA World Congress 2015. EPMA J. 2016;7(Suppl 1). 10.1186/s13167-016-0054-6
14. Golubnitschaja O, Costigliola V, Grech G. EPMA World Congress: traditional forum in predictive, preventive and personalised medicine for multi-professional consideration and consolidation. EPMA J. 2017;8(Suppl 1):1. 10.1007/s13167-017-0108-4.

**National eHealth System in Republic of North Macedonia - Platform for Preventive, Predictive and Personalized Metabolic Control in Diabetes Patients**

Smokovski I*^1,2^, Sadikario S^3,4^, Cibisev A^2,5^

^1^ University Clinic of Endocrinology, Diabetes and Metabolic Disorders, Skopje, Republic of North Macedonia

^2^ University Goce Delcev, Faculty of Medical Sciences, Stip, Republic of North Macedonia

^3^ University Clinic of Cardiology, Skopje, Republic of North Macedonia

^4^ University Sts Cyrillus and Methodius, Medical Faculty, Skopje, Republic of North Macedonia

^5^ University Clinic of Toxicology and Emergency Internal Medicine, Skopje, Republic of North Macedonia

*Corresponding author: Ivica Smokovski; e.mail: ivica.smokovski@ugd.edu.mk

**Keywords:** National eHealth System, Predictive preventive personalized medicine, Diabetes, Metabolic control, Health economics, Education, Cardiovascular disease, Comorbidities, North Macedonia

**Background**

The diabetes mellitus pandemic is a major challenge for public health, healthcare systems and economies, due to the numerous associated comorbidities [1].

The Republic of North Macedonia is estimated to have one of the highest age-adjusted comparative diabetes prevalences in Europe, mainly due to the estimated high prevalence of type 2 diabetes [2–4].

Several factors have been implicated in such a high prevalence of type 2 diabetes in the country, and its steep rise in the past three decades, including: (1) dietary and lifestyle habits similar to Turkey, the country with the highest age-adjusted comparative diabetes prevalence in Europe; (2) transition from planned to market economy in the last decade of the past century, with sharp rise of unemployment in middle-age populations, and associated psycho-social effects; (3) very high smoking prevalence [3].

The fact that cardiovascular diseases (CVD) are the major cause for morbidity and mortality in the country, and that the majority of diabetes patients would be diagnosed or die from CVD, as well as the fact that the Republic of North Macedonia is categorized as a European country with a very high risk for cardiovascular mortality, further aggravate the situation regarding the very high national diabetes prevalence [3,5,6].

The National eHealth System (NeHS) was introduced in the country on 1 July 2013, covering all citizens across primary, secondary and tertiary healthcare levels [3]. Implementation of the National eHealth System has been praised internationally as a key platform for improving the performance of the national healthcare system [7].

In order to manage the national diabetes burden, several initiatives have been undertaken, such as integration of diabetes care module in NeHS since 1 Jan 2015, and formation of National Diabetes Committee according to the Law on Healthcare since February 2015. In addition, international guidelines have been adopted as national guidelines for diabetes care and treatment, and were published in the Official Journal of Republic of North Macedonia, where laws and bylaws are published, further emphasizing the importance attributed to diabetes as a nationwide condition.

The National eHealth System module for diabetes care was upgraded with possibility to record metabolic parameters, such as glycemic control, weight, lipid profile, blood pressure and diabetes complications, thus strengthening the role of NeHS as a critical resource for monitoring of diabetes care in the country.

Furthermore, NeHS has become essential platform for preventive, predictive and personalized metabolic control in diabetes patients, covering the total population of Republic of North Macedonia.

Taking into consideration that diabetes has been a huge healthcare and socio-economic burden for the country, the analysis of data on metabolic control parameters in diabetes patients derived from NeHS would be of utmost importance.

Since there are no different targets for metabolic control in adult type 1 and insulin treated type 2 diabetes patients, data on metabolic parameters in insulin treated diabetes patients derived from NeHS would be cumulatively analyzed and reported.

The concept of preventive, predictive and personalized medicine (PPPM) has emerged as the focal point of efforts in healthcare aimed at controlling the prevalence and management of non-communicable chronic diseases, including diabetes [8–11]. The management of diabetes and the critical role of PPPM in modernization of healthcare have been acknowledged as priorities by global and regional organizations and health-related institutions such as the Organization of the United Nations, the European Union and the National Institutes of Health [8–11].

The possible predictive role of novel biomarkers in diabetic complications, particularly nephropathy, has been reported, with the possibility for predictive diagnosis and targeted preventive measures [12]. In addition, a nomogram for prediction of 3-year personalized risk of type 2 diabetes mellitus based on age, body mass index, fasting blood glucose, low-density lipoprotein cholesterol, high-density lipoprotein cholesterol, and triglycerides was developed; and suboptimal health status was identified as an independent risk factor for type 2 diabetes mellitus in a community-based cohort [13,14].

This would be the first study to evaluate the role of NeHS in the Republic of North Macedonia as a platform for Preventive, Predictive and Personalized Metabolic Control in diabetes patients, through analysis of metabolic parameters in insulin treated diabetes patients.

**Methods**

The National eHealth System, covering the total population of the country across all three healthcare levels, plans to use ICD-10 (Tenth Revision of International Classification of Diseases) to search for living patients with codes E10–E14 in their Electronic Healthcare Records (EHR), who are on insulin treatment and with any data for metabolic parameters in their EHR.

Since the entry of metabolic parameters in EHR is not mandatory, the number and proportion of insulin treated diabetes patients with any data for metabolic parameters out of all insulin treated diabetes patients are to be identified (*n*, %), with a cut-off date of 1 May 2017.

In addition, number and percentage of insulin treated diabetes patients with HbA1c <7% (adequate glycemic control), 7–7.9% (inadequate glycemic control) and ≥ 8% (poor glycemic control) are to be identified, according to the current Guidelines [15].

The number and percentage of insulin treated diabetes patients with body mass index (BMI) <25 kg/m^2^ (normal weight patients), 25–29.9 kg/m^2^ (overweight patients) and ≥ 30 kg/m^2^ (obese patients) are to be identified.

Achievement of lipid profile targets is to be evaluated through identification of number and percentage of insulin treated diabetes patients with recommended target values for total cholesterol (TC), LDL cholesterol (LDL) and triglycerides (TG); namely with TC <5 mmol/l. LDL < 2.6 mmol/l, and TG < 1.7 mmol/l, respectively.

Finally, the number and percentage of insulin treated diabetes patients with systolic blood pressure (SBP) <140 mmHg (adequate SBP) and with diastolic blood pressure (DBP) <90 mmHg (adequate DBP) are to be identified.

**Results**

Previous publications with data derived from NeHS have reported the total number of diagnosed diabetes patients in the Republic of North Macedonia to be 84,568 (57.3% females), with a mean age of diagnosed diabetes patients of 62.6 ± 12.5 years [4]. The national prevalence of diagnosed diabetes cases in the total population was 4.0%, whereas it was 5.0% 20–79 years ago [4]. The recent International Diabetes Federation (IDF) Diabetes Atlas estimated total diabetes cases, including both diagnosed and undiagnosed, for the country to be 175,100 (95% Confidence Interval (CI) 131,200–218,200), and 20–79 years ago it was 11.2% (95% CI 8.4–13.9%) and diabetes age-adjusted comparative prevalence in population 20–79 years of 9.3% (95% CI 6.8–11.5%) [2].

Furthermore, the IDF Diabetes Atlas has emphasized that the estimation of total diabetes prevalence study in the Republic of North Macedonia was done based on a study conducted within the past five years, further strengthening the accuracy of the estimation [2].

Previous publications with data derived from NeHS have reported the total number of insulin treated diabetes patients in the country to be 37,011 patients, or 43.8% of all diagnosed diabetes patients [3].

As the entry of metabolic parameters in individual EHRs of NeHS remains optional, it is expected that the majority of insulin treated diabetes patients would have no data for a single metabolic parameter. Of those who have, it would be interesting to identify the results of national metabolic control, taking into account the results from the GUIDANCE Study, a retrospective analysis of EHRs of 7597 diabetes patients from eight major European countries, that found the majority of diabetes patients to be in inadequate or poor glycemic control, with percentage of patients achieving target HbA1c <7% to be 60% in Belgium (*n* = 1044), 65% in France (*n* = 1056), 49% in Germany (*n* = 959), 53% in Ireland (*n* = 950), 36% in Italy (*n* = 984), 71% in Netherlands (*n* = 1021), 57% in Sweden (*n* = 550) and 39% in United Kingdom (*n* = 1033) [16].

**Conclusions and Expert recommendations**

This is the first study to analyse the metabolic control parameters in insulin treated diabetes patients from the Republic of North Macedonia derived from the NeHS, covering the total population of the country. Furthermore, it is the first study to evaluate the significance of NeHS as a national platform for preventive, predictive and personalized metabolic control in diabetes patients.

The National eHealth System has been established as a platform for predictive diabetes care, as it enables monitoring of metabolic control parameters and the associated predicted cardiovascular risk in diabetes patients, taking into consideration that CVDs are the primary cause of morbidity and mortality in diabetes patients.

The National eHealth System has also been established as a platform for preventive diabetes care, since it enables monitoring of development of micro-vascular complications in diabetes patients, and through monitoring of glycemic control and risk factors, it provides directions for focusing preventive activities to avoid or delay diabetic complications.

Finally, the National eHealth System has served as a platform for personalized diabetes care, since it enables personalized diabetes care that is based on the glycemic control and concomitant conditions and treatments; NeHS data are available to care-givers across the spectrum of healthcare levels (primary, secondary, tertiary), and there is a potential to add new scientifically sound and approved biomarkers to NeHS nationwide at a patient level in the future, to further personalize diabetes care.

It is expected that this first study of metabolic control in insulin-treated diabetes patients derived from the NeHS would suggest a need for improvement of glycemic, weight, lipid and blood pressure control. Monitoring of metabolic control parameters through NeHS is expected to confirm its value as a platform for predictive, preventive and personalized metabolic control in insulin treated diabetes patients at an individual level for every citizen with diabetes in the country, thus contributing to the overall, national metabolic control.

**References**

1. Duarte AA, Mohsin S, Golubnitschaja O. Diabetes care in figures: current pitfalls and future scenario. EPMA J. 2018;9(2):125–131.
2. International Diabetes Federation. IDF Diabetes Atlas. 9th ed. Brussels, Belgium: International Diabetes Federation. 2019; http://www.idf.org/diabetesatlas [accessed 10 December 2019].
3. Smokovski I, Milenkovic T, Trapp C, Mitov A. Diabetes care in Republic of Macedonia: challenges and opportunities. Ann Global Health 2015;81(6):792–802.
4. Smokovski I, Milenkovic T, Nam H Cho. First stratified diabetes prevalence data for Republic of Macedonia derived from the National e-Health System. Diabetes Res Clin Pract. 2018;143:179–183.
5. Perk J, De Backer G, Gohlke H, Graham I, Reiner Z, Verschuren M, et al. European Guidelines on cardiovascular disease prevention in clinical practice (version 2012). The Fifth Joint Task Force of the European Society of Cardiology and Other Societies on Cardiovascular Disease Prevention in Clinical Practice. Eur Heart J. 2012;33(13):1635–701.
6. Ministry of Health of RoM. Program for prevention of cardiovascular diseases 2015. Official J RoM. 2014;196:76e7.
7. Health Consumer Powerhouse. Euro Health Consumer Index Report; 2014. https://healthpowerhouse.com/media/EHCI-2014/EHCI-2014-report.pdf [accessed 10 December 2019].
8. Golubnitschaja O, Kinkorova J, Costigliola V. Predictive, Preventive and Personalised Medicine as the hardcore of ‘Horizon 2020’: EPMA position paper. EPMA J. 2014;5(1):6.
9. Golubnitschaja O, Costigliola V. European strategies in predictive, preventive and personalised medicine: highlights of the EPMA World Congress 2011. EPMA J. 2011;2(4):315–32.
10. Golubnitschaja O, Costigliola V. General report & recommendations in predictive, preventive and personalised medicine 2012: white paper of the European Association for Predictive, Preventive and Personalised Medicine. EPMA J. 2012;3(1):14.
11. Golubnitschaja O. Time for new guidelines in advanced healthcare: the mission of The EPMA Journal to promote an integrative view in predictive, preventive and personalized medicine. EPMA J. 2012; 3(1):5.
12. Issa YA, Abd ElHafeez SS, Amin NG. The potential role of angiopoietin-like protein-8 in type 2 diabetes mellitus: a possibility for predictive diagnosis and targeted preventive measures? EPMA J. 2019;10(3):239–248.
13. Wang K, Gong M, Xie S, Zhang M, Zheng H, Zhao X, Liu C. Nomogram prediction for the 3-year risk of type 2 diabetes in healthy mainland China residents. EPMA J. 2019;10(3):227–237.
14. Ge S, Xu X, Zhang J, Hou H, Wang H, Liu D, Zhang X, Song M, Li D, Zhou Y, Wang Y, Wang W. Suboptimal health status as an independent risk factor for type 2 diabetes mellitus in a community-based cohort: the China suboptimal health cohort study. EPMA J. 2019;10(1):65–72.
15. American Diabetes Association. Standards of Medical Care in Diabetes, 2020. Diabetes Care. 2020;43(Suppl 1): s1-s212.
16. Stone MA, Charpentier G, Doggen K, Kuss O, Lindblad U, Kellner C, Nolan J, Pazderska A, Rutten G, Trento M, Khunti K; GUIDANCE Study Group. Quality of care of people with type 2 diabetes in eight European countries: findings from the Guideline Adherence to Enhance Care (GUIDANCE) study. Diabetes Care. 2013;36:2628–38.

**Educating High School Students in the West Bohemia Region: PPPM approach in Stroke management**

Stibrana K^1^, Stela J^1^, Polivka J*^2,3^, Rohan V^2,3^, Polivka J Jr.^3, 4^

^1^ Faculty of Medicine in Pilsen, Charles University, Czech Republic

^2^ Department of Neurology, Faculty of Medicine in Pilsen, Charles University, Czech Republic

^3^ Department of Neurology, University Hospital Pilsen, Czech Republic

^4^ Department of Histology and Embryology and Biomedical Center, Faculty of Medicine in Pilsen, Charles University, Czech Republic

*Corresponding author: Jiri Polivka, MD, Department of Neurology, University Hospital Pilsen, Czech Republic; e.mail: POLIVKA@fnplzen.cz

**Keywords:** Targeted prevention, Preventive, predictive, personalized medicine (PPPM), Educating the public, Education, Stroke, High school students, Primary prevention, Personalized medicine

**Introduction**

Strokes have serious health and social consequences worldwide [1–3]. Education and lifestyle adjustment are key tools for the successful prevention of many civilization diseases. Furthermore, education has an important role in the detection of clinical signs and symptoms of illness and in choosing the right procedure. Stroke is an ideal topic for education and prevention [4–7], optimally in a young population because a “young stroke” is by no means a rare occurrence [8] and the long-term effect of preventive measures can pay off [1,9,10].

The aim of this project was to obtain information about the sample’s previous awareness regarding strokes and learn about the lifestyle and health status of a young population sample and that of their family members. Furthermore, we set out to educate a young community sample about strokes.

**Methods**

Medical students who completed a neurology course and passed special training on the topic of strokes were in charge of giving special preventive lectures to high school students. All activities were performed in school classes (each for 25–32 students). After a short introduction and explanation of the aim and purpose of the educational activity, the students were asked to fill in an anonymous questionnaire online/a printed questionnaire. The questionnaire consists of three parts: (a) Their general knowledge regarding strokes (definition, principles, etiology, signs and symptoms, consequences, treatment and prevention options) and how to react in case of a suspected stroke (b) Personal health and lifestyle information, (c) Questions regarding the students’ family members’ health. A presentation on strokes and a discussion followed. The informed students completed the first part of the questionnaire and were then asked to take a quiz 14–21 days later.

**Results**

A total number of 16 sessions for 356 students (150 men, 206 women), age range 15–18 years of age, took place between April and May 2019 in four different high schools in the West Bohemia region. Results showed that only 33% of students had previous knowledge on the topic of strokes and stroke signs and symptoms, only 11% knew how to respond to a suspected stroke and 31% of students knew one or more stroke risk factors; 28% are cigarette smokers, 39% confirm occasional alcohol consumption and 10% are frequent alcohol drinkers; 47% of the females in the sample did not know the relationship between smoking and hormonal contraception. 37% did not know or did not care about their glucose and lipid metabolism. Improved knowledge of the topic of strokes was detectable later in 81% students, 37% discussed it within their family and 7% showed the presentation to their family members. Of the students, 87% considered the education activity beneficial, 7% had a neutral response to it and 5% perceived it as a useless harassment. In addition, 74% agreed with the fact that a healthy lifestyle is of importance, 18% were not sure and only 8% of the students considered any measures and lifestyle changes useless.

**PPPM relevant conclusions**

As far as predictive medicine is concerned, this activity enables the identification of a risk group in a young population (individual risk factors, inappropriate lifestyle, family risk and burden). Primary prevention and providing information on preventive measures are the main goals of this activity; especially to populations at risk. The realization of this educational activity is of importance for encouraging lifestyle adjustments among the young population and embodies the potential of information trickling down to their family members. The principles of personalized medicine can be achieved with the dissemination of knowledge and by educating the public about strokes and the optimal measures to take in case of a stroke [3]. This type of educational activity is not expensive, is well feasible and could be used on young populations worldwide.

**Acknowledgement**

Supported by MH CZ-DRO (University Hospital Plzen-FNPl, 00669806); supported by the Charles University Research Fund (Progres Q39).

**References**

1. Pandian JD, Gall SL, Kate MP, Silva GS, Akinyemi RO, Ovbiagele BI, et al. Prevention of stroke: a global perspective. Lancet Lond Engl. 2018;392:1269–78.
2. Polivka J, Krakorova K, Peterka M, Topolcan O. Current status of biomarker research in neurology. EPMA J. 2016;7:14.
3. Polivka J, Polivka J, Rohan V. Predictive and individualized management of stroke-success story in Czech Republic. EPMA J. 2018;9:393–401.
4. Matsuzono K, Yokota C, Takekawa H, Okamura T, Miyamatsu N, Nakayama H, et al. Effects of stroke education of junior high school students on stroke knowledge of their parents: Tochigi project. Stroke. 2015;46:572–4.
5. Ilunga Tshiswaka D, Sikes LE, Iwelunmor J, Ogedegbe G, Williams O. Transferring Stroke Knowledge from Children to Parents: A Systematic Review and Meta-Analysis of Community Stroke Educational Programs. J Stroke Cerebrovasc Dis. 2018;27:3187–99.
6. Umar AB, Koehler TJ, Zhang R, Gilbert V, Farooq MU, Davis AT, et al. Stroke knowledge among middle and high school students. J Int Med Res. 2019;47:4230–41.
7. Simmons C, Noble JM, Leighton-Herrmann E, Hecht MF, Williams O. Community-Level Measures of Stroke Knowledge among Children: Findings from Hip Hop Stroke. J Stroke Cerebrovasc Dis. 2017;26:139–42.
8. Polivka Jr., Polivka J, Pesta M, Rohan V, Celedova L, Mahajani S, et al. Risks associated with the stroke predisposition at young age: facts and hypotheses in light of individualized predictive and preventive approach. EPMA J. 2019;10:81–99.
9. Meschia JF, Bushnell C, Boden-Albala B, Braun LT, Bravata DM, Chaturvedi S, et al. Guidelines for the primary prevention of stroke: a statement for healthcare professionals from the American Heart Association/American Stroke Association. Stroke. 2014;45:3754–832.
10. Steiger N, Cifu AS. Primary Prevention of Stroke. JAMA. 2016;316:658–9.

**Innovative Center for Preventive and Personalized Medical Services: Concepts, Challenges and Potential Solutions**

Safonicheva O*^1^, Ovchinnikova M^1^, Golubnitschaja O^2^

^1^I.M. Sechenov First Moscow State Medical University, Russian Federation

^2^Predictive, Preventive and Personalised (3P) Medicine, Department of Radiation Oncology, University Hospital Bonn, Rheinische Friedrich-Wilhelms-Universität Bonn, Germany

*Corresponding author: Prof. Dr. O. Safonicheva, I.M. Sechenov First Moscow State Medical University, Russian Federation; e.mail: safonicheva.o@mail.ru

**Keywords:** Predictive preventive personalized medicine (PPPM), infrastructure project, new concept, center of attraction, salutogenic design, non-communicable diseases, ICT, paradigm change, public health, implementation, innovative services, optimized health management, multi-level diagnostics, life-style, modifiable risks, infrastructure, e-medicine, healthcare economics

**Introduction**

In the early twenty-first century, high levels of urbanization and sub-optimal life-styles have strongly contributed to epidemics of non-communicable diseases (NCDs) [1–6]. Environment and lifestyle are decisive factors in human health. According to WHO (2015), “the epidemic of lifestyle diseases is a much greater threat to public health than any other epidemic known to humanity”.

The paradigm shift from reactive to predictive, preventive and personalized medicine (PPPM) in public healthcare is evidence-based justified and effectively promoted by the European Association for Predictive, Preventive and Personalised Medicine (EPMA, Brussels) [7]. The Russian Project of the National Technological Initiative follows the PPPM concepts to effectively implement the paradigm change to improve heathcare services for the population and healthcare economy [8].

**Aims**

To create a new model for hospitals to implement innovative PPPM concepts and services – see Fig. 1.
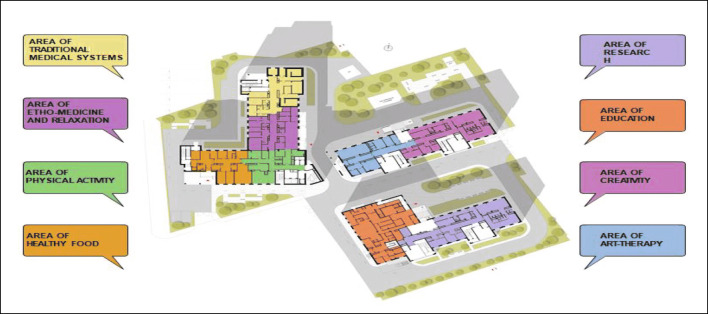


**Fig. 1** Preliminary concepts of PPPM Center implementation at the stage of transition from reactive to predictive, preventive and personalized services

**Concepts, Challenges and Potential Solutions**

At the stage of transition to PPP Medicine, Innovative Health and Development Centers should become the Centers of Attraction for the whole family.

We consider extending the list of Departments in the PPPM Center to include healthcare architecture, salutogenic design of interiors to cover research, education, creativity and other solutions for health.

I. The Department of Research may propose preclinical predictive diagnosis with different tests for detection of mental, physica and somatic health. The cardinal objective of this Department is the development of approaches and technologies for recognizing the so-called suboptimal health conditions before disease appearance and the application of scientifically based interventions countering identified risks. Since the main goal of the PPPM Center is to promote health and reduce disease burden, this Department has to accumulate, analyze and validate data on healthy people to provide big data analytics and create the intervention platform with personalized preventive programs for healthy lifestyle, healthy food, ergonomics for professional longevity, etc.
  The novel examination protocol includes: neuropsychological study for examination of cognitive, emotional status, memory tests, stress factors; as well as clinical, laboratory, instrumental and genetic tests to search for the markers of early NCDs stages.
II. The Department of Education has to organize education throughout life—from childhood to “silver age”: Master classes and courses may cover the practical information in personalized health management, in holistic medicine with fundamental laws to support mental, physical and spiritual health for creativity and professional realization. This Department may present the conceptions of balance and happiness and include healthy rooms with ergonomic equipment.
III. The Department of art-therapy and communications may include spaces for self-expression in paint, music and dance therapy therapy for development of brain plasticity.
IV. The Department of Traditional Medical Systems enables enhancing the knowledge towards the disease prevention and treatment. From a predictive and preventive medicine point of view, Ethno-medicine provides a unique expertise for recognizing the pre-clinical health conditions before a clinical manifestation of severe pathologies.
V. The Department of Creativity may propose solutions for personalized fashion style.
VI. The Department of Physical Activity analyzes psychological and physical data to prescribe personalized physical programs to strengthen the systemic mechanisms for disease prevention and cognition improvement. Different categories of individuals can receive scientifically based programs in the smart halls with functional space for fitness and movement skills development.
VII. The Department of Healthy Food can organize master-classes in cooking for healthy life, advocate healthy nature food, dietetics for participants, according to individual constitution approach.

**Conclusions and outlook**

To achieve the paradigm change from reactive to preventive, predictive and personalized medicine, it is essential to establish innovative PPPM Centers based on evidence-based scientific achievements, multi-professional expertise, ICT solutions and robust infrastructure strongly supported by adequate budgets and supportive political decisions to create attractive conditions and market opportunities for the healthcare industry [9,10]. Involvement of the above-described Center as the institutional member of the European Association for Predictive, Preventive and Personalized Medicine (EPMA, Brussels) is foreseen to develop a long-term European-Russian collaboration within the multi-professional and international network and to effectively implement PPPM concepts in the Russian Federation.

**References**

1. Polivka J Jr., Altun I, Golubnitschaja O. Pregnancy associated breast cancer: the risky status quo and new concepts of predictive medicine. EPMA J. 2018;9(1):1–13. 10.1007/s13167-018-0129-7.
2. Polivka J Jr., Polivka J, Pesta M, Rohan V, Celedova L, Mahajani S, Topolcan O, Golubnitschaja O. Risks associated with the stroke predisposition at young age: facts and hypotheses in light of individualized predictive and preventive approach. EPMA J. 2019;10(1):81–99. 10.1007/s13167-019-00162-5.
3. Duarte AA, Mohsin S, Golubnitschaja O. Diabetes care in figures: current pitfalls and future scenario. EPMA J. 2018;9(2). 10.1007/s13167-018-0133-y
4. Bubnov R, Polivka J Jr, Zubor P, Konieczka K, Golubnitschaja O. Pre-metastatic niches" in breast cancer: are they created by or prior to the tumour onset? “Flammer Syndrome” relevance to address the question. EPMA J. 2017;8(2):141–57. 10.1007/s13167-017-0092-8
5. Golubnitschaja O and Costigliola V. Predictive, preventive and personalised medicine as the medicine of the future: anticipatory scientific innovation and advanced medical services. In: Nadin M (ed) Anticipation and medicine. Cham: Springer; 2016. pp 69–85.
6. Golubnitschaja O. Flammer syndrome – from phenotype to associated pathologies, prediction, prevention and personalization, vol 11. Cham: Springer; 2019. 10.1007/978-3-030-13550-8
7. Golubnitschaja O, Baban B, Boniolo G, Wang W, Bubnov R, Kapalla M, Krapfenbauer K, Mozaffari M, Costigliola V. Medicine in the early twenty-first century: paradigm and anticipation – EPMA position paper 2016. EPMA J. 2016;7:23, 10.1186/s13167-016-0072-4.
8. Safonicheva O, Martynchik S. Challenges of scientific platform for medical sciences “Preventive Protection”: technological solutions. Success Nat Sci History J. 2015;3: 102–106.
9. Golubnitschaja O, Andrews RJ. Patient-centered care: making the modern hospital truly modern. In: Latifi R (ed) The modern hospital: patients centered, disease based, research oriented, technology driven. Cham: Springer; 2018.
10. Barrett M et al. Artificial intelligence driven patient self-care: a paradigm shift in chronic heart failure treatment. EPMA J. 10(4):445–464. 10.1007/s13167-019-00188-9.

**Vision of the essential extension of health-related services provided in the hospital as an initial step towards future healthcare and practical realization of predictive, preventive and personalized medicine**

Lapuníková M^1^, Sklenková I^1^, Bečková Z^1^, Ďurajová V^1^, Kubáň J^2^, Kapalla M*^1,2^

^1^ F.D. Roosvelt University Hospital with Policlinics, Banská Bystrica, Slovak Republic

^2^ PPPM Centre, s.r.o., Ružomberok, Slovak Republic

*Corresponding author: Dr. Marko Kapalla, FNsP FDR, Nám. L. Svobodu 1, Banská Bystrica, Slovak Republic; e.mail: marko.kapalla@gmail.com

**Keywords:** predictive preventive personalized medicine (PPPM / 3PM), vision, future healthcare, disease care, education, hospital, laboratory diagnostics, health support, health literacy

**Introduction**

F.D. Roosevelt University Hospital with Policlinic (FDRH) in Banská Bystrica, Slovakia [1] is one of the largest hospitals in Slovakia, ISO 9001 certified, having almost 1000 beds, approximately 2300 employees, and providing medical services to as many as half a million patients annually. The management team of the Hospital permanently monitors the trends in the development of the healthcare worldwide and creates its long-term development strategy being familiar with the concept of the predictive, preventive, and personalized medicine (3PM), promoted by the European Association for Predictive, Preventive and Personalised Medicine (EPMA) since 2009 [2–10].

Working well in consensus with the 3PM concepts, FDRH with the approval from the Ministry of Health of the Slovak Republic, became an Institutional member of the EPMA, following the general vision to accomplish the pioneering mission and bringing practical 3PM aspects into daily practice that will benefit the patient and healthcare as a whole in Slovakia. Keeping this in mind, FDRH encourages medical facilities and organizations in other regions of Slovakia to join our initiative for better coordination of joint efforts in tight collaboration with the international professional network led by EPMA on a global scale.

**Implementation of 3 PM concepts into daily practice in steps**

- The first step

Keeping in mind the complexity of the objectives, FDRH decided, in the first step, to focus on significantly extending the range of tests in our Clinical laboratory complex that would provide the value added to the spectrum of the routine tests [11]. FDRH expects to equip the laboratory diagnostics with powerful analytical technologies for clinical biochemistry, clinical microbiology, and toxicology, such as high performance liquid chromatography (HPLC), liquid chromatography coupled to mass spectrometry (LCMS), gas chromatography/mass spectrometry (GCMS), atomic absorption spectrometry (AAS), matrix-assisted laser desorption/ionization mass spectrometry with time-of-flight detector (MALDI-TOF), as well as other most recent analytical methods in international collaboration with other EPMA Institutional members. Furthermore, FDRH creates cooperation with national and regional partners who would provide analytical services toward health relevant environmental factors [12]. The complementary services to related medical units in the context of 3PM are also under consideration such as services related to healthy nutrition, relax and sport in the mountains, and wellness. To achieve this, we plan to gradually transform one of our external hospital facility into a climatic spa. This option creates a wide spectrum of opportunities for an international collaboration within the European Union and worldwide [13].

- Follow-up step

In the next step we plan to significantly increase capacities in medical imaging and in multi-level diagnostics [9, 10, 14], thus expanding the overall predictive diagnostic potential of the hospital.

- Essential step forward

The essential step forward is a continual education of the patients and individuals interested in advanced healthcare supporting their health status. This aspect has attracted particular attention due to the worldwide pandemic of COVID-19, as declared by WHO on 11 March 2020 [15]. any medical professional would disagree that having had the concept of 3PM been implemented in healthcare, including improved health literacy, the current pandemic situation would have been much easier to deal with and more effectively managable.

**The vision**

The vision we have is to offer, in a supportive international cooperation, the health-focused services in predictive diagnostics, primary prevention and health-enhancing services to the patients, through which a wide spectrum of medical and other professionals would take care of individual patients at either particular stages of their diseases, or the clients who do not suffer of any apparent health problems or individuals with suboptimal health status [16] who are interested in an assessment of their health in order to predict and prevent potential health deterioration well before the manifestation of a pathology that is later difficult to reverse or treat.

We expect that by implementing 3PM as an integral part of our professional medical care, parallel focusing on health-supporting services, alongside the standard therapeutic procedures, we will, in the long run, significantly reduce healthcare costs and make the future healthcare more sustainable and evolving (Fig. 1).
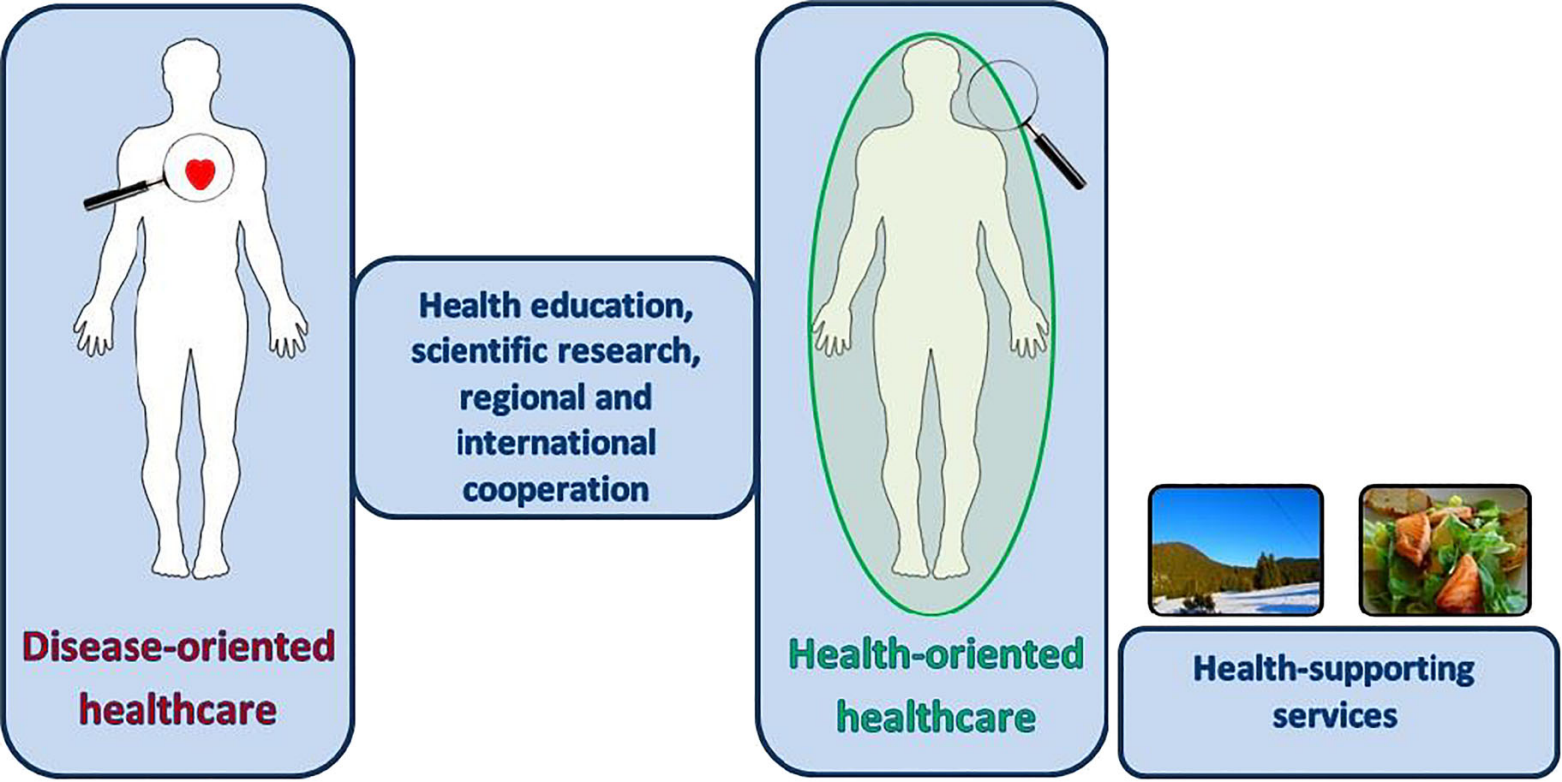


**Fig. 1** A simple illustration of our vision in practical implementation of PPPM in the hospital where diseases-oriented healthcare is complemented by health-oriented healthcare and health supporting services. Both pillars are entangled by other essential elements—health education, scientific research, and regional and international cooperation.

**Conclusion**

In the current situation of unprecedented pressure on the quality of the healthcare system, caused by the pandemic of COVID-19, we realized that it is time to significantly change the healthcare system and transform it to be more viable and pro-active. We understand the worldwide outbreak of SARS-CoV-2 as an ultimate “wake-up call” that essentially needs to be answered. We are ready to complement, in parallel, the EPMA’s visions [11,17,18] of specialized 3PM centers by implementing the 3PM attitude directly within the hospital structure, thus bringing future 3PM healthcare closer to the patients as well as healthy individuals. Such an evolutionary step would, of course, require a broader collaboration at both the national and international levels. The platform for such collaboration exists, so let us get started.

**References**

1. https://www.fnspfdr.sk/, Accessed March 31, 2020
2. https://epmanet.eu, Accessed March 31, 2020
3. Golubnitschaja O, Costigliola V, EPMA General report & recommendations in predictive, preventive and personalised medicine 2012: white paper of the European association for predictive, preventive and personalised medicine. EPMA J. 2012;1;3(1):14. 10.1186/1878-5085-3-14
4. Golubnitschaja O, Costigliola V, EPMA. EPMA summit 2014 under the auspices of the presidency of Italy in the EU: professional statements. EPMA J. 2015;6(1):4. 10.1186/s13167-015-0026-2
5. Golubnitschaja O, Baban B, Boniolo G, Wang W, Bubnov R, Kapalla M, Krapfenbauer K, Mozaffari M, Costigliola V. Medicine in the early twenty-first century: paradigm and anticipation – EPMA position paper 2016. EPMA J. 2016;7:23, 10.1186/s13167-016-0072-4
6. Golubnitschaja O, Watson ID, Topic E, Sandberg S, Ferrari M, Costigliola V. Position paper of the EPMA and EFLM: a global vision of the consolidated promotion of an integrative medical approach to advance health care. EPMA J. 2013;4:12 10.1186/1878-5085-4-12
7. Golubnitschaja O, Andrews RJ. Patient-centered care: making the modern hospital truly modern. The modern hospital: patients centered, disease based, research oriented, technology driven. In: Latifi R, editor. Cham: Springer; 2019.
8. Golubnitschaja O, Lemke HU, Kapalla M, Kent A. Designing for personalisation in predictive and preventive medicine. In: Kuksa I, Fisher T, editors. Design for personalisation. New York: Taylor and Francis; 2017. pp. 150–169. 10.4324/9781315576633
9. Golubnitschaja O, Stolzenburg-Veeser L, Avishai E, Costigliola V. Wound healing: proof-of-principle model for the modern hospital - patient stratification, prediction, prevention and personalisation of treatment. In: Rifat L, editor. The modern hospital: patients centered, disease based, research oriented, technology driven. Cham: Springer; 2019.
10. Golubnitschaja O. Flammer syndrome – from phenotype to associated pathologies, prediction, prevention and personalisation. Cham: EPMA/Springer; 2019. 10.1007/978-3-030-13550-8
11. Kapalla M, Kubáň J. Extension of laboratory medicine to provide complex health assessment in the context of PPPM. EPMA J. 2017; 8(Supl. 1)23–24. 10.1007/s13167-017-0108-4
12. Kapalla M, Kubáň J. How to estimate health-related costs: Economic aspect of healthy life style and its importance for PPPM. EPMA J. 2014;5(Suppl 1): A103. 10.1186/1878-5085-5-S1-A103
13. Costigliola V, Kapalla M, Kubáň J, Golubnitschaja O. EPMA initiative for effective organization of medical travel: European concepts and criteria. EPMA J. 2016;7(Suppl 1):9, A39:19–20. 10.1186/s13167-016-0054-6
14. Bauer J, Hoq N, Mulcahy J, Tofail SAM, Gulshan F, Silien C, Podbielska H, Akbar M. Implementation of artificial intelligence and non-contact infrared thermography for prediction and personalized automatic identification of different stages of cellulite. EPMA J. 2020;11:17–29. 10.1007/s13167-020-00199-x
15. WHO, WHO Director-General’s opening remarks at the media briefing on COVID-19 - 11 March 2020, https://www.who.int/dg/speeches/detail/who-director-general-s-opening-remarks-at-the-media-briefing-on-covid-19%2D%2D-11-march-2020, Accessed on March 31st, 2020
16. Yan YX, Liu YQ, LiM, Hu PF, Guo AM, Yang XH, Qiu JJ, Yang SS, Shen J, Zhang LP, Wang W. Development and evaluation of a questionnaire for measuring suboptimal health status in urban Chinese. J Epidemiol. 2009;19(6):333–341. 10.2188/jea.je20080086
17. Kapalla M, Kubáň J, Costigliola V, Golubnitschaja O. Vision of the first EPMA center for predictive, preventive and personalized medicine in Europe. EPMA J. 2014;5(Suppl 1):A153. 10.1186/1878-5085-5-S1-A153
18. Kapalla M, Kubáň J. Focus of PPPM centre & block scheme of PPPM centre. https://pppmc.sk, Accessed March 31, 2020

## Interdisciplinary 3PM approach

**Plastic surgery: Is an advanced approach by 3P medicine on the horizon?**

Tsygankova V^1^, Sokolovskiy A^2^, Tsygankova N^3^, Golubnitschaja O*^4^

^1^Medical University of Vienna, Vienna, Austria

^2^Goethe University Frankfurt, Frankfurt am Main, Germany

^3^ASTRO, Clinic of aesthetic plastic surgery, Obninsk, Russian Federation

^4^Predictive, Preventive and Personalised (3P) Medicine, Department of Radiation Oncology, University Hospital Bonn, Friedrich-Wilhelms-University Bonn, Germany

*Corresponding author: Prof. Dr. Olga Golubnitschaja, Predictive, Preventive and Personalised (3P) Medicine, Department of Radiation Oncology, University Hospital Bonn, Rheinische Friedrich-Wilhelms-Universität Bonn, Venusberg-Campus 1, 53127 Bonn, Germany; e.mail: Olga.Golubnitschaja@ukbonn.de

**Keywords:** predictive preventive personalised medicine (3PM) plastic surgery, reconstructive surgery, aesthetic surgery, cosmetic surgery, rejuvenation, complications, post-treatment sustainability, life-quality, healthcare economy, individualised patient profile, patient stratification, chronic non-healing wounds, biologic age, true-personalised, treatment algorithm, multi-level diagnostics, non-modifiable risk, preventable risk, biomarker-panel, modelling, disease management

**Introduction**

Plastic surgery is a surgical area which can be divided into two categories: reconstructive and aesthetic surgery. While reconstructive surgery aims to reconstruct a damaged (post-traumatic, cancer-removal) body site or to improve its functioning, the main purpose of aesthetic surgery is improving the appearance. As an elective surgery, cosmetic surgery has a highly specific risk/benefit ratio.

The predictive and prognostic approach tailored to the individualised patient profile is highly desirable in the area, in order to develop perioperative stratification tolls, predict postoperative complications and calculate final treatment costs.

An evidence-based approach is essential to provide optimal care in plastic surgery and protect interests of all key players in the healthcare system (the treating plastic surgeon, the patient him/herself and insurance).

The aim of this study is to identify which kind of parameters are to be considered for individualised patient profiling, evidence-based patient stratification and consequently true-personalised treatment algorithms.

**Medline search results**

In September 2019, we performed a Medline search to find out how extensively the principles of 3PM, as well as corresponding tools and approaches, are explored and utilised by the plastic surgery community. The search results are summarized in the Table 1.

**Table 1.** Medline search results utilising the below listed keywords; corresponding publication numbers are presented

**Table:** 

	Plastic surgery
Predictive	1189
preventive	583
personalised	142
stratification	202
Biomarker	1837
Genomics	1135
Proteomics	147
Metaboomics	24
predictive preventive personalised medicine	2 (EPMA Journal)
Multiomics	1 (EPMA Journal)

**3PM related conclusions and expert recommendations**

Obviously, the area of plastic surgery still lacks evidence-based 3PM approaches, including multi-omic biomarker panels for predictive diagnostic purposes, targeted prevention and true-personalised treatment algorithms.

Non-modifiable risk factors include a genetic predisposition to hypertrophic or keloid scarring, skin type (pigmentation, elasticity, thickness, sebaceous quality, location), Ehlers–Danlos syndrome, Pseudoxantoma elasticum, Cutis laxa, Ataxia-teleangiectasia, Klinefelder syndrome (diminished fibrinolysis, high levels of the fibrinolysis inhibitor PAI-1), disorders of the immune system (Leukocyte adhesion deficiencies (LADs), TAP deficiency syndrome), disorders of hemoglobin synthesis (Sickle cell anemia, thalassemia), autoimmune diseases (rheumatoid arthritis, systemic lupus erythematosus), accelerated and advanced aging (Progeria, Werner syndrome, Down syndrome), amongst others [1,2].

In turn, modifiable—meaning preventable risk factors—are psychological stress, smoking, inappropriate alcohol consumption, imbalanced diet, sedentary life-style, suboptimal health conditions such as Flammer syndrome, disturbed microcirculation, impaired wound healing, accelerated aging, preventable metabolic syndrome(s) and cardio-vascular pathologies, among others [1,2].

Consequently, a robust predictive/prognostic approach by multi-level diagnostics should be based on the individualised patient profiling essentially including

- family history (non-modifiable factor of genetic predisposition, e.g. to accelerated aging)
- biologic vs. chronologic age
- anthropometric parameters
- tissue composition (fat/water/mussels)
- sub/cellular biomarker-panels providing information for healing quality (e.g. scar formation), post-treatment sustainability of the modification and potential complications including chronic non-healing wounds, amongst others [1–4].

Consequent preventive measures may include individualised patient consultations, dietary and life-style aspects, adaptive modifications to the treatment approach and/or overall disease management.

**References**

1. Stolzenburg-Veeser L, Golubnitschaja O. Mini-encyclopaedia of the wound healing - Opportunities for integrating multi-omic approaches into medical practice. Journal of Proteomics. 2018;188:71–84. 10.1016/j.jprot.2017.07.017
2. Avishai E, Yeghiazaryan K, Golubnitschaja O. Impaired wound healing: facts and hypotheses for multi-professional considerations in predictive, preventive and personalised medicine. EPMA J. 2017;8(1)23–33. 10.1007/s13167-017-0081-y
3. Golubnitschaja O, Stolzenburg-Veeser L, Avishai E, Costigliola V. Wound healing: proof-of-principle model for the modern hospital - patient stratification, prediction, prevention and personalisation of treatment. In: Latifi R, editior. The modern hospital: patients centered, disease based, research oriented, technology driven. Cham: Springer; 2018.
4. Golubnitschaja O, Andrews RJ. Patient-centered care: making the modern hospital truly modern. In: Latifi R, editior. The modern hospital: patients centered, disease based, research oriented, technology driven, Cham: Springer; 2018.

**Flammer syndrome phenotype and vascular status of pregnant women potentially relevant to foetal developmental particularities**

Evsevyeva M^1^, Sergeeva O^1^, Kudryavtseva V^1^, Stchetinin E^1^, Golubnitschaja O*^2^

^1^Stavropol State Medical University, Russian Federation

^2^Predictive, Preventive and Personalised (3P) Medicine, Department of Radiation Oncology, University Hospital Bonn, Friedrich-Wilhelms-University Bonn, Germany

*Corresponding author: Prof. Dr. Olga Golubnitschaja, Predictive, Preventive and Personalised (3P) Medicine, Department of Radiation Oncology, University Hospital Bonn, Rheinische Friedrich-Wilhelms-Universität Bonn, Venusberg-Campus 1, 53127 Bonn, Germany; e.mail: Olga.Golubnitschaja@ukbonn.de

**Keywords:** predictive preventive personalised medicine, Flammer syndrome, vasospastic women, reproductive age, questionnaire, primary vascular dysregulation, phenotype, pregnancy, foetal development, growth-weight parameters, new-borns hypotrophy, Quetelet I index, in-depth diagnostics, peripheral augmentation index, intensive care, modifiable risk factors, risk assessment, multi-parametric analysis

**Background**

Flammer syndrome (FS) phenotype has been abundantly demonstrated as a suboptimal health condition which, if not corrected well in time, may potentially predispose affected individuals to a cascade of secondary complications, e.g. in pregnancy [1] and even severe pathologies, due to chronic vasospastic condition and significant alterations described at molecular, cellular and systems level [2].

**Working hypothesis**

Potential impacts of FS phenotype to the altered vascular status of pregnant women being potentially relevant to foetal developmental particularities have been hypothesised.

**Material and methods**

Forty-five pregnant women (mean age 28.4 ± 2.3) were under observation. The target group comprised 21 pregnant women demonstrating clear symptoms of FS-phenotype diagnosed by utilising the FS-specific questionnaire [2]. The control group comprised 24 age-matched FS-phenotype-free pregnant women. Vascular rigidity indices in each trimester, growth-weight parameters of new-borns (Quetelet I index) and delivery term were statistically evaluated for each group.

**Results**

New-borns of mothers with FS-phenotype were characterised by significantly lower weight (3169 ± 296 vs. 3620 ± 363 g), height (50.8 ± 1.51 vs. 53 ± 1.6 cm) and gestational age (38.4 ± 0.81 vs. 39.5 ± 0.53). Trophic status of new-borns, reflecting nutritional particularities during the intrauterine period, differed markedly between the groups: a hypotrophy in new-borns was significantly more frequent (40% vs. 5.5%), whereas both the normotrophy (53.4% and 72.2%) and hypertrophy were much rarer (6.6% and 22.7%) in the group with FS-phenotype vs. control. Furthermore, the FS-group had a significantly increased vascular stiffness compared with the control. Significant differences were monitored for peripheral augmentation index AIx75 in first trimester (−45% vs. −61%, respectively).

**Conclusions and expert recommendations**

The FS-phenotype of pregnant women is demonstrated as being strongly associated with significant alterations in maternal vascular status and, amongst other, trophic parameters of the new-born; all other potential alterations are to be explored further. The recorded alterations are a clear indication to include the FS-phenotype as a criterion for predictive diagnosis and intensive care during pregnancy. In-depth follow-up studies are essential to be performed, in order to elaborate on targeted preventive measures and personalised treatment algorithms tailored to the FS-affected women in reproductive age.

**References**

1. Golubnitschaja O, Flammer J. Individualised patient profile: Clinical utility of Flammer syndrome phenotype and general lessons for predictive, preventive and personalised medicine. EPMA J 2018;9(1):15–20. 10.1007/s13167-018-0127-9.
2. Golubnitschaja O. Flammer syndrome: from phenotype to associated pathologies, prediction, prevention and personalisation. Cham: Springer; 2019.

**Individualized nutritional approach for individuals with the Flammer syndrome phenotype**

Shapira N*^1^ and Golubnitschaja O^2,3,4^

^1^Nutrition Department, Ashkelon Academic College, Ashkelon, Israel

^2^Predictive, Preventive Personalised (3P) Medicine, Department of Radiation Oncology, University Hospital Bonn, Rheinische Friedrich-Wilhelms-Universität Bonn, Germany

^3^Breast Cancer Research Centre, University Hospital Bonn, Rheinische Friedrich-Wilhelms-Universität Bonn, Germany

^4^Centre for Integrated Oncology, Cologne-Bonn, Rheinische Friedrich-Wilhelms-Universität Bonn, Germany

*Corresponding author: Prof. Dr. Niva Shapira, Nutrition Department, Ashkelon Academic College, Ashkelon, Israel; e.mail: nivnet@inter.net.il

**Keywords:** predictive preventive personalized medicine (3PM), Flammer syndrome, nutrition, individualized patient profile, symptoms, primary vascular dysregulation, mental health, metabolic particularities, impairment, eye disorders, neurodegeneration, modifiable risks, psychological aspects

**Background**

Flammer syndrome (FS) was primarily defined as an ophthalmological phenomenon, associated with central and peripheral symptoms, resulting mostly from dysregulation of systemic and local blood circulation [1]. Recently, a number of pathologies potentially linked to FS as a suboptimal health condition have been described, making the FS-related research field of great interest specifically in the context of predictive preventive personalized medicine (PPPM / 3PM) [2–6]. As nutrition is a basic factor in the body’s functions, including blood flow regulation and related physiological manifestations, a dietary approach would be assumed to contribute to amelioration of the various disorders potentially linked to FS. The nutritional approach described here addresses some of the specific symptoms and outcomes, and expresses shared nutritional effects on biochemical and physiological mechanisms.

**Cold extremities and primary vascular dysregulation in FS**

Cold hands and feet may indicate nutritional deficiencies, including of B-vitamins, iron, magnesium, and proteins that result from either dietary insufficiency or poor absorption. Folate and vitamins B6 and B12 support blood hemoglobin synthesis. Sufficient iron levels increase metabolic activity and consequent peripheral blood vessel dilatation that results in warming the whole body and extremities. Vitamin B3 supports blood vessel functionality, and supplements have been shown to intensify blood flow and warming of extremities.

**Body dehydration as a consequence of decreased feeling of thirst in FS**

FS individuals demonstrate reduced feeling of thirst. Moreover, systemic low hydration is directly linked to pathophysiologic pathways triggered in the eye; for example, in dry eye syndrome, cataracts, refractive changes, and retinal vascular disease. To this end, the hydration factor was shown to be involved in the development of both diabetic retinopathy and glaucoma [7]. Body dehydration is a risk factor for breast cancer development [8].

**Changes in odor perception are characteristic for FS**

Taste and smell disorders can range from complete loss of function (ageusia and anosmia, respectively) to partial loss (hypogeusia and hyposmia), and/or inappropriate sensation for a given stimulus (dysgeusia and dysosmia). Changes in odor perception are characteristic for both otherwise healthy FS individuals and different patient cohorts with characteristic FS phenotype conditions, such as glaucoma [1] and breast cancer [3]. Though chemosensory complaints identify both taste and smell loss, olfactory dysfunction is primarily responsible. Zinc and other micronutrients such as vitamins A, E, and the B-family, as well as iron and iodine have been associated with chemosensory function. The overall limited success of providing these nutrients in supplement form for treating shifted taste and smell perception suggests the greater potential of food sources in this respect [9].

**Arterial hypotension is characteristic for FS**

Arterial hypotension is a key manifestation of autonomic dysfunction, typically observed when cardiovascular adaptive mechanisms fail to compensate the reduction in venous return that normally occurs with sitting up and standing. Arterial hypotension is characteristic in FS individuals highly predisposed to instability in ocular blood flow, oxidative stress and normal-tension glaucoma [1]. Nutritional means aimed at hypotension prevention include adequate dietary habits such as frequent and small meals, limited alcohol consumption [10], sufficient body hydration and intake of essential vitamins and minerals, including sodium and stimulating phytonutrients. Among micronutrients, vitamin C is supportive for blood vessel functionality, vitamin D is essential for baro-reflexive mechanisms [11] and folate together with vitamins B6 and B12 prevents anemia and hyperhomocysteinaemia. Caffeine management supports stable blood pressure [12].

**Nutrition recommendations to protect FS individuals against glaucomatous damage**

Glaucoma refers to progressive optic neuropathy that can result in irreversible vision loss and blindness. Oxidative stress is suggested as an important risk factor for glaucomatous retinal ganglion cell loss [1, 6]. Here a dietary approach adapted to the antioxidative diet is highly recommended [13]. Among micronutrients, magnesium supports blood flow and endothelial function, and balances calcium influx and glutamate release, thus protecting against oxidative stress and cell death [14]. Balanced calcium and iron levels [15], vitamin B3, and flavonoids demonstrated protective effects against glaucoma and further age-related effects. Phytonutrients such as anthocyanosides from fruits and vegetables, catechins from green tea and cocoa, flavonoids from cocoa, and curcuminoids from turmeric, showed protection of retinal ganglions and support of blood flow. Reduced caffeine is advised against acute increase in blood pressure [15].

**Conclusions and expert recommendations**

A comprehensive nutritional approach, with **emphasis on specific components as reviewed above, offers a strategy for treating individuals** with the FS phenotype. A personalized approach based on individualized patient profiling [16] is essential for a predictive medical approach in treating FS individuals with the FS phenotype [17–20]. Targeted preventive measures are possible and highly recommended [21, 22]. Most of the FS symptoms are respond negatively to nutritional imbalances and/or positively to corrections and functional interventions. Recommendations related to general dietary habits include small frequent meal intake, avoiding starvation [6], as well as sufficient amounts of essential minerals—mainly calcium, magnesium, zinc, and iron—and vitamins—such as A, C, D, and the B family. Phytonutrients and long-chain omega-3 fatty acid contributions have to be considered for stratified patient cohorts [21]. Patient stratification for the 3P medical approach is recommended via individualized patient profiling and non-invasive liquid biopsy [23, 24].

**References**

1. Flammer J, Konieczka K. The discovery of the Flammer syndrome: a historical and personal perspective. EPMA J. 2017;8(2):75–97.
2. Avishai E, Yeghiazaryan K, Golubnitschaja O. Impaired wound healing: facts and hypotheses for multi-professional considerations in predictive, preventive and personalised medicine. EPMA J. 2017;8(1):23–33. 10.1007/s13167-017-0081-y
3. Bubnov R, Polivka J Jr., Zubor P, Konieczka K, Golubnitschaja O. Pre-metastatic niches” in breast cancer: are they created by or prior to the tumour onset? “Flammer Syndrome” relevance to address the question. EPMA J. 2017;8(2):141–57. 10.1007/s13167-017-0092-8
4. Polivka J Jr., Altun I, Golubnitschaja O. Pregnancy Associated Breast Cancer: The Risky Status Quo and New Concepts of Predictive Medicine EPMA J. 2018;9(1):1–13. 10.1007/s13167-018-0129-7.
5. Polivka J Jr., Polivka J, Pesta M, Rohan V, Celedova L, Mahajani S, Topolcan O, Golubnitschaja O. Risks associated with the stroke predisposition at young age: facts and hypotheses in light of individualized predictive and preventive approach. EPMA J. 2019;10(1):81–99. 10.1007/s13167-019-00162-5.
6. Golubnitschaja O. Flammer syndrome – from phenotype to associated pathologies, prediction, prevention and personalization, vol. 11. Cham: Springer; 2019. 10.1007/978-3-030-13,550-8
7. Sherwin JC, Kokavec J, Thornton SN. Hydration, fluid regulation and the eye: in health and disease. Clin Exp Ophthalmol. 2015;43(8):749–764.
8. Golubnitschaja O. Feeling cold and other underestimated symptoms in breast cancer: anecdotes or individual profiles for advanced patient stratification? EPMA J. 2017;8(1):17–22. 10.1007/s13167-017-0086-6.
9. Kershaw JC, Mattes RD. Nutrition and taste and smell dysfunction. World J Otorhinolaryngol Head Neck Surg. 2018;4(1):3–10.
10. Luciano GL, Brennan MJ, Rothberg MB. Postprandial hypotension. Am J Med. 2010;123(3):281 e281–286.
11. Annweiler C, Schott AM, Rolland Y, Beauchet O. Vitamin D deficiency is associated with orthostatic hypotension in oldest-old women. J Intern Med. 2014;276(3):285–295.
12. Guessous I, Eap CB, Bochud M. Blood pressure in relation to coffee and caffeine consumption. Curr Hypertens Rep. 2014;16(9):468.
13. Ekici F, Korkmaz S, Karaca EE, et al. The role of magnesium in the pathogenesis and treatment of glaucoma. Int Sch Res Notices. 2014;2014:Article 745,439).
14. Flammer J, Konieczka K, Flammer AJ. The primary vascular dysregulation syndrome: implications for eye diseases. EPMA J. 2013;4(1):14.
15. Bussel, II, Aref AA. Dietary factors and the risk of glaucoma: a review. Ther Adv Chronic Dis. 2014;5(4):188–194.
16. Golubnitschaja O, Flammer J. Individualised patient profile: Clinical utility of Flammer syndrome phenotype and general lessons for predictive, preventive and personalised medicine. EPMA J. 2018;9(1):15–20. 10.1007/s13167-018-0127-9.
17. Kunin A, Polivka J Jr., Moiseeva N, Golubnitschaja O. “Dry mouth” and “Flammer” syndromes - neglected risks in adolescents and new concepts by predictive, preventive and personalised a. EPMA J. 2018;9(3):307–317. 10.1007/s13167-018-0145-7
18. Goncharenko V, Bubnov R, Polivka J Jr., Zubor P, Biringer K, Bielik T, Kuhn W, Golubnitschaja O. Vaginal dryness: individualised patient profiles, risks and mitigating measures. EPMA J. 2019;10(1):73–79. 10.1007/s13167-019-00164-3.
19. Golubnitschaja O, Filep N, Yeghiazaryan K, Blom HJ, Hofmann-Apitius M, Kuhn W. Multi-omic approach decodes paradoxes of the triple-negative breast cancer: lessons for predictive, preventive and personaliseedicine. Amino Acids. 2018;50(3–4):383–395. 10.1007/s00726-017-2524-0.
20. Qian S, Golubnitschaja O, Zhan X. Chronic inflammation: key player and biomarker-set to predict and prevent cancer development and progression based on individualized patient profiles. EPMA J. 10(4):365–381. 10.1007/s13167-019-00194-x
21. Kapinova A, Kubatka P, Golubnitschaja O, Kello M, Zubor P, Solar P, Pec M. Dietary phytochemicals in breast cancer research: Anticancer effects and potential utility for effective chemoprevention. Environ Health Prevent Med. 2018;23–36. 10.1186/s12199-018-0724-1.
22. Bubnov R, Babenko L. Lazarenko L, Kryvtsova M, Shcherbakov O, Zholobak, N, Golubnitschaja O, Spivak M. Can tailored nanoceria act as a prebiotic? Report on improved lipid profile and gut microbiota in obese mice. EPMA J. 2019. 10(4):317–335. 10.1007/s13167-019-00190-1.
23. Golubnitschaja O. The keyrole of multiomics in the predictive, preventive. Klinische Monatsblätter für Augenheilkunde. 2018;235:1–5. 10.1055/s-0044-101164.
24. Gerner C, Costigliola V, Golubnitschaja O. Multiomic patterns in body fluids: technological challenge with a great potential to implement the advanced paradigm of 3P medicine. Mass Spectrom Rev. 2019. 10.1002/mas.

**Voice as a biomarker for early detection and prediction of related disorders**

Sargheini N^1^ and Golubnitschaja O*^2^

^1^Center of Molecular Biotechnology, Rheinische Friedrich-Wilhelms-Universität Bonn, Germany

^2^Predictive, Preventive and Personalised (3P) Medicine, Department of Radiation Oncology, University Hospital Bonn, Rheinische Friedrich-Wilhelms-Universität Bonn, Germany

*Corresponding author: Prof. Dr. Olga Golubnitschaja, Predictive, Preventive and Personalised (3P) Medicine, Department of Radiation Oncology, Friedrich-Wilhelms-University Bonn, Venusberg-Campus 1, 53127 Bonn, Germany; e.mail: Olga.Golubnitschaja@ukbonn.de

**Keywords:** voice, biomarker, predictive preventive personalized medicine, individualized patient profile, disease predisposition, risk factors, stress, ethics, healthcare

**Classification of voice perturbation and disorders**

Voice disorders, also called dysphonia, are characterized by abnormalities in vocal quality, pitch, loudness, and vocal effort resulting from neurological, structural or functional problems which affect the voice-related quality of life. Dysphonia occurs in 3–9% of the population [1]. Factors, including family history, gender, age, occupation, and smoking are obviously associated with the voice perturbation and disorders Table 1.

**Table 1** Risk factors of voice disorders

**Table:** 

Risk factors	Significance	Reference
Gender	• Higher prevalence of voice disorders in women (*p* < 0.01)	1
Age	• Increased laryngeal disorders within elderly (>65 years old)	1
Puberty	• Remarkable decrease in F0 and voice pitch	2,3
Family history of voice disorders	• Relationship between family history of hoarseness and voice disorders in teachers (*P* = 0.0001)	4
Occupation	• Voice disorders in 66% of teachers, 59% of call centre workers, 47% of singers • Voice perturbation in 82% of teachers for first time in professional job	4
Smoking (Tobacco)	• Higher risk of laryngeal disorder (*p* < 0.05) • Lower values of F0 and MPT (*p* < 0.05) • Increased jitter, shimmer percentage (*p* < 0.05)	5

“F0”–Fundamental frequency; “MPT”–Maximum phonation time

Dysphonia is aetiologically classified into three main categories: functional, organic, and neurological.

1. Functional dysphonia (FD)

FD involves voice abnormalities as the consequence of voice misuse in the absence of anatomical or neurological perturbations. FD includes psychogenic voice disorder (PVD), muscle tension voice disorder (MTVD), stuttering, childlike prosody, and articulation abnormalities. PVD and MTVD are the most prevalent types of FD [6]. The clinical features of FD are summarized in Table 2.

**Table 2** Clinical features of FD

**Table:** 

Disorders	Clinical features	References
Psychogenic dysphonia	• Abrupt onset of dysphonia • Hoarseness, chronic aphonia, intermittent voicing • Tremor, vocal fold adduction related to muscle tone limitation during phonation, glottic cleft • Common in women 20–40 years	7,8
Muscle tension voice disorder (MTVD)	• Slow start of dysphonia • Primary MTVD: dysphonia without laryngeal pathology • Secondary MTVD: dysphonia with laryngeal pathology like vocal cord lesion	8
Stuttering	• Stuttering on every syllable, word • Extreme variability • Presentation of accent on wrong syllable	6
Infantile prosody	Infantile speech	6
Abnormal articulation	• Lingual, facial weakness incompatible with inaccurate articulation on tasks irrelevant to speech • Wrong-way tongue deviation with hemiparesis	6

2. Organic dysphonia

Organic dysphonia, resulting from structural changes of the larynx, due to malformation, trauma, infection, and tumors, is non-related to voice usage and considered as the common drawback of dysphonia [6, 7]. In Table 3, the laryngeal manifestations of organic dysphonia are summarized.

**Table 3** Pathologies related to organic dysphonia

**Table:** 

Risk factors	Significance	References
Laryngectomy and post-laryngectomy	• Increased jitter, shimmer percentage • Reduced MPT, HNR, mean F0 • Roughness, hoarseness after radiotherapy • Lower speech intelligibility, voice quality, speech acceptability (*P* < 0.05)	14
Post-thyroidectomy	• Significant deterioration of F0 and MPT • Transient vocal folds paralysis • Voice fatigue • Voice pitch changes • Significantly increased shimmer	15
Rheinke oedema	• Significant improvement in perceptual and acoustic vocal features and MPT in women in the postoperative	7
Acute laryngitis	• Laryngeal oedema and erythema • Inadequate phonation pressureHoarseness • Aphonia, breathy, raspy voice • low pitch level	16
Chronic laryngitis	• Continuous hoarseness • Throat sensations • Urge to clear throat	7
Vocal cord polyps	• Hoarseness, breathiness, roughness • Vocal fatigue • Adequate to low F0 • Increased noise in acoustic aspects • Lower jitter values • Airway blockage • Decreased amplitude and mucosal wave	17
Recurrent respiratory papillomatosis (RRP)	• Hoarseness, stridor • Blockage of respiratory tract	7

F0–Fundamental frequency; HNR–Harmonics-to-noise ratio; MPT–Maximum phonation time

3. Neurologic dysphonia

Lesions in the central or peripheral nervous system have been associated with neurologic dysphonia usually leading to low tone, tremor, poor prosody, and impaired articulation. Changes in voice features may accompany the development of neurological disorders [6]. Evaluations of perceptual and acoustic vocal features have revealed frequent voice dysfunction in neurological disorders, such as Parkinson disease [8], Huntington’s chorea [9], spasmodic dysphonia [6], multiple sclerosis [10], amyotrophic lateral sclerosis [8], stroke [10], myasthenia gravis [11], bulimia nervosa [12], Down’s syndrome [13], vocal cord paralysis [6], and depression [8].

**Health conditions and disorders with the laryngeal manifestation**

Gastrointestinal reflux [7,8], autoimmune diseases [7] (e.g., rheumatic arthritis, Sjögren’s syndrome, systemic lupus erythematosus, amyloidosis), respiratory disorders [18] (e.g. chronic obstructive pulmonary disease, cystic fibrosis, asthma), diabetes [3], thyroid disorders [3], acromegaly [19], postmenopausal period [3], and cardio-vocal syndrome [20] — all frequently cause changes in voice quality secondary to either functional or structural laryngological alterations. Corresponding laryngeal symptoms are summarized in Table 4.

**Table 4** Laryngeal manifestations link to certain types of disorders
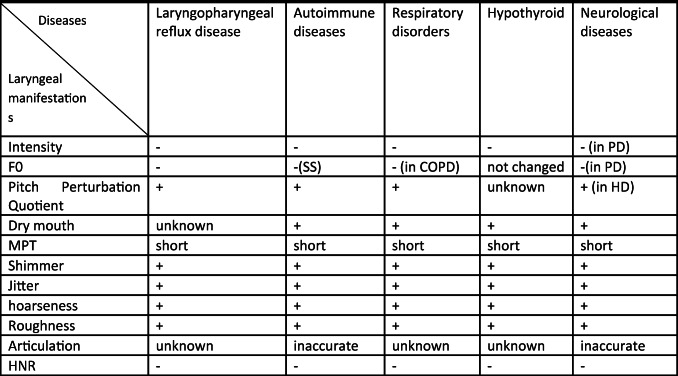


“-”–decreased; “+”–increased; “COPD”–Chronic obstructive pulmonary disease; “F0”–Fundamental frequency; “HNR”–Harmonics-to-noise ratio; “HD”–Huntington disease; “MPT”–Maximum phonation time; “PD”–Parkinson disease; “SS”–Sjögren’s syndrome

**Voice perturbations as a predictor for disease development and indicator for preventive treatments tailored to the person**

As demonstrated above, voice perturbations might be an early indicator of developing pathologies. Moreover, depending on individual parameters, the voice perturbation can be instrumental for a disease prediction and indicative of preventive measures tailored to the person. One of the best examples is an individual stress reaction causing a variety of disorders and thereby changing several biomarker patterns including voice parameters [21]. The technology of speech analysis has, therefore, great potential in predictive, preventive and personalized medicine. Further, a complementary application of specialized questionnaires, body fluids, and multi-omic analysis may significantly improve the overall predictive power of the approach providing also valuable information for targeted treatments [22, 23].

**References**

1. Lyberg-Åhlander V, Rydell R, Fredlund P, Magnusson C, Wilén S. Prevalence of voice disorders in the general population, based on the Stockholm public health cohort. J Voice. 2019;33(6):900–905.
2. Markova D, Richer L, Pangelinan M, Schwartz DH, Leonard G, Perron M, et al. Age- and sex-related variations in vocal-tract morphology and voice acoustics during adolescence. Horm Behav. 2016;81:84–96.
3. Hari Kumar KV, Garg A, Ajai Chandra NS, Singh SP, Datta R. Voice and endocrinology. Indian J Endocrinol Metab. 2016;20(5):590–594.
4. Alrahim AA, Alanazi RA, Al-Bar MH. Hoarseness among school teachers: A cross-sectional study from Dammam. J Family Community Med. 2018;25(3):205–210.
5. Ayoub MR, Larrouy-Maestri P, Morsomme D. The effect of smoking on the fundamental frequency of the speaking voice. J Voice. 2019;33(5):802.e11–802.e16.
6. Chung DS, Wettroth C, Hallett M, Maurer CW. Functional speech and voice disorders: Case series and literature review. Mov Disord Clin Pract. 2018;5(3):312–316.
7. Reiter R, Hoffmann TK, Pickhard A, Brosch S. Hoarseness—causes and treatments. Dtsch Arztebl Int. 2015;112(19):329–37.
8. Behlau M, Madazio G, Oliveira G. Functional dysphonia: Strategies to improve patient outcomes. Patient Related Outcome Measures. 2015;6:243–53.
9. Rusz J, Klempíř J, Tykalová T, Baborová E, Čmejla R, Růžička E, Roth J. Characteristics and occurrence of speech impairment in Huntington’s disease: Possible influence of antipsychotic medication. J Neural Transm. 2014;121(12):1529–39.
10. Núñez-Batalla F, Díaz-Molina JP, Costales-Marcos M, Moreno Galindo C, Suárez-Nieto C. Neurolaryngology. Acta Otorrinolaringol Esp. 2012;63(2):132–40.
11. Konstantopoulos K, Christou YP, Vogazianos P, Zamba-Papanicolaou E, Kleopa KA. A quantitative method for the assessment of dysarthrophonia in myasthenia gravis. J Neurol Sci. 2017;377:42–46.
12. Fontenelle LF, Mendlowicz MV, Moreira RO, Appolinario JC. An empirical comparison of atypical bulimia nervosa and binge eating disorder. Braz J Med Biol Res. 2005;38(11):1663–1667.
13. O’Leary L, Hughes-McCormack L, Dunn K, Cooper SA. Early death and causes of death of people with Down syndrome: A systematic review. J Appl Res Intellect Disabil. 2018;31(5):687–708.
14. Ahmadi F, Noorian F, Novakovic D, van Schaik A. A pneumatic bionic voice prosthesis—Pre-clinical trials of controlling the voice onset and offset. PLoS One. 2018;13(2):e0192257.
15. Iyomasa RM, Tagliarini JV, Rodrigues SA, Tavares ELM, Martins RHG. Laryngeal and vocal alterations after thyroidectomy. Braz J Otorhinolaryngol. 2019;85(1):3–10.
16. Jaworek AJ, Earasi K, Lyons KM, Daggumati S, Hu A, Sataloff RT. Acute infectious laryngitis:a case series. Ear Nose Throat J. 2018;97(9):306–313.
17. Vasconcelos D, Gomes AOC, Araújo CMT. Vocal fold polyps: Literature review. Int Arch Otorhinolaryngol. 2019;23(1):116–124.
18. Shastry A, Balasubramanium RK, Acharya PR. Voice analysis in individuals with chronic obstructive pulmonary disease. Int J Phonosurgery Laryngol. 2014;4(2):45–49.
19. Aydin K, Turkyilmaz D, Ozturk B, Dagdelen S, Ozgen B, Unal F, Erbas T. Voice characteristics of acromegaly. Eur Arch Otorhinolaryngol. 2013;270(4):1391–6.
20. Wu VCC, Chen CC, Hung KC, Chern MS, Wan YL, Tsai FC, et al. Reversal of hoarseness with recognition of Ortner syndrome in a patient with severe mitral regurgitation. J Cardiol Cases. 2013;7(2): e48-e50.
21. Slavich GM, Taylor S, Picard RW. Stress measurement using speech: Recent advancements, validation issues, and ethical and privacy considerations. Stress. 2019;22(4):408–413.
22. Golubnitschaja O, Stolzenburg-Veeser L, Avishai E, Costigliola V. 2018. Wound healing: proof-of-principle model for the modern hospital - patient stratification, prediction, prevention and personalisation of treatment. In: Latifi R, editior. The modern hospital: patients centered, disease based, research oriented, technology driven. Cham: Springer; 2018.
23. Patient-centered care: making the modern hospital truly modern. In: Latifi R, editior. The modern hospital: patients centered, disease based, research oriented, technology driven, Cham: Springer; 2018.

**Application of artificial intelligence to the management of chronic pathologies on example of heart failure: predictive, preventive and personalised medical approach**

Fuentes Jiménez JS^1,2^, Lage-Rupprecht V^3^, Hofmann-Apitius M^3^, Golubnitschaja O*^4^

^1^Escuela Técnica Superior de Ingeniería Agronómica, Alimentaria y de Biosistemas – Universidad Politécnica de Madrid (ETSIAAB - UPM), Spain

^2^Center of Molecular Biotechnology, Excellence Rheinische Friedrich-Wilhelms-University of Bonn, Germany

^3^Fraunhofer Institute for Algorithms and Scientific Computing SCAI - Department of Bioinformatics, Sankt Augustin, Germany

^4^Predictive, Preventive and Personalised (3P) Medicine, Department of Radiation Oncology, University Hospital Bonn, Rheinische Friedrich-Wilhelms-Universität Bonn, Germany

*Corresponding author: Prof. Dr. Olga Golubnitschaja, Predictive, Preventive and Personalised (3P) Medicine, Department of Radiation Oncology, Friedrich-Wilhelms-University Bonn, Venusberg-Campus 1, 53127 Bonn, Germany; e.mail: Olga.Golubnitschaja@ukbonn.de

**Keywords:** predictive preventive personalised medicine, artificial intelligence, chronic pathologies, heart failure, risk factors, preventable risk, collateral pathologies, healthcare economy, disease monitoring, device, multi-level diagnostics, patient stratification, biomarker panel, life quality, IT tool

**Background**

Heart failure (HF) is a life-threatening chronic and rising disease nowadays [1, 2]. HF risk factors comprise both non-modifiable (e.g. inborn HF) and modifiable ones [1, 3]. Collateral pathologies can contribute to the development and progression of HF [1, 4].

Patients diagnosed with HF need permanent monitoring. Regular visits to the cardiologist and acute hospitalisation and life-threatening risks increase the overall costs of the HF related healthcare. Contextually dedicated devices for non-stop disease monitoring (application of artificial intelligence in medicine) are considered to provide cost-effective solutions for improved HF care.

For such devices, multi-professional input by clinicians, bio-technologists, designers and computational scientists is needed to carefully stratify the patients for individualised monitoring and to adapt the device to the personalised needs of the patient. As the “proof-of-principal” healthcare model, the HF patient stratification based on characteristic clinical parameters and biomarker sets is crucial for creating an optimal device for disease monitoring and non-stop personalised patient care. This is a consideration by internationally highly recognised consortia [1].

**Materials and methods**

The project utilised the SCAIView, *a Semantic Search Engine for Biomedical Research Utilizing a Microservice Architecture*, which is the IT tool for biological and medical literature search developed by the Fraunhofer Institute for Algorithms and Scientific Computing, Sankt Agustin, Germany (SCAI). SCAIView provides an easy and fast literature review based on an automatic information extraction of the knowledge in the literature available. SCAIView offers the possibility to search in more than 90 databases and ontologies worldwide. The research was performed using two portals, SCAIVIew and PubMed; the former provided the first directions for the research, and the latter provided more detailed information for the project. With all the obtained information, a wide analysis for preventing HF was performed.

**Results interpretation and conclusions**

HF events prevention is complex and requires the analysis of many indicators. Factors such as age [5,6], BMI [7,8] or blood pressure [9,10] help influence the prognosis of HF. Biomarkers have proven a key role in the diagnosis of HF and its prevention [11, 12, 13].

Regrading the indicators analysed, patients can be classified, and HF events can even be predicted depending on the individual.

Figure 1 provides a clue to contrast clinical situations challenging the overall HF management that clearly demonstrates why a detailed patient stratification is needed to optimise predictive, preventive and personalised medical approach. Both patients suffer from HF. However, the disease origin and accompanying risks differ dramatically from each other in both clinical situations. Consequently, predictive biomarker patterns and preventive measures are highly individual for both patients.
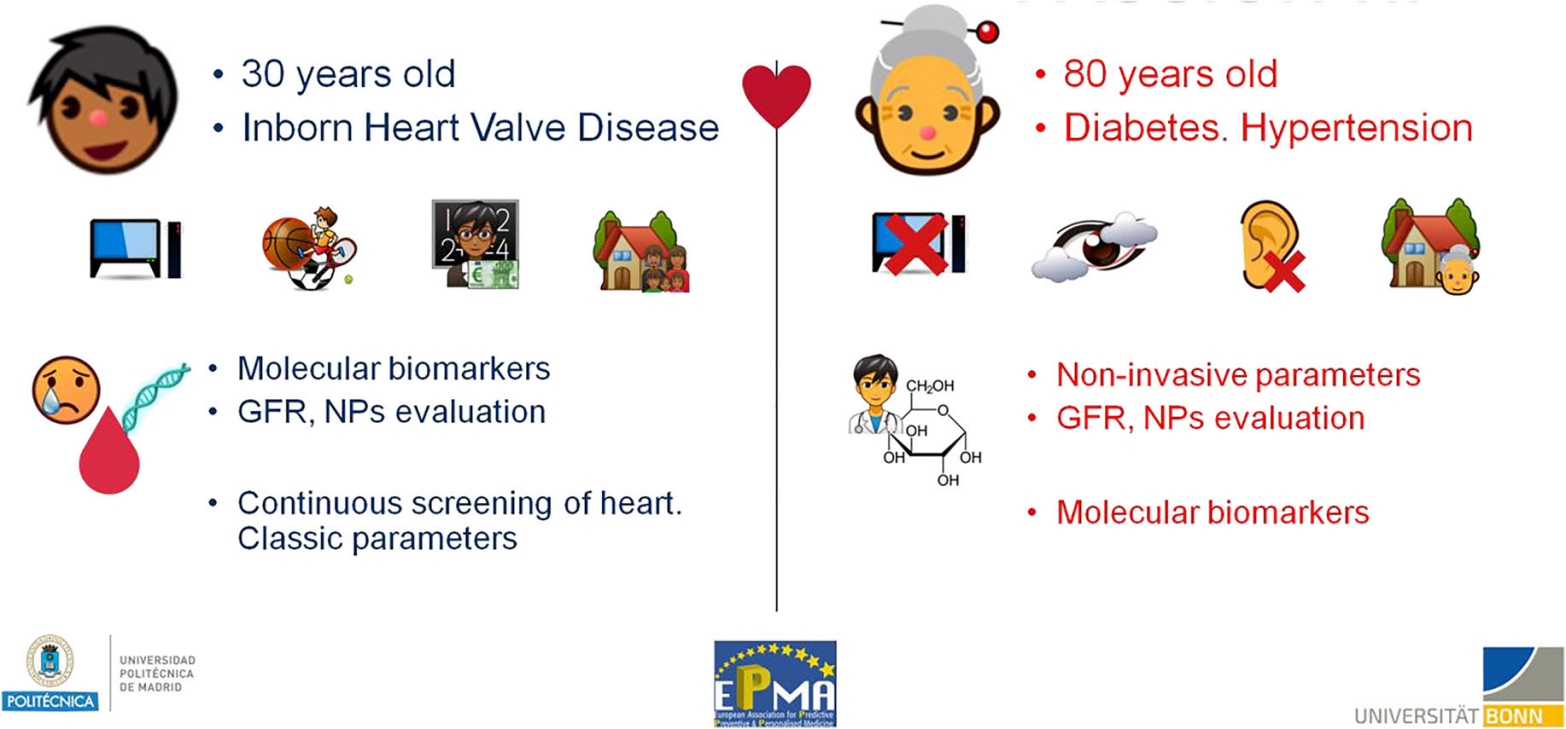


**Fig. 1** HF disease modelling and patient stratification: Is a 3P medical approach on the horizon?

**References**

1. Barrett M, Boyne J, Brandts J, Brunner-La Rocca H, De Maesschalck L, De Wit K, et al. Artificial intelligence supported patient self-care in chronic heart failure: a paradigm shift from reactive to predictive, preventive and personalised care. EPMA J. 2019;10(4):445–464.
2. Tanai E, Frantz S. Pathophysiology of heart failure. Compr Physiol. 2015;6(1):187–214.
3. Farmakis D, Parissis J, Lekakis J, Filippatos G. Acute heart failure: Epidemiology, risk factors, and prevention. Rev Esp Cardiol (Engl Ed). 2015;68(3):245–248.
4. Clinton D Kemp, John V Conte. The pathophysiology of heart failure. Cardiovasc Pathol. 2012;21(5):365–371.
5. Jacobs L, Efremov L, Ferreira JP, Thijs L, Yang W-Y, Zhang Z-Y, et al. Heart “OMics” in AGEing (HOMAGE) investigators. Risk for incident heart failure: a subject-level meta-analysis from the Heart “OMics” in AGEing (HOMAGE) Study. J Am Heart Assoc. 2017;6(5):e005231.
6. Wong CY, Chaudhry SI, Desai MM, Krumholz HM. Trends in comorbidity, disability, and polypharmacy in heart failure. Am J Med. 2011;124(2):136–143.
7. Scrutinio D, Passantino A, Guida P, Ammirati E, Oliva F, et al. Relationship among body mass index, NT-proBNP, and mortality in decompensated chronic heart failure. Heart Lung. 2017;46(3):172–177.
8. Aune D, Schlesinger S, Norat T, Riboli E. Body mass index, abdominal fatness, and the risk of sudden cardiac death: a systematic review and dose-response meta-analysis of prospective studies. Eur J Epidemiol. 2018;33(8):711–722.
9. Zhao W, Katzmarzyk PT, Horswell R, Li W, Wang Y, Johnson J, Heymsfield SB, Cefalu WT, Ryan DH, Hu G. Blood pressure and heart failure risk among diabetic patients. Int J Cardiol. 2014;176(1):125–132.
10. Segal O, Segal G, Leibowitz A, Goldenberg I, Grossman E, Klempfner R. Elevation in systolic blood pressure during heart failure hospitalization is associated with increased short and long-term mortality. Medicine (Baltimore). 2017;96(5):e5890.
11. Takaya Y, Yoshihara F, Yokoyama H, Kanzaki H, Kitakaze M, Goto Y, et al. Risk stratification of acute kidney injury using the blood urea nitrogen/creatinine ratio in patients with acute decompensated heart failure. Circ J. 2015;79(7):1520–1525.
12. K Gaggin HK, Januzzi JL Jr. Biomarkers and diagnostics in heart failure. Biochim Biophys Acta. 2013;1832(12):2442–2450.
13. Januzzi JL, van Kimmenade R, Lainchbury J, Bayes-Genis A, Ordonez-Llanos J, Santalo-Bel M, et al. NT-proBNP testing for diagnosis and short-term prognosis in acute destabilized heart failure: an international pooled analysis of 1256 patients: the International Collaborative of NT-proBNP Study. Eur Heart J. 2006;27(3):330–337.

**Consensual reaction as an indication for personalization of physiotherapeutic treatment**

Bauer J* and Podbielska H

Department of Biomedical Engineering, Faculty of Fundamental Problems of Technology, Wrocław University of Science and Technology, Wybrzeże Wyspiańskiego 27, 50-370 Wrocław, Poland

*Corresponding author: Dr. Joanna Bauer, Department of Biomedical Engineering, Wrocław University of Science and Technology, Wybrzeże Wyspiańskiego 27, 50-370 Wrocław, Poland; e.mail: joanna.bauer@pwr.edu.pl

**Keywords:** consensual reaction, physiotherapy, patient stratification, physiotherapeutic procedures management, individualized patient profiling, improved outcomes, health economy

**Introduction**

It is well known that the patient’s response to the physical agents as used in contemporary physiotherapy is person dependent;however, this phenomenon is seldom taken into account in daily practice. The proper anamnesis, allowing to collect the necessary information on patients medical history such as illnesses, underwent treatments, family diseases and lifestyle, may help to choose or to restructure the current treatment. Therefore, the individualized patient profiling may lead to proper evaluation of individual details and play a crucial role in the proper approach for the patient. For example, recently, a case study on Flammer syndrome to understand the patient’s illness and family history, in order to make recommendations based on predictive, preventive, and personalized medicine (PPPM), was reported [1]. To understand the individual details is an important issue in view of prediction of therapy results. Such an approach may result not only in improved therapy outcomes but may have a direct economic impact (earlier rehabilitation of the patient, cost effective treatment strategy etc.). Additionally, the exact information on a patient should ease the stratification [2, 3]. Proper stratification is also very important in physiotherapy. Therefore, we would like to demonstrated that response to physical stimuli is an individual feature, even for consensual reaction. The aim of the study was to assess the contralateral response in healthy volunteers exposed to various physiotherapeutic treatments such as local cryotherapy, ultrasonotherapy, electrostimulation with diadynamic currents and visible-near infrared (VIS-NIR) light irradiation. Treatments were performed on the quadriceps muscle of the right leg, whilst corresponding place on the left leg was the reference limb. Thermographic images were taken before and after the procedure at set intervals. Altogether, 712 thermograms were analyzed. The temperature differences between the treated limb and the reference limb, were observed, confirming the presence of consensual reaction after the four monotherapies were analyzed. It was observed that the consensual reaction depends on the age and gender of the patients, and it is an individual characteristic of the person, and thus can be used for patient stratification and lead to personalization of physiotherapeutic treatment.

**Material and methods**

The study was performed under the permission from the Senate Commission of Bioethics of the Wrocław University School of Physical Education, maintaining the ethical guidelines of the Declaration of Helsinki. The research group consisted of 178 healthy volunteers, including seniors (aged 58–80) and juniors (aged 19–28 years). The participants were a priori informed about the course of the experiment and voluntarily agreed to take part in it. The thermal response of the organism was examined using the FLIR T335 camera. All treatments were performed on the quadriceps muscle of the right tight. The left leg was the reference limb and was used for evaluation of the consensual reaction. Four images were captured for each volunteer—before the treatment (T0), immediately after the treatment (T1), and then 15 min (T2) and 30 min (T4) after the procedure. Altogether, 712 thermograms were recorded and analyzed using ThermaCAM Researcher 2.10 software.

**Results**

The experiment shows that the duration and strength of the contralateral reaction depends on the gender of the patient (Table 1). Both, cryotherapy and infrared radiation cause a strong contralateral response of the body, which persists for up to 30 min after treatment. Ultrasonotherapy and electrostimulation with diadynamic currents also cause a consensual reaction, but less intensive.

**Table 1** The differences of mean temperatures between women and men for experimental phases (T1, T2, and T3) in comparison to the temperature before treatment (T0) for the left limb
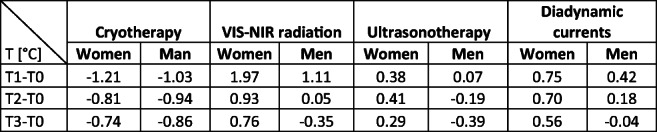


Age of the patients also seems to be a factor that affects the contralateral body’s response (Table 2). In all the analyzed cases, the consensual reaction in seniors was stronger. A particularly strong response was observed after thermotherapy involving infrared radiation.

**Table 2** The differences of mean temperatures between Juniors and Seniors for experimental phases (T1, T2 and T3) compared to the temperature before treatment (T0) for the left limb
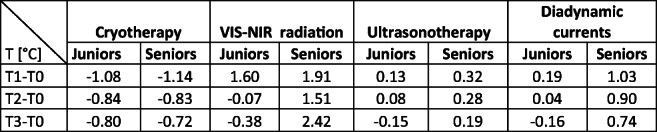


Figure 1 shows the changes in average temperature of the reference limb for the examined phases of the experiment, compared to the initial temperature. The strongest consensual reaction was observed in junior women and senior men just after the treatment. The thermal response of the body after this procedure was long-lasting. In no case, within 30 min after completing the treatment, the reference thigh temperature returned to the initial value.
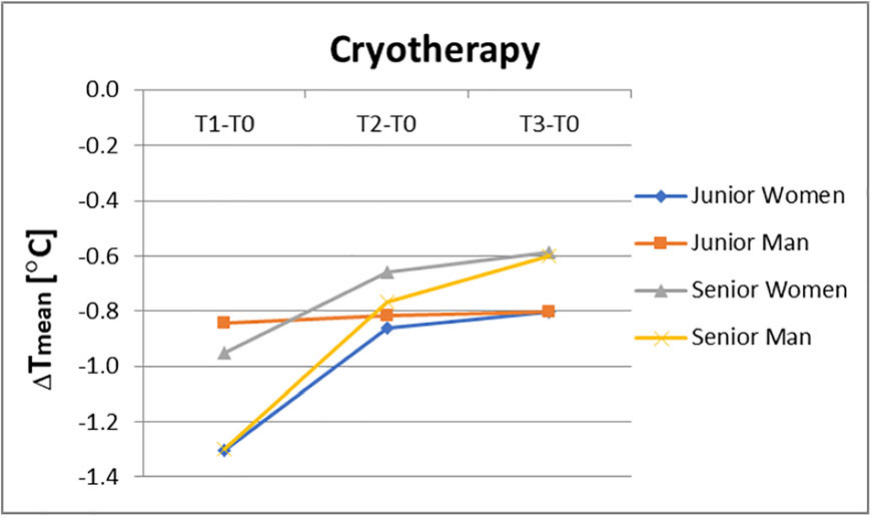


**Fig. 1** Changes of the mean temperatures of the left limb after local cryotherapy’ application

Figure 2 shows the consensual reaction observed in patients after thermotherapy with use of visible and near infrared radiation. In this case, immediately after the procedure response was stronger in women, but in men the reaction lasted longer.

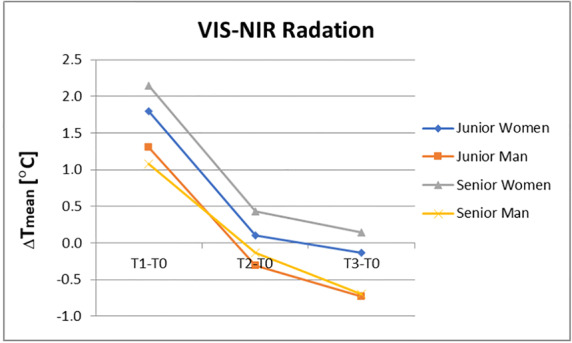



**Fig. 2** Changes of the mean temperatures of the left limb after VIS-IR radiation’ application

Figure 3 presents analogous data for ultrasonotherapy. We can see strong consensual reaction in the case of the senior women. The final figure illustrates the contralateral response observed after application of diadynamic currents (Fig. 4). Again, the reaction in women was more visible than that in men.
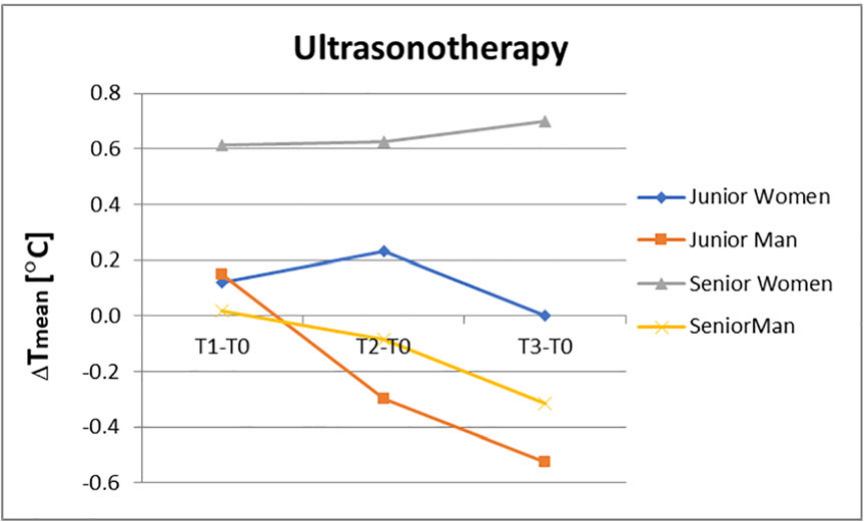


**Fig. 3** Changes of the mean temperatures of the left limb after ultrasonotherapy application
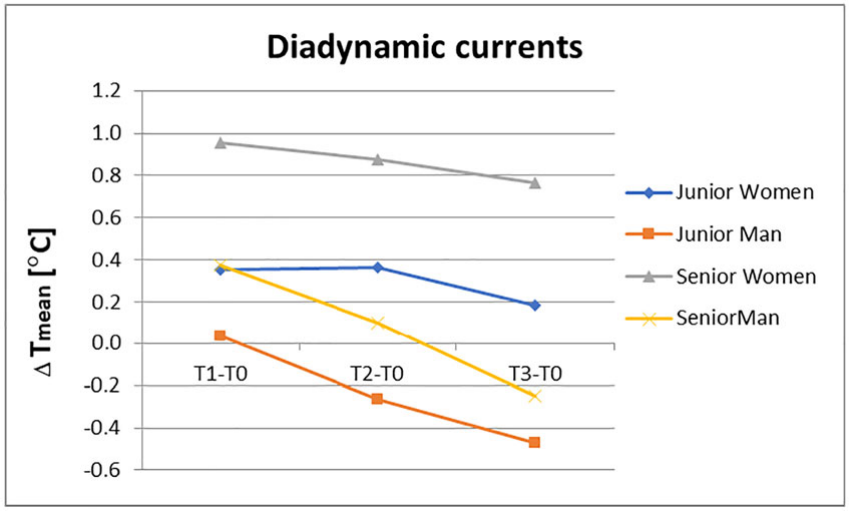


**Fig. 4** Changes of the mean temperatures of the left limb after diadynamic currents’ application

**3PM related conclusions regarding the physiotherapeutic treatment**

Modern physiotherapy is based on a comprehensive approach to patients and includes many methods that can have a synergistic effect in the treatment and prevention of diseases. Physiotherapeutic procedures, including kinesitherapy, massage and physical therapies are nowadays used mainly for combating pain and inflammations, speeding up the healing process of soft and hard tissues and improving general patients’ mobility. Parameters of physical stimuli are determined in accordance with accepted standards as well as knowledge and experience of a physiotherapist. Procedures are personalized to a small extent, mainly due to the lack of tools to assess the patient’s response to a given stimulus. Contemporary physiotherapy should consider the general recommendations of PPP medicine [4–6], but gaps remain.

Most of the physical treatments cause thermal effects, resulting in a change of the temperature in the treated region, as well as the reaction occurring on the opposite side of the body—called consensual response [7, 8]. Here, infrared thermography is a valuable tool because it is fast, non-invasive, non-destructive, and an accurate method of the evaluation of the surface temperature. A contralateral reaction occurs due to segmental innervation of the body and is defined as the response of a symmetrical body segment to a stimulus acting on the opposite area. It arises from crossed vasomotor reflexes and under certain circumstances, leads to additional benefits for patients by engaging the whole-body response. In other cases, however, it may adversely affect the results of treatment and may even be contraindicated.

Assessing the size and length of a consensual response can be one of the valuable factors used for personalization of physiotherapeutic treatment. Although the contralateral reflex is a very important factor in physiotherapeutic practice, it remains little-known and minimal attention has been paid to it in the literature attention has been paid to it in the literature. Our paper intends to address this drawback.

The application of infrared imaging for monitoring the physiotherapeutic procedures seems to have many advantages. Thermography may be a valuable tool for preventing possible tissue damage during physiotherapy procedures, e.g., as a result of incorrect selection of physical stimulus parameters. It may also help to stratify the patients based on factors such as gender, age, thermal response of the treated region as well as the contralateral one and further lead to more patient-oriented therapy. If known, the patient’ thermal profile may be used for personalization of physioterapeutic procedures and help to increase the treatment’ efficiency. Also, if needed, the thermal profile of the entire stratified group may be used, this however requires extended research, which will indicate the statistical relationships between the stimuli parameters and the group response.

**References**

1. Golubnitschaja O, Flammer J. Individualised patient profile: clinical utility of Flammer syndrome phenotype and general lessons for predictive, preventive and personalised medicine. EPMA J. 2018;9(1):15–20. 10.1007/s13167-018-0127-9.
2. Qian S, Golubnitschaja O, Zhan X. Chronic inflammation: key player and biomarker-set to predict and prevent cancer development and progression based on individualized patient profiles. EPMA J. 2019;10:365–381. 10.1007/s13167-019-00194-x
3. Kunin A, Polivka J, Moiseeva N et al. “Dry mouth” and “Flammer” syndromes—neglected risks in adolescents and new concepts by predictive, preventive and personalised approach. EPMA J. 2018;9:307–317. 10.1007/s13167-018-0145-7
4. Golubnitschaja O, Costigliola V, EPMA. General report & recommendations in predictive, preventive and personalised medicine 2012: white paper of the European Association for Predictive, Preventive and Personalised Medicine. EPMA J. 2012;3(1):14. 10.1186/1878-5085-3-14
5. Golubnitschaja O, Costigliola V. EPMA summit 2014 under the auspices of the presidency of Italy in the EU: professional statements. EPMA J. 2015;6(4):1–11 https://doi.org//10.1186/s13167-015-0026-2.
6. Golubnitschaja O, Baban B, Boniolo G, Wang W, Bubnov R, Kapalla M, Krapfenbauer K, Mozaffari MS, Costigliola V. Medicine in the early twenty-first century: paradigm and anticipation - EPMA position paper 2016. EPMA J. 2016;7(23):1–13. 10.1186/s13167-016-0072-4.
7. Boerner E, Bauer J, Kuczkowska M, Podbielska H, Ratajczak B. Comparison of the skin surface temperature on the front of thigh after application of combined red-IR radiation and diadynamic currents executed in a different sequence. J Therm Anal Calorim. 2015;120(1):921–8.
8. Boerner E, Bauer J, Ratajczak B, Dereń E, Podbielska H. Application of thermovision for analysis of superficial temperature distribution changes after physiotherapy Comparison of infrared irradiation and cryotherapy. J Therm Anal Calorim. 2015;120(1):261–7.

**Radiomics, machine learning and biobanking — integrative role in PET-CT/MR imaging and in the medicine of the future.**

Assuane Duarte A*^1^, Huellner MW^2^

^1^ IMEPAC University Center, Araguari, Minas Gerais, Brazil.

^2^ University Hospital of Zürich, Department of Nuclear Medicine / University of Zürich, Zürich, Switzerland.

*Corresponding author: Alexandre Assuane Duarte, IMEPAC University Center, Av. Minas Gerais 1889, 38444-128 Araguari, Minas Gerais, Brazil. e.mail: alexandreassuane@hotmail.com

**Keywords:** predictive preventive personalized medicine, radiomics, multi-omics, machine learning, biobanking, pet/ct, pet/mr, pet, fdg pet/ct, medical care, in-depth diagnostics, survival, therapy prognosis, risk assessment, multi-parametric analysis, biomarker patterns

**Introduction**

Medical imaging plays a central role in modern medicine. Besides assessing and monitoring the status of patients with acute illnesses, it serves as an important tool in cancer staging, restaging, and monitoring response to oncological treatment [1]. Since the clinical implementation of positron emission tomography/computed tomography (PET/CT) and—later—positron emission tomography/magnetic resonance (PET/MR), an increasing number of oncological patients undergo such exams, owing to their often decisive impact [1].

Traditionally, such medical images are assessed qualitatively by the trained human eye, in critical cases by many eyes [2]. Nowadays, there is a trend toward computer-based and artificial intelligence-aided approaches [2]. Innovative methods for the quantification of imaging findings, or for extracting and translating data within such images, might be particularly useful in nuclear medicine [2]. Multiomics is one of the most complex approaches to diseases [2]. In oncology, it takes into account the molecular profile of cancer and uses as information sources not only DNA but also RNA, proteins, metabolites as well as medical imaging (radiomics) [2].

**Biomarkers in Nuclear Medicine**

A biomarker is any characteristic item that can be measured with high reproducibility as an indicator of normal physiological or pathological processes, or responses to a given exposure or intervention, including therapeutic ones [3]. Biomarkers are also present in imaging modalities and are expected to play an increasing role in the medicine of the future [3].

In nuclear medicine, there are numerous radiotracers that highlight specific organs, tissue, and even cellular characteristics and functions [3]. One of the most important radiotracers is FDG (fluorodeoxyglucose) that serves as a surrogate marker for glucose metabolism, which is often increased in inflamed and neoplastic tissue [4]. Traditional nuclear medicine image reading relies on the qualitative assessment of abnormal high (or low) radiotracer uptake in certain body parts, or on the quantitation of such uptake using measures of single or multiple voxels [4].

**Radiomics**

Radiomics is an inherent part of the multiomics terminology, which describes the use of computer-based high-volume imaging analysis, generating mineable data that can describe tumor heterogeneity features of, e.g., texture, shape, size, edge, and wavelet [5]. Radiomic extraction need to be done systematically and follow quality protocols. There are multiple challenges for radiomics, practically mainly reproducibility and redundancy, but also costs and transferability [5]. Innovative studies involving multiomics technology have been recently published in the area of predictive, preventive and personalized medicine (3P medicine) [11,12].

**Recent advances of radiomics and machine learning in medical services**

Arshad et al. in a multicenter study with 358 subjects analyzed the validation of radiomics in FDG-PET as a risk stratification image biomarker for non-small-cell lung cancer (NSCLC) with regard to radiotherapy and/or chemotherapy response [6]. They discovered a predictive radiomics feature that was independent of other already known prognostic factors, such as tumor volume and disease stage, and was also independent of the PET/CT machines [6].

Vuong et al. tested the interchangeability of radiomic features in FDG-PET/CT and FDG-PET/MR. They found higher stability for shape, intensity and texture, and lower stability for wavelet features [7]. They concluded that the features of shape and intensity were robust among the two imaging modalities. They also concluded that instability of specific radiomic features need to receive attention when being transferred from PET/CT to PET/MRI or when are used in combination [7].

While not exploiting radiomics as per definition, Schwyzer et al. studied a machine learning algorithm (deep neural networks) that was able to detect lung cancer fully automatically on FDG-PET images even at very low dosage levels [8].

**Biobanking in medical imaging**

Biobanks play an important role in the discovery of medical biomarkers [9,10]. A biobank consists of a database of medical information derived from samples of healthy and diseased patients [9]. Imaging biobanks are also encouraged by international medical societies [9,10].

**Conclusions and PPPM recommendations**

**Predictive medicine**: Radiomics are a promising tool for predicting individual outcomes such as tumor aggressiveness, tumor recurrence, therapeutic response and tumoral gene expression [11]. Machine learning software may improve the data mining of radiomics and eventually automatize medical image analysis.

**Preventive medicine**: Image analysis through radiomics and machine learning may prevent iatrogenesis, e.g., by decreasing unnecessary and ineffective treatments, based on precognition of treatment response. Biobanks foster international medical cooperation and drug discovery, revealing changes at the molecular level (multiomics approach) in a suboptimal stage, with the ulterior motive of preventing disease or forcing early diagnosis.

**Personalized medicine**: Multiomics are a bridge to personalized medicine. Radiomics personalize medical imaging, creating a new role for image analysis in tumor staging [11]. Machine learning software may help individualize image findings by data mining [2]. Biobanks unify medical knowledge and improve individualization of treatments.

**References**

1. Huellner MW, Appenzeller P, Kuhn FP et al. Whole-body nonenhanced PET/MR versus PET/CT in the staging and restaging of cancers: preliminary observations. Radiology. 2014;273:859–869. 10.1148/radiol.14140090
2. Lu M, Zhan X. The crucial role of multiomic approach in cancer research and clinically relevant outcomes. EPMA J. 2018;9:77. 10.1007/s13167-018-0128-8
3. FDA-NIH Biomarker Working Group. BEST (Biomarkers, EndpointS, and other Tools) resource. Silver Spring (MD): Food and Drug Administration (US) 2016. Available from: https://www.ncbi.nlm.nih.gov/books/NBK326791/.
4. International Atomic Energy Agency (IAEA). Standard operating procedures for PET/CT: a practical approach for use in adult oncology. Human Health Series 2013;26. Available from: https://www.iaea.org/publications/10423/standard-operating-procedures-for-pet/ct-a-practical-approach-for-use-in-adult-oncology
5. Bogowicz M, Vuong D, Huellner MW et al. CT radiomics and PET radiomics: ready for clinical implementation? Q J Nucl Med Mol Imaging. 2019;63:355–370. 10.23736/S1824-4785.19.03192-3
6. Arshad MA, Thornton A, Lu H et al. Discovery of pre-therapy 2-deoxy-2-18 F-fluoro-D-glucose positron emission tomography-based radiomics classifiers of survival outcome in non-small-cell lung cancer patients. Eur J Nucl Med Mol Imaging. 2019;46:455–466. 10.1007/s00259-018-4139-4).
7. Vuong D, Tanadini-Lang S, Huellner, MW et al. Interchangeability of radiomic features between [18F]-FDG PET/CT and [18F]-FDG PET/MR. Med Phys. 2019;46:1677–1685. 10.1002/mp.13422.
8. Schwyzer M, Ferraro DA, Muehlematter UJ et al. Automated detection of lung cancer at ultralow dose PET/CT by deep neural networks – Initial results. Lung Cancer. 2018;126:170–173. 10.1016/j.lungcan.2018.11.001
9. European Society of Radiology (ESR) et al. ESR position paper on imaging biobanks. Insights Imaging. 2015;6:403–410. 10.1007/s13244-015-0409-x
10. Kinkorová J, Topolčan O. Biobanks in Horizon 2020: sustainability and attractive perspectives. EPMA J. 2018;9:345–353. 10.1007/s13167-018-0153-7
11. Yi X, Guan X, Zhang Y et al. Radiomics improves efficiency for differentiating subclinical pheochromocytoma from lipid-poor adenoma: a predictive, preventive and personalized medical approach in adrenal incidentalomas. EPMA J. 2018;9:421–429. 10.1007/s13167-018-0149-3
12. Fröhlich H, Patjoshi S, Yeghiazaryan K et al. Premenopausal breast cancer: potential clinical utility of a multi-omics based machine learning approach for patient stratification. EPMA J. 2018;9:175–186. 10.1007/s13167-018-0131-0

**A new Down syndrome screening algorithm, including a personalized prediction of the risk of preeclampsia and thyroid disorders in the first trimester of pregnancy**

Springer D*

First Faculty of Medicine, Charles University, Department of Medical Biochemistry and Laboratory Diagnostics; General University Hospital, Praha, Czech Republic

*Corresponding author: Drahomira Springer, Ph.D., Assoc. Professor: e.mail: drahomira.springer@lf1.cuni.cz

**Keywords:** Preventive predictive personalized medicine (PPPM), Screening, Down syndrome, Preeclampsia, Thyroid disorders, Preventative procedure, Pregnancy complications, Risk assessment, Personalized prediction, Screening algorithm.

**Introduction**

Advances in modern medicine have given us the opportunity to identify many diseases before they occur and in a timely enough measure to apply preventative procedures so that morbidity and mortality may be avoided. As maternal and perinatal mortality has decreased in many countries, the focus of perinatal medicine continues to be expanded to include further examination of maternal and fetal health indicators. The laboratory’s role in risk management differs strategically according to the phase of pregnancy [1].

As early as the first visit to the gynecologist, when the physician combines data about the mother with the findings of biophysical and biochemical tests, specific risks that can lead to a variety of pregnancy complications can be identified. Those include: miscarriage and fetal death, preterm delivery, preeclampsia, gestational diabetes, fetal growth restriction, etc.

**Screening for chromosomal aberrations**

Down’s syndrome (DS) is the most common chromosomal abnormality in humans. The risk of the birth of a child with DS increases with the mother’s age. Currently, screening for Down’s syndrome is offered to all pregnant women and the screening test varies according to the week of pregnancy. DS screening strategies involve the more traditional second trimester serum biochemistry tests, the first trimester tests that combine ultrasound markers and serum biomarkers, and the most effective system is the integration of both tests. Screening methods include detection of cell-free fetal DNA from a maternal serum as well.

Between 10 and 12 weeks of pregnancy, a combined test is offered. The test combines the results of the determination of pregnancy specific protein A (PAPP-A) and free beta-hCG in maternal serum with measurement of fetal nuchal translucency using ultrasonography (USG). Additionally, the USG examination indicates the presence of the nasal bone and flow in the ductus venosus, hepatic artery, and across the tricuspid valve bringing other screening improvements [2].

In the second trimester, the risk of having a child with DS is calculated from the levels of alpha-1-fetoprotein (AFP), human chorionic gonadotrophin (hCG), unconjugated oestriol (uE3), and inhibin A. This test is considered obsolete on its own and is only intended for women who come to the gynecologist after the 15th week of pregnancy.

The integrated test commonly evaluates the results from testing in both trimesters. Its efficacy is the best (more than 90%); it is also the safest and most cost efficient. Where ultrasonography is not available, a serum integrated test is recommended [3]. The risk is only calculated from the biochemical markers (free beta-hCG, PAPP-A, AFP, uE3, and inhibin A) and the test is as efficacious as the combined first-trimester test [2].

New developments in the area of screening include the possibility of testing for Down’s syndrome by extraction of cell-free nucleic acids from a maternal serum sample. The non-invasive prenatal testing (NIPT) has a detection rate approximately 96% for trisomy 21, with a false positive rate of 0.04% in a general obstetric population. This rate compares favorably to the combined screening detection rate of 85–90% and false positive rate of 3–5% [2]. There are different models of implementation NIPT in the screening, one of them is on Schema 1.
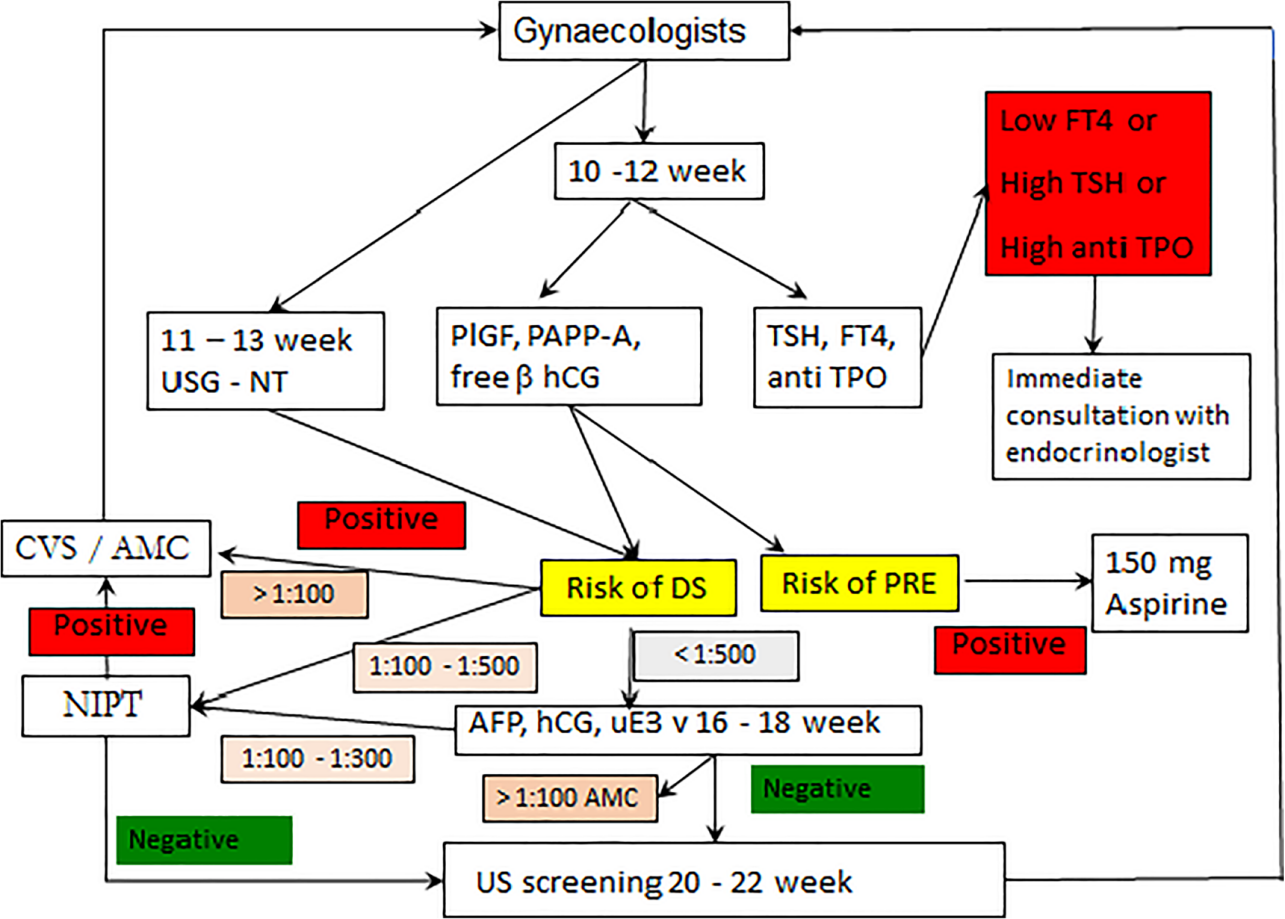


**Schema 1** Screening algorithm with the involvement of risk assessment of early pre-eclampsia and thyroid disorders in chromosomal defects screening

NIPT is a profoundly important development in prenatal care that is substantially advancing the individual patient and public health benefits achieved through conventional prenatal screening. Even the best combination of USG findings, biochemistry variables, and NIPT are only predictive screening methods. Invasive methods (amniocentesis or chorionic villus sampling) are used for confirmation of diagnosis by examination of the chromosomes of the fetus.

**Preeclampsia**

Preeclampsia (PE), which affects about 2% of pregnancies, is a major cause of maternal and perinatal morbidity and mortality. Preeclampsia is a complication of pregnancy in which affected women develop hypertension and proteinuria. However, only a minority of pregnant women with high blood pressure are at risk of preeclampsia. Further examinations are used to detect these women. It is a combination of maternal characteristics and history with uterine artery pulsatility index (PI), mean arterial pressure (MAP), serum pregnancy-associated plasma protein-A (PAPP-A), and placental growth factor (PLGF) at 11–13 weeks’ gestation [4]. The prevalence of PE can potentially be halved by a strategy of early predictive identification of the high-risk group and the prophylactic use of aspirin (150 mg at night) [5,6].

**Thyroid diseases in pregnancy**

Thyroid diseases are quite frequent among young women. Undiagnosed thyroid diseases can have serious consequences during a person’s life. Pregnancy creates a burden on the thyroid, which can result principally in hypothyroidism and/or postpartum thyroiditis in women having antibodies against thyroid peroxidase (TPOAb) positivity. Thyroid hormones are crucial for the growth and maturation of many target tissues, especially the brain and skeleton. In case of maternal hypothyroidism, substitution with levothyroxine must be started in early pregnancy. After the 14th gestational week, the fetal brain development may already be irreversibly affected by lack of thyroid hormones [7,8].

The prevalence of manifest hypothyroidism in pregnancy reaches about 0.3–0.5%. The prevalence of subclinical hypothyroidism varies between 4 and 17%, strongly depending on the definition of the upper TSH cut-off limit. Hyperthyroidism occurs in 0.1–1% of all pregnancies. Positivity for TPOAb has been reported in between 5.1% and 12.4% of cases [9].

The main laboratory parameters for thyroid diagnosis are thyroid-stimulating hormone (TSH), free thyroxine (FT4), and TPOAb. TSH in pregnancy is physiologically lower due to the high level of hCG.

Thyroid disorders in pregnancy are associated with serious maternal, fetal, and newborn complications. The universal screening for hypothyroidism in pregnancy is recommended but it is necessary to take into consideration the necessity to determine the reference interval for the investigated region and the analytical method used [10]. If this specific reference interval is not available, the recommended interval for TSH in the first trimester should be 0.1–2.5 mIU/L [7]. It is the aim of preventive medical care to diagnose thyroid function failure as soon as possible, when the disease is at its most easily curable stage.

For some diseases of the mother (thyroid disorders, gestational diabetes, preeclampsia) or fetus (chromosomal deviations, growth restriction), a single blood collection is enough for a screening examination.

**Conclusions and PPPM-based expert recommendations**

- Screening programs are an important part of modern medicine and fit the PPPM principles.
- Screening for chromosomal aberrations combines USG and biochemical markers. A suitably selected algorithm will incorporate NIPT into screening with increasing detection rate without unnecessarily increasing costs.
- Screening for preeclampsia allows early detection and prevention of serious fetal developmental complications that may also endanger the mother’s life.
- Screening for hypothyroidism allows an early diagnosis of thyroid function failure, when the disease is still in a curable stage.

**References**

1. Golubnitschaja O, Baban B, Boniolo G, Wang W, Bubnov R, Kapalla M, et al. Medicine in the early twenty-first century: paradigm and anticipation - EPMA position paper 2016. EPMA J. 2016; 10.1186/s13167-016-0072-4.
2. The UK NSC recommendation on Down’s syndrome screening in pregnancy. January 2016; https://legacyscreening.phe.org.uk/downs
3. Springer D, Loucky J, Tesner P, Cutka D, Stejskal D, Gregot V, et al. Importance of the integrated test in the Down’s syndrome screening algorithm. J Med Screen. 2018; 10.1177/0969141317752533.
4. Phipps E, Prasanna D, Brima W, Jim B. Preeclampsia: updates in pathogenesis, definitions, and guidelines. Clin J Am Soc Nephrol. 2016; 10.2215/cjn.12081115.
5. Akolekar R, Syngelaki A, Poon L, Wright D, Nicolaides KH. Competing risks model in early screening for preeclampsia by biophysical and biochemical markers. Fetal Diagn Ther. 2013; 10.1159/000341264.
6. Park F, Russo K, Williams P, Pelosi M, Puddephatt R, Walter M, et al. Prediction and prevention of early-onset pre-eclampsia:impact of aspirin after first-trimester screening. Ultrasound Obstet Gynecol. 2015; 10.1002/uog.14819.
7. De Groot L, Abalovich M, Alexander EK, Amino N, Barbour L, Cobin RH, et al. Management of thyroid dysfunction during pregnancy and postpartum: an Endocrine Society clinical practice guideline. J Clin Endocrinol Metab. 2012; https://www.ncbi.nlm.nih.gov/pubmed/22869843
8. Medici M, Korevaar TI, Visser WE, Visser TJ, Peeters RP. Thyroid function in pregnancy: what is normal? Clin Chem. 2015; 10.1373/clinchem.2014.236646.
9. Springer D, Jiskra J, Limanova Z, Zima T, Potlukova E. Thyroid in pregnancy: From physiology to screening. Crit Rev Clin Lab Sci. 2017; 10.1080/10408363.2016.1269309.
10. Springer D, Bartos V, Limanova Z, Zima T. Reference intervals for thyroid markers in early pregnancy determined by 7 different analytical systems. Scand J Clin Lab Invest. 2014; 10.3109/00365513.2013.860617.

**The Text neck syndrome effect on microcirculation of periodontal tissues blood vessels: application of 3PM concepts in dentistry**

Orekhova L^1,2^, Iamanidze N*^1,2^, Loboda E^1,2^, Pachkoria M^1^, Tachalov V^1^

^1^Department of therapeutic dentistry and periodontology of Federal State Budgetary Educational Institution of Higher Education «Academician I.P. Pavlov First St. Petersburg State Medical University» of the Ministry of Healthcare of Russian Federation.

^2^City Periodontal Center «PAKS» Ltd., Saint-Petersburg, Russian Federation.

*Corresponding author: Nino Iamanidze, assistant of Therapeutic Dentistry and Periodontology Department, Pavlov First Saint Petersburg State Medical University, 6/8 Lva Tolstogo Street, 197022, St. Petersburg, Russia; e.mail: ninihd@mail.ru

**Keywords:** Text neck syndrome, incorrect posture, periodontal diseases, Minimax-Doppler-K, individual oral hygiene, relevance of treatment, predictive preventive personalized medicine, personalized hygiene, 3PM, individualized patient profile, improved outcome, preventive predictive periodontology, preventive dentistry, dental disease prevention, personalized oral care, pshylogical approach in periodontology.

**Introduction**

3PM concepts. The high prevalence and increasing intensity of periodontal disease make urgent the problem of active prevention of periodontal disease. In this aspect, clinical periodontology needs early diagnosis, prevention and early treatment of the corresponding disease [1].

Periodontal diseases are becoming more common among young patients [2,3].

Bad posture caused by excessive use of computers and electronic gadgets can affect dynamics of blood flow greatly. A prolonged static posture leads to constriction of vessels of neck, in particular, external carotid artery, as a result of which centralization of blood circulation occurs, blood is redistributed and its main volume flows to the brain. Thus, periodontal tissues cease to receive sufficient blood supply [4,9].

The “Text neck” syndrome has taken on an epidemic character and is most acute for schoolchildren, students, and IT-specialists [5].

The purpose of the study was to reveal the existence of the causal connection between incorrect posture when using computers and mobile devices and circulatory disorders in periodontal tissues.

**Methods** The study involved 60 persons aged 19 to 25 years (female – 73.2%, male – 26.8%) and used the following methods—questionnaire, survey, standard dental examination (determination DMF, PMA, BOP, OHI-s indexes)), and functional examination using the ultrasonic dopplerography “Minimax-Doppler-K” [6,7]. The participants in the study were people whose activities do not involve daily work on computers and mobile devices and people associated with information technology.

The research objectives also included the development of a leaflet-memo for the prevention of the “Text neck” syndrome.

**Results** According to the questionnaire, subjects use smartphones more often (95%). At the same time, 41.5% of the subjects spend from 2 to 4 h daily with a mobile device in their hands (group No. 1), 39% – more than 4 h a day (group No. 2), 19.5% – less than 2 h a day (group No. 3).

Moreover, questionnaires showed that only 21.9% of respondents know about the syndrome.

The results of the survey in both groups turned out to be different. According to the survey, characteristics of the representatives of each group of subjects were compiled.

In a functional study, initial level of the average linear velocity of blood flow in group A was 0.57 cm/s and 0.47 cm/s in group B.

After a 30-min experiment, indicators decreased to 0.35 cm/s (group A) and 0.32 cm/s (group B).

After 1 h, hemodynamic parameters in both groups were almost equal and amounted to 0.30 cm/s (group A) and 0.28 cm/s (group B).

After 2 h it was 0.27 cm/s (group A) and 0.22 cm/s (group B).

**3PM related conclusion and outlook**

During the study, it was established experimentally that long head tilting and poor posture when working with computers and electronic gadgets have a negative effect on microcirculation in periodontal tissues.

Early diagnostic of the initial manifestations of periodontal diseases is very important to prevent the development of more severe forms of the disease, which was previously lacking [8]. The overall approach provided here will allow more accurate predictions, which will enable better patient stratification and early detection of predispositions followed by preventive measures. Text neck syndrome disorders should be included in the list of globally screened diseases because it can change the local periodontal microenvironment [10].

**References**

1. Garaicoa-Pazmino C, Decker AM, Polverini PJ. Personalized medicine approaches to the prevention, diagnosis, and treatment of chronic periodontitis. In: Polverini P. (eds) Personalized oral health care. Cham: Springer; 2015. 10.1007/978-3-319-23297-3_8
2. Glick M, Williams DM, Kleinman DV, Vujicic M, Watt RG, Weyant RJ. A new definition for oral health developed by the FDI world dental federation opens the door to a universal definition of oral health. J Public Health Dent. 2017;77(1):3–5.
3. Yao K, Yao Y, Shen X et al. Assessment of the oral health behavior, knowledge and status among dental and medical undergraduate students: a cross-sectional study. BMC Oral Health. 2019:19:26. 10.1186/s12903-019-0716-6
4. Damasceno GM, Ferreira AS, Nogueira LAC et al. Text neck and neck pain in 18–21-year-old young adults. Eur Spine J. 2018;27:1249–1254. 10.1007/s00586-017-5444-5
5. Genez Tarrifa SZ, De la Hoz LR. (2019) Text Neck, More Technology, Less Health?. In: Bagnara S, Tartaglia R, Albolino S, Alexander T, Fujita Y (eds) Proceedings of the 20th Congress of the International Ergonomics Association (IEA 2018). IEA 2018. Advances in intelligent systems and computing, vol 826. Cham: Springer; 2019. 10.1007/978-3-319-96065-4_79
6. Orekhova LY, Barmasheva AA. Doppler flowmetry as a tool of predictive, preventive and personalised dentistry. EPMA Journal 2013 Aug 28;4(1):21. 10.1186/1878-5085-4-21
7. Tachalov VV, Orekhova, LYu, Isaeva ER et al. Characteristics of dental patients determining their compliance level in dentistry: relevance for predictive, preventive, and personalized medicine. EPMA J. 2018;9, 379–385. 10.1007/s13167-018-0152-8
8. EPMA World Congress: Traditional Forum in Predictive, Preventive and Personalised Medicine for Multi-Professional Consideration and Consolidation. EPMA J. 2017;8, 1–54. 10.1007/s13167-017-0108-4
9. Barbero M, Schneebeli A, Koetsier E, Maino P. Myofascial pain syndrome and trigger points: evaluation and treatment in patients with musculoskeletal pain. Curr Opin Support Palliat Care. 2019 Sep;13(3):270–276. 10.1097/SPC.0000000000000445.
10. Decker A, Askar H, Tattan M et al. The assessment of stress, depression, and inflammation as a collective risk factor for periodontal diseases: a systematic review. Clin Oral Invest. 2020;24: 1–12. 10.1007/s00784-019-03089-3

**Identification of early signs of exacerbations in terms of prediction and prevention by assessing the quality of life in children with bronchial asthma**

Mokina N*^1^, Safonicheva O^2^, Pyatin V^1^, Mokin E^1^

^1^Departament of medical rehabilitation, Samara State Medical University, Russian Federation

^2^First Moscow State Medical University of I.M. Sechenov, Russian Federation

*Corresponding author: Prof. Dr. Natalia Mokina, Departament of medical rehabilitation and balneology, Samara State Medical University (SamSMU), Chapaevskaya str. 89, 443079 Samara, Russian Federation; e.mail: mokina1@mail.ru

**Keywords**: children, adolescents, bronchial asthma, sanatorium, diagnostics, lung function, prevention, prediction, healthcare quality, predictive medicine, personalised medicine

**Introduction** The most common problem in managing of patients with bronchial asthma (BA), especially children, is to predict and the means to prevent exacerbations in their early stage in order to maintain sufficient control over the disease and prevent further progression and deterioration. BA is certainly a common disease in pediatric group of population, with significant disruption of quality of life (QoL) [1,2,7]. Children with BA show the decrease in all of their quality of life areas, specifically physical and emotional, which primarily affects their personal school study profile, social activity and psycho-emotional development [3–6]. Such a preventive approach is especially relevant in the aspect of the concept of PPP-medicine that allows personalising the prediction of the disease progression and maintaining the optimal health status at the level of both secondary and tertiary prevention in chronic diseases, which is especially important in children and young people [8–10].

**Aim** To identify the early signs of exacerbations, in terms of prevention and prediction of further progression and deterioration of disease, in children with bronchial asthma.

**Materials and methods** In this study, which was conducted in the Samara Regional Children’s Sanatorium, 62 children with moderate BA, aged 5 to 11 years (9.6 ± 1.7 years old): 38 boys (9.7 ± 1, 7 years old) and 24 girls (9.6 ± 1.8 years old), participated. The patients received the basic therapy by GINA as step 2/3. The children were observed during their treatment in the pulmonological sanatorium, highlighting the areas of the child’s personality functioning, most affected by BA/clinical and functional signs of BA control, BA-test questionnaire and BA-quality of life (QOL) questionnaire (assessment of symptoms, physical and emotional sphere) were analysed. Statistical analysis was carried out with SPSS Statistics 19.0.

**Results** Significant differences were found in the integral QOL indicator by the end of the sanatorium treatment course: integral QOL indicator at admission to the sanatorium - 123.4 ± 20.5, integral QOL indicator before discharge from the sanatorium - 97.2 ± 9.7* (*p* < 0.05). Significant differences were established in the following parameters: the restrictions on the vital activity before discharge over the last 7 days; the restrictions of vital activity 3 months after discharge in total: restrictions of vital activity in general - 31.67 ± 3.343, emotional sphere in general - 6.170 ± 0.924 (*p* < 0.05). When analysing the average values of the integral QOL indicator at admission and before discharge from the sanatorium, depending on the sex, significant dynamics differences in QOL were found at: integral QOL indicator in boys with BA - 97.220 ± 9.668, restrictions of vital activity in boys with BA - 6.300 ± 0.949, BA symptoms in boys with BA before discharge - 6.140 ± 0.770 (*p* < 0.05).

When comparing the individual answers scoring of the QOL for boys, significant differences were found in the following parameters. Restrictions on vital activity before discharge - question 1 (How often BA bothers the child during his/her physical exertion (such as running, swimming, other sports activities, walking uphill/stairs and riding a bike) over the last 7 days?), Symptoms of asthma before discharge - question 20 (How often because of BA the child sleep badly at night over the last 7 days?). In girls, before discharge from the sanatorium, the integral QOL indicator showed significant differences: integral QOL indicator in girls - 132.330 ± 11.622, restrictions of vital activity in girls - 33.86 ± 0.378, emotional sphere in girls - 6.33 ± 1.211 (*p* < 0.05).

The discriminant analysis with the standardized canonical coefficient of the discriminant function (SCCDF) was performed. The most informative indicators were: Integral QOL indicator at sanatorium admission; Integral QOL indicator before discharge from the sanatorium; Integral QOL indicator at sanatorium admission -1.292; Integral QOL indicator before discharge from the sanatorium - 5.813. The AUROC-curve analysis showed the reliable sensitivity/specificity and information content of such indicators as: The emotional sphere summary parameter upon admission and before discharge in sanatorium in general. The integral QOL indicator before discharge was highly sensitive/specific. Integral QOL indicator at admission - 0.595; Integral QOL indicator before discharge - 1.000; The emotional sphere at admission in general was as 0.627. The emotional sphere before discharge in general was as 0.614, The integral QOL indicator 3 months after discharge indicated as 0.515 (AUROC≥0.5).

**Conclusion** Thus, identification of early signs of exacerbations in terms of prevention and prediction by assessing the quality of life in children with bronchial asthma, in the children’s pulmonary sanatorium, highlighting areas of the child’s personality functioning, most susceptible to BA, against the background of sanatorium treatment, revealed significant differences in the integral QOL parameter in children with BA: restrictions of vital activity in terms of physical activity and emotional sphere with the final dynamics of the integral QoL indicator. At the same time, discriminant analysis confirmed the information content, and AUROC analysis showed significant sensitivity/specificity of QOL integral indicator parameters in monitoring QOL in children with bronchial asthma at the sanatorium stage which allow to predict and prevent exacerbation and deterioration at all stages of medical care from outpatient to sanatorium care with the generation of a personalised long-term management and preventive plan.

**References**

1. Mokina N, Mazur L, Gudkova M. Application of methods for assessing the quality of life during a comparative analysis of the pharmacotherapy effectiveness in bronchial asthma in the children’s sanatorium. Collection of works of the 27th National Congress on respiratory diseases 2017:42.
2. Chuchalin A, Belevsky A, Smolenov I. Quality of life of children with bronchial asthma in Russia: results of a multicentre population study. Allergology. 2003;3:1–7.
3. Forrest CB, Zorc JJ, Moon J. et al. Evaluation of the PROMIS paediatric global health scale (PGH-7) in children with asthma. J Asthma. 2019;56(5):534–542. 10.1080/02770903.2018.1471701. Epub 2018 Jun 5.
4. Doğru H, Sürer-Adanır A, Özatalay E. Psychopathology, health-related quality-of-life and parental attitudes in paediatric asthma. J Asthma. 2019;56(11):1204–1211. 10.1080/02770903.2018.1531995. Epub 2018 Oct 18.
5. Kouzegaran S, Samimi P, Ahanchian H. et al. Quality of Life in Children with Asthma versus Healthy Children. Open Access Maced J Med Sci. 2018;6(8):1413–1418. 10.3889/oamjms.2018.287. eCollection 2018 Aug 20.
6. Mosenzadeh A, Ahmadipour S, Mardani M. et al. The effect of self-care education on the quality of life in children with allergic asthma. Compr Child Adolesc Nurs. 2019;42(4):304–312. 10.1080/24694193.2018.1513098. Epub 2018 Sep 5.
7. Sheng N, Ma J, Ding W, et al. Effects of caregiver-involved interventions on the quality of life of children and adolescents with chronic conditions and their caregivers: a systematic review and meta-analysis. Qual Life. Qual Life Res. 2019;28(1):13–33. 10.1007/s11136-018-1976-3. Epub 2018 Aug 30.
8. Seifirad S, Haghpanah V. Inappropriate modeling of chronic and complex disorders: How to reconsider the approach in the context of predictive, preventive and personalized medicine, and translational medicine. EPMA J. 2019;10(3):195–209. 10.1007/s13167-019-00176-z. eCollection 2019 Sep.
9. Polivka J, Pesta M, Rohan V et al. Risks associated with the stroke predisposition at young age: facts and hypotheses in light of individualized predictive and preventive approach. EPMA J. 2019;10(1):81–99. 10.1007/s13167-019-00162-5. eCollection 2019 Mar.
10. Balicza P, Terebessy A, Grosz Z et al. Implementation of personalized medicine in Central-Eastern Europe: pitfalls and potentials based on citizen’s attitude. EPMA J. 2018;9(1):103–112. 10.1007/s13167-017-0125-3. eCollection 2018 Mar.

**Vitamin D Utility: A Short Overview in the 3PM Context**

Simanek V^1^, Topolcan O^1^, Broz P^1,2^, Slouka D^1^, Kucera R*^1^

^1^Department of Immunochemistry Diagnostics, ^2^Institute of Clinical Biochemistry and Haematology, University Hospital in Pilsen, Pilsen, Czech Republic

*Corresponding author: Radek Kucera, Ph.D., Associate Professor, Department of Immunochemistry Diagnostics, University Hospital in Pilsen, Czech Republic; e.mail: kucerar@fnplzen.cz, ORCID: 0000-0002-2739-2302

**Keywords:** preventive predictive and personalized medicine (PPPM), vitamin D, calcidiol, targeted prevention, deficiency in childhood, civilization diseases, life style, reference ranges, predictive factor, personalized supplementation.

**Introduction**

Vitamin D is produced by the effect of sun rays on the skin which is where vitamin D_3_ (cholecalciferol) is formed. Vitamin D is metabolized in the liver to create the “stock form” of vitamin D (calcidiol). Its blood levels are commonly measured to assess the overall status of vitamin D in the human body [1]. Types of vitamin D are shown in Table 1.

**Table 1** Types of vitamin D

**Table:** 

Vitamin D type	Trivial name	Description
Vitamin D_2_	Ergocalciferol	Present in plants and taken in as part of the diet.
Vitamin D_3_	Cholecalciferol	Animal origin and taken in via diet or produced in the skin.
25-hydroxyvitamin D	Calcidiol	Produced by metabolism of vitamin D_2_ or D_3_ in the liver. Measured in blood.
1,25-dihydroxyvitamin D	Calcitriol	Active metabolite acts as a steroid hormone.Produced by metabolism of calcidiol in the kidneys and in other tissues.

The synthesis of vitamin D in the skin only occurs if the angle of impact is 45° or higher. In central Europe, vitamin D “from the sun” is only obtainable from mid-April to the first week of September [2]. The role of vitamin D in the prevention of rickets in children, osteomalacia in adults and osteoporosis in the elderly has been understood as important for many years now. A significant effect on the cognitive functions has been recorded. Furthermore, in elderly people with a vitamin D deficiency, a higher number of fractures is attributed to their reduced coordination of movement and sense of balance [3]. Vitamin D enhances muscle protein synthesis, affects calcium and phosphate transport through cell membranes, plays a role in muscle fiber growth and differentiation, and has a positive effect on contractile fibers and thus muscle strength. It is believed to boost faster muscle regeneration after intensive training, and there is continued speculation on its potential for athletes’ performance capacity [4]. Vitamin D stimulates both innate and acquired immunity throughout life. An association has been shown between winter and spring viral and bacterial infection, and weaknesses in the immune system caused by vitamin D deficiency [5].

**Prevention of Vitamin D Deficiency**

The general awareness of the benefits of sufficient levels of vitamin D in the human body remains unsatisfactory. Systematic and targeted prevention is the first and foremost powerful PPPM tool which should be employed to raise awareness among the professional and general public [6]. Proper stratification and spreading of potentially preventive information are necessary in order to prevent vitamin D deficiency and reduce the risks associated with inadequate vitamin D levels [7].

**Insufficient Serum Levels of Vitamin D as a Predictive Factor for Supplementation**

The link between low levels of vitamin D and many civilization diseases has been recognized: e.g., autoimmune diseases, multiple sclerosis, diabetes, infertility, osteoporosis and different types of cancer [8]. Confirmed low vitamin D levels should always be considered a predictive factor for vitamin D supplementation with respect to the conditions presented by each individual (age, gender, genetics, comorbidities, etc.), all of which must be carefully considered and taken into account as part of a personalized approach [9,10].

**Personalization of Supplementation**

According to the available statistics, 70% of the world population is vitamin D deficient. This includes people living at higher latitudes but, surprisingly, also populations living at latitudes where sunshine is plentiful year round [11]. Vitamin D supplementation is a logical preventive step which could be used to rectify this current unfavorable status. However, only personalized doses can lead to effective and safe supplementation [12]. Each supplementation should be initiated as a result of the measurement of calcidiol, the main stock form of vitamin D. Recommended doses of supplemental vitamin D are shown in Table 2 in correlation with the serum levels of calcidiol measured.

**Table 2** Calcidiol levels and the corresponding supplementation doses

**Table:** 

25-hydroxyvitamin D (calcidiol) level (ng/mL)	Supplement dose (IU/day)
> 10	10 000
10–20	10 000
20–30	8 000
30–40	5 000
40–50	2 000

Vitamin D_3_ (cholecalciferol) is the preferred form for supplementation. The daily requirement of vitamin D_3_ is reported to be 15–20 μg/day (600–800 IU/day). This value is now only considered to be satisfactory in subjects with regular exposure to sunlight. In cases where lower levels of are suspected (e.g., if exposure to sunlight is limited) the recommended dose should be increased to 50 μg/day (2000 IU/day), or higher [13]. A noticeable effort has been made to achieve better standardization in vitamin D measurement over recent years [14]. Even though toxic or side effects are relatively rare, measurement of vitamin D levels helps monitor treatment and helps prevent overdose [15]. Therefore, measurement of vitamin D serum levels is appropriate. Only this described procedure can assure proper, personalized supplementation with the highest efficacy and a positive impact on the health status of patients [16].

**Conclusions and PPPM-Based Expert Recommendations**

- Vitamin D acts as a steroid hormone and is involved in many metabolic processes in the human body.
- PPPM principles can serve as a useful tool in the prevention of vitamin D deficiency by indicating predictive and personalized supplementation.
- Low vitamin D serum levels should be the clear predictive factor for supplementation in each age group; from newborns to the elderly, and not only in childhood, as was previously routinely applied.
- Methods for vitamin D monitoring are currently available in almost every laboratory. When supplementation of vitamin D is indicated, the monitoring of vitamin D (calcidiol) serum levels is strictly recommended to assure optimally personalized vitamin D doses.
- The toxicity of vitamin D was overstated in the past. Recent research has emphasized the optimal vitamin D levels required to assure its proper effect in all the vitamin D related metabolic processes.

**References**

1. Bouillon R, Marcocci C, Carmeliet G, Bikle D, White JH, Dawson-Hughes B, et al. Skeletal and extraskeletal actions of vitamin D: current evidence and outstanding questions. Endocr Rev. 2018;40:1109–51.
2. Wacker M, Holick MF. Sunlight and Vitamin D. Dermatoendocrinol. 2013;5:51–108.
3. Koduah P, Paul F, Dörr J-M. Vitamin D in the prevention, prediction and treatment of neurodegenerative and neuroinflammatory diseases. EPMA J. 2017;8:313–25.
4. Chiang C-M, Ismaeel A, Griffis RB, Weems S. Effects of vitamin D supplementation on muscle strength in athletes: a systematic review. J Strength Cond Res. 2017;31:566–74.
5. Martineau AR, Jolliffe DA, Hooper RL, Greenberg L, Aloia JF, Bergman P, et al. Vitamin D supplementation to prevent acute respiratory tract infections: systematic review and meta-analysis of individual participant data. BMJ 2017; 10.1136/bmj.i6583.
6. Golubnitschaja O, Costigliola V, EPMA. General report & recommendations in predictive, preventive and personalised medicine 2012: white paper of the European Association for Predictive, Preventive and Personalised Medicine. EPMA J. 2012;3:14.
7. Golubnitschaja O, Baban B, Boniolo G, Wang W, Bubnov R, Kapalla M, et al. Medicine in the early twenty-first century: paradigm and anticipation - EPMA position paper 2016. EPMA J. 2016;7:23.
8. Feldman D, Krishnan AV, Swami S, Giovannucci E, Feldman BJ. The role of vitamin D in reducing cancer risk and progression. Nature Reviews Cancer. 2014;14:342–57.
9. Janssens JP, Schuster K, Voss A. Preventive, predictive, and personalized medicine for effective and affordable cancer care. EPMA J. 2018; 10.1007/s13167-018-0130-1.
10. Seifirad S, Haghpanah V. Inappropriate modeling of chronic and complex disorders: How to reconsider the approach in the context of predictive, preventive and personalized medicine, and translational medicine. EPMA J. 2019;10:195–209.
11. Leary PF, Zamfirova I, Au J, McCracken WH. Effect of Latitude on Vitamin D Levels. J Am Osteopath Assoc. 2017;117:433–9.
12. Molacek J, Treska V, Zeithaml J, Hollan I, Topolcan O, Pecen L, et al. Blood biomarker panel recommended for personalized prediction, prognosis, and prevention of complications associated with abdominal aortic aneurysm. EPMA J. 2019; 10.1007/s13167-019-00173-2.
13. Pludowski P, Holick MF, Grant WB, Konstantynowicz J, Mascarenhas MR, Haq A, et al. Vitamin D supplementation guidelines. J Steroid Biochem Mol Biol. 2018;175:125–35.
14. Binkley N, Dawson-Hughes B, Durazo-Arvizu R, Thamm M, Tian L, Merkel JM, et al. Vitamin D measurement standardization: The way out of the chaos. J Steroid Biochem Mol Biol. 2017;173:117–21.
15. Kennel KA, Drake MT, Hurley DL. Vitamin D Deficiency in Adults: When to Test and How to Treat. Mayo Clin Proc. 2010;85:752.
16. Marcus-Kalish M, Gozes I. Multilevel, Multisource Bio- Medical rule discovery system – essential enabler to preventive, predictive and personalized medicine. EPMA J. 2011;2:186.

**Acupuncture in the complex treatment of elderly patients with chronic cerebral ischemia. Personalized approach**

Smirnova N*^1^, Mikhailova A^1^, Khrypunova O^1^

^1^I.M. Sechenov First Moscow State Medical University, Russian Federation

*Corresponding author: Dr. N Smirnova, I.M. Sechenov First Moscow State Medical University, Russian Federation; e.mail: smirnovanp61@bk.ru

**Keywords:** personalized preventive participative medicine, polymorbidity, chronic cerebral ischemia, depression, acupuncture, pharmacopuncture.

**The aim of study was** to develop personalized treatment schemes using the acupuncture (AP) in combination with pharmacopuncture (PhP) and psycho-pharmacotherapy (PPhT) in order to reduce number of side-effects, relieve depression and microcirculation disorders for elderly patients with polymorbid pathology and provide evidence of the effectiveness of complex treatment in comparison with PPhT.

**The study outlines.** The study included 127 patients (30 men and 97 women), average age 65 ± 4.6 years, treatment duration from 5.4 to 11.5 years. Patients were treated at the Center of borderline disorders with a diagnosis of chronic cerebral ischemia (CHCI) (ICD - 10), diabetes mellitus of type 2 [1,2]. The Minnesota Multiphasic Personality Inventory questionnaire was used for the personalized treatment approach. The questionnaire helped to reveal anxiety-depressive syndrome in 52 people and depressive-hypochondria syndrome in 75 patients.

Patients who were assigned (according to the randomization procedure) to the control group (*n* = 30) received only PPhT: antidepressants, tranquilizers, nootropics, vasoactive, hypoglycemic drugs. The treatment group of 97 patients was divided into three subgroups split by acupuncture treatment methods in combination with PPhT:

- Group 1, received AP, (*n* = 34): the course consisted of 10–12 sessions, conducted every other day;
- Group 2 received PhP (*n* = 32) with antihomotoxic drugs (AHTD) from the Biologische Heilmittel Heel GmbH company according to the developed recommendations [2]: Traumeel S, Cerebrum compositum N. Injections were performed into the area of acupuncture points at 0.3–0, 5 ml each. The course duration was 4 weeks, twice a week;
- Group 3 received a combination of AP and PhP, (*n* = 31): pharmaco-puncture sessions were held three times a week, simultaneously with acupuncture sessions lasting 4 weeks.

According to the PHQ-9 health questionnaire, different level of depression were observed: mild in 11% patients, moderate in 47.3%, middle severity in 37%, severe depression in 4.7%.

Cerebral microcirculation (CMC) was examined using a Slit Lamp SL-45 Shin Nippon (Japan). The conjunctival microcirculation index (CMI) was calculated; 63.8% of patients had mild CMC disorders, and 36.2% of patients had moderate severity CMC. Among patients with anxiety-depressive syndrome, spastic characteristics in the microvasculature were prevailed. In the patient group with depressive-hypochondriac syndrome, disorders of venous outflow and rheology prevailed. A decrease in the number of functioning capillaries was observed in 28 patients with severe asthenic symptoms. Microcirculatory disorders with different qualitative and quantitative characteristics were regarded as predictors of treatment effectiveness. Depression rates and CMC parameters were evaluated on the 28th day.

**Results**

Clinical indicators improved significantly in treatment groups: headache, dizziness and gait unsteadiness decreased 4–5 days earlier than in the control group (*p* < 0.05); blood pressure stabilized in the AP groups after 6th–8th session (see summarising Fig. 1).
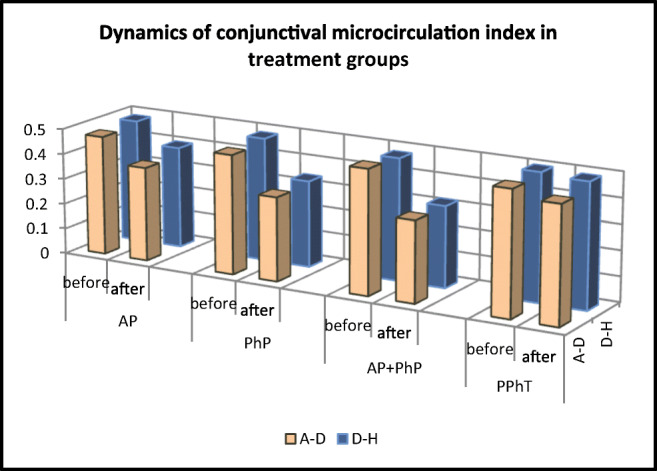


**Fig. 1** Dynamics of conjunctival microcirculation index (CMI) in the main and control groups with various psychopathological syndromes (*p < 0.05) before and after treatment. Footnote: **A–D** – patients with anxious-depressive syndrome. **D–H** – patients with depressive-hypochondriac syndrome. **PPhT –** control group. **AP** – acupuncture group. **PhP** – pharmacopuncture group. **AP** + **PhP** – acupuncture and pharmacopuncture group

Complex therapy including PhP and a combination of AP + PhP had a significant effect for the reduction of microcirculatory disorders. Using AP in patients with anxiety-depressive syndrome, CMI decreased by 21.3%, and in patients with depressive-hypochondriac syndrome, CMI decreased by 17.5%. The therapeutic effect was reached faster and was more long-lasting due to steady anti-depressive and vegetative-stabilizing effects.

In the control group, the decrease in microcirculation disorders was not significant, and PPhT reduced the severity of anxiety and depression. However, the control group could not get a pronounced improvements in affective and vascular disorders by the 28th day in comparison with AP groups. Patients felt weakness and decreased appetite.

Improvements in clinical and psychological symptoms and in the level of microcirculation were observed earlier in AP groups: for patients with anxiety-depressive syndrome results were achieved at the 6–7 session, for patients with depressive-hypochondria syndrome results were achieved at the 8–9 session. Combined therapy significantly reduced the side-effects of PPhT [3,4] and significantly increased adherence to treatment at the same time. This specificity of AP determined the compatibility between patients and physician’s efforts to overcome risk-factors.

**Conclusions**

1. The use of AP and PhP with antihomotoxic drugs helped to improve the clinical and psychological condition and level of the microvasculature in patients from treatment groups in shorter terms. In the control group, which used PPhT, improvement was achieved on 22 ± 1.5 day, which corresponded with the l manifestation of antidepressants efficacy.
2. The effectiveness of complex treatment, including AP and PPhT at the same time, is significantly higher for elderly patients with chronic cerebral ischemia, metabolic and depressive disorders, than in the group only taking psychopharmacological drugs.

Current research shows the role of acupuncture in the complex treatment of elderly patients. This combinated treatment correlates with principles of PPPM: a personalized approach takes into account the psychological status of the patient; it prevents side-effects of pharmacotherapy. Collaboration with patients is crucial involving them in the treatment process that helps to overcome the modifiable risk-factors effectively for patients in the treatment group.

**References**

1. Camus V, Kraehenbuhl H, Preisig M, Bula CJ, Waeber G. Geriatric depression and vascular diseases: what are the links? J Affect Disord. 2004;81(1):1–16. 10.1016/j.jad.2003.08.003
2. Duarte AA, Mohsin S, Golubnitschaja O. Diabetes care in figures: current pitfalls and future scenario. EPMA J. 2018;9(2):125–131. 10.1007/s13167-018-0133-y.
3. Sabel BA, Wang J, Cárdenas-Morales L, Faiq M, Heim C. Mental stress as consequence and cause of vision loss: the dawn of psychosomatic ophthalmology for preventive and personalized medicine. EPMA J. 2018;9(2):133–160. 10.1007/s13167-018-0136-8.
4. Agasarov LG, Davyan OS, Edelev DA. Mechanisms, efficiency and security of pharmacopuncture. J New Med Tech. 2018;25(4):110–115. 10.24411/1609-2163-2018-16280

**Do Biomarkers Contribute to a Personalized Approach to Obstructive Sleep Apnea Syndrome?**

Slouka D^1^, Gal B^3^, Betka J^4^, Topolcan O^2^, Kucera R*^2^

^1^Department of Otorhinolaryngology, ^2^Department of Immunochemistry Diagnostics, University Hospital in Pilsen, Charles University, Faculty of Medicine in Pilsen, Czech Republic

^3^Masaryk University, Faculty of Medicine, Department of Otorhinolaryngology and Head and Neck Surgery, Brno, Czech Republic

^4^Charles University in Prague, 1 st Faculty of Medicine, Department of Otorhinolaryngology and Head and Neck Surgery, Prague, Czech Republic

*Corresponding author: Radek Kucera, Ph.D., Associate Professor, Department of Immunochemistry Diagnostics, University Hospital Pilsen, Dr. E. Benese 1128/13, 305 99 Pilsen, Czech Republic; e.mail: kucerar@fnplzen.cz, ORCID: 0000-0002-2739-2302

**Keywords:** sleep apnea syndrome, obstructive sleep apnea syndrome, predictive biomarkers, pentraxin-3, primary prevention, predictive factor, positive airway pressure therapy, patient stratification, genetic burden, personalized treatment

**Introduction**

Sleep Apnea Syndrome (SAS) is defined as repeated apnea breathing during sleep lasting more than 10 s. The incidence of SAS in the adult male population over 40 years of age is 4% and in the female population after menopause 2%. We distinguish 3 forms of SAS: obstructive (80%), central (10%) and mixed (10%). Risk factors include obesity, large neck circumference and anatomical obstruction in the upper airways. Cardiovascular and metabolic disorders are common comorbidities of OSAS [1].

The basic diagnosis of OSAS consists of upper airway patency and sleep monitoring. Lighter forms of the condition are indicated for surgical treatment while in heavier forms, positive airway pressure therapy (PAP) is preferred. Obese individuals need to lose weight and adopt a healthy lifestyle. The current OSAS diagnostics is time consuming, placing high demands on personnel and technology. In the past decade, the principles of personalized medicine have begun to be applied to OSAS. The first task should be to improve prevention by emphasizing a healthy lifestyle that minimizes OSAS risk factors [2]. Personalized treatment, taking into account individual conditions such as ethnic, gender, genetic or phenotypic differences, can bring increased benefits to the patient [3]. The use of biomarkers in OSAS diagnostics within the framework of the principles of preventive, predictive, personalised medicine is a field that is insufficiently explored [4]. We decided to study the ability of selected biomarkers to identify OSAS patients among a wider cohort of healthy people. The following blood biomarkers were tested: C-reactive protein (CRP), pentraxin-3 (PTX-3), interleukin-6 (IL-6), high sensitive troponin (hsTnI) and brain natriuretic peptide (BNP).

**Materials and methods**

In total we evaluated the results of 134 persons. The control group consisted of 70 persons, the OSAS group of 64 patients. Inclusion criteria: OSAS indicated for the treatment of positive airway pressure (PAP) (apnea-hypopnea index, AHI ≥ 15), no prior OSAS conservative treatment, no prior OSAS surgical treatment, complete sleep monitoring results. AHI is defined as the average number of apnea-hypopnoea events per hour of sleep. Exclusion criteria: incomplete documentation of sleep monitoring, chronic obstructive pulmonary disease, previous OSAS treatment, previous upper respiratory tract surgery, patient non-compliance. The age characteristics of the compared groups are shown in Table 1. The age of the compared groups did not differ significantly (*p* = 0.1443). Peripheral blood was drawn and after serum separation the levels of CRP, PTX-3, IL-6, hsThI and BNP were determined.

**Table 1** Age characteristics of the studied group

**Table:** 

Studied Group	Count	Age (years)			p-value Wilcoxon test
		Mean	Minimum	Maximum	
OSAS group	*64*	57.5	32.0	86.0	*p* = 0.1352
Control group	*70*	56.5	38.0	81.0	

**Results**

Table 2 shows the comparison of the biomarker levels between the OSAS group and the group of healthy persons. With the exception of BNP, all the examined biomarkers had statistically significantly higher levels in the OSAS group compared to the control group. We calculated the AUC for each individual biomarker. The values of the AUC are shown in Table 3. The best values were achieved by PTX-3 (AUC = 0.7346). We created a multivariate model using the logistic stepwise regression. The AUC of the model was the highest (AUC = 0.7679) but with no clinical relevance compared to the best individual marker PTX-3 (*p* < 0.5556).

**Table 2** Biomarker levels: OSAS group vs. control group

**Table:** 

Biomarker	Status	Mean	Median (Min–Max)	p-value Wilcoxon test
CRP (mg/l)	OSAS	6.52	3.69 (0.19–16.9)	<0.0001
	Controls	2.19	1.82 (0.11–14.2)	
PTX-3 (pg/ml)	OSAS	5312	5126 (592–13752)	<0.0001
	Controls	3136	2785 (598–11234)	
IL-6 (pg/ml)	OSAS	3.24	2.63 (0.11–64.5)	0.0013
	Controls	2.15	1.12 (0.11–15.1)	
hsTnl (ng/l)	OSAS	6.25	3.59 (0.60–59.2)	<0.0001
	Controls	3.32	2.14 (0.45–39.6)	
BNP (pg/ml)	OSAS	27.6	11.3 (5.59–756)	0.6925
	Controls	19.1	10.2 (10.0–159)	

**Table 3** AUC values

**Table:** 

Parameter	AUC
PTX-3	0.7346
CRP	0.7102
hsTnI	0.6738
IL-6	0.6284
BNP	0.4652
Multi-parameter Model	0.7679

*p* value PTX-3 vs. 3-parameter model: *p* = 0.5556

**Data Interpretation**

The panel of biomarkers was selected based on their close relation to the cardio- and cerebrovascular complications of OSAS. Sahlman et al. [5] have explored CRP potential for use with OSAS. The authors demonstrate a relation to OSAS, but with no significant benefit for diagnosis. These results are in line with our conclusions. PTX-3 is secreted in damaged tissue. In agreement with our results, Kanbay [6] and Kobukai [7] reported the benefit of PTX-3 for OSAS diagnosis. IL-6 is involved in the activation of “acute phase” proteins. In our study, in agreement with Liu [8], we demonstrated the association between IL-6 and OSAS, but with no wider clinical utility. The new generation of highly sensitive troponin I assay has allowed for the extension of indications from myocardial infarction detection to small myocardial cell damage, particularly in connection with a number of chronic diseases. Whereas Maeder [9] did not show a significant increase in hsTnI, we demonstrated the statistically significant increase of hsTnI in the OSAS group. Serum BNP levels were not associated with the presence of OSAS in our study. We created a multi-parameter model, a proven tool of the personalized approach in diagnostics [10], using PTX-3, CRP and hsTnI. This model achieved the highest AUC but we still have to prove the statistical significance in a larger cohorts.

**Conclusions and PPPM-based expert recommendations**

- The primary prevention of OSAS should be improved by placing a preventive emphasis on a healthy lifestyle.
- Currently, sleep monitoring has no comparable alternative. There are, as yet, no simple applicable predictive factors for the stratification of patients and the design of personalized treatment.
- PTX-3 serum levels were identified as one possible biomarker for the above mentioned purpose.
- The multi-parameter model looks promising, but we need larger cohorts to prove its ability to identify patients with OSAS.
- Early diagnosis of OSAS made using technically simple methods will allow a higher level of personalization of treatment and thereby higher treatment efficiency.

**Acknowledgements:** Supported by Ministry of Health, Czech Republic - conceptual development of research organization (Faculty Hospital in Pilsen - FNPl, 00669806) and BBMRI-CZ: Biobank network - a versatile platform for the research of the etiopathogenesis of diseases CZ.02.1.01/0.0/0.0/16_013/0001674.

**References**

1. Epstein LJ, Kristo D, Strollo PJ Jr., Friedman N, Malhotra A, Patil SP, et al. Adult Obstructive Sleep Apnea Task Force of the American Academy of Sleep Medicine. Clinical guideline for the evaluation, management and long-term care of obstructive sleep apnea in adults. J Clin Sleep Med. 2009;5:263–76.
2. Golubnitschaja O. Time for new guidelines in advanced healthcare: the mission of The EPMA Journal to promote an integrative view in predictive, preventive and personalized medicine. EPMA J. 2012; 10.1186/1878-5085-3-5.
3. Golubnitschaja O, Baban B, Boniolo G, Wang W, Bubnov R, Kapalla M, et al. Medicine in the early twenty-first century: paradigm and anticipation - EPMA position paper 2016. EPMA J. 2016;7:23.
4. Golubnitschaja O, Costigliola V, EPMA. General report & recommendations in predictive, preventive and personalized medicine 2012: white paper of the European Association for Predictive, Preventive and Personalized Medicine. EPMA J. 2012; 10.1186/1878-5085-3-14.
5. Sahlman J, Miettinen K, Peuhkurinen K, Seppä J, Peltonen M, Herder C, et al. Sleep Apnoea Group. The activation of the inflammatory cytokines in overweight patients with mild obstructive sleep apnoea. J Sleep Res. 2010; 10.1111/j.1365-2869.2009.00787.
6. Kanbay A, Kaya E, Büyükoğlan H, Kaya MG, Şimşek ZÖ, Tutar N, et al. Correlation between pentraxin-3 and endothelial dysfunction in obstructive sleep apnea syndrome. Ann Thorac Med. 2015; 10.4103/1817-1737.160840.
7. Kobukai Y, Koyama T, Watanabe H, Ito H. Morning pentraxin3 levels reflect obstructive sleep apnea-related acute inflammation. J Appl Physiol. 2014; 10.1152/japplphysiol.00237.2014.
8. Liu H, Liu J, Xiong S, Shen G, Zhang Z, Xu Y. The change of interleukin-6 and tumor necrosis factor in patients with obstructive sleep apnea syndrome. J Tongji Med Univ. 2000;20:200–2.
9. Maeder MT, Strobel W, Christ M, Todd J, Estis J, Wildi K, et al. Comprehensive biomarker profiling in patients with obstructive sleep apnea. Clin Biochem. 2015; 10.1016/j.clinbiochem.2014.09.005.
10. Golubnitschaja O, Polivka J Jr., Yeghiazaryan K, Berliner L. Liquid biopsy and multiparametric analysis in management of liver malignancies: new concepts of the patient stratification and prognostic approach. EPMA J. 2018; 10.1007/s13167-018-0146-6.

**Integrated aging rate index as a basis for personalized medicine technologies selection for active longevity**

Nikolaev VA*

*Corresponding author: Sechenov University, Moscow, Russia; e.mail: managervit@mail.ru

**Keywords:** predictive, preventive and personalized medicine (PPPM), preventive active longevity technology, predictive biomarkers, markers of premature aging, patient stratification, individualized patient profile.

**Introduction**

Traditional medicine is aimed at the treatment of diseases and, to a lesser extent, at their prevention. The future is for predictive, preventive and personalized medicine [1–3]. The biomarkers can be used as a first criterion in evaluation of the development of an anti-premature aging approach in personalized medicine and to determine, monitor and track the aging process. However, it is essential to demonstrate the clinical utility of biomarkers prior to their implementation in medical services [1, 2]. Recent studies show that the use of only biomarkers is not enough in developing technologies of active longevity and personalized medicine. Moreover, the publications in the field have highlighted the value of predictive, preventive and personalized medicine and its effects on quality of life, active aging and life extension [4–6]. This paper introduces an approach for developing active longevity technology based on the determination of the aging rate index.

**Methods**

Analytical methods have been used to analyze advantages and disadvantages of existing medical methods for assessment of biological age and aging rate index as well as current trends and technologies of personalized medicine. Moreover, a significant number of medical methods have been examined: clinical methods, in/out lab-based testing methods and procedures, methods of biomarkers assessment, biomarkers group of physical examinations methods, olfactory test, mental health and cognitive disorders tests, and biological/chronological age estimation techniques.

**Results and discussion**

The aging process affects all the systems of a human being. In general, an increase in chronological age results in an increase in biological age. It means a reduction of physical and mental health of individuals; however, the aging rate can be different.

The results revealed that the aging rate index is a complex indicator calculated as an integrated index from a number of following medical tests for a particular person at a specific chronological age. In general, the calculation of the aging rate index consists of the following stages: firstly, a selection of specific indicators from which the integral indicator is built, secondly, a transformation of indicators for the purpose of comparability is needed, and finally, choosing a method for aggregating the transformed specific indicators to unify the index is required. The transformation stage is essential and complex due to the fact that different specific indicators are measured using different methods and units. It is also important to have insignificant difficulties interpreting the overall results of four or more biomarkers which are monitored continuously on a system basis.

Further studies should consider a number of tests. The biomarkers (plasma albumin, alpha-1 acid glycoprotein, particle size of low-density lipoproteins and citrates) are typically determined based on a blood test (BT). A number of molecular biomarkers relating but not limited to biological age are as follows: a leukocyte telomere length, γ-H2A.X, DNA methylation, heterogeneity of CD38 in CD4^+^ CD27+ T cells, miR-34a, miR-21, miR-126-3p and MIR31HG [7]. In addition, a mental test (MT) is used to assess the contribution of the current cognitive abilities [8]. For instance, 25–30% of people aged 85 or older suffer from dementia diseases [9].

An olfactory test (OT) allows assessing age-related changes [10], which are typically pronounced at the age of 65–80, while an endurance test (ET) determines a reduction in physical ability due to daily physical activities, stress and fatigue. Furthermore, the results of those tests vary for different individuals and relate to genetic and epigenetic factors. Thus, a personalized approach to the results of four tests at different periods of the chronological age of a person allows calculating the aging rate index:

$$ \mathrm{ARI}=f\left(\mathrm{BT},\mathrm{MT},\mathrm{OT},\mathrm{ET}\right) $$

As a result a complex plan of medical and social activities aimed at reducing the rate of aging and extend the active longevity of a person can be developed. The studies show that ARI can be tailored for the needs of individuals in terms of personalized medicine, active aging and life longevity. It appears from the studies that individuals can receive a targeted and precise therapy, rehabilitation, physical excursuses, social programs, environment and other technologies of personalized medicine.

Moreover, the integrated aging rate index with respect to personalized medicine is a useful tool for medical professionals and policymakers to monitor outcomes of the public health of society and take actions for the development and implementation of PPPM at the local, regional or country level.

**Conclusions and outlook**

The results revealed that it is hard to choose a specific aging index, which reflects all the aspects of aging of human being. The integrated aging rate index that was proposed in the paper can be taken under consideration as an attempt to develop a basis for choosing the technologies of personalized medicine for active aging and longevity. The index incorporates a number of markers for a given test, which are essential to carry out. Furthermore, the integrated aging rate index can be a useful tool for policymakers to monitor outcomes of public health organizations and society and take actions for the development and implementation of PPPM at the regional or country level. Additionally, the future work will concentrate on developing and testing the models of personalized medicine for active aging. We are confident that our research will also serve as a base for future studies on predictive, preventive and personalized medicine and consolidate professionals in the field.

**References**

1. Golubnitschaja O, Kinkorova J, Costigliola V. Predictive, preventive and personalised medicine as the hardcore of ‘Horizon 2020’: EPMA position paper. EPMA J. 2014;5(1):6. 10.1186/1878-5085-5-6.
2. Topolcan O, Pesta M, Svobodova S. Conference scene: ISOBM 2013 in Israel: part 1: biomarkers - where we are now? Biomark Med. 2013;7(3):403–6. 10.2217/bmm.13.38.
3. Bürkle A, Moreno-Villanueva M, Bernhard J, Blasco M, Zondag G, Hoeijmakers JH, et al. MARK-AGE biomarkers of ageing. Mech Ageing Dev. 2015;151:2–12. 10.1016/j.mad.2015.03.006.
4. Golubnitschaja O. Paradigm change from curative to predictive medicine: novel strategic trends in Europe. Croat Med J. 2009;50(6):596–597. 10.3325/cmj.2009.50.596.
5. Golubnitschaja O, Costigliola V. European strategies in predictive, preventive and personalised medicine: highlights of the EPMA World Congress 2011. EPMA J 2011;2(4):315–332. 10.1007/s13167-011-0134-6.
6. Golubnitschaja O, Costigliola V, Grech G. EPMA World Congress: traditional forum in predictive, preventive and personalised medicine for multi-professional consideration and consolidation. EPMA J. 2017;8:(Suppl):1–54. 10.1007/s13167-017-0108-4.
7. Xia X, Chen W, McDermott J, Jackie Hana J.D. Molecular and phenotypic biomarkers of aging. F1000Res. 2017;6:860. 10.12688/f1000research.10692.1.
8. Cole JH, Franke K. Predicting age using neuroimaging: innovative brain ageing biomarkers. Trends Neurosc. 2017;40(12):681–690. 10.1016/j.tins.2017.10.001.
9. World Health Organization. https://www.who.int/ageing/publications/global_health.pdf.
10. Doty RL, Kamath V. The influences of age on olfaction: a review. Front Psychol. 2014;5:20. 10.3389/fpsyg.2014.00020.

**The role of** ^**18**^**F-FDG-PET/CT in detection of the cause of sepsis and its impact on choice of treatment**

Ferdova E^1^, Jan Baxa J^1^, Matejovic M^2^, Topolcan O^3^, Ferda J*^1^

^1^Department of Medical Imaging, ^2^First Internal Medicine Department, ^3^Department of Immunochemistry Diagnostics, University Hospital in Pilsen, Medical Faculty in Pilsen, Charles University, Czechia

*Corresponding author: Prof. Jiri Ferda, M.D., Ph.D., Department of Medical Imaging, University Hospital in Pilsen, Czechia; e.mail: ferda@fnplzen.cz

**Keywords:** preventive predictive and personalized medicine (PPPM), ^18^F-FDG-PET/CT, hybrid imaging, patient stratification, sepsis, systemic inflammatory response, metastatic spread, targeted prevention, personalized triggering, personalized treatment

**Introduction**

Sepsis and bacteremia of unknown origin remain a serious problem in current healthcare. The prolonged investigations to discover the cause behind these conditions can lead to a serious delay in starting targeted therapy resulting in increased morbidity and mortality [1]. Previous studies have confirmed that PET/CT with the application of ^18^F-fluordeoxyglucose (^18^F-FDG-PET/CT) is superior to computed tomography, magnetic resonance or conventional scintigraphy in detecting hidden inflammatory processes [2, 3]. An analogous approach to patients with sepsis of unknown origin could be used. Such an imaging should detect the source of bacteremia which remains hidden on conventional imaging methods [2]. In order to select a personalized treatment for patients suffering from symptoms of sepsis of unknown origin, ^18^F-FDG-PET/CT should be used in the localization of the primary focal inflammation, and/or of the metastatic spread of the infection within the same organ system, or multi-systemic dissemination [4]. Moreover, personalized treatment is then tailored to the affected organ or organ system using the targeted microbiological sampling (including the investigation of antimicrobial treatment sensitivity), local surgical, or mini-invasive therapy. Our investigation was aimed at evaluating the value of ^18^F-FDG-PET/CT performed early in the detection of the cause of sepsis of unknown origin and at assessing how the results may contribute to personalized treatment.

**Material and method**

*Patient population*

Our cohort was included a total of 50 patients (27 males, 23 females, mean age 56.6 years, ranging 26–84 years) with SIRS of suspected or confirmed bacterial origin. Imaging was performed when conventional clinical or imaging strategy did not explain the patients’ condition within 72 h from the actual onset of SIRS symptoms.

*PET/CT procedure*

We used PET/CT (BiographmCT 128 UltraHD, Siemens Healthineers, Erlangen, Germany) for the hybrid imaging. The glucose blood levels control was followed by an intravenous application of ^18^F-fluorodeoxyglucose at 2.5 MBq/kg per body weight. The data acquisition started 60 min after the radiopharmaceutical application. The imaging covered the whole body from vertex to toe. The data acquisition starts with a CT after intravenous application of 80 ml of the iodinated contrast material (Ultravist 370, Bayer Pharma, Berlin, Germany), except in patients with renal failure, or with a documented allergic reaction to iodinated contrast material.

*Analysis of the diagnostic performance*

The laboratory findings prior to the imaging and during the disease were documented in the digital clinical information system. The retrospective analysis was based on a comparison between the ^18^F-FDG-PET/CT findings and the clinical course of the disease, response to the therapy (including documented changes after ^18^F-FDG-PET/CT imaging) and microbiology lab results, including cultivation and/or PCR. ^18^F-FDG-PET/CT results were assumed to have a strong correlation to the cause of the patient’s clinical state. The following modifications in therapy were considered triggered therapy: change of antibiotics, surgery or mini-invasive abscess drainage.

**Results**

^18^F-FDG-PET/CT results contributed to the detection of the cause of sepsis of unknown origin in 46 patients (resulted in 92%). Results are summarized in Tables 1–3. Despite unexplained causes of sepsis, negative findings that helped exclude the possibilities of inflammation in the musculoskeletal system, including the vertebral column, and/or inflammation within the lungs, liver or bowel, have helped personalized treatment in patients.

**Table 1** List of findings

**Table:** 

Finding of PET/CT	No.	%
Cause undetected	4	8.00
Positive findings Monofocal Multifocal Multisystemic	46 8 31 7	92.00 17.39 67.39 15.22
Spondylodiscitis	15	32.61
Musculoskeletal infection Pyogenous artritis Pyomyositis Joint prosthesisi infection	13 3 3 8	28.26 6.52 6.52 17.39
Cadiovascular infection Endocarditis Myco otis aneurysma Vascular graft infection	10 4 3 3	21.74 8.70 6.52 6.52
Other findings GIT infection Lung infection Hodgkin lymphoma	8 4 3 1	17.39 8.70 6.52 2.17

**Table 2** Microbial agents confirmation and treatment modification

**Table:** 

Microbial agens confirmation (of 45)	42	93.33
Haemoculture Local sampling (icl. BAL, FNB or drainage) PCR	24 16 2	53.33 35.56 4.44
Treatment modified (of 50)	28	56
Switched antibiotic therapy Surgical intervention Image giuded drainage Oncological treatment	9 13 5 1	18 26 10 2

**Table 3** Microbial agents

**Table:** 

Microbial agens (of 42)	No.	%
*Staphylococcus aureus* Methiciline resistant Methiciline sensitive	22 13 9	52.38 30.95 21.43
*Streptococus faecalis*	7	16.67
*Streptococcus pneumoniae*	3	7.14
*Escherichia coli*	3	7.14
*Klebsiella pneumonia*	5	11.90
*Mycobacterium tuberculosis*	2	4.76

**Data Interpretation**

The use of 18F-FDG-PET/CT has been discussed in several studies in relation to the detection of uncertain causes of fever, bacteremia or inflammation, yet only a few studies have successfully exploited ^18^F-FDG-PET/CT in the indication of sepsis of unknown origin, or shortly after SIRS occurred [5, 6]. Precise evaluation of the extent of inflammatory focuses forms the basis of a personalized treatment strategy. Early use of ^18^F-FDG-PET/CT in predictive diagnostics will lead to the personalized selection of local treatment alongside systemic causal anti-microbial therapy.

We might have had more findings than other studies focused on^18^F-FDG-PET/CT for two reasons: the selection of ^18^F-FDG-PET/CT sooner than in other studies and the inability to perform an examination in critically ill patients under artificial ventilation [4, 5].

The effectivity of the ^18^F-FDG-PET/CT could be high regardless of the state of the patients’ immune system. Immunocompetent patients exhibit a strong inflammatory response to common infections as well as to nosocomial microbial organisms [8]. In addition, patients with a known impaired immune response tend to have positive PET/CT findings. It has been shown in several studies that the infective foci could be multiple and multi-systemic. Multifocal septic arthritis or spondylodiscitis, or a combination of arthritis with spondylitis are typical. The hybrid imaging covering the whole body—from vertex to toe—is advantageous in such cases, because the success of systemic therapy often has to be supported with the evacuation of abscesses. Our findings of multi-focal afflictions in patients with a staphylococcal infection reflected the results of other studies [6, 7].

**Conclusions and expert recommendations**

The treatment of infection is based on the identification of the microbial cause, the extent of disease and local disease control [9]. Patients suffering from SIRS are conventionally investigated using CT or MRI, rarely with the use of scintigraphy using labelled leucocytes. Such a conventional approach leads to many inconclusive or unexplained, findings.

Given the tried and tested high diagnostic performance of^18^F-FDG-PET/CT in inflammatory diseases, this approach could be very effective in the detection of the cause of septic state and might lead to the personalized treatment [8].

- A high number of positive findings of ^18^F-FDG-PET/CT led to the changes in therapy of patients; ^18^F-FDG-PET/CT makes the diagnostics more precise.
- Targeted sampling using a fine needle biopsy could help in identifying the microbial cause of infection. In some patients, the cause of sepsis can be seen in the imaging, for example, in patients with mycobacterial spondylitis or lung infection.
- The use of ^18^F-FDG-PET/CT in the early phases of investigation offers a quicker route to the initiation of individualized therapy, enabling the reduction of hidden sites of infection not receiving local therapy
- ^18^F-FDG-PET/CT could be recommended as a reliable instrument for the personalization of therapy as well as a predictive prevention measure aimed at minimizing the patient’s burden.

**Acknowledgement:** Supported by Czech Ministry of Health by Conceptual Development of the Institution 00669806 – FN Plzen and Charles University Project Progress q39.

**References**

1. Yu JC, Khodadadi H, Baban B. Innate immunity and oral microbiome: a personalized, predictive, and preventive approach to the management of oral diseases. EPMA J. 2019; 10.1007/s13167-019-00163-4.
2. Pijl JP, Glaudemans AWJM, Slart RHJA, Yakar D, Wouthuyzen-Bakker M, Kwee TC. FDG-PET/CT for Detecting an Infection Focus in Patients With Bloodstream Infection: Factors Affecting Diagnostic Yield. Clin Nucl Med. 2019;44:99–106.
3. Ferda J, Baxa J, Ferdova E, Kucera R, Topolcan O, Molacek J. Abdominal aortic aneurysm in prostate cancer patients: the “road map” from incidental detection to advanced predictive, preventive, and personalized approach utilizing common follow-up for both pathologies. EPMA J. 2019; 10.1007/s13167-019-00193-y.
4. Tsai HY, Lee MH, Wan CH, Yang LY, Yen TC, Tseng JR. C-reactive protein levels can predict positive (18)F-FDG PET/CT findings that lead to management changes in patients with bacteremia. J Microbiol Immunol Infect. 2018;51:839–846.
5. Tseng JR, Chen KY, Lee MH, Huang CT, Wen YH, Yen TC. Potential usefulness of FDG PET/CT in patients with sepsis of unknown origin. PLoS One. 2013; 10.1371/journal.pone.0066132.
6. Colombo J, Elzi L, Treglia G, Perren A. Light in the dark: (18)F-FDG PET/CT in *Staphylococcus aureus* bacteremia of unknown origin. Intensive Care Med. 2018;44:488–489.
7. Berrevoets MAH, Kouijzer IJE, Aarntzen EHJG, Janssen MJR, De Geus-Oei LF, Wertheim HFL, Kullberg BJ, Oever JT, Oyen WJG, Bleeker-Rovers CP. (18)F-FDGPET/CT Optimizes Treatment in *Staphylococcus aureus* Bacteremia and Is Associated with Reduced Mortality. J Nucl Med. 2017;58:1504–1510.
8. Kouijzer IJ, Vos FJ, Janssen MJ, van Dijk AP, Oyen WJ, Bleeker-Rovers CP. The value of 18F-FDG PET/CT in diagnosing infectious endocarditis. Eur J Nucl Med Mol Imaging. 2013;40:1102–1107.
9. Abdelhamid AG, El-Masry SS, El-Dougdoug NK. Probiotic Lactobacillus and Bifidobacterium strains possess safety characteristics, antiviral activities and host adherence factors revealed by genome mining. EPMA J. 2019; 10.1007/s13167-019-00184-z.

**Clinical effectiveness evaluation of remineralization therapy in children with oncology disorders undergoing chemotherapy**

Vinnichenko Y^1^, Kupets T*^2^, Alexandrova O^1^, Matelo S^2^

^1^Central Research Institute of Dental and Maxillofacial Surgery,

^2^WDS Laboratory, Moscow, Russia

*Corresponding author: Prof. Dr. T. Kupets, ^2^WDS Laboratory, Moscow, Russia

Cancer chemotherapy can increase the risks of dental diseases, which calls for enhanced preventive methods. Three different products were tested and compared to select the most effective for these particular conditions.

Osteosarcoma and Ewing’s sarcoma patients admitted in Blokhin National Medical Research Center of Oncology, in September–December 2016, were offered to participate in the caries prevention program while undergoing chemotherapy. 115 children aged 12–17 signed up and randomly joined groups 1–3. Refusers formed the control group (4).

Group 1 used a remineralizing gel containing Xylitol and Calcium Glycerophosphate. Group 2 used a dental cream containing Casein Phosphopeptides-Amorphous Calcium Phosphate (CPP-ACP). Group 3 used a 5% sodium fluoride liquid compound. Group 4 used no special caries prophylaxis method. All participants were instructed on proper toothbrushing techniques.

The average baseline DMFT for the four groups range from 4.04 to 4.48 with no significant difference. After 12 months, groups 1 and 4 (*р* = 0.0017), 2 and 4 (*р* = 0.005), 3 and 4 (*р* = *р* = 0.009), 1 and 3 (*р* = 0.009) showed significant statistical differences in DMFT values. No significant statistical differences (*р* > 0.05) were found for groups 1 and 2, 2 and 3.

As a result of the prophylaxis undertaken, groups 1, 2, and 3, achieved a reduction in caries increment of 67.7%, 52.3%, and 46.2%, respectively, compared to the control group.

It follows that the Xylitol and Calcium Glycerophosphate containing gel is more effective in dental caries prophylaxis in children undergoing cancer chemotherapy compared to both the CPP-ACP and the 5% sodium fluoride containing products.

## 3PM in oncology

**Multiomics analysis of energy metabolism heterogeneity and its molecular pattern biomarkers in ovarian cancer: contribution to the 3PM approach in disease management**

Li N^1,2^, Zhan X*^1,2^

^1^ University Creative Research Initiatives Center, Shandong First Medical University, 6699 Qingdao Road, Jinan, Shandong 250117, P. R. China

^2^ Key Laboratory of Cancer Proteomics of Chinese Ministry of Health, Xiangya Hospital, Central South University, 87 Xiangya Road, Changsha, Hunan 410008, P. R. China

*Corresponding author: Xianquan Zhan, University Creative Research Initiatives Center, Shandong First Medical University, 6699 Qingdao Road, Jinan, Shandong 250117, P. R. China; e.mail: yjzhan2011@gmail.com

**Keywords:** predictive preventive personalized medicine, multiomics, genomics, transcriptomics, proteomics, metabolomics, interactomics, signaling pathway, molecular network, biomarker, ovarian cancer, energy metabolism heterogeneity, Warburg effect, reverse Warburg effect, glycolysis pathway, Krebs cycle pathway, oxidative phosphorylation (OXPHOS) pathway, early diagnosis, prognostic assessment, lncRNA, miroRNA, RNA-binding protein, SNHG3, EIF4A3

**Multi-parameter systematic opinion of ovarian cancer**

Ovarian cancer (OC) is a serious malignant tumor in women with multi-causes, multi-processes, and multi-consequences, which is involved in a series of molecular changes in DNAs, RNAs, proteins, and metabolites, and those molecules function mutually in a molecular network system [1–3]. It is very difficult to use a single-one parameter model for accurately predictive, preventive, and personalized treatment [4], and even for a tumor pathophysiological feature such as chronic inflammation in cancer [5]. The multi-parameter system model is necessary to meet the real situation of an OC [6]. The development of multiomics and systems biology has driven the rapid transition of a single-one model to multi-parameter systematic model. A multiomics-based molecular pathway network study will significantly drive the discovery of effective and reliable biomarkers to in-depth understand the molecular mechanism, discover effective therapeutic targets, and identify reliable biomarkers for prediction, diagnosis, and prognostic assessment for OC patients [1, 7].

**Energy metabolism abnormality in ovarian cancer**

Energy metabolism abnormality is an important pathophysiological phenomenon in OC. Traditionally, the energy supply of cancer cells is mainly derived from aerobic glycolysis, but not from the Krebs cycle and oxidative phosphorylation (OXPHOS), which is called the Warburg effect [8]. However, the Warburg effect cannot explain the energy supply of all cancer cells. Thus, a novel reverse Warburg effect was put forward in 2009 [9], which emphasizes the interactions between cancer cells and tumor-microenvironment (TME), and cancer cells and cancer-associated fibroblasts (CAFs) become metabolically coupled. Cancer cells secrete many reactive oxygen species (ROS) into TME to enhance oxidative stress in CAFs. When the inflammation, autophagy, loss of stromal caveoloin-1 (Cav-1), and nitric oxide synthase (NOS) are increased in CAFs, the aerobic glycolysis is prone to occur. As a result, CAFs secrete lots of energy-rich fuels to TME, such as lactate and pyruvate [10]. These nourishment fuels feed mitochondrial OXPHOS and ATP supplement. In this process, mono-carboxylate transporter (MCT1 and MCT4) is highly expressed in cell membrane in cancer cells and CAFs. MCT1 is upregulated specifically in cancer cells to participate in lactate uptake, whereas MCT4 is distributed specifically in CAFs to participate in lactate efflux progress [11]. Some studies also demonstrate that aerobic glycolysis is not the main energy metabolism approach for many human cancer cells [12]. The past decades’ studies regarding Warburg and reverse Warburg effects have formed the scientific frontiers and hotpot in the field of cancer energy metabolism abnormality.

**Mitochondria-based multiomics strategy for analysis of energy metabolism abnormality in ovarian cancer**

Mitochondria are the center of energy metabolism in eukaryotic cells. Mitochondrial dysfunctions are closely associated with tumor relapse, metastasis, and progression. Structural and morphological changes of mitochondria are observed inside cancer cells [13]. Further experimental evidence found that mitochondria play essential roles in growth, survival, and therapeutic treatment of OC cells [12, 14]. It is well-known that the glycolysis pathway occurs in cytoplasmic region, the Krebs cycle and OXPHOS pathways occur in mitochondria, and energy metabolism pathways are regulated by long non-coding RNAs (lncRNAs) [15], and the TCGA database contains large-scale RNA data and clinical data for several hundred OC patients [16, 17]. Thus, we designed a multiomic strategy to study energy metabolism abnormalities in human OC tissues, and energy metabolism pathways-based biomarkers (Fig. 1) [18]. (i) Quantitative mitochondrial proteomics is used to study the changes of the Krebs cycle and OXPHOS pathways [19]. The basic procedure is that mitochondria are isolated and purified from human OC and control ovary tissues. The extracted mitochondrial proteins (cancers; controls) are subjected to digestion with trypsin, 6-plex iTRAQ labeling, strong exchange cation (SCX), and liquid chromatography-tandem mass spectrometry (LC-MS/MS) analyses to identify mitochondria differentially expressed proteins (mtDEPs), followed by molecular pathway network analysis. (ii) Quantitative proteomics of human whole OC tissues is used to study the changes of the glycolysis pathway [20]. The basic procedure is the same as quantitative mitochondrial proteomics, only the mitochondrial protein samples are replaced with the whole tissue protein samples (cancers; controls), which results in identification of DEPs between whole tissues of OCs and controls, and changes in molecular pathway networks. (iii) Quantitative transcriptomics data and the complete clinical data from TCGA OC tissue RNA-seq data are used to determine survival-related lncRNAs, RNA-binding proteins, and microRNAs to be associated with energy metabolism pathways in OC tissues [16]. (iv) The changed key molecules (mRNAs; proteins) in energy metabolism pathways are validated at the OC cell and human tissue levels, with qRT-PCR and western blotting [16]. (v) Comprehensive analysis of all above data determines mulitomics-based energy metabolism heterogeneity and its biomarker pattern in human OCs.
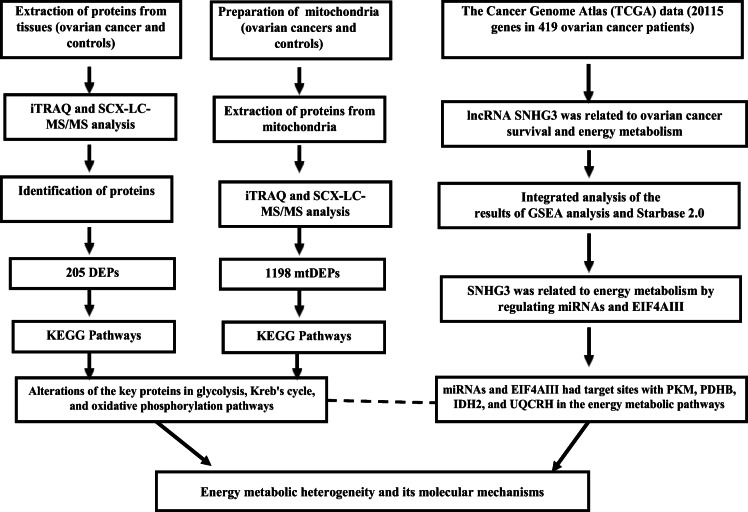


**Fig. 1** The experimental flow-chart to study energy metabolic heterogeneity and its molecular mechanisms. Reproduced from Li, Zhan, and Zhan (2018) [18], with permission from InTech-Open science publisher open access

**Molecule-pattern changes and their regulatory molecules of energy metabolism pathways in ovarian cancer**

Quantitative mitochondrial proteomics and quantitative whole tissue proteomics revealed the changes of key proteins in the energy metabolism signaling pathways [16, 20]. The Krebs cycle, and OXPHOS pathways were significantly enriched from 1198 mtDEPs, and the key proteins (PDHB, IDH2, and UQCRH) in these two pathways were significantly upregulated (Fig. 2) [16]. It is coincided with the reserve Warburg effect [9]. Glycolysis pathway was significantly enriched from 205 DEPs, and the key enzyme PKM2 in this pathway was significantly upregulated [20]. It coincides with with the Warburg effect [8]. The Warburg effect and reverse Warburg effect are complementary in energy metabolism reprogramming in OC tissues. Moreover, the key proteins (PFKP, PKM, CS, PDHB, IDH2, IDH3A, IDH3B, OGDHL, ND5, ND2, UQCRH, and CYB) were verified in OC cells (SK-OV3, TOV-21G, and OVCAR-3) with qRT-PCR, and found that PKM, PDHB, IDH3A, IDH3B, ND5, ND2, and CYB were highly expressed in OC cells compared to normal cells [16].
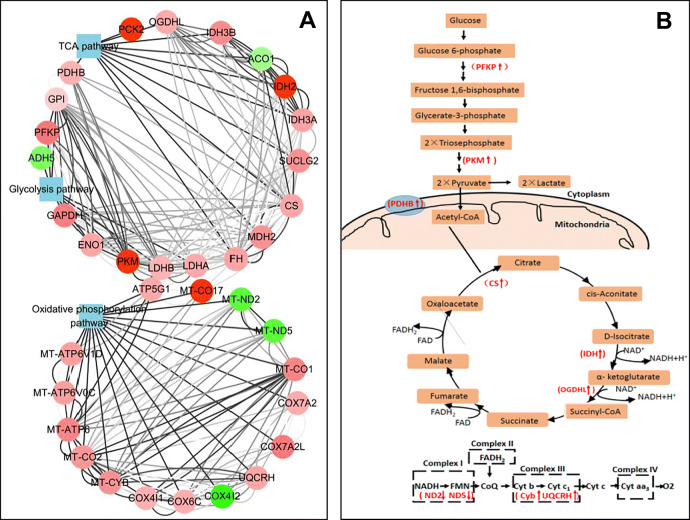


**Fig. 2** The changes of key proteins in oxidative phosphorylation, Krebs cycle, and glycolysis pathways. Reproduced from Li, Zhan, and Zhan (2018) [16], with permission from Elsevier, copyright 2018

Quantitative transcriptomics revealed that survival-related lncRNA SNHG3 regulated the key molecules (PFKM, PKM, PDHB, CS, IDH2, IDH3A, IDH3B, OGDHL, ND2, ND5, CYB, and UQCRH) in three energy metabolism pathways by their RNA-binding proteins (EIF4A3) to bind PKM in glycolysis, IDH2 in the Krebs cycle, and UQCRH in OXPHOS pathways, or by miRNA (hsa-miR-186-5p) to bind the 3’UTR of PDHB linking glycolysis with the Krebs cycle [16]. Additionally, SNHG3 was significantly upregulated in OC cells (SKOV3, TOV21G, and OVCAR3) relative to control cells (IOSE80), and SNHG3 was associated with drug sensitivity or multidrug resistance of tamoxifen in OCs. Moreover, inhibition of SNHG3 expression in OC cell (SKOV3) caused significant decreases of the key molecules (PFKM, PKM, PDHB, CS, IDH2, IDH3A, IDH3B, OGDHL, ND5, ND2, CYB and UQCRH) in three energy metabolism pathways at the levels of mRNAs and proteins [16].

**Cancer cells and stroma cells (especially CAFs) have metabolic symbiosis**

Cancer cells induce oxidative stress of CAFs by secreting ROS to enhance aerobic glycolysis of CAFs. In turn, CAFs produced lots of nourishment to be ‘eaten’ up by cancer cells for producing ATP through the Krebs cycle and oxidative phosphorylation [10]. They are connected by a lactate shuttle (MCT-1 and MCT4) between cancer cells and CAFs [11], and MCT-1 and MCT-2 are significantly upregulated at the mRNA level in three OC cells [16]. The nanomaterial-siRNAs of SNHG3 might be promising for OC patients to block the abnormal energy metabolism [16].

**Conclusions and expert recommendation**

Energy metabolism heterogeneity is a hallmark in OC. The changed key molecules and regulators in three energy metabolism pathways are the precious resource to help discover effective therapeutic drugs and molecule-pattern biomarkers for PPPM in OCs and patient individualized stratification. We recommend the use of multiomics to study energy metabolism abnormalities, and reveal the complete changes of molecules in three energy metabolism pathways and their regulators. Energy metabolism pathway network-based molecule-pattern biomarkers and therapeutic targets have more important scientific merits in OC.

**Acknowledgments**

The authors acknowledge the financial supports from the Shandong First Medical University Talent Introduction Funds (to X.Z.), and the Hunan Provincial Hundred Talent Plan (to X.Z.).

**References**

1. Li N, Zhan X. Signaling pathway network alterations in human ovarian cancers identified with quantitative mitochondrial proteomics. EPMA J. 2019;10(2):153–172. 10.1007/s13167-019-00170-5.
2. Lu M, Zhan X. The crucial role of multiomic approach in cancer research and clinically relevant outcomes. EPMA J. 2018;9(1):77–102. 10.1007/s13167-018-0128-8.
3. Cheng T, Zhan X. Pattern recognition for predictive, preventive, and personalized medicine in cancer. EPMA J. 2017;8:51–60. 10.1007/s13167-017-0083-9.
4. Golubnjitschaja O, Costigliola V, EPMA. General report & recommendations in predictive, preventive and personalized medicine 2012: White Paper of the European Association for Predictive, Preventive and Personalised Medicine. EPMA J. 2012;3:14. 10.1186/1878-5085-3-14.
5. Qian S, Golubnitschaja O, Zhan X. Chronic inflammation: key player and biomarker-set to predict and prevent cancer development and progression based on individualized patient profiles. EPMA J. 2019;10(4):365–381. 10.1007/s13167-019-00194-x.
6. Golubnitschaja O, Polivka J Jr., Yeghiazaryan K, Berliner L. Liquid biopsy and multiparametric analysis in management of liver malignancies: new concepts of the patient stratification and prognostic approach. EPMA J. 2018;3:271–285. 10.1007/s13167-018-0146-6.
7. Zhan X, Desiderio DM. Editorial: Molecular network study of pituitary adenomas. Front Endocrinol. 2020;11:00026. 10.3389/fendo.2020.00026.
8. Warburg O. On the origin of cancer cells. Science. 1956;123:309–314. 10.1126/science.123.3191.309.
9. Pavlides S, Whitaker-Menezes D, Castello-Cros R, Flomenberg N, Witkiewicz AK, Frank PG, et al. The reverse Warburg effect: aerobic glycolysis in cancer associated fibroblasts and the tumor stroma. Cell Cycle. 2009; 8:3984–4001. 10.4161/cc.8.23.10238.
10. Lee M, Yoon JH. Metabolic interplay between glycolysis and mitochondrial oxidation: The reverse Warburg effect and its therapeutic implication. World J Biol Chem. 2015; 6: 148–61. 10.4331/wjbc.v6.i3.148.
11. Whitaker-Menezes D, Martinez-Outschoorn UE, Lin Z, Ertel A, Flomenberg N, Witkiewicz AK, et al. Evidence for a stromal-epithelial “lactate shuttle” in human tumors: MCT4 is a marker of oxidative stress in cancer-associated fibroblasts. Cell Cycle. 2011;10:1772–83. 10.4161/cc.10.11.15659.
12. Vyas S, Zaganjor E, Haigis MC. Mitochondria and cancer. Cell. 2016; 166:555–66. 10.1016/j.cell.2016.07.002.
13. Mintz HA, Yawn DH, Safer B, Bresnick E, Liebelt AG, Blailock ZR, et al. Morphological and biochemical studies of isolated mitochondria from fetal, neonatal, and adult liver and from neoplastic tissues. J Cell Biol. 1967;34:513–23. 10.1083/jcb.34.2.513.
14. Chen M, Huang H, He H, Ying W, Liu X, Dai Z, et al. Quantitative proteomic analysis of mitochondria from human ovarian cancer cells and their paclitaxel-resistant sublines. Cancer Sci. 2015;106:1075–83. 10.1111/cas.12710.
15. Huarte M. The emerging role of lncRNAs in cancer. Nat Med. 2015;21:1253–61. 10.1038/nm.3981.
16. Li N, Zhan XH, Zhan X. The lncRNA SNHG3 regulates energy metabolism of ovarian cancer by an analysis of mitochondrial proteomes. Gynecol Oncol. 2018;150(2):343–354. 10.1016/j.ygyno.2018.06.013.
17. Li N, Li H, Cao L, Zhan X. Quantitative analysis of the mitochondrial proteome in human ovarian carcinomas. Endocrine-Related Cancer. 2018; 25(10): 909–931. 10.1530/ERC-18-0243.
18. Li N, Zhan X, Zhan X. Energy Metabolism heterogeneity-based molecular biomarkers for ovarian cancer. In: Molecular medicine. Nalbantoglu S, Amri H (eds.). London: InTech; 2018. 10.5772/intechopen.80622.
19. Zhan X, Zhou T, Li N, Li H. The differentially mitochondrial proteomic dataset in human ovarian cancer relative to control tissues. Data In Brief. 2018;20:459–462. 10.1016/j.dib.2018.08.028.
20. Wang LN, Tong SW, Hu HD, Ye F, Li SL, Ren H, et al. Quantitative proteome analysis of ovarian cancer tissues using a iTRAQ approach. J Cell Biochem. 2012;113:3762–3772. 10.1002/jcb.24250.

**Systematic analyses reveal long non-coding RNA (PTAF)-mediated promotion of EMT and invasion-metastasis in serous ovarian cancer**

Liang H*

*Corresponding author: Department of Pharmacology (State-Province Key Laboratories of Biomedicine-Pharmaceutics of China, Key Laboratory of Cardiovascular Research, Ministry of Education), College of Pharmacy, Harbin Medical University, Harbin 150081, China

A deeper mechanistic understanding of epithelial-to-mesenchymal transition (EMT) regulation is needed to improve current anti-metastasis strategies in ovarian cancer (OvCa). Here, by systematically analyzing high-throughput gene expression profiles of both lncRNAs and protein-coding genes in OvCa samples with integrated epithelial (iE) subtype and integrated mesenchymal (iM) subtype labels, we identified a lncRNA-mediated competing endogenous RNA (ceRNA) regulatory network that affects the expression of many EMT-related protein-coding genes in mesenchymal OvCa. Using a combination of in vitro and in vivo studies, we provide evidence that the lncRNA PTAF-miR-25-SNAI2 axis controls EMT in OvCa. Our results revealed that up-regulated PTAF induced elevated SNAI2 expression by competitively binding to miR-25, which in turn promoted OvCa cell EMT and invasion. Moreover, we found that silencing of PTAF inhibited tumor progression and metastasis in an orthotopic mouse model of OvCa. We then observed a significant correlation between PTAF expression and EMT markers in OvCa patients. These findings suggest that the lncRNA PTAF, a mediator of TGF-β signaling, could predispose OvCa patients to metastases and may serve as a potential target for anti-metastatic therapies for mesenchymal OvCa patients.

**Liver carcinomas: the emergent field for the paradigm shift from reactive to predictive, preventive and personalised medicine**

Barilo A^1^, Golubnitschaja O*^2^

^1^Center of Molecular Biotechnology, Friedrich-Wilhelms-University of Bonn, Germany

^2^Predictive, Preventive and Personalised (3P) Medicine, Department of Radiation Oncology, University Hospital Bonn, Rheinische Friedrich-Wilhelms-Universität Bonn, Germany

*Corresponding author: Prof. Dr. Olga Golubnitschaja, Predictive, Preventive and Personalised (3P) Medicine, Department of Radiation Oncology, Friedrich-Wilhelms-University Bonn, Venusberg-Campus 1, 53127 Bonn, Germany; e.mail: Olga.Golubnitschaja@ukbonn.de

**Keywords:** predictive preventive personalised medicine, liver carcinoma, malignancy, patient stratification, hepatocellular carcinoma, solid tumours, metastasis, multi-omics, biomarker patterns, in-depth diagnostics, survival, therapy prognosis, liquid biopsy, modifiable risk factors, risk assessment, multi-parametric analysis

**Highly heterogeneous origin of LC as a challenge for diagnostics and treatment**

Liver carcinomas (LC) with highly heterogeneous origin are one of the most widespread and severe cancer forms worldwide with particularly poor prognosis.

Primary liver cancer includes hepatocellular carcinoma (HCC) (75%–85%), intrahepatic cholangiocarcinoma (10%–15%) and other rarer cases. According to the global cancer statistics, in addition to 782,000 deaths from liver cancer, the estimation of 841,000 occurred in 2018 [1]. Besides the primary liver tumours, almost any malignancy could metastasize to the liver. To this end, the liver is one of the predominant sites for breast cancer metastases contributing 21% to all the cases. A recent paper analysed the data from 224,449 patients from the USA; 11,997 (5.3%) patients had the metastatic disease, and 3276 (1.5%) patients were diagnosed with liver metastases [2]. The liver is also a site for colorectal cancer metastases occurring in 15% of all cases [3]. Liver metastases are more common than primary tumours [3]. A recent study looked at the patient treatment across the regions and showed that more than 80% of patients from the Asia Pacific, Japan, Europe, and the USA were males with median age over 54 years old [4].

**Modifiable risk factors play a key role in LC**

Noteworthy for the absolute majority of LC cases, modifiable risk factors play a decisive role in the pathology manifestation and progression and could be prevented by targeted measures that should be well considered for educational programs in the population. Risk factors include viral infections such as hepatitis B and hepatitis C (80% of cases) [3], hemochromatosis, Diabetes mellitus (8%), abnormal alcohol consumption (7–50%), unbalanced diet and smoking (25%), amongst others [5]. Educational programmes could promote health literacy and more effective prevention in the population.

**LC is frequently diagnosed at advanced stages**

Owing to the current lack of effective strategies for LC prevention and targeted screening programs, the disease is frequently diagnosed at advanced stages followed by palliative treatment with 5-year survival rates of 0–10%. In contrast, early diagnosed patients have been demonstrated as cured with significantly better success; however currently still with 50% reoccurrence rate and about 25% survival within 5 years.

**LC management requires a paradigm change from reactive to predictive, preventive and personalised medicine**

To this end, the main problem is an extremely high heterogeneity of the LC patient cohort that requires a paradigm change from cost-ineffective reactive medical services to an advanced predictive, preventive and personalised medicine approach based on the targeted preventive measures utilising educational programmes, innovative population screening, early multi-level diagnostics, improved patient stratification and treatment algorithms tailored to the person.

**Application of liquid biopsy, multi-omics and multiparametric analysis is crucial to improve individual survival in LC**

A recent study demonstrated that application of liquid biopsy, multi-omics and multiparametric analysis is crucial for the patient stratification and accurate consideration of therapy modalities that depends on the individualised patient profile including sub-cellular imaging by comet assay, expression patterns of specific biomarkers involved in tumour development and progression and activity of tissue-remodelling enzymes [6].

**Outlook**

Follow-up large-scale multi-centre research is essential to validate the preliminary conclusions of the recently published pilot study [6], in order to validate the presented biomarker-panels and consequently optimise individual patient outcomes.

**References**

1. Bray F, Ferlay J, Soerjomataram I, et al. Global cancer statistics 2018: GLOBOCAN estimates of incidence and mortality worldwide for 36 cancers in 185 countries. CA Cancer J Clin 2018;68:394–424. 10.3322/caac.21492
2. Zhao H, Gong Y, Ye F, et al. Incidence and prognostic factors of patients with synchronous liver metastases upon initial diagnosis of breast cancer: a population-based study. Cancer Manag Res. 2018;10:5937–5950. 10.2147/cmar.s178395
3. Golubnitschaja O, Sridhar K. Liver metastatic disease: new concepts and biomarker panels to improve individual outcomes. Clin Exp Metastasis. 2016;33:743–755. 10.1007/s10585-016-9816-8
4. Kudo M, Lencioni R, Marrero J, et al. Regional differences in sorafenib-treated patients with hepatocellular carcinoma: GIDEON observational study. Liver Int. 2016;36:1196–1205. 10.1111/liv.13096
5. Ananthakrishnan A, Gogineni V, Saeian K. Epidemiology of Primary and Secondary Liver Cancers. Semin Intervent Radiol. 2016;23:047–063. 10.1055/s-2006-939841
6. Golubnitschaja O, Polivka J Jr., Yeghiazaryan K, Berliner L. Liquid biopsy and multiparametric analysis in management of liver malignancies: New concepts of the patient stratification and prognostic approach. EPMA J. 2018;9:271–285. 10.1007/s13167-018-0146-6

**In-depth multiomic analysis and patient stratification to advance the management of liver malignancies according to the principles of 3P Medicine**

Goldstein E^1,2^, Golubnitschaja O*^3^

^1^Bar-Ilan University, Ramat-Gan, Israel

^2^State NRW-Israel program, Friedrich-Wilhels-University Bonn, Germany

^3^Predictive, Preventive and Personalised (3P) Medicine, Department of Radiation Oncology, University Hospital Bonn, Friedrich-Wilhelms-University Bonn, Germany

*Corresponding author: Prof. Dr. Olga Golubnitschaja, Predictive, Preventive and Personalised (3P) Medicine, Department of Radiation Oncology, Friedrich-Wilhelms-University Bonn, Venusberg-Campus 1, 53127 Bonn, Germany; e.mail: Olga.Golubnitschaja@ukbonn.de

**Keywords:** predictive preventive personalised medicine, liver malignancy, patient stratification, hepatocellular carcinoma, colorectal cancer, metastasis, multi-omics, biomarker patterns, in-depth diagnostics, survival, therapy prediction, prognosis, metalloproteinase, comet assay, calgranulin A, catalase, superoxide-dismutase 2, profilin, Rho A, thioredoxin

**Background**

Patients with primary and metastatic liver malignancies represent a highly heterogeneous patient pool characterised by some of the shortest life expectancies amongst oncology patients. Computational tools utilising multi-parametric analysis are instrumental for comprehensive individualised patient profiling, predictive diagnostics and prognosis with a high potential for clinical implementation. This approach may significantly contribute to the paradigm change from reactive to predictive, preventive and personalised medicine benefiting patients predisposed to and/or diagnosed with the liver malignancies as well as healthcare systems at large [1,2]. A prospective pilot research project performed at the UKB, University of Bonn has identified potential multi-omic biomarker patterns for predictive stratification of liver cancer patients who underwent SIRT (selective internal radio-therapy) versus TACE (trans-arterial chemoembolisation) treatment [3,4]. It was concluded that the treatment option is decisive for the quality of individual outcomes. Consequently, the treatment selection should be tailored to the individualised patient profile.

**Working hypothesis, materials and methods**

In the current project we hypothesised that a comprehensive multi-omic analysis may reveal differences in relevance of individual biomarkers for overall survival, depending on the original tumour diagnosed. Contextually, the patient stratification was performed as following:

- colorectal carcinoma (CRC) patients with liver metastasis and overall survival shorter than 6 months (18 patients)
- CRC patients with liver metastasis and overall survival longer than 24 months (8 patients)
- hepatocellular carcinoma (HCC) patients demonstrating the overall survival shorter than 6 months (11 patients)
- HCC patients demonstrating the overall survival longer than 24 months (9 patients).

All CRC patients (26) underwent TACE, whereas HCC underwent either TACE (11) or SIRT (9).

The multi-omic biomarker patterns used in this study were proven earlier as being generally relevant for prediction of the overall survival in the patients’ pool with liver malignancies [3, 4], if measured before the therapy applied:

- gelatinase activities of metalloproteinases (MMP) 2 and 9 measured in blood serum
- expression patterns of calgranulin A, catalase, superoxide-dismutase (SOD) 2, profiling 1, RhoA, and thioredoxin (Trx) – all measured at the protein level in circulating leucocytes
- sub-cellular imaging by “comet assay” DNA analysis in circulating leucocytes.

The molecular pathways this biomarker-panel is involved in and their relevance for liver malignancies and therapy success were detailed by Golubnitschaja et al. [4].

**Results** Below presented figures (see summarising Figs. 1–3) demonstrate the distribution of biomarker-patterns correlated with the primary diagnosis (CRC versus HCC) and overall survival of the stratified patient groups marked as short (< 6 months) and long (> 24 months). All the values were normalised before plotting; consequently all the biomarkers are scaled within the same range between 0 and 1.
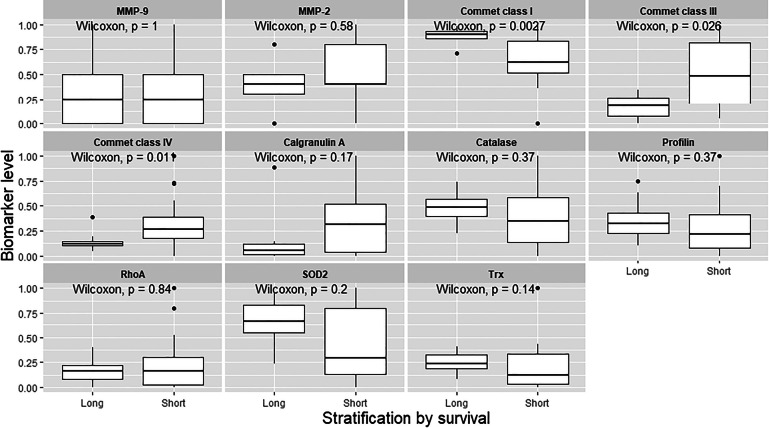


**Fig. 1** Distribution of the biomarker-patterns in CRC patients with liver metastases stratified by short and long survival: statistically significant differences are demonstrated for patterns with an increased level of comet class I (intact DNA) and decreased level of class IV (apoptotic DNA) in the group with the long survival; although being statistically non-significant the tendencies to decreased MMP-2 activity, decreased level of comet class III (strongly damaged DNA), decreased expression levels of calgranulin A on the one hand, but on the other hand, increased expression levels of catalase, SOD-2 for the long survival are demonstrated.
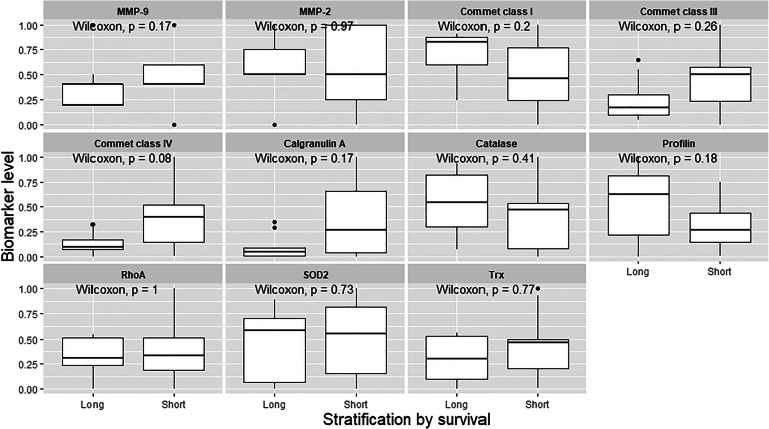


**Fig. 2** Distribution of the biomarker-patterns in HCC patients stratified by short and long survival: although being statistically non-significant, a tendency to decreased MMP-9 activity, decreased level of comet class III (strongly damaged DNA) and class IV (apoptotic DNA), decreased expression levels of calgranulin A on the one hand, but on the other hand increased level of comet class I (intact DNA), increased expression levels of catalase and profilin for a long survival are demonstrated


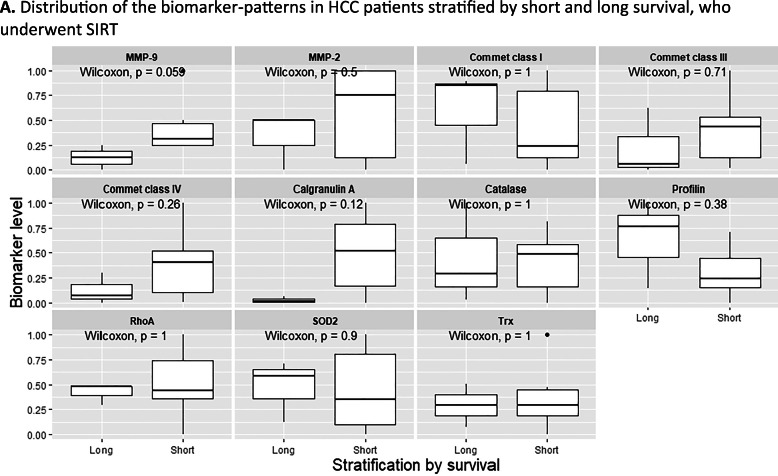

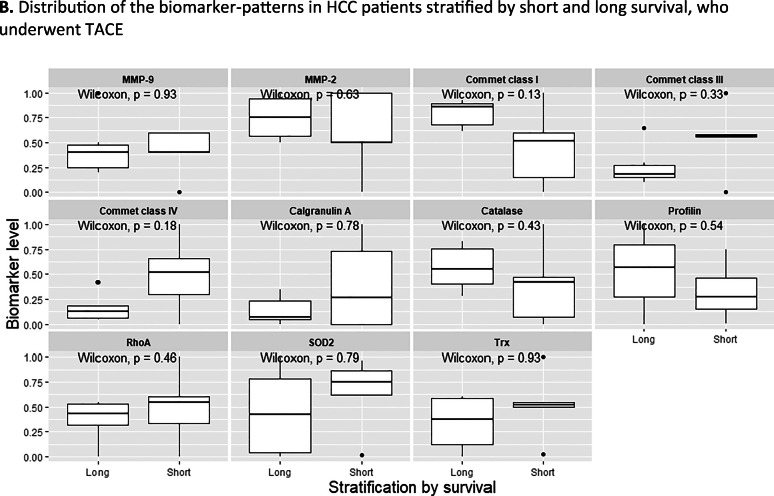


**Fig. 3** Distribution of the biomarker-patterns in HCC patients stratified by short and long survival as well as therapy approach (**a**) SIRT versus (**b**). TACE: although being statistically non-significant, differences in the pattern distribution are evident for both long versus short survival and SIRT versus TACE in HCC patients. This can be concluded as following: the comet patterns may be instrumental for the survival prediction under the TACE condition, whereas the MMP-9 patterns seem to be more relevant for the survival prediction under the SIRT condition

**Conclusions and expected impacts** The pilot study demonstrated evident differences in the biomarker-patterns correlated with the short versus long survival, primary tumour and the therapy type applied. Besides in-depth understanding of the molecular mechanisms which underlie the treatment approaches used and, consequently, individual outcomes, better justified selection of the treatment algorithms is expected by the above-proposed patient stratification considering the primary diagnosed tumour. Treatments tailored to the personalised patient profile may lead to significantly improved individual outcomes benefitting the affected patients and the overall disease management. Multi-omic pattern recognition, individualised profiling and detailed patient stratification are instrumental for the advanced concepts of predictive, preventive and personalised medicine in the management of liver malignancies [4–6].

**Acknowledgements**

The study stay of Mr. E. Goldstein at the 3PM unit (Head - Prof. Dr. Olga Golubnitschaja), University Hospital Bonn, Rheinische Friedrich-Wilhelms-Universität Bonn, Germany was supported by the governmental NRW(Germany)/Israeli programme for academic exchange, fellowship 2019. Mr. E. Goldstein was awarded by the European Association for Predictive, Preventive and Personalised Medicine, EPMA Brussels, for the best scientific presentation at the Workshop of Young Professionals in PPPM, EPMA World Congress 2019, September 19–22 in Pilsen, Czech Republic.

**References**

1. Golubnitschaja O, Baban B, Boniolo G, Wang W, Bubnov R, Kapalla M, Krapfenbauer K, Mozaffari M, Costigliola V. Medicine in the early twenty-first century: paradigm and anticipation – EPMA position paper 2016. EPMA J 2016;7:23, 10.1186/s13167-016-0072-4.
2. Patient-centered care: making the modern hospital truly modern. In: Latifi R, editior. The modern hospital: patients centered, disease based, research oriented, technology driven, Cham: Springer; 2018.
3. Golubnitschaja O, Yeghiazaryan K, Stricker H, Trog D, Schild HH, Berliner L. Patients with hepatic breast cancer metastases demonstrate highly specific profiles of matrix metalloproteinases MMP-2 and MMP-9 after SIRT treatment as compared to other primary and secondary liver tumours. BMC Cancer 2016;16(1):357. 10.1186/s12885-016-2382-2.
4. Golubnitschaja O, Polivka J Jr., Yeghiazaryan K, Berliner L. Liquid biopsy and multiparametric analysis in management of liver malignancies: New concepts of the patient stratification and prognostic approach. EPMA J. 2018;9(3):271–285. 10.1007/s13167-018-0146-6.
5. Cheng T, Zhan X. Pattern recognition for predictive, preventive, and personalized medicine in cancer. EPMA J. 2017;8(1):51–60. 10.1007/s13167-017-0083-9.
6. Lu M, Zhan X. The crucial role of multiomic approach in cancer research and clinically relevant outcomes. EPMA J. 2018;9(1):77–102. 10.1007/s13167-018-0128-8.

**Prostate cancer: decreasing age, increasing incidence of metastatic disease and high CTC potential prompts the paradigm shift from reactive to predictive, preventive and personalised medicine in disease management**

Golubnitschaja O*^1^, Raj Dahal A^2^

^1^Predictive, Preventive and Personalised (3P) Medicine, Department of Radiation Oncology, University Hospital Bonn, Friedrich-Wilhelms-University Bonn, Germany

^2^Center of Molecular Biotechnology, Rheinische Friedrich-Wilhelms-Universität Bonn, Germany

*Corresponding author: Prof. Dr. Olga Golubnitschaja, Predictive, Preventive and Personalised (3P) Medicine, Department of Radiation Oncology, University Hospital Bonn, Rheinische Friedrich-Wilhelms-Universität Bonn, Venusberg-Campus 1, 53127 Bonn, Germany; e.mail: Olga.Golubnitschaja@ukbonn.de

**Keywords:** predictive preventive personalised medicine, prostate cancer (PCa), malignancy, incidence, mortality, disease manifestation, circulating tumour cells (CTC), young age, patient stratification, aggressive metastatic disease, multi-omics, biomarker patterns, survival, liquid biopsy, modifiable risk factors, risk assessment, multi-parametric analysis

**PCa incidence and related mortality in the global context of oncologic diseases**

Current statistics demonstrate PCa among the most frequent types of cancer worldwide [1], see Fig. 1. Although on average PCa is ranked as the fourth most frequent oncologic disease, the prevalence and incidence rates differ significantly from country to country.
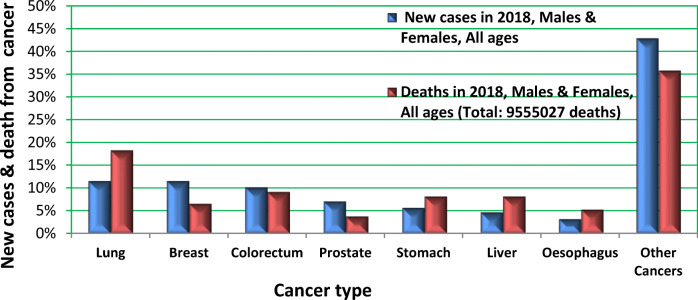


**Fig. 1** Global statistics of oncologic diseases released by GLOBOCAN in 2018 [1]: In total, 18,078,957 new cases and 9,555,027 related deaths were registered for males and females of all ages

**Breast, prostate and lung cancers spread the highest amount of CTC**

Accumulated evidence demonstrates high density of circulating tumour cells spread by primary tumours as a reliable indicator for developing metastatic disease [2]. In particular, three types of malignancies have been demonstrated to spread the highest amount of CTC in blood, namely breast, prostate and lung. Consequently, liquid biopsy tests for enumeration and molecular characterisation of CTC are highly recommended for these patient cohorts to predict and prevent metastatic disease [3–5].

**Incidence of metastatic PCa is increasing**

As reported by several groups worldwide, incidence of metastatic PCa is rapidly increasing in populations. Although the epidemiology of the metastatic PCa is not completely understood yet, and the reasons for the rapidly increasing incidence remain unclear, multi-factorial risks have been proposed [6].

**PCa incidence and related mortality is increasing in older adolescents and young adults**

Since 1990, PCa has increased in most countries in older adolescent and young adult men between ages 15 and 40 years—globally at a steady rate averaging 2% per year since 1990 (*P* < .01) [7]. A prominent example comes from UK, where national records show that although PCa incidence is permanently increasing in almost all age groups since 1990s, the increase is disproportionally high for men aged 25–49 years by over 400%, compared to 285% for men aged 50–59 year, 142% for men aged 60–69 years, 42% and 23% for men aged 70–79 and 80+ years, respectively [8]. Several European countries and regions report alarming statistics for PCa cases recorded in adolescence (15–19 years of age) and young adults aged 20–24 years and 25–29 years as demonstrated in Fig. 2. Similarly to the increasing incidence of metastatic PCa, the reasons for the increasing PCa incidence in young populations is considered to carry a multi-factorial character [7].
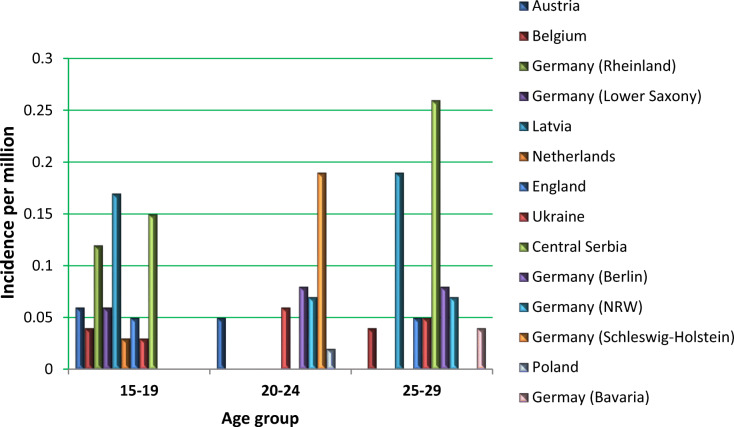


**Fig. 2** PCa as a burden in young European populations: Incidence rates per million of inhabitants recoded in years 2004–2010 [9]

**Conclusions and outlook**

In summary, PCa belongs to the cancer types with highest incidence recorded worldwide. The PCa incidence is annually increasing. Moreover, the incidence of metastasing PCa is increasing as well. Further, alarming statistics have been registered for adolescents and young adults aged 15–40 years with a tendency to particular rapidly increasing incidence rates. Multi-factorial risks for these trends have been proposed. One of the evident deficits is the reactive character of medical services provided, which does not meet the needs of the population and in particular of young people [10]. Consequently, the paradigm shift is essential to provide cost-effective healthcare utilising multi-professional expertise for predictive diagnosis, targeted prevention and treatments tailored to the person—the concepts of 3P medicine presented by the EPMA [11]. Concretely, innovative screening programmes are essential to identify persons in suboptimal health conditions (such as suffering from systemic hypoxic conditions and low grade inflammation) followed by the targeted primary prevention [12–14]. Secondary prevention of PCa metastatic disease is based on the liquid biopsy tests for CTC enumeration and their molecular characterisation. Comprehensive individualised patient profiling is to be used for in-depth diagnostics and personalisation of medical services [14].

**References**

1. GLOBOCAN 2018, https://www.uicc.org/news/new-global-cancer-data-globocan-2018, last access on February 21st 2020
2. Allard WJ, Matera J, Miller MC, Repollet M, Connelly MC, Rao C, et al. Tumor cells circulate in the peripheral blood of all major carcinomas but not in healthy subjects or patients with nonmalignant diseases. Clin Cancer Res. 2004;10(20):6897–904. 10.1158/1078-0432.CCR-04-0378
3. Hench IB, Cathomas R, Costa L, Fischer N, Gillessen S, Hench J, et al. Analysis of AR/ARV7 Expression in Isolated Circulating Tumor Cells of Patients with Metastatic Castration-Resistant Prostate Cancer (SAKK 08/14 IMPROVE Trial). Cancers 2019. 10.3390/cancers11081099;
4. Buscail E, Chiche L, Laurent C, Vendrely V, Denost Q, Denis J, et al. Tumor-proximal liquid biopsy to improve diagnostic and prognostic performances of circulating tumor cells. Mol Oncol. 2019;13(9):1811–1826. 10.1002/1878-0261.12534;
5. Stefanovic S, Deutsch TM, Wirtz R, Hartkopf A, Sinn P, Schuetz F, et al. Molecular Subtype Conversion between Primary and Metastatic Breast Cancer Corresponding to the Dynamics of Apoptotic and Intact Circulating Tumor Cells. Cancers 2019;11(3). pii: E342. 10.3390/cancers11030342.
6. Yang DX, Makarov DV, Gross CP, Yu JB. Geographic-Level Association of Contemporary Changes in Localized and Metastatic Prostate Cancer Incidence in the Era of Decreasing PSA Screening. Cancer Control 2020;27(1):1073274820902267. 10.1177/1073274820902267].
7. Bleyer A, Spreafico F, Barr R. Prostate cancer in young men: An emerging young adult and older adolescent challenge. Cancer 2020;126(1):46–57. 10.1002/cncr.32498
8. Cancer Research Uk, https://www.cancerresearchuk.org/health-professional/cancer-statistics/statistics-by-cancer-type/prostate-cancer/incidence#heading-Two, last access on February 21st 2020
9. European Cancer Information System, https://ecis.jrc.ec.europa.eu/explorer.php, last access on February 21st 2020
10. Golubnitschaja O (Ed.). Flammer syndrome – from phenotype to associated pathologies, prediction, prevention and personalization, vol. 11. Cham: Springer; 2019. 10.1007/978-3-030-13550-8
11. Golubnitschaja O, Baban B, Boniolo G, Wang W, Bubnov R, Kapalla M, et al. Medicine in the early twenty-first century: paradigm and anticipation – EPMA position paper 2016. EPMA J. 2016;7:23. 10.1186/s13167-016-0072-4.
12. Patient-centered care: making the modern hospital truly modern. In: Latifi R, editior. The modern hospital: patients centered, disease based, research oriented, technology driven, Cham: Springer; 2018.
13. Qian S, Golubnitschaja S, Zhan X. Chronic inflammation: key player and biomarker-set to predict and prevent cancer development and progression based on individualized patient profiles. EPMA J. 10(4):365–381. 10.1007/s13167-019-00194-x.
14. Gerner C, Costigliola V, Golubnitschaja O. Multiomic patterns in body fluids: technological challenge with a great potential to implement the advanced paradigm of 3P medicine. Mass Spectrom Rev. 2019. 10.1002/mas.

**New Potential Molecular Biomarkers in Prostate Cancer Diagnostics**

Rezackova H, Windrichova J, Topolcan O, Kucera R*

Department of Immunochemistry Diagnostics, University Hospital in Pilsen, Medical Faculty in Pilsen, Charles University, Czech Republic

*Corresponding author: Radek Kucera, Ph.D., Associate Professor, University Hospital Pilsen, CZ; e.mail: kucerar@fnplzen.cz, ORCID: 0000-0002-2739-2302

**Keywords**: Preventive predictive personalized medicine (PPPM), prostate cancer, microRNA, diagnostic biomarkers, predictive biomarkers, prognostic biomarkers, biomarker, liquid biopsy, circulating level, transcriptome

**Introduction**

Prostate cancer (PCa) is the most commonly diagnosed malignancy and the fifth leading cause of cancer-related death among males in Western countries. Worldwide 1.4 million new cases of prostate cancer occurred in 2016 [1]. The rising burden of prostate cancer requires the active role of PPPM in predicting PCa more precisely and providing a reliable risk stratification of patients. This aim could be satisfied by novel diagnostic and prognostic tools.

Nowadays, PCa is diagnosed by a histological inspection of prostate needle biopsies, generally indicated by an elevated serum prostate specific antigen (PSA) test and/or a suspect digital rectal examination (DRE) [2, 3]. PSA has low specificity for PCa while biopsies have high false negative rates. The introduction of novel biomarkers will help prevent overtreatment or delayed intervention [4, 5]. New molecular biomarkers could furthermore improve the accuracy of PCa detection and contribute to predictive diagnostics, patient stratification and the subsequent design of individualized treatment [6, 7].

**Prostate cancer, novel biomarkers**

An EPMA position paper reviewed protein candidates for novel prostate biomarkers: members of the Cip/Kip family, p21 and 27, are potential prognostic factors while upregulation of the HER-2/neu protein bFGF VEGF has been proven to be associated with adverse outcomes and the over expression of bcl-2 is linked with acquired resistance [8]. Circulating cell free molecular biomarkers could be a solution to the demand for minimally invasive diagnostic tools made by EPMA [9, 10]. Based on our own work, we would emphasize that regulatory non-coding RNAs represent an interesting target for the discovery of biomarkers. This includes small ncRNAs, including microRNAs (miRNAs), Piwi-interacting RNAs (piRNAs), medium ncRNAs, including small nuclear RNA (snRNA), small nucleolar RNA (snoRNA), and long non-coding RNA (lncRNAs). The LncRNA group is now represented by the highly evaluated Prostate Cancer Antigen 3 (PCA3; originally referred to as DD3), which has been shown to be a prostate-specific molecule upregulated in PCa and androgen-regulator. It is said to be the most specific biomarker of PCa identified to date [3]. This biomarker could serve as an example of a successful molecular biomarker pipeline discovery for personlized medicine.

**MiRNAs and LncRNAs as Candidate Biomarkers for Prostate Cancer**

MicroRNA-based diagnostics show great promise in the field of early cancer detection and secondary prevention [11]. Each miRNA can target several hundred mRNAs, thus playing a critical role in multiple physiological processes [12, 13]. Deregulated miRNA expression has been associated with tumor progression in several cancer types. Circulating miRNAs which can be isolated from the body fluids of cancer patients offer many advantages as biomarkers: their high pH, resistance to ribonuclease degradation, incubation at room temperature for 24 h, stability after frequent freeze thawing, and relatively simple quantification by PCR-based techniques [13].

There are many miRNA candidates—see Table 1 for a review - that can be used to predict PCa (miR-21, miR-200b, miR-429), as a prognostic marker (miR-141, miR-221, miR-375) or as a diagnostic marker (miR-106a, miR-200c). The LncRNAs could be represented by three promising biomarkers for prostate cancer: Metastasis associated lung adenocarcinoma transcript 1 (MALAT 1), Second Chromosome Locus Associated with Prostate 1 (SChLAP 1 or known as LINC00913) or FR0348383 [14].

**Table 1** New candidates for predictive and prognostic biomarkers

**Table:** 

miRNA candidates	Source	Purpose	References
hsa-mir-21-5p	serum, plasma, tissue	predictive, diagnostic, prognostic	[2, 11–18]
hsa-mir-25-3p	tissue	diagnostic	[10, 14–16, 19]
hsa-mir-32-5p	tissue	diagnostic	[10, 14–16]
hsa-miR-93-5p	serum, tissue	diagnostic	[10, 14–16]
hsa-miR-141-3p	serum, tissue	diagnostic, prognostic	[2, 13, 15–17]
hsa-mir-143-3p	serum	diagnostic	[17]
hsa-miR-155-5p	serum	diagnostic	[17]
hsa-mir-375-3p	serum, plasma, tissue	diagnostic, prognostic	[2, 13–17]
hsa-mir-429-3p	serum, plasma, tissue	predictive, prognostic,	[2, 15]
hsa-mir-451a-5p	serum	diagnostic, prognostic	[16]
hsa-mir-106a-5p	serum, plasma, tissue	diagnostic, prognostic	[2, 15, 16, 20]
hsa-mir-145-5p	plasma, tissue	diagnostic	[13, 16, 17]
hsa-mir-148a-3p	tissue	diagnostic	[15]
hsa-mir-15b-5p	tissue	diagnostic	[15]
hsa-mir-182-5p	tissue	diagnostic	[15, 16, 18]
hsa-mir-200b-3p	serum, plasma	predictive, diagnostic, prognostic	[2, 13, 15, 16]
hsa-mir-200c-3p	serum, plasma	diagnostic, prognostic	[2, 13, 15]
hsa-mir-20a-5p	serum, plasma, tissue	prognostic	[2, 15, 16, 20]
hsa-mir-221-3p	serum, plasma, tissue	diagnostic, prognostic	[2, 13, 15–17]

**Expert recommendations and a PPPM relevant outlook:**

- Wide-ranging and precise studies have to be conducted in order to select candidates and clinically validate miRNA, or their combinations, as biomarkers before we are able to use them in non-invasive methodology for PCa diagnostics and treatment management.
- Patients will benefit from novel validation studies directly focused on developing a powerful diagnostic tool based on circulating molecular biomarkers for patient profiling and stratification. This will lead to predictive and evidence-based treatment, individualized in order to reduce the use of invasive techniques such as biopsies or radical surgical treatment, resulting in a higher quality of life for patients and greater cost-effectiveness.

**Acknowledgements** Supported by Ministry of Health, Czech Republic - conceptual development of research organization (Faculty Hospital in Pilsen - FNPl, 00669806).

**References**

1. Sharma R. The burden of prostate cancer is associated with human development index: evidence from 87 countries, 1990–2016. EPMA J.2019; 10.1007/s13167-019-00169-y.
2. Hoey C, Liu SK. Circulating blood miRNAs for prostate cancer risk stratification: miRroring the underlying tumor biology with liquid biopsies. Dovepress. 2019; 10.2147/RRU.S165625.
3. Das R, Feng FY, Selth LA. Long non-coding RNAs in prostate cancer: Biological and clinical implications. Mol Cell Endocrinol. 2019;480:142–152.
4. Qian S, Golubnitschaja O, Zhan X. Chronic inflammation: key player and biomarker-set to predict and prevent cancer development and progression based on individualized patient profiles. EPMA J. 2019; 10.1007/s13167-019-00194-x.
5. Li N, Zhan X. Identification of clinical trait-related lncRNA and mRNA biomarkers with weighted gene co-expression network analysis as useful tool for personalized medicine in ovarian cancer. EPMA J. 2019; 10.1007/s13167-019-00175-0.
6. Janssens JP, Schuster K, Voss A. Preventive, predictive, and personalized medicine for effective and affordable cancer care. EPMA J. 2018; 10.1007/s13167-018-0130-1.
7. Li N, Zhan X. Signaling pathway network alterations in human ovarian cancers identified with quantitative mitochondrial proteomics. EPMA J. 2019; 10.1007/s13167-019-00170-5.
8. Grech G, Zhan X, Yoo BC, Bubnov R, Hagan S, Danesi R, et al. EPMA position paper in cancer: current overview and future perspectives. EPMA J. 2015; 10.1186/s13167-015-0030-6.
9. Golubnitschaja O, Kinkorova J, Costigliola V. Predictive, Preventive and Personlized Medicine as the hardcore of ‘Horizon 2020’: EPMA position paper. EPMA J. 2014; 10.1186/1878-5085-5-6.
10. Lee JH, Yu SE, Kim KH, Yu MH, Jeong IH, Cho JY, et al. Individualized metabolic profiling stratifies pancreatic and biliary tract cancer: a useful tool for innovative screening programs and predictive strategies in healthcare. EPMA J. 2018; 10.1007/s13167-018-0147-5.
11. Fabris L, Ceder Y, Chinnaiyan AM, Jenster GW, Sorensen KD, Tomlins S, et al. The Potential of MicroRNAs as Prostate Cancer Biomarkers. Eur Urol. 2016; 10.1016/j.eururo.2015.12.054.
12. Massillo C, Dalton GN, Farré PL, De Luca P, De Siervi A. Implications of microRNA dysregulation in the development of prostate cancer. Reproduction. 2017;154:R81-R97.
13. Razdan A, de Souza P, Roberts TL. Role of MicroRNAs in Treatment Response in Prostate Cancer. Curr Cancer Drug Targets. 2018;18:929–944.
14. Lim MCJ, Baird A, Aird J, Greene J, Kapoor D, Gray SG, et al. RNAs as Candidate Diagnostic and Prognostic Markers of Prostate Cancer—From Cell Line Models to Liquid Biopsies. Diagnostics. 2018; 10.3390/diagnostics8030060.
15. Kojima S, Goto Y, Naya Y. The roles of microRNAs in the progression of castration-resistant prostate cancer. Reproduction. 2017; 10.1038/jhg.2016.69.
16. Kumar B, Lupold SE. MicroRNA expression and function in prostate cancer: a review of current knowledge and opportunities for discovery. Asian J Androl. 2016;18:559–567.
17. Bertoli G, Cava C, Castiglioni I. MicroRNAs as Biomarkers for Diagnosis, Prognosis and Theranostics in Prostate Cancer. Int J Mol Sci. 2016; 10.3390/ijms17030421.
18. Kristensen H, Thomsen AR, Haldrup C, Dyrskjøt L, Høyer S, Borre M, et al. Novel diagnostic and prognostic classifiers for prostate cancer identified by genome-wide microRNA profiling. Oncotarget. 2016; 10.18632/oncotarget.8953.
19. Fabris L, Ceder Y, Chinnaiyan AM, Jenster GW, Sorensen KD, Tomlins S, et al. The Potential of MicroRNAs as Prostate Cancer Biomarkers. Eur Urol. 2016; 10.1016/j.eururo.2015.12.054.
20. Hoey C, Ahmed M, Fotouhi Ghiam A, Vesprini D, Huang X, Commisso K, et al. Circulating miRNAs as non-invasive biomarkers to predict aggressive prostate cancer after radical prostatectomy. J Transl Med. 2019;17:173.

**The influence of different treatment methods used in prostate cancer on PSA response, with a special focus on radiation therapy**

Svoboda T*, Jindrich Finek J

Department of Oncology and Radiotherapy, University Hospital in Pilsen, Czech Republic

*Corresponding author: Tomas Svoboda, MD., Ph.D.; e.mail: svobodat@fnplzen.cz

**Keywords:** preventive, predictive, personalised medicine (PPPM), prognostic and predictive value, PSA-prostate specific antigen, prostate cancer, radiation therapy, radical prostatectomy, vaccination, immunotherapy, bone metastases, radioisotop labelled PSMA.

**Introduction**

According to the International Comparison Diagnostics, the treatment of prostate cancer in our country is of high quality. Despite high incidence, the latest results published in GLOBOCAN (citation) showed that the mortality of our patients is much lower, or similar, to other high income or high human development index countries [1]. PSA testing is performed by GPs and urologists in the early stages of the disease, as well as by medical and radiation oncologists later on during follow-ups after oncological treatment other than surgery or androgen deprivation.

PSA is a glycoprotein produced by healthy glandular cells and ducts as well as in prostate cancer in response to some prostate changes (e.g. benign hyperplasia, inflammation, cancer and post biopsy – it has a high prognostic, but lower predictive, value). PSA is also age dependent. Because of the disease’s steeply rising prevalence we started an early detection program aimed at primary prevention in patients who have been followed-up for other malignancies in the Czech Republic since April 2018. Our decision was intimately linked to the paradigm shift from disease care to a personalised and preventive attitude to cancer care, de facto this means the employment of new technologies and a multilevel diagnostic process [2].

**Current situation in the clinical practice**

In clinical practice we are well experienced with PSA testing in patients treated by “older” methods such as androgen deprivation, radical surgery (prostatectomy) or external beam radiation with known and expected PSA response. However, we remain unfamiliar with patient response to newer treatment modalities. We compared our own clinical data with results published in the literature describing studies that presented conditions that, for various reasons, are different from those in our department, or country.

Huge progress has been made; in radiation oncology. We have completely moved from simple 3D conformal techniques to 4D radiation in the past two decades owing to better equipment (enabling special techniques such as IMRT, arc therapy up to IGRT with daily positioning and verification), more sophisticated treatment planning systems (avoiding radiation of surrounding healthy tissues, using data from modern mpMRI or even PET/CT for contouring) and/or immobilisation devices [3]. Moreover, brachytherapy (source of radiation placed right inside the tumour) has advanced and we are also able to use the newest brachytherapy-like based treatments, such as radium (Ra^223^) in bone metastases or PSMA-617 using beta radiation of Lu^177^ or Y^90^ and alpha radiation of Bi^213^ or Ac^225^ isotopes, which show very promising results.

**Treatment methods overview and PSA following a radical approach**

There are two radical treatment methods used in localised prostate cancer; namely radical prostatectomy (surgery) and radiation therapy. In some cases they can be supplemented by an endocrine approach in the form of androgen deprivation (ADT) and, most importantly, they can be applied to advanced/recurrent disease, or metastatic setting in patients with distant (mainly bone) metastases chemotherapy. As far as newer androgen receptor targeted agents (ARTA) are concerned, we have specific methods of cancer vaccination or brachytherapy-based treatments by Radium^223^ isotope. Furthermore, prostate specific membrane antigen (PSMA) guided therapy is used today. Simply put, prostate cancer treatment modalities and possibilities have at least doubled within the past few years. It has a clear impact on the need for regular PSA testing to determine treatment response and progression time because PSA levels in some cases are not in correlation with the achieved response [4].

After radical prostatectomy (RP), a PSA decline to 0 ng/ml (maximal detection level < 0,2 ng/ml) should be reached within hours, or a few days. Higher levels are associated with the presence of residual disease in the prostate bed or distant metastases. A similar PSA course can be seen after radical radiation (RT); however, it is much slower (weeks or months because of tumour cell radiobiology). It could be faster after some types of brachytherapy, or maybe extreme hypo fractionated regimens, but this concept requires further investigation. In clinical practice we do not always reach an undetectable PSA level, but the nadir’s long-term stabilisation is also considered a positive treatment result. Unfortunately, where PSA levels are low, a minimal residual disease after RP or RT is not detectable by any other method. When androgen deprivation (incl. new androgen receptor blockade by ARTA) or chemotherapy is used, we expect PSA to decrease more slowly (weeks or months) again. The flare phenomenon of LH-RH analogues is also known to occur in the treatment of all stages of prostate cancer. A similar bounce phenomenon with a temporary PSA rise can be seen 1–2 years after brachytherapy.

**Other therapeutic possibilities**

Vaccination (e.g. allogenic GVAX, DNA or viral vectors-based methods) is another treatment option available nowadays. Many patients are included in studies with anti-CTLA-4 or check-point inhibitor immunotherapy (ipilimumab, nivolumab, atezolizumab). Autologeus vaccination by Sipuleucel-T has already been approved in the US (but not by the European EMA). We know treatment response cannot be measured by PSA testing in this case. Even after special and new RT methods such as Radium^223^ (Xofigo), we often see a good patient response despite a rise in PSA. This means some treatments used today are not necessarily associated with a PSA decrease. PSMA isotopes might present the same dilemma, but we are unfamiliar with this method. Preliminary data showed about a1–2/3 PSA decline (by ≥50% in 30–60% of Lu^177^ cases, by 60–70%, in ^131^I-labelled PSMA ligand and similarly by ^255^Ac-PSMA-617 even when complete response caused by the treatment of the primary tumour was achieved, affecting all the metastatic sites [5]. In other words, methods that work on a similar principle could have a different PSA response.

**Conclusions and PPPM-based expert recommendations**

The incidence and prevalence of prostate cancer is rising fast. Although there are some promising predictive diagnostic features such as slow tumour growth and lower metastatic potential—predominantly with bone involvement—enabling waiting and observing in some cases, huge tumour heterogeneity, a high mutation burden and different intracellular signal pathway activations are difficulties that lead to varied individual treatment response and diverse efficacy of therapeutic interventions [6]. PSA testing has an important role in disease staging determination in prostate cancer, as well as in personalising treatment in response to tumour relapse and distant dissemination. Although a vaccination or Radium^223^ can be very effective, we usually see some kind of PSA increase. It therefore seems necessary to look for other treatment response parameters such as quality of life or pain relief instead of measuring PSA levels. According to expert recommendations, PSA levels should be evaluated individually which is in agreement with the principles of personalised medicine [7].

**References**

1. Sharma R. The burden of prostate cancer is associated with human development index: evidence from 87 countries, 1990–2016. EPMA J. 2019; 10.1007/s13167-019-00169-y.
2. Golubnitschaja O, Baban B, Boniolo G, Wang W, Bubnov R, Kapalla M, et al. Medicine in the early twenty-first century: paradigm and anticipation - EPMA position paper 2016. EPMA J. 2016;7:23–23.
3. Molacek J, Treska V, Zeithaml J, Hollan I, Topolcan O, Pecen L, et al. Blood biomarker panel recommended for personalized prediction, prognosis, and prevention of complications associated with abdominal aortic aneurysm. EPMA J. 2019; 10.1007/s13167-019-00173-2.
4. Ferda J, Baxa J, Ferdova E, Kucera R, Topolcan O, Molacek J. Abdominal aortic aneurysm in prostate cancer patients: the “road map” from incidental detection to advanced predictive, preventive, and personalized approach utilizing common follow-up for both pathologies. EPMA J. 2019;10(4):415–423. 10.1007/s13167-019-00193-y.
5. Rahbar K, Ahmadzadehfar H, Kratochwil C, Haberkorn U, Schäfers M, Essler M, et al. German multicenter study investigating ^177^Lu-PSMA-617 radioligand therapy in advanced prostate cancer patients. J Nucl Med. 2017; 10.2967/jnumed.116.183194.
6. Hu R, Wang X, Zhan X. Multi-parameter systematic strategies for predictive, preventive and personalised medicine in cancer. EPMA J. 2013; 10.1186/1878-5085-4-2.
7. Janssens JP, Schuster K, Voss A. Preventive, predictive, and personalized medicine for effective and affordable cancer care. EPMA J. 2018; 10.1007/s13167-018-0130-1.

**Targetable gene fusions in PPPM oriented glioma management**

Polivka J Jr^1,5^, Svajdler M*^2,3,4^, Martinek P^3^, Ptakova N^3^, Ostasov P^1^, Polivka J^5^

^1)^ Department of Histology and Embryology and Biomedical Center;

^2)^ Sikl’s Department of Pathology, Faculty of Medicine in Pilsen, Charles University, Czech Republic

^3)^ Biopticka laborator s.r.o, Pilsen, Czech Republic

^4)^ Cytopathos, s.r.o., Bratislava, Slovakia

^5)^ Department of Neurology, University Hospital Pilsen, Czech Republic

*Corresponding author: Marian Svajdler, MD, Ph.D., Biopticka laborator s.r.o., Mikulasske namesti 4, 326 00 Pilsen, Czech Republic; e.mail: svajdler@biopticka.cz

**Keywords:** Preventive predictive personalized medicine (PPPM), Glioma, Glioblastoma, Oncology, Neuro-oncology, Gene fusions, Targeted therapy, Next generation sequencing, Molecular genetics

**Introduction**

Gliomas are a primary malignancy of the central nervous system (CNS) [1]. Particularly high grade gliomas show poor prognosis with limited survival despite standard therapy. A novel approach is needed to advance glioma management based on predictive diagnostics; targeted preventive measures, as well as patient stratification should be carried out as part of the individualization of treatment algorithms [2–8]. Treatment personalization using targeted anticancer drugs and immunotherapy is the subject of extensive research in neuro-oncology [9,10]. Gene fusions were identified as an emerging therapeutic target for gliomas [11]. In this article we describe our use of next-generation sequencing to identify targetable gene fusions. We also discuss an innovative preventive, predictive, personalized medicine (PPPM) oriented approach to glioma management.

**Patients and Methods**

Patients with CNS gliomas were enrolled in the study between 2017 and 2019. Patients underwent a histopathological evaluation according to the WHO [1]. Mutation analysis and fusion transcript detection in FFPE samples were performed using TruSight Tumor 170 assay (Illumina, USA). All nucleic acids were extracted using a FFPE DNA kit (automated on RSC 48 Instrument, Promega, USA), quantified using Qubit Broad Range DNA and RNA Assays (Thermo Fisher Scientific, USA), and the quality of DNA was assessed using FFPE QC kit (Illumina), and the quality of RNA using Agilent RNA ScreenTape Assay (Agilent, USA). DNA samples with Cq < 5 and RNA samples with DV200 ≥ 20 were used. After DNA fragmentation with KAPA FragKit (KAPA Biosystems, USA), DNA and RNA libraries were prepared with TruSight Tumor 170 assay (Illumina) and sequenced on NextSeq 500 sequencer (Illumina). Data analysis was performed using TruSight Tumor 170 application on BaseSpace Sequence Hub (Illumina). DNA variant filtering and annotation were performed using Variant Interpreter (Illumina). The custom variant filter set up included only variants with coding consequences and a GnomAD database frequency value < 0.01 [12]. The remaining subset of variants was checked visually and suspected artifactual variants excluded. Additionally, fusion transcripts were detected by two FusionPlex kits (Solid Tumor and Comprehensive Thyroid and Lung) [13].

**Results**

Patients with gene fusions are presented in Table 1. Potential for experimental personalized treatment is listed.

**Table 1** Patients with gene fusions; histopathological diagnosis and potential for experimental therapy

**Table:** 

Age	Gene fusion	Diagnosis	Experimental therapeutics [11]
4	KIAA1549-BRAF	Pilocytic astrocytoma	**Second generation BRAF inhibitors** PLX-PB3 **MEK inhibitors** tramet inib selumetinib
4		Pilomyxoid astrocytoma	
7		Pilocytic astrocytoma	
12		Pilocytic astrocytoma	
8		Pilocytic astrocytoma	
2		Pilocytic astrocytoma	
5		Pilocytic astrocytoma	
11		Pilocytic astrocytoma	
38		Diffuse leptomeningealglioneuronal tumor	
33		Ganglioglioma	
1		Pilocytic astrocytoma	
6		Pilocytic astrocytoma	
33		Anaplastic pilocytic astrocytoma	
55	SRGAP3-BRAF	Pilocytic astrocytoma	
9	C11orf95-RELA	Anaplastic ependymoma	–
38		Anaplastic ependymoma	
20	RAF1-TRIM2	Pilocytic astrocytoma	**MEK inhibitors** trametinib selumetinib
8	EWSR1-PALGL1	Low grade glial tumor, NOS	–
43	FGFR1-TACC1	Pilocytic astrocytoma	**FGFR inhibitors** erdafitinib BGJ398 AZD4547
35	FGFR3-TACC3	Glioblastoma, IDH-wildtype	
55		Glioblastoma, IDH-wildtype	
53		Glioblastoma, IDH-wildtype	
44		Oligodendroglioma	
55		Anaplastic astrocytoma, IDH-wildtype, Grade III	
65		Anaplastic astrocytoma, IDH-wildtype	
34	FGFR2-CTNNA3	Diffuse astrocytoma, IDH-wildtype	
67	FGFR3-CKAP5	Glioblastoma, IDH-wildtype	
62	FGFR3-AMBRA1	Anaplastic oligodendroglioma, IDH-mutant with 1p/19 codeletion	
68	SRPK2-MET	Glioblastoma, IDH-wildtype	**MET inhibitors** foretinib crizotinib
40	PTPRZ1-MET	Glioblastoma, IDH-wildtype	
70	EGFR-SEPT14	Glioblastoma, IDH-wildtype	**EGFR inhibitors** erlotinib lapatinib
48		Glioblastoma, IDH-wildtype	
59	TERT-ALK	Glioneuronal tumor with neuropil-like islands(grade III astrocytoma, IDH-wildtype)	**ALK inhibitors** entrectinib crizotinib

**Expert recommendation and PPPM relevant outlook**

Several cancer syndromes increase the risk of gliomas (Neurofibromatosis 1/2, Tuberous sclerosis, Lynch syndrome, Li–Fraumeni syndrome, Melanoma-neural system tumor syndrome, Ollier disease) [14] as well as seven genomic variants (TERT, rs2736100; EGFR, rs2252586 and rs11979158; CCDC26, rs55705857; CDKN2B, rs1412829; PHLDB1, rs498872; TP53, rs78378222; RTEL1, rs6010620). Beyond genomics, specific phenotypic characteristics may play a role in predisposition to CNS malignancies [15]. Innovative algorithms reflecting these risk factors must be developed to implement predictive diagnostics to glioma management.

Since the exposure to ionizing radiation is of the highest value as a predictive risk evaluation factor for gliomas, with an observed dose-response relationship, preventive measures must be oriented at avoiding irradiation of subjects [14]. Prevention of other environmental hazards potentially associated with glioma development such as toxins (N-nitroso compounds, pesticides) should also be stressed [16].

More precise patient stratification that will help personalize the treatment algorithms to match patient profiles (personalized medicine) can be achieved by employing advanced molecular technologies as was shown in our study. Patients with specific targetable gene fusions could benefit from experimental therapies which might improve their quality of life and survival in case of standard treatment options failure.

**Acknowledgement**

Supported by Charles University Research Fund (Progres Q39); MH CZ-DRO (University Hospital Plzen-FNPl, 00669806); National Sustainability Program I (NPU I) Nr. LO1503 provided byMEYS CZ.

**References**

1. Louis DN, Perry A, Reifenberger G, von Deimling A, Figarella-Branger D, Cavenee WK, et al. The 2016 World Health Organization Classification of Tumors of the Central Nervous System: a summary. Acta Neuropathol (Berl). 2016;131:803–20.
2. Janssens JP, Schuster K, Voss A. Preventive, predictive, and personalized medicine for effective and affordable cancer care. EPMA J. 2018;9:113–23.
3. Grech G, Zhan X, Yoo BC, Bubnov R, Hagan S, Danesi R, et al. EPMA position paper in cancer: current overview and future perspectives. EPMA J. 2015;6:9.
4. Golubnitschaja O, Baban B, Boniolo G, Wang W, Bubnov R, Kapalla M, et al. Medicine in the early twenty-first century: paradigm and anticipation - EPMA position paper 2016. EPMA J. 2016;7:23.
5. Qian S, Golubnitschaja O, Zhan X. Chronic inflammation: key player and biomarker-set to predict and prevent cancer development and progression based on individualized patient profiles. EPMA J. 2019;10:365–81.
6. Golubnitschaja O, Kinkorova J, Costigliola V. Predictive, Preventive and Personalised Medicine as the hardcore of “Horizon 2020”: EPMA position paper. EPMA J. 2014;5:6.
7. Seifirad S, Haghpanah V. Inappropriate modeling of chronic and complex disorders: How to reconsider the approach in the context of predictive, preventive and personalized medicine, and translational medicine. EPMA J. 2019;10:195–209.
8. Golubnitschaja O, Polivka J, Yeghiazaryan K, Berliner L. Liquid biopsy and multiparametric analysis in management of liver malignancies: new concepts of the patient stratification and prognostic approach. EPMA J. 2018;9:271–85.
9. Polivka J, Polivka J, Holubec L, Kubikova T, Priban V, Hes O, et al. Advances in experimental targeted therapy and immunotherapy for patients with glioblastoma multiforme. Anticancer Res. 2017;37:21–33.
10. Polivka J, Krakorova K, Peterka M, Topolcan O. Current status of biomarker research in neurology. EPMA J. 2016;7:14.
11. Xu T, Wang H, Huang X, Li W, Huang Q, Yan Y, et al. Gene fusion in malignant glioma: an emerging target for next-generation personalized treatment. Transl Oncol. 2018;11:609–18.
12. Lek M, Karczewski KJ, Minikel EV, Samocha KE, Banks E, Fennell T, et al. Analysis of protein-coding genetic variation in 60,706 humans. Nature. 2016;536:285–91.
13. Švajdler M, Michal M, Martínek P, Ptáková N, Kinkor Z, Szépe P, et al. Fibro-osseous pseudotumor of digits and myositis ossificans show consistent COL1A1-USP6 rearrangement: a clinicopathological and genetic study of 27 cases. Hum Pathol. 2019;88:39–47.
14. Ostrom QT, Bauchet L, Davis FG, Deltour I, Fisher JL, Langer CE, et al. The epidemiology of glioma in adults: a “state of the science” review. Neuro-Oncol. 2014;16:896–913.
15. Polivka J, Kralickova M, Polivka J, Kaiser C, Kuhn W, Golubnitschaja O. Mystery of the brain metastatic disease in breast cancer patients: improved patient stratification, disease prediction and targeted prevention on the horizon? EPMA J. 2017;8:119–27.
16. Vienne-Jumeau A, Tafani C, Ricard D. Environmental risk factors of primary brain tumors: A review. Rev Neurol (Paris). 2019;

**Potential of extracellular long non-coding RNAs and mRNAs in plasma as a source of novel biomarkers for the personalization of colorectal cancer treatment**

Ostasov P*^1,2^, Rosendorf J^1,3^, Vycital O^1,3^, Palek R^1,3^, Polivka J^1,2^, Holubova M^1^, Liska V^1,3^

^1^Biomedical Center, ^2^Department of Histology and Embryology, Faculty of Medicine in Pilsen, Charles University, Pilsen, Czech Republic

^3^Department of Surgery, University Hospital and Faculty of Medicine in Pilsen, Charles University, Pilsen, Czech Republic

*Corresponding author: Pavel Ostasov, Biomedical Center, Faculty of Medicine in Pilsen, Charles University, Czech Republic; e.mail: pavel.ostasov@lfp.cuni.cz

**Keywords:** preventive predictive personalized medicine (PPPM), cell-free RNA, treatment personalization, biomarkers, colorectal cancer, liquid biopsy, differential expression, mRNA, long non-coding RNA, sequencing

**Introduction**

Colorectal cancer was estimated to be the second most frequent cause of cancer related death in the EU among men and the third among women in 2019 [1]. New clinically relevant biomarkers could play an important role in prevention and are needed for a more efficient management of patient quality of life [2]. Unfortunately, there has been a decrease in the detection of new, clinically useful biomarkers [3]. Interestingly, cell-free long non-coding RNAs and mRNAs (cfRNA) in plasma have received minimal attention. We have therefore tested their potential as a source of novel biomarkers for the personalization of colorectal cancer treatment.

**Patients and methods**

Two data sets were used for the analysis. The public one contained datasets GSE100206 [4] with 32 normal samples and GSE100063 [5] with 12 colorectal cancer samples. The dataset produced in our laboratory was derived from cell-free plasma collected from patients with colorectal cancer and healthy controls in 2018 and 2019 at the University Hospital in Pilsen. After precipitation by 8% PEG 6000, RNA was isolated using a miRNA easy serum/plasma kit (Qiagen). Libraries were prepared using a NEB next Ultra II directional RNA kit (NEB). Kallisto and DeSeq2 were used for differential gene expression.

**Results**

The analysis of gene expression from the public and our data yielded a set of 74 significantly differentially expressed genes in both data sets with the same direction of change (Table 1).

**Table 1** Genes significantly changed in both datasets between patients and healthy controls
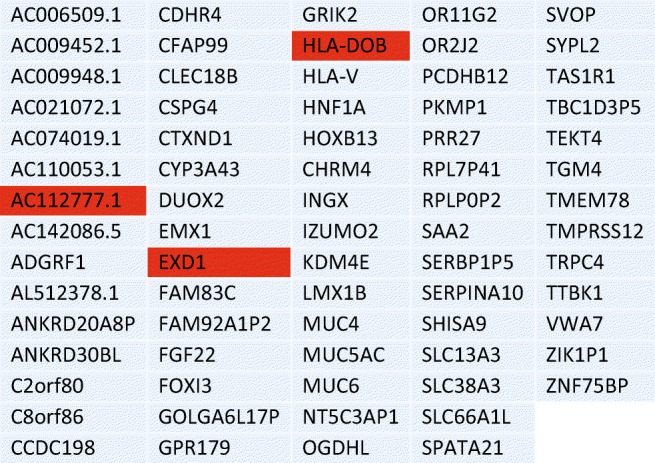


Upregulated genes are marked in blue, downregulated in red

**Conclusions and PPPM Expert Recommendations**

Genes such as hypoxia marker HIF1A, or those associated with mucosa and epithelial tissue maintenance (MUC4, MUC5AC, MUC6), were upregulated in patients with colorectal cancer. Downregulation was seen in many HLA molecules, although they differed within HLA types and only HLA-DOB was downregulated in both datasets. This indicates an alteration in the immune system of cancer patients. Overall, we were able to detect significant changes in gene expression of colorectal cancer patients’ cfRNA from plasma.

We recommend further investigation of cfRNAs for biomarker discovery as our results show that the physiological changes within the body are reflected in plasma cfRNA. The changes are likely coming not only from tumor cells but also other systems affected by disease, such as the immune system. This might prove useful for the personalization of immunotherapy. cfRNA, similar to other liquid biopsies, allows repeated collection of samples. This enables easy follow ups in patients and adds additional information about the course of the disease that will contribute to treatment personalization. The predictive potential of cfRNA can also be used for early detection and prevention of therapy side effects, such as liver damage. These could be effectively prevented by modifying the therapy regimen, leading to improvement of patients’ quality of life and possibly contributing to therapy effectiveness. As both 1) the collection of cell-free plasma and 2) isolation and preparation of cfRNA are relatively cheap, simple and unintrusive. cfRNA has the potential to become a routine practice with great potential for helping discover predictive markers. It could furthermore be easily combined with other technologies to form multilevel diagnostics that are necessary for a true shift to preventive, predictive, personalized medicine (PPPM) [6–10].

**Acknowledgements**

Supported by the Charles University Research Fund (Progres Q39), Charles University Research Centre program UNCE/MED/006 “University Center of Clinical and Experimental Liver Surgery”, European Regional Development Fund Project “Application of Modern Technologies in Medicine and Industry” (CZ.02.1.01/0.0/0.0/17_048/0007280) and by the National Sustainability Program I (NPU I) Nr. LO1503 by the Ministry of Education Youth and Sports of the Czech Republic.

**References**

1. Malvezzi M, Carioli G, Bertuccio P, Boffetta P, Levi F, La Vecchia C, et al. European cancer mortality predictions for the year 2019 with focus on breast cancer. Ann Oncol. 2019;30:781–7.
2. Grech G, Zhan X, Yoo BC, Bubnov R, Hagan S, Danesi R, et al. EPMA position paper in cancer: current overview and future perspectives. EPMA J. 2015;6(1):9–9.
3. Golubnitschaja O, Watson ID, Topic E, Sandberg S, Ferrari M, Costigliola V. Position paper of the EPMA and EFLM: a global vision of the consolidated promotion of an integrative medical approach to advance health care. EPMA J. 2013;4(1):12–12.
4. Li Y, Zheng Q, Bao C, Li S, Guo W, Zhao J, et al. Circular RNA is enriched and stable in exosomes: a promising biomarker for cancer diagnosis. Cell Res. 2015;25:981–4.
5. Li S, Li Y, Chen B, Zhao J, Yu S, Tang Y, et al. exoRBase: a database of circRNA, lncRNA and mRNA in human blood exosomes. NucleicAcids Res. 2018;46:D106–12.
6. Golubnitschaja O, Baban B, Boniolo G, Wang W, Bubnov R, Kapalla M, et al. Medicine in the early twenty-firstcentury: paradigm and anticipation - EPMA position paper 2016. EPMA J. 2016;7:23–23.
7. Janssens JP, Schuster K, Voss A. Preventive, predictive, and personalized medicine for effective and affordable cancer care. EPMA J. 2018;9:113–23.
8. Golubnitschaja O, Polivka J, Yeghiazaryan K, Berliner L. Liquid biopsy and multiparametric analysis in management of liver malignancies: new concepts of the patient stratification and prognostic approach. EPMA J. 2018;9:271–85.
9. Polivka J, Krakorova K, Peterka M, Topolcan O. Current status of biomarker research in neurology. EPMA J. 2016;7:14.
10. Seifirad S, Haghpanah V. Inappropriate modeling of chronic and complex disorders: How to reconsider the approach in the context of predictive, preventive and personalized medicine, and translational medicine. EPMA J. 2019;10:195–209.

**A personalised approach to differential diagnostics in CT imaging of peripheral lung adenocarcinoma**

Mirka H*^1,6^, Ferda J^1^, Krakorova G^2^, Vodicka J^3^, Mukensnabl P^4^, Topolcan O^5^, Kucera R^5^

^1^Department of Medical Imaging, ^2^Department of Pneumology and ftiseology, ^3^ Department of Surgery, ^4^ Sikl’s department of pathology, ^5^ Department of Immunochemistry Diagnostics, University Hospital and Faculty of Medicine in Pilsen, Charles University, Czech Republic; ^6^ Biomedical Centre, Faculty of Medicine in Pilsen, Charles University in Prague, Pilsen, Czech Republic;

*Corresponding author: Hynek Mirka, assoc. prof., MD, PhD, Department of Medical Imaging, University Hospital in Pilsen, Czech Republic; e.mail: mirka@fnplzen.cz, ORCID ID: 0000-0002-7546-5625

**Keywords:** Preventive predictive and personalised medicine, Lung, Tumour, Pulmonary nodule, Adenocarcinoma, Histological type, Invasivity, Diagnostic imaging, Computed tomography

**Introduction**

Lung cancer is a disease whose incidence has increased dramatically over the past hundred years. The most common histological type is adenocarcinoma [1]. New knowledge of the biology, clinics and radiological images of adenocarcinoma has changed the approach to its diagnosis and treatment, resulting in a new classification developed in 2011. Among other things, uncertainties regarding the variants of adenocarcinoma originally called bronchioloalveolar carcinoma have been removed [2].

Non-invasive and minimally invasive variants of lung adenocarcinoma grow very slowly and have a very good prognosis [3]. When these tumours are radically removed, patients have up to 100% survival [4].

The aim of this study is to assess the ability of CT to distinguish non-invasive and minimally invasive types of pulmonary adenocarcinoma by evaluation of the size of the solid component, thus allowing prediction and personalised decision making regarding the next therapeutic approach.

**Material and method**

We enrolled a total of 64 patients (38 men, 26 women, mean age 64 years) operated on for peripheral lung cancer with histological confirmation of adenocarcinoma and preoperative finding of a subsolid node on CT. CT scans were performed less than 1 month before surgery on Somatom Definition, Somatom Definition AS and Somatom Definition Flash multidetector devices (Siemens, Forchheim, Germany). Pulmonary nodes were evaluated on thin sections with a collimation of 0.6 mm reconstructed using the edge enhancement algorithm. The radiologist was blinded to the results of histological examination. A three-dimensional analysis was performed on all nodules, including the automatic measurement of the maximum dimensions of the entire node and the solid component (Fig. 1). The findings were correlated with histology. Nodes with a maximum size of 3 cm and a solid component of up to 5 mm were evaluated as non-invasive or minimally invasive tumour variants. We also calculated the volume doubling time for the nodes that were followed-up.
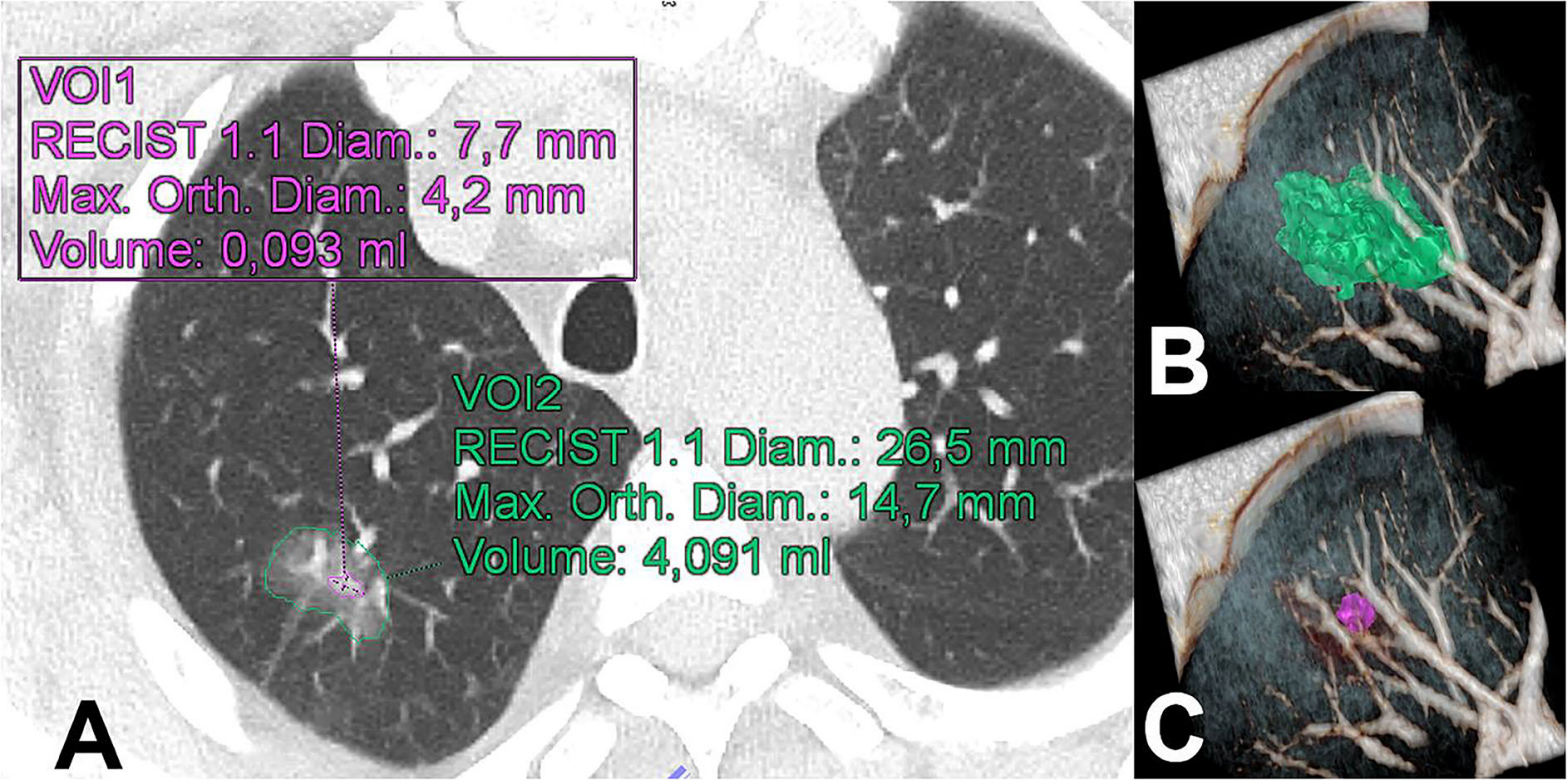


**Fig. 1** Three-dimensional automatic analysis of semisolid nodule in the upper right lobe. **a** transverse scan, a solid component in violet and a complete nodule in green. **b** three-dimensional model of the whole nodule. **c** three-dimensional model of solid part

**Results**

According to the histological examination, 18 tumours were non-invasive or minimally invasive and 46 were invasive. Results of CT findings were correctly evaluated as non-invasive or minimally invasive in 14 cases and as invasive in 45 cases. False positivity (overestimation of non-invasive minimally invasive tumour) occurred in four cases and false negativity (underestimation of invasive tumour) occurred in one case. Thus, the CT achieved a sensitivity of 77.8, a specificity of 97.8%, a positive predictive value of 93.3% and a negative predictive value of 91.8% when distinguishing non-invasive and minimally invasive variants of peripheral pulmonary adenocarcinoma. We could determine the doubling time in 28 patients. This was on average 677 days for non-invasive and minimally invasive variants and 588 days for invasive variants.

**Data Interpretation and PPPM-based Expert Recommendations**

The results of our study demonstrate that CT is able to distinguish between invasive and minimally invasive variants of lung adenocarcinoma. The differentiation of these tumour groups is important in predictive diagnostics and can potentially be used in making personalised decisions about a more precise treatment. For example, in determining the timing of surgical treatment, the extent of resection, or in deciding on the necessity systemic treatment. The extent of solid tumour component can be used as a predictor of invasiveness [7]. The gradual transition between solid and ground glass opacities where the boundary between the two components is very difficult to determine reduces CT sensitivity. Solid tumourous tissue may also be indistinguishable from collapsed lung, fibrous tissue or mucin accumulation. A possible solution could be to evaluate a solid component in the mediastinal window, which filters out lower densities [8]. The predictive potential of texture analysis using mathematical and statistical methods seems promising [9]. Finding a subsolid pulmonary nodule does not always mean that it is an adenocarcinoma. The literature indicates that up to 40% of these nodules may be of benign, primarily inflammatory, origin. For this reason, subsolid (but also solid) pulmonary nodules smaller than 8 mm are usually monitored preventively. Malignancy is suspected in nodules that tend to grow. This is expressed in terms of volume doubling time. It is generally accepted that malignancy suspects are nodules with a doubling time between 30 and 400 days. However, for subsolid nodule these times may be longer, which is also evident from our analysis. For this reason, subsolid nodules should be subject to targeted prevention in the form of longer monitoring intervals [10,11]. Another evaluation method of subsolid pulmonary nodes is PET/CT with fluorine-18-labeled fluorodeoxyglucose (18F-FDG). In contrast to the non-invasive component, 18F-FDG has been shown to accumulate in the invasive component. The problem however is the mucinous tumour variants which, despite the presence of the invasive component, may not show increased 18F-FDG uptake. It should also be noted that nodes smaller than 5–8 mm may be falsely negative due to their small size. In such cases the CT finding should be taken into account [12,13]. Inflammatory consolidations, such as FDG tumours, may accumulate and be a source of false positives.

In conclusion, personalised medicine enables a whole new approach to the treatment of patients with pulmonary adenocarcinoma. PPPM is optimal for the assessment of the tumour’s invasiveness and therefore contributes to the long- and short-term treatment strategies of pulmonary adenocarcinoma patients. The PPPM approach entails the use of biomarkers, imaging techniques and a clinical examination of the patient. A personalised management of the disease is more precise, can help time surgery better, decide on the extent of resection and possibly indicate a systemic treatment that can play a role in secondary prevention.

**Acknowledgements**

Supported by Ministry of Health - Conceptual Development of research institutions 00669806, by the project CZ.1.05/2.1.00/03.0076 from European Regional Development Fund and Program Progress Q39 of the Charles University Prague.

**References**

1. de Groot PM, Wu CC, Carter BW, Munden RF. The epidemiology of lung cancer. Transl Lung Cancer Res. 2018;7:220–233.
2. Travis WD, Brambilla E, Nicholson AG, Yatabe Y, Austin JHM, Beasley MB, et al. The 2015 World Health Organization Classification of LungTumors. Impact of Genetic, Clinical and Radiologic Advances Since the 2004. Classification. J Thorac Oncol. 2015;10:1243–1260.
3. Russell PA, Wainer Z, Wright GM, Daniels M, Conron M, Williams RA. Does Lung Adenocarcinoma Subtype Predict PatientSurvival? A Clinicopathologic Study Based on the New International Association for the Study of Lung Cancer/American Thoracic Society/European Respiratory Society International Multidisciplinary Lung Adenocarcinoma Classification. J Thorac Oncol. 2011;6:1496–1504.
4. Aherne EA, Plodkowski AJ, Montecalvo J, Hayan S, Zheng J, Capanu M, et al. What CT characteristics of lepidic predominant pattern lung adenocarcinomas correlate with invasiveness on pathology? Lung Cancer. 2018;118:83–89.
5. Ichinose J, Kohno T, Fujimori S, Harano T, Suzuki S, Fujii T. Invasiveness and malignant potential of pulmonary lesions presenting as pure ground-glass opacities. Ann Thorac Cardiovasc Surg. 2014; 20:347–352.
6. Saito H, Kameda Y, Masui K, Murakami S, Kondo T, Ito H, et al. Correlations between thin-section CT findings, histopathological and clinical findings of small pulmonary adenocarcinomas. Lung Cancer. 2011;71:137–143.
7. Liu Y, Liu S, Qu F, Li Q, Cheng R, Ye Z. Tumor heterogeneity assessed by texture analysis on contrast-enhanced CT in lung adenocarcinoma: association with pathologic grade. Oncotarget. 2017; 10.18632/oncotarget.15399.
8. MacMahon H, Naidich D, Goo JM, Lee KS, Leung ANC, Mayo JR, et al. Guidelines for Management of Incidental Pulmonary Nodules Detected on CT Images: From the Fleischner Society 2017. Radiology. 2017;284:228–224.
9. Qian S, Golubnitschaja O, Zhan X. Chronic inflammation: key player and biomarker-set to predict and prevent cancer development and progression based on individualized patient profiles. EPMA J. 2019; 10.1007/s13167-019-00194-x.
10. Wu H, Wang L, Wang Q, Han Y, LI H, Zhou W, Tian Y. Adenocarcinoma with BAC Features Presented as the Nonsolid Nodule Is Prone to Be False-Negative on 18F-FDG PET/CT. BioMed Res Int. 2015; 10.1155/2015/243681.
11. Hu XL, Xu ST, Wang XC, Luo JL, Hou DN, Zhang XM, et al. Development and validation of nomogram estimating post-surgery hospital stay of lung cancer patients: relevance for predictive, preventive, and personalized healthcare strategies. EPMA J. 2019; 10.1007/s13167-019-00168-z.

**An Overview of Cancer Biomarker Strategies in the PPPM Pipeline**

Windrichova J*^1^, Topolcan O^1^, Rezackova H^1^, Fuchsova R^1^, Pesta M^2^, Kucera R^1^

^1^Department of Immunochemistry Diagnostics, Faculty Hospital in Pilsen ^2^Department of Biology, Faculty of Medicine in Pilsen, Charles University in Prague, Czech Republic

*Corresponding author: Jindra Windrichova, Ph.D., Department of Immunochemistry Diagnostics, Faculty Hospital in Pilsen, CZ; e.mail: windrichovaj@fnplzen.cz, ORCID ID: 0000-0003-0365-0968,

**Keywords:** preventive predictive personalised medicine, biomarker pipeline, transcriptomic, multi-omics, microRNA, multi-objective optimisation, biobanks, molecular biology biomarkers, multiplexing, computational method, evolutionary algorithm

**Introduction**

The aims behind the use of biomarkers are in concordance with the principles of PPPM. Biomarkers enable the prediction of individual predisposition before disease on-set, the provision of targeted preventive measures and the creation of personalised treatment algorithms tailored to the individual [1]. They are strategically important for early diagnosis, patient stratification, assessment of drug toxicity and efficacy, disease risk, staging and prognosis. Biomarker applicability greatly depends on a proper process of transition of each biomarker from primary research to proper clinical evaluation. A standardised validation of biomarkers is crucial for PPPM application in routine medical practice [2]. The laboratory services have to become more complex, not only providing a multifactorial analysis but also including recommendations and active advice for clinicians [3]. Practical implementation of novel and complex laboratory tests should be considered according to their scientific value, cost-effectiveness and added value for patients [4].

**What is the current situation in the field of biomarkers?**

A wide range of clinically used markers incorporates classical tumour markers with unknown physiological roles or increased/decreased factors of protein physiology, e.g. growth factors, angiogenetic factors or apoptotic factors. In recent times there has been a rise, not only in single candidate biomarkers or their combinations but also in a whole novel class of regulatory molecules from the fields of transcriptomics and genomics. The expanding interest in molecular biology for preventive, predictive and personalised medicine applications could be illustrated by studies of cell free miRNA in serum for potential biomarkers [5]. An example of this are the biomarkers for hepatocellular carcinoma long non-coding RNA Myd88, methylation of SEPT9 promoter or microRNA: miR-122, miR-21 and miR192 for diagnostics, and plasma miRNAs and low PD-L1 molecule expression or variations in oncogenes for targeted therapy [6]. Traditional markers are studied in novel formats; e.g. protein chip detection for diagnosis of pancreatic cancer incorporating CA19-9, NSE, CEA, CA242 and CA125 [7]. The promising results support combinations of molecular and traditional routine biomarkers, successfully combined microRNA and CEA and CA19-9 in colorectal cancer [8].

**Predictive Biomarkers for Proper Treatment Management**

Predictive molecular biomarkers are already routinely used for treatment and personalised stratification of patients in clinical practice. To speed up research in the molecular biomarker field, new molecular markers are often correlated with traditionally used biomarkers. Alternatively, the data driven approach is currently employed as a further option. This is based on arrays of molecules designed to determine response/non-response to treatment or presence/absence of disease. It is then followed by molecular differential expression analysis for the selection of individual molecules for further validation. To further these novel approaches to systematic biomarker selection, the growing number of databases and computational review systems should be used [9].

**The Systematic Genome, Transcriptome and Multiomics Approach**

The next level in biomarker studies is a broad approach focused on systematic genome/transcriptome/multi-omics. This type of study is driven by large projects such as genome-wide research projects. The progress in genomics helping understand cancer, accompanied by a whole range of other omics, has created a new biomarker multiplatform in PPPM medicine [10]. Golubnitschaja et al. proposed multi-omics as a promising tool for diagnosis, targeted prevention and personalised treatments in the management of triple-negative breast cancer [11]. The development of high-output and cost-effective multiple omics technologies promotes an efficacious treatment of cancer [12].

**Biobanks and New Biomarker Testing**

Over the past 30 years, biobanking has grown dramatically and has become an important source for biomarker research. Biobanks are one of the pillars of personalised medicine. Samples stored in biobanks are an important source of biological material with appropriate data. Being as “civilisation diseases” such as cardiovascular diseases, diabetes, neurodegenerative diseases or cancer are currently on the rise, biobanks can help research, speeding up the search for successful solutions to these serious diseases. Using biobank sources, both individual biomarkers or multiparameter models can quickly be tested and afterwards implemented into routine clinical practice [13].

**Expert Recommendations and PPPM Relevance:**

- New options for biomarker discovery have already become available and should be properly used to add new biomarkers to those currently routinely used.
- Multi-omics, as well as computational frameworks, are highly promising approaches that should be included in the pipeline for the development of predictive diagnostic biomarkers, biomarkers for targeted prevention and personalised treatment in general.
- The most promising approaches in the search for personalised patient-pathways and markers are those based on system knowledge and multi-objective optimisation while taking into account the predictive power and functional relevance of the biomarker. Samples stored in biobanks can speed up current research into “civilisation diseases” and help to find successful solutions to these serious conditions.

**Acknowledgements:** Supported by Ministry of Health, Czech Republic - conceptual development of research organization (Faculty Hospital in Pilsen - FNPl, 00669806) and BBMRI-CZ: Biobank network - a versatile platform for the research of the etiopathogenesis of diseases CZ.02.1.01/0.0/0.0/16_013/0001674, Bank of the clinical samples LM2018125.

**References**

1. Golubnitschaja O, Kinkorova J, Costigliola V. Predictive, Preventive and Personalised Medicine as the hardcore of ‘Horizon 2020’: EPMA position paper. EPMA J. 2014; 10.1186/1878-5085-5-6.
2. Golubnitschaja O, Costigliola V, EPMA. General report & recommendations in predictive, preventive and personalised medicine 2012: white paper of the European Association for Predictive, Preventive and Personalised Medicine. EPMA J. 2012; 10.1186/1878-5085-3-14.
3. Golubnitschaja O, Baban B, Boniolo G, Wang W, Bubnov R, Kapalla M, et al. Medicine in the early twenty-first century: paradigm and anticipation - EPMA position paper 2016. EPMA J. 2016; 10.1186/s13167-016-0072-4.
4. Golubnitschaja O, Watson I, Topic E, Sandberg S, Ferrari M, Costigliola V. Position paper of the EPMA and EFLM: a global vision of the consolidated promotion of an integrative medical approach to advance health care. 2013; 10.1186/1878-5085-4-12.
5. de Ronde MWJ, Ruijter JM, Moerland PD, Creemers EE, Pinto-Sietsma S. Study Design and qPCR Data Analysis Guidelines for Reliable Circulating miRNA Biomarker Experiments: A Review. Clin Chem. 2018; 10.1373/clinchem.2017.285288.
6. Duan J, Wu Y, Liu J, Zhang J, Fu Z, Feng T, et al. Genetic Biomarkers For Hepatocellular Carcinoma In The Era Of Precision Medicine 2019; 10.2147/JHC.S224849.
7. Liu F, Du F, Chen X. Multiple tumor marker protein chip detection system in diagnosis of pancreatic cancer. World J Surg Oncol. 2014; 10.1186/1477-7819-12-333.
8. Pesta M, Kucera R, Topolcan O, Karlikova M, Houfkova K, Polivka J, et al. Plasma microRNA Levels Combined with CEA and CA19-9 in the Follow-Up of Colorectal Cancer Patients. Cancers (Basel). 2019; 10.3390/cancers11060864.
9. Rigden DJ, Fernández X. The 26th annual Nucleic Acids Research database issue and Molecular Biology Database Collection. 2018; 10.1093/nar/gky1267.
10. Janssens JP, Schuster K, Voss A. Preventive, predictive, and personalised medicine for effective and affordable cancer care. EPMA J. 2018; 10.1007/s13167-018-0130-1.
11. Golubnitschaja O, Filep N, Yeghiazaryan K, Blom HJ, Hofmann-Apitius M, Kuhn W. Multi-omic approach decodes paradoxes of the triple-negative breast cancer: lessons for predictive, preventive and personalised medicine. Amino Acids. 2018; 10.1007/s00726-017-2524-0.
12. Lu M, Zhan X. The crucial role of multiomic approach in cancer research and clinically relevant outcomes. EPMA J. 2018; 10.1007/s13167-018-0128-8.
13. Kinkorová J, Topolčan O. Biobanks in Horizon 2020: sustainability and attractive perspectives. EPMA J. 2018; 10.1007/s13167-018-0153-7.

**Microcells as a possible predictive factor for tumour treatment efficiency**

Simsone Z^*1^, Freivalds T^1^, Bēma D^1^, Harju L^1^, Bērziņš J^1^, Buiķis I^1^

1. University of Latvia, Institute of Cardiology and Regenerative Medicine, Jelgavas Str. 3, Riga, LV-1004, Latvia

*Corresponding author: e.mail: z.simsone@gmail.com

**Keywords:** Biomarkers, microcell, resistance prediction, individualized posttreatment cell population characterization, predictive factor selection, individualized treatment, SOX2, NANOG, PCNA

**Introduction**

Biomarkers are efficient tools (features) for predicting specific types of diseases, malignant and/or benign, as well as enabling researchers to distinguish the origin of the various types of normal cells and tissues in a patient. One can say that the personalization of specific features may influence the treatment as well as possible outcome of disease [1].

Several biomarkers have been identified in cancer patients having significant value for treatment prediction of the malignant diseases [2,3].

As a rule, these markers have been estimated before the perspective treatment of the patients with the aim to predict the outcome of treatment applied and prevent therapy side-effects [4]. SOX2 and NANOG are markers usually associated with cancer stem cells [5]. The presence of high numbers of undifferentiated stem cells in a tumour usually is a marker of poor prognosis for the survival of the cancer patient, and consequently, low treatment efficacy [6]. As surprisingly as it may seem, these surviving cells are rarely being searched for and estimated after treatment procedures. Their features are rarely studied. Partly, it is explained by difficulties of obtaining these cells and studying them in vivo.

Our previous investigations in cell cultures undergoing changes caused by chemotherapy agents have shown the development of a population of small-sized cells, called “microcells” [7]. Microcells are part of the cancer population and their count usually increases after treatment with anticancer agents. Phenotypically, they are small round or oval cells with small rim of cytoplasm around the nucleus, and they look darker when stained.

**Working hypothesis, materials and methods**

Our present hypothesis postulates that these microcells gain resistance after treatment and present the anticancer treatment resistant population within the tumour. The aim of the present investigation is to reveal possible biomarkers of this hypothetically treatment resistant cell population within tumours under the influence of anticancer agents.

Melanoma cells culture SK-MEL-28 were acquired from the American Type Culture Collection and maintained as producer recommended. All cell lines were seeded into 24 multi-well plates with an initial density of ~1 × 10^5^ cells/well and grown in a humidified atmosphere containing 5% CO_2_ at 37 °C.

Paclitaxel (PTX, Paclitaxel - TEVA 6 mg/ml) was used as a stress factor for 24 h at the end concentration of 0.6 mg/ml at 37 °C in a 5% CO_2_ atmosphere for cancer microcell induction. A cultivation medium was replaced with fresh medium after treatment, and cells were cultivated for 48 h at 37 °C in a 5% CO_2_ atmosphere.

SK-MEL-28 cells were immunocytochemically stained using standard technique and labelled with primary antibodies SOX2, NANOG and PCNA antibody, conjugated with relevant secondary antibody Alexa fluor 488 or Alexa fluor 594 as manufacturer recommended (Abcam, Origene). Nuclei were counter stained with DAPI (MP Biomedicals) and cells mounted in CV Ultra (Leica).

The Leica SP8 Confocal and ZEISS LSM900 microscopes and 63x objectives (oil, apochromatic, aperture value 1.40) were used. LAS X lite (Leica Microsystems) and ZEN Blue lite (ZEISS) image analysis and processing programs were used.

**Results and data interpretation**

PCNA (proliferating cell nuclear antigen) expression (Fig. 1) mainly is related to cell replication and is involved in DNA repair systems [8]; otherwise in control cells, PCNA expression is observed in cell nuclei and in cytoplasm. PCNA expression in cancer cells is more likely as a result of post-translation modification, and it is related to malignant breast cells [9]. After PTX treatment, we observed microcells near macrocell nuclei (Fig. 1b, c white➔). These are functional, small (only 2.56 μm per diameter) cells containing DNA (Fig. 1C white➔). Increased expression of PCNA represents high replication activity in microcells (Fig. 2b and c), and it indicates their high capacity for renewal.
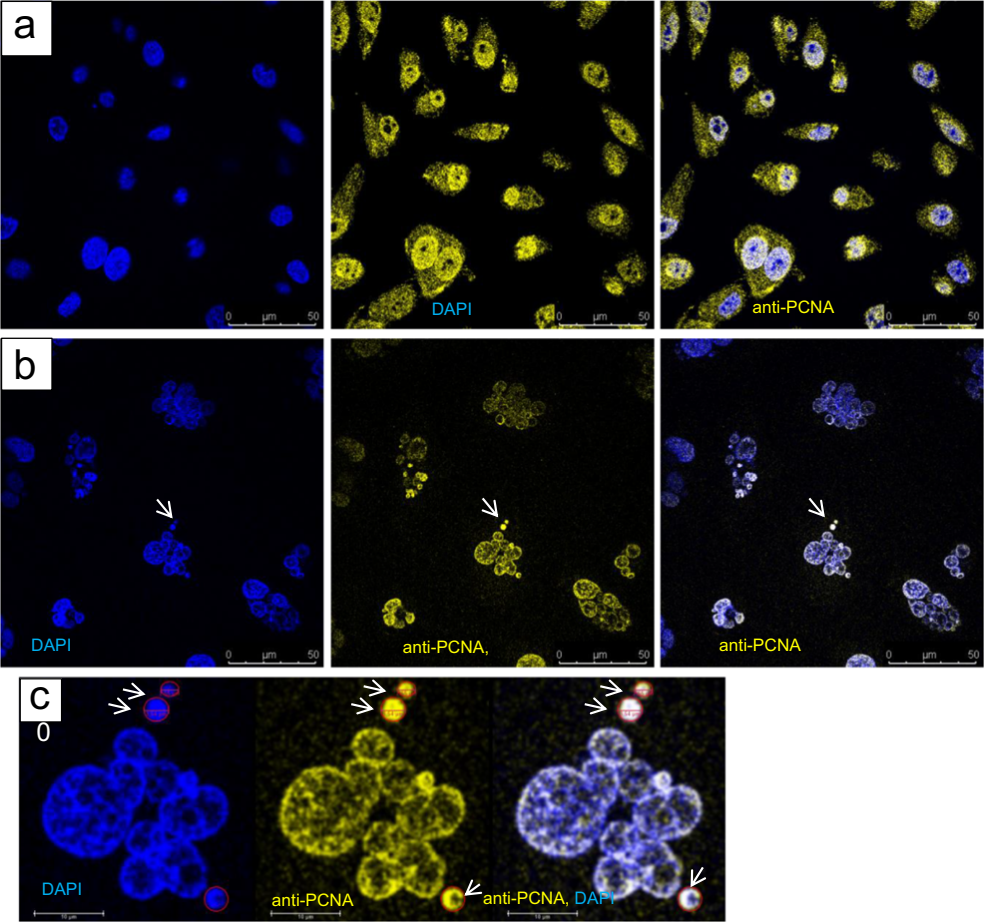


**Fig. 1** The expression of PCNA antigen in SK-MEL-28 cell. (**a**) Control cells stained against anti-PCNA (1:100; Abcam yellow-Alexa fluor 594, Abcam), nuclei stained with DAPI (0.2 μg/ml, MP Biomedical blue), overlay DAPI (blue), PCNA (yellow). (**b**) PTX 0.6 mg/ml treated cells stained against PCNA (yellow), nuclei stained with DAPI (blue) after 48 h. Overlay DAPI (blue), anti-PCNA (yellow). (**c**) Microcells (white➔) are small-sized cells with a diameter from 2.56 to 3.54 μm up to 5 μm

SOX2 is involved in somatic cell reprogramming, reversing the epigenetic configuration of dedifferentiation of cells [10]. After PTX treatment (Fig. 2D, SOX2), the antigen SOX2 shows higher expression in nuclei than control cells (Fig. 2a, SOX2). SOX2 expression have been shown as a potential CSC marker. Noteworthy, NANOG-cell nucleus marker, is expressed in cell cytoplasm equally strong in both control and treated cells (Fig. 2, NANOG). The antigen NANOG expression in cell cytoplasm points to cell malignancy and therapy resistance [11]. SOX2 and NANOG biological markers are core proteins in stemness and regulating transcription factors in pluripotency to preserve cell self-renewal [5]. Microcells indicate homogeneous DNA labelling and high expression of stem cell biomarkers, which characterize microcell metabolic activity after anti-cancer treatment.
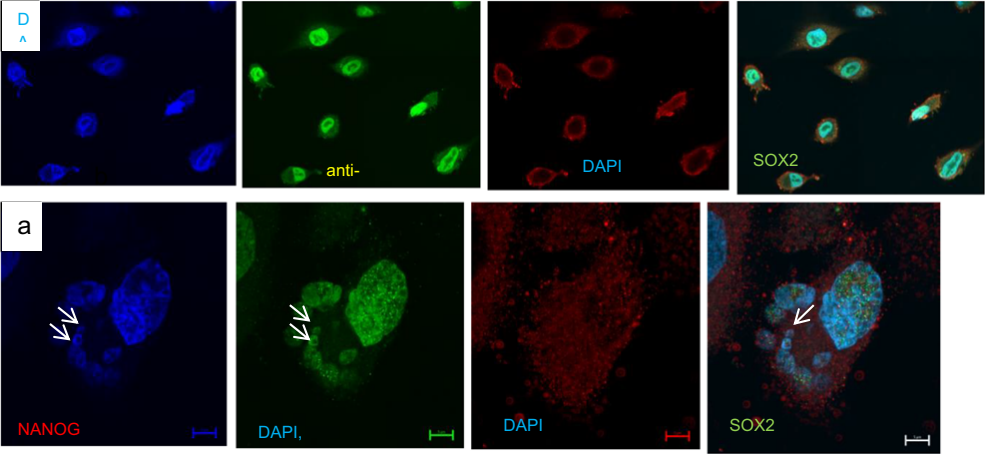


**Fig. 2** Expression images

**Conclusions**

Compared to control cells, microcells have a higher capacity of renewal and, at the same time, the ability to dedifferentiate to a pluripotent cell, thus presenting an important source of cancer resistance to repeated administration of anticancer treatment. One can postulate that microcells are a predictive resistance factor of utmost importance. Newly developed microcells acquire increased mobility and show potency regarding endocytosis. Therefore, analysis of the microcell population in a tumour undergoing anticancer treatment could be a strong prognostic factor for patient’s survival, and on the other hand, be a basis for individualized characterization (and profiling) because the cell population future structure changes during treatment of a patient.

**Acknowledgements**

This project is supported by the University of Latvia Donor SIA “Mikrotīkls”. The University of Latvia Foundation administered this donation.

The cell culture cultivation was carried out in cooperation with the Latvian Biomedical Research and Study Centre.

The confocal microscopy was carried out in collaboration with Carl Zeiss Microscopy Democenter Oberkochen, Germany.

**References**

1. Janssens JP, Schuster K, Voss A. Preventive, predictive, and personalized medicine for effective and affordable cancer care. EPMA J. 2018;9:113–23. https://pubmed.ncbi.nlm.nih.gov/29896312
2. Sacco K, Grech G. Actionable pharmacogenetic markers for prediction and prognosis in breast cancer. EPMA J. 2015;6:4–9. 10.1186/s13167-015-0037-z
3. Lu M, Chen W, Zhuang W, Zhan X. Label-free quantitative identification of abnormally ubiquitinated proteins as useful biomarkers for human lung squamous cell carcinomas. EPMA J. 2020;73–94. 10.1007/s13167-019-00197-8
4. Qian S, Golubnitschaja O, Zhan X. Chronic inflammation: key player and biomarker-set to predict and prevent cancer development and progression based on individualized patient profiles. EPMA J. 2019;10:365–81. 10.1007/s13167-019-00194-x
5. Yu P, Nie Q, Tang C, Zhang L. Nanog induced intermediate state in regulating stem cell differentiation and reprogramming. BMC Syst Biol. 2018;12:1–13. 10.1186/s12918-018-0552-3
6. Lu EM-C, Ratnayake J, Rich AM. Assessment of proliferating cell nuclear antigen (PCNA) expression at the invading front of oral squamous cell carcinoma. BMC Oral Health. 2019;19:233. 10.1186/s12903-019-0928-9
7. Buiķis I, Harju L, Freivalds T. Origin of microcells in the human sarcoma cell line HT-1080. Anal Cell Pathol. IOS Press; 1999;18:73–85. 10.1155/1999/461805
8. Peng A, Xu X, Wang C, Yang J, Wang S, Dai J, et al. EZH2 promotes DNA replication by stabilizing interaction of POLδ and PCNA via methylation-mediated PCNA trimerization. Epigenetics and Chromatin. BioMed Central; 2018;11:1–14. 10.1186/s13072-018-0213-1
9. Malkas LH, Herbert BS, Abdel-Aziz W, Dobrolecki LE, Liu Y, Agarwal B, et al. A cancer-associated PCNA expressed in breast cancer has implications as a potential biomarker. Proc Natl Acad Sci U S A. 2006/12/11. National Academy of Sciences; 2006;103:19472–7. www.pnas.org/cgi/doi/10.1073/pnas.0604614103
10. Debeb BG, Zhang X, Krishnamurthy S, Gao H, Cohen E, Li L, et al. Characterizing cancer cells with cancer stem cell-like features in 293 T human embryonic kidney cells. Mol Cancer. 2010;9:1–12. 10.1186/1476-4598-9-180
11. Gu T-T, Liu S-Y, Zheng P-S. Cytoplasmic NANOG-Positive Stromal Cells Promote Human Cervical Cancer Progression. Am J Pathol. Elsevier; 2012;181:652–61.: 10.1016/j.ajpath.2012.04.008

## 3PM in ophthalmology

**Comparative proteomic analysis of tear fluid aimed at prediction, targeted prevention and treatments tailored to the patient with diabetic cataract**

Kovalevskaia MA*^1^, Kunin AA^2^, FilinaLA^1^, Vladimirova YV^1^, Veremeenko AI^1^, Golubnitschaja O^3^

^1^Department of Ophthalmology, Voronezh State Medical University named after N.N. Burdenko, Ministry of Healthcare, Voronezh, Russian Federation

^2^Department of Dentistry, Voronezh State Medical University named after N.N. Burdenko, Voronezh, Russian Federation

^3^Predictive, Preventive and Personalised (3P) Medicine, Department of Radiation Oncology, University Hospital Bonn, Rheinische Friedrich-Wilhelms-Universität Bonn, Germany

*Corresponding author: Prof. M.A. Kovalevskaia, of Ophthalmology, Voronezh State Medical University named after N.N. Burdenko, Voronezh; Russian Federation; e.mail: ipkovalevskaya@gmail.com

**Keywords:** predictive preventive personalised medicine, clinical proteomics, differential omics, patient stratification, biomarker patterns, in-depth diagnostics, tailored therapy, liquid biopsy, tear fluid, risk assessment, cataract, oxidation, antioxidant defence, diabetes mellitus, ageing, peroxiredoxin, pirenoxin

**Introduction**

Diabetes mellitus (DM) is a complex metabolic disorder leading to a cascade of collateral pathologies, including cardiovascular and neurological diseases, cancer as well as diabetic retinopathy as the worldwide leading cause of blindness in humans. Owing to its high prevalence and comprehensive character of the pathology, 3P medicine based on the predictive diagnostics, targeted prevention and personalisation of treatment algorithms is considered to be the most optimal approach to DM management [1].

Visual impairments are characteristic for DM patients, whereby pathological processes linked to the DM-related cataract appear to be more complex compared to the ageing-related non-diabetic one. Accumulated evidence demonstrates the tear fluid analysis as a clinically relevant tool to predict disease development based on the molecular make-up providing the targets which can be further used for preventive measures and treatments tailored to the person [2]—the concepts known as 3P medical approach [3].

In contrast to the ageing-related non-diabetic cataract, the diabetes-related cataract is considered to result mainly from the antioxidant protection dysfunction. Indeed our previous investigations revealed peroxiredoxin 6 to be underrepresented in the tear fluid of patients affected by diabetic cataract [4, 5]. The current study is dedicated to comparative analysis of peroxiredoxin 6 in the tear fluid of treated versus untreated diabetic cataract compared to that of the ageing-related non-diabetic cataract subtype and healthy controls.

**Patients’ recruitment and examination**

Patients recruited for the study were 64.1 years old on average. Three groups were created:

- the first group was diabetes-free; all 50 patients were diagnosed with the ageing-related cataract; age in the group was 66.5 ± 4.3 years.
- the second group comprised 50 patients with diabetic history and DM-related cataract; age in the group was 59.4 ± 1.2 years.
- the control group comprised 25 healthy individuals; age in the group was 44.3 ± 2.4 years.

Ophthalmological examinations were carried out according to the generally accepted standard methodology [6].

**Tear fluid sampling and analysis**

Of the tear fluid, 0.1 ml per patient was taken without additional stimulation using a disposable sterile polymer cannula, which was placed in the lower part of the conjunctival sac. The tear fluid was frozen immediately and stored at −20 °C.

The tear protein composition was analysed using mass spectrometry as described elsewhere [7]. Mass spectra were obtained on a MALDI time-of-flight mass spectrometer Ultraflex II BRUKER (Germany) equipped with a UV (Nd) laser in the mode of positive ions using reflectron. Protein identification was carried out using the Mascot program (www.matrixscience.com).

The level of expression of PRDX6 was determined after a careful analysis of the data obtained by spectrophotometry, which presented the total expression level of antioxidant enzymes and the expression level attributable to all antioxidants except PRDX6. Peroxiredoxin 6 levels were additionally visualised and quantified using western blot analysis as described elsewhere [8].

**Statistical analysis**

For statistical processing of the research results, the STATISTICA 10.0 software package from StatSoftInc was used. A statistically significant difference is noted below as *p ≤ 0.05.

**Results interpretation**

Protein concentrations in the tear fluid are presented in Table 1 for each group of comparison.

**Table 1** The level of peroxyredoxin 6 expression in the tear fluid of group 1 patients before and after surgical treatment compared to the control group.

**Table:** 

Indicators (expression units)	Group 1 (*n* = 50)	Control group (*n* = 25)
Peroxyredoxin 6 before surgery	3.57* ± 0.35	2.74 ± 0.4
Peroxyredoxin 6 after surgical treatment	6.92* ± 0.2	2.74 ± 0.4

*р ≤ 0.05 – significantly higher than in the control group

Protein concentrations in the tear fluid are presented in Table 2 for each group of comparison.

**Table 2** The level of peroxyredoxin 6 expression in the tear fluid of group 2 patients before and after surgical treatment compared to the control group.

**Table:** 

Indicators (expression units)	Group 2 (*n* = 50)	Control group (*n* = 25)
Peroxiredoxin 6 to surgical treatment	1.12* ± 0.3	2.74 ± 0.4
Peroxiredoxin 6 with the use of Pirenoxine to surgical treatment	3.25* ± 0.5	2.74 ± 0.4
Peroxyredoxin 6 with Pyrenoxine after surgical treatment	4.07 ± 0.2	2.74 ± 0.4

*р ≤ 0.05 – significantly higher than in the control group

The level of expression of peroxiredoxin 6 in the tear fluid:

- healthy controls: 2.74 ± 0.4 activity nMol/mg/10
- group 1: before surgical treatment 3.57* ± 0.35; after surgical treatment 6.92 ± 0.2
- group 2: before surgical treatment 1.12* ± 0.3; with Pirenoxin before surgical treatment 3.25* ± 0.5; with Pirenoxin after surgical treatment 4.07 ± 0.2

*p ≤ 0.05 – significantly higher than in the control group.

**Case report**

- A 52-year-old patient with compensated DM type 2
- Complains of dryness and itching of the skin
- Ophthalmic complaints: complaints of lack of vision of the right eye; visual acuity of the right eye is 0.01; visual acuity of the left eye is 0.7 n/a.
- Diagnosis: OD – Complete Diabetic Cataract; OS – incomplete diabetic cataract, non-proliferative diabetic angioretinopathy

The tear protein spectrum was determined for the patient: the average tear protein concentration was 7.85 μg/ml; active peroxyredoxin 6 (14 kDa) is not detectable, see Fig. 1.
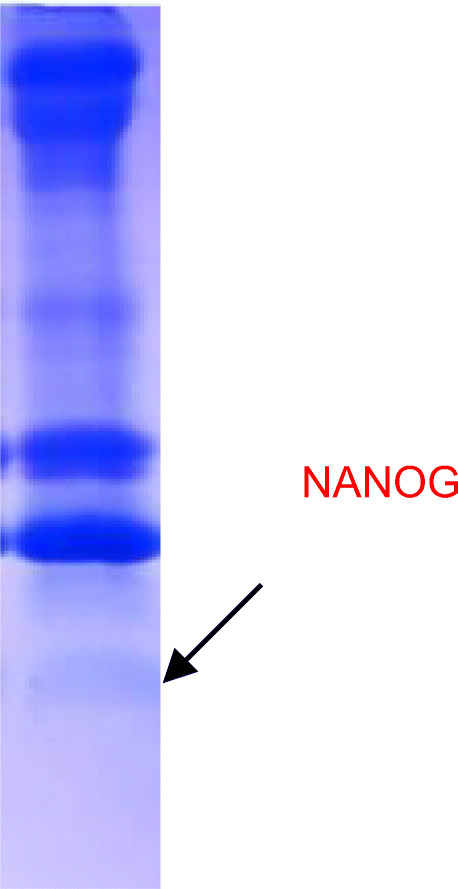


**Fig. 1** Western blot analysis of the tear fluid proteins: peroxyredoxin 6 is undetectable before surgical treatment

The patient was treated with pirenoxin to stabilize the clouding of the lens and to increase the antioxidant protection, see Fig. 2. The average concentration of tear proteins was 7.35 μg/ml, active peroxyredoxin 6, 14 kilodaltons – 4.01; an increase in the activity of peroxyredoxin 6 was recorded.
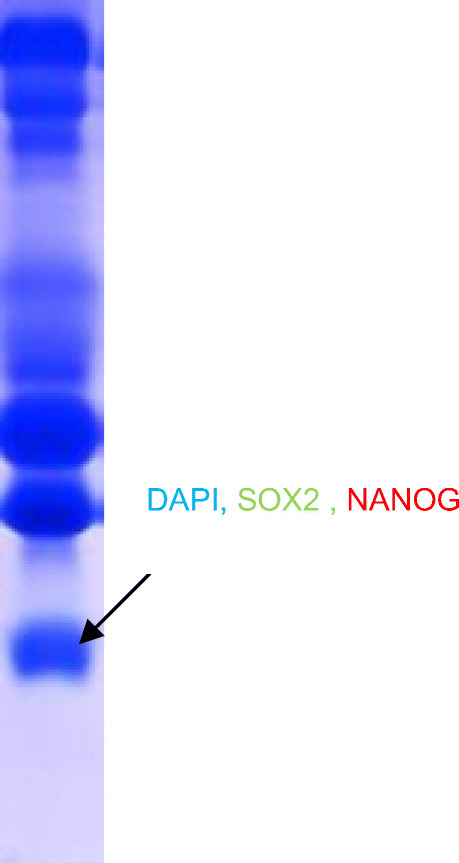


**Fig. 2** Western blot analysis of the tear fluid proteins after pirenoxine treatment: peroxyredoxin 6 (14 kDa) is wellbdetectable

**Conclusions and expert recommendations**

The study demonstrated significant qualitative and quantitative differences in protein profiles between non-diabetic ageing-related and diabetic cataract. Furthermore, the antioxidant protection in eyes of diabetic patients is evidently suppressed as demonstrated for the peroxiredoxin system which is a useful target for both predictive diagnostic analysis and preventive measures to restore the antioxidant defence and protect lens against oxidative cloudiness. Recommended treatment with pirenoxin appeared to be beneficial to compensate diabetes-related antioxidant deficits. Personalised algorithms might be useful to optimize treatments before and after cataract surgery. Further molecular biological analysis of the tear fluid in both ageing-related and diabetic conditions is highly recommended to investigate potential targets for a predictive and preventive approach.

**References**

1. Duarte AA, Mohsin S, Golubnitschaja O. Diabetes care in figures: current pitfalls and future scenario. EPMA J. 2018;9(2). 10.1007/s13167-018-0133-y.
2. Gerner C, Costigliola V, Golubnitschaja O. Multiomic patterns in body fluids: technological challenge with a great potential to implement the advanced paradigm of 3P medicine. Mass Spectrom Rev. 2019. 10.1002/mas.
3. Golubnitschaja O, Baban B, Boniolo G, Wang W, Bubnov R, Kapalla M, Krapfenbauer K, Mozaffari M, Costigliola V. Medicine in the early twenty-first century: paradigm and anticipation – EPMA position paper 2016. EPMA J. 2016;7:23, 10.1186/s13167-016-0072-4.
4. Zemskov AM, Kovalevskaya MA, Goncharova OV, Filina LA, Veprintsev N. Influence of oxidative stress on the postoperative period of diabetic cataract surgery. Russ J Immunol. 2017;11 (20):324–330.
5. Kovalevskaya MA, Filina LA. Approaches to predicting and preventing complications of cataract surgery of various types. Bull Exp Clin Surg. 2017; 10(3):246–252.
6. Kovalevskaya MA, Filina LA, Kokorev VL. Factors of the risk of developing a secondary cataract and recommendations for conducting a primary posterior capsulorhexis. J Exp Clin Surg. 2018;11(3):213–217.
7. Sharapov MG, Novoselov VI, Penkov NV, Fesenko EE, Vedunova MV, Bruskov VI, Gudkov SV. Protective and adaptogenic role of peroxiredoxin 2 (Prx2) in neutralization of oxidative stress induced by ionizing radiation. Free Radical Biol Med. 2019;134:76–86.
8. Goncharov RG, Rogov KA, Temnov AA, Novoselov VI, Sharapov MG. Protective role of exogenous recombinant peroxiredoxin 6 under ischemia-reperfusion injury of kidney. Cell Tissue Res. 2019:378(2):319–332.

**Reduced retinal microcirculation may predict glaucoma progression**

Kurysheva NI*

*Corresponding author: The Ophthalmological Center of the Federal Medical and Biological Agency of the Russian Federation, A.I. Burnazyan Federal Medical and Biophysical Center of FMBA, Gamalei St., Moscow, 123098, Russian Federation; e.mail: e-natalia@list.ru.

**Keywords**: predictive preventive personalized medicine, POAG, glaucoma progression, risk assessment, prognosis, predictors of progression, ocular blood flow, optical coherence tomography, OCT-angiography, personalized treatment, perfusion, GPA, retinal ganglion cells, vessel density, parafovea and peripapillary retina, intraocular pressure, ocular perfusion pressure, corneal hysteresis, age, glaucoma monitoring

**Background**

To determine the predictors of primary open angle glaucoma (POAG) progression successful monitoring and a personalized treatment approach. This postulate is an integral principle of predictive preventive personalized medicine (PPPM) that is to predict a disease development/progression and targetly prevent it by providing a treatment tailored to the person.

Increased intraocular pressure (IOP) and its fluctuations, thin cornea, low corneal hysteresis, optic disc hemorrhages, peripapillary atrophy of the choroid, age of patients, female sex, pseudoexfoliations, late detection of glaucoma, and arterial hypotension or hypertension are commonly considered to be the main recognized factors of POAG progression. Though systemic and local vascular factors in regard to glaucoma progression have been emphasized in literature [1–3], the predictive role of retinal microcirculation is not elucidated.

Optical coherence tomography angiography (OCTA) is a new non-invasive method for examination of the microcirculation of retina, optic nerve and choroid—the key structures affected by glaucoma [4].

The purpose of the present work is to study the predictive ability of peripapillary and inner macula microcirculation in glaucoma progression.

**Materials and methods**

In the prospective and observational study, 85 patients (85 eyes) were followed-up for 2 years and were divided into two groups on the basis of the presence/absence of progression detected using the Guided Progression Analysis (GPA) software on the Humphrey Field Analyzer II.

The study was approved by the Ethical Committee (Institutional Review Board) of the Institution of Federal Medical and Biological Agency of Russia and was conducted in accordance with Good Clinical Practice within the tenets of the Declaration of Helsinki (Protocol N 5, 05.02, 2016).

At follow-up visits with a 4-month interval, all the patients underwent complete ophthalmologic examinations and several imaging and functional tests [4]. The vessel density (VD) of the parafoveal superficial plexus and in whole image (wi) en face VD in the disc/peripapillary region were assessed using optical coherence tomography-angiography. Retinal nerve fiber layer (RNFL) and ganglion cells complex and the inner macular thickness were measured by SD-OCT, IOP, and corneal hysteresis using the Ocular Response Analyzer.

The inclusion and excludion criteria have been detailed by Kurysheva et al. [5].

Mean ocular perfusion pressure (MOPP) was calculated using the formula: MOPP = (2/3 diastolic blood pressure + 1/3 systolic blood pressure) × 2/3 –IOP.

**Statistical processing**

Mann–Whitney U test using Rosner–Glynn–Lee method and Pearson chi-squared test were applied to compare two independent groups by one characteristic.

The area under receiver operating characteristic curve (AUC) as a parameter’s importance measure for distinguishing the progression from non-progression eyes was accessed.

Cut-off value was determined by means of Youden’s index. The numerical data are represented as the mean ± SD. Statistical processing of the obtained results was carried out using the standard package of statistical analysis software “SPSS 16.0 for Windows.” Parameters with *p* < 0.05 were considered statistically significant.

**Results**

The clinical variables with statistically significant AUC for distinguishing the eyes with glaucoma progression and non-progression eyes are represented in Table 1. According to this data, wiVD disc and parafovea vessel density in superficial plexus had the high AUC that was comparable with AUC for peak follow-up IOP, MOPP, baseline corneal hysteresis, and structural parameters measured by SD-OCT.

**Table 1** Diagnostic ability of studied clinical parameters in differentiating the POAG progression and non-progression groups and their cut-off scores

**Table:** 

Variables	AUC ± S.E. 95% CI	*p*	Criterion (cut-off)
Peak follow-up IOP, mm Hg	0.792 ± 0.05 (0.677 0.880)	0.000	> 23.8
Corneal hysteresis, mm Hg	0.755 ± 0.07 (0.606 0.870)	0.000	<=9.6
OCT thickness ILM-IPL inferior- Hemi, μm	0.736 ± 0.07 (0.589 0.853)	0.001	<=105
wiVD disc, %	0.715 ± 0.07 (0.566 0.865)	0.001	<=45.2
Parafovea vessel density superficial	0.707 ± 0.07 (0.558 0.829)	0.005	<=45
Age, years along with	0.710 ± 0.07(0.588 0.813)	0.001	>70
Avg, RNFL, μm	0.692 ± 0.06 (0.567 0.799)	0.002	<=95.7
MOPP, mm Hg	0.682 ± 0.08 (0.521 0.819)	0.030	<=40

Abbreviations: AUC – area under ROC curve; CI – confidence interval, S.E. – standard error; OCT – optical coherence tomography, ILM – internal limiting membrane, IPL – inner plexiform layer

A positive correlation was observed between the thickness of the retinal inner layers in parafovea and the parafovea vessel density in the superficial layer (r = 0.4, *p* = 0.01).

**Conclusion and expected impact**

To the best of our knowledge, this is the first study where the predictive ability of OCTA parameters in glaucoma progression was comparable with well-known clinical predictors such as IOP, corneal hysteresis, and RNFL and OCT thickness ILM-IPL in inferior hemisphere. There is a growing body of evidence that early glaucomatous damage involves the macula, especially its inferior hemisphere [6]. The insufficient blood supply to ganglion cells in macula occurs in early glaucoma [7]. This is a reason to look more closely at the macula VD in glaucoma monitoring in order to *predict* and to detect glaucoma progression earlier that corresponds to the first principle of PPPM.

Identification of the progression predictors of any disease allows determining a patient’s individual profile. This is required in accordance with the concept of PPPM [8].

In fact, when a disease is diagnosed for the first time, prediction of its progression allows optimizing the treatment methods in each particular case and making the treatment personalized. In glaucoma it means the optimized prescribed method, for example, an earlier transition to laser or even surgical treatment, that may *prevent* further deterioration of visual function; in agreement with the second principle of PPPM. Even in the case of disease progression, the identification of individual markers of this progression will allow choosing the *personalised* treatment aimed specifically at these factors, for example, at reduction of ocular perfusion pressure; in line with the third principle of PPPM.

In conclusion, the present study has revealed new highly reliable predictors of glaucoma progression that allow significantly expanding the boundaries of generally accepted standards for glaucoma monitoring.

**References**

1. Binggeli T, Schoetzau A, Konieczka K. In glaucoma patients, low blood pressure is accompanied by vascular dysregulation. EPMA J. 2018;9(4):387–391. 10.1007/s13167-018-0155-5
2. Shiga Y, Aizawa N, Tsuda S, et al. Preperimetric Glaucoma Prospective Study (PPGPS): predicting visual field progression with basal optic nerve head blood flow in normotensive PPG eyes. Trans Vis Sci Tech. 2018;7(1):11. 10.1167/tvst.7.1.11
3. Kurysheva NI, Ryabova TY, Shlapak VN. Heart rate variability: the comparison between high tension and normal tension glaucoma. EPMA J. 2018;9(1):35–45. 10.1007/s13167-017-0124-4
4. Huang D, Liu L, You Q. OCTA: a new tool for glaucoma evaluation. Ophthalmol Manage. June, 2018;22:22–24.
5. Kurysheva NI, Maslova EV, Zolnikova IV, et al. A comparative study of structural, functional and circulatory parameters in glaucoma diagnostics. PLoS One. 2018;13(8):e0201599. 10.1371/journal.pone.0201599
6. Hood DC. Improving our understanding, and detection, of glaucomatous damage: An approach based upon optical coherence tomography (OCT). Prog Retin Eye Res. 2017;57:46–75. 10.1016/j.preteyeres.2016.12.002
7. Kurysheva NI. Macula in Glaucoma: Vascularity Evaluated by OCT Angiography. Res J Pharm Biolo Chem Sci. 2016;7(5):651–62.
8. Golubnitschaja O, Baban B, Boniolo G, et al. Medicine in the early twenty-first century: paradigm and anticipation - EPMA position paper 2016. EPMA J. 2016;7:23. 10.1186/s13167-016-0072-4

**Modern aspects of personalization and optimization of treatment and prophylactic measures for managing patients in the early postoperative period after complicated cataract surgery**

Zilov VG^1^, Smekalkina LV*^1^, Akulov SN^2^, Shurygina IP^3^

^1^Sechenov First Moscow State Medical University (Sechenov University)

^2^Ophthalmology Department, Rostov Regional Clinical Hospital

^3^ Department of Ophthalmology, Rostov State Medical University

*Corresponding author: Prof. Dr. Larisa Smekalkina, 119991 Trubetskaya 8-235, Moscow, Russia; e.mail: smekalkinal@bk.ru

**Keywords:** predictive preventive personalized medicine, cataract surgery, complications, early postoperative period, myopia, optical coherence tomography, total area average, retina

**Introduction**

According to WHO data there are currently 39 million blind people worldwide, and cataract was found as the cause of blindness in 51%. Most patients with combined pathology of the organ of vision (cataract and myopia) have complications in the early postoperative period [1,2]. The first place among such complications after cataract surgery belongs to neurodegenerative changes in the retina [3]. A dynamic personalized diagnostic approach to patients with complicated cataract for the prevention of complications in the early postoperative period in order to improve the quality of vision and quality of life of this contingent was determined in the present study.

**Materials and methods**

The study analyzed the examination of a clinical group of 100 patients (100 eyes) with a diagnosis of complicated cataract and myopia. All patients underwent cataract phacoemulsification (FEC) with implantation of an intraocular lens (IOL). Among them were 69 men and 31 women, with the average age of 58.5 years. For comparison, a control group of patients was selected (100 patients, 100 eyes). These patients were diagnosed with cataracts without combined ophthalmopathology. All patients in this group also underwent FEC with IOL implantation. The control group consisted of 58 men and 42 women, with the average age of 60.6 years. Inclusion criteria: 2 degree of lens opacity and visual acuity reduction up to 20/40.

All patients of both groups underwent a morphometric study of the retina before and on the second day after cataract surgery. The diagnostic study was performed on a Cirrus HD-OCT optical coherent tomograph (Carl Zeiss Meditec, Inc., USA) using Macula 3D for macula disease/single analysis protocols. To identify neurodegenerative changes in the retina, all patients underwent a qualitative retinal analysis and a quantitative assessment of the average retinal thickness (total area average, TAA) before and on the second day after cataract surgery.

**Results**

An analysis of the protocols of OCT scans showed that in the main clinical group, 62 patients (62%) on the second day after surgery showed an increase in TAA by an average of 25% from the initial parameters, in 31 patients by 10%, respectively, and in 7 patients (7%) the TAA value remained unchanged. In the control group, in most cases (84%), the TAA value on the second day after the operation did not change.

Visometry (determination of visual acuity with optical correction) on the 2nd day after the operation showed that, in the main group of 68 patients (68%), the maximum corrected visual acuity (MCP) was 20/30, in 20 patients (20%) –20/25 respectively, and in 12 patients (12%), MKOZ was 20/20. On the contrary, in the control group, in 85 patients (85%), MKOZ became 20/20, in 10 patients (10%) it was reduced to 20/25, and in 5 patients (5%) to 20/30, respectively.

In patients of both groups, the appearance of neurodegenerative changes in the retina in the form of the degree of thickening of the retina on the second day after the operation correlated with the visual result of the operation (MCH). However, the clinical efficacy of cataract surgery in groups was different. The worst visual effect on the second day after the operation was noted in the main group and was due to the appearance of neurodegenerative processes in the retina, confirmed by OCT data. Therefore, in each case, depending on the parameters of TAA, patients were offered an individual treatment regimen in the early postoperative period, which prevented retinal complications in the late postoperative period and obtained a high visual result on the 5–7th day after the operation.

**Conclusions**

Optical coherent tomography of the retina is considered to be a modern promising direction associated with predictive preventive personalized medicine in complicated cataract surgery [4]. It is advisable to include for each patient with combined ophthalmopathology (cataract and myopia) an innovative diagnostic technique—optical coherence tomography (OCT) with an assessment of the TAA parameter before surgery and on the second day after cataract surgery. The conducted targeted diagnosis contributes to suggest early detection of the initial neurodegenerative processes in the retina [5]. Up to date prevention of retinal complications in the early postoperative period of complicated cataract surgery will improve the quality of vision in particular and, accordingly, the quality of life of patients as a whole.

**References**

1. Malyugin B, Martsinkevich A. Modern approaches to the prevention of postoperative inflammatory complications in cataract surgery in patients with diabetes mellitus. Fyodorov J Ophthal Surg. 2016;1: 85–90. 10.25276/0235-4160-2016-1-85-90
2. Jabbarvand M, Hashemian H, Khodaparast M. Endophthalmitis occurring after cataract surgery: outcomes of more than 480,000 cataract surgeries. Epidemiologic features and risk factors. Ophthalmology. 2016;123(2):295–301. 10.1016/j.ophtha.2015.08.08.023
3. Egorova E, Obukhova O, Gorbenko O, et al. The effect of primary posterior capsulorexis on the activity of the local inflammatory process during phacoemulsification of complicated cataracts against the background of pseudoexfoliation syndrome. Siberian Sci Med J. 2018;38(1):53–58. 10.15372/SSMJ20180108
4. Bashina N, Frolov M, Lipatov D. Prevention of macular edema after cataract surgery in patients with diabetes mellitus. Diabetes mellitus. 2017;20(5):350–355. 10.14341/DM8729.
5. Sabel B, Wang J, Cárdenas-Morales L, et al. Mental stress as consequence and cause of vision loss: the dawn of psychosomatic ophthalmology for preventive and personalized medicine. EPMA J. 2018;9(2):133–160. 10.1007/s13167-018-0136-8

## 3PM in neurological, neurodegenerative and neuropsychiatric disorders

**Flammer syndrome and autoimmune inflammatory conditions of the central nervous system: multifactorial interrelations**

Paul F*

*Corresponding author: NeuroCure Clinical Research Center and Experimental and Clinical Research Center, Max Delbrueck Center for Molecular Medicine and Charité Universitaetsmedizin Berlin, Charitéplatz 1, D-10117 Berlin, Germany; e.mail: friedemann.paul@charite.de

**Keywords** Multiple sclerosis, Flammer syndrome, neuroinflammation, autoimmunity, demyelination, neurodegeneration, vasculature, perfusion, vascular dysregulation, predictive preventive personalized medicine (3PM)

**Multiple sclerosis (MS)**

Epidemiology and symptomatology

Multiple sclerosis (MS) is the most common chronic autoimmune CNS disease in young and predominantly female adults [1]. People with MS suffer from debilitating symptoms such as poor vision, impairment of motor functions and ambulation, sensory disturbances, bowel and bladder problems, but also “covert” symptoms such as depression, fatigue, cognitive dysfunction, pain, and poor sleep [2–4]. Most patients experience onset of the disease with a relapsing course (RMS), characterized by attacks of neurological dysfunction lasting from several days to weeks. Many patients advance to a secondary progressive disease course (SPMS) with or without relapses with longer disease duration. Up to 15% of patients exhibit a primary progressive disease course (PPMS) with insidious progression of neurological deficits from disease onset.

Etiology and risk factors of MS

Although the cause of MS is not known, individual disease risk is influenced by both genetic and to a larger extent environmental factors, the most important of which are Epstein–Barr virus (EBV) infection, latitude (higher risk with increasing latitude), vitamin D deficiency (associated with sunlight exposure and thus the latitude gradient), obesity, and smoking [5].

Neuropathological features

MS is neuropathologically defined by disseminated CNS white matter demyelinating lesions (“plaques”), a large proportion of which is located around small cerebral veins [6, 7], suggesting that the vasculature may be involved in lesion formation. However, advanced imaging and neuropathological studies have demonstrated that tissue damage is not limited to the white matter. Demyelination and lesions of both the cortex and the deep gray matter (DGM) are frequently detectable in MS and significantly contribute to progression of disability and cognitive deterioration. In general, progressive brain volume loss in untreated MS patients is about 1.5 to 2-fold faster than the physiological brain volume decrease in healthy aging.

The optic nerve and the retina as part of the CNS also show frequent involvement in MS. Orbital MRI can visualize optic nerve lesions in clinically overt optic neuritis (ON), and retinal axons and ganglion cells degenerate after an ON attack, which can be quantified by optical coherence tomography [8–10]. However, thinning of the retinal nerve fiber and ganglion cell layer may also occur without optic neuritis, pointing to subclinical optic nerve involvement or retrograde transsynaptic degeneration within the visual pathway [11]. Loss of retinal tissue is already measurable early over the course of MS and correlates with poor visual function and reduced visual quality of life.

The spinal cord is also frequently affected by demyelination and axonal damage, which can result in substantial clinical disability, such as walking difficulties and bladder problems, and may lead to cord atrophy. Spinal cord atrophy may also be caused by degeneration of tracts elsewhere in the CNS and could indicate the inception of the progressive phase of MS.

Immunopathogenesis of MS

MS immunopathogenesis has not been fully clarified. It is however generally accepted that the disease is a consequence of a derailed immune system and that autoreactive T cells, B cells, and the innate immune system are involved in immunopathogenesis [12]. The influx of activated immune cells into the CNS is enabled by a leaky blood–brain barrier with increased vascular permeability and precipitates demyelination in experimental autoimmune encephalomayelitis (EAE) and presumably also in human MS. The interaction of CNS-invading activated immune cells with glial cells (oligodendrocytes, astrocytes, and microglia) and neurons and axons initiates and perpetuates tissue damage.

Diagnosis of MS

An MS diagnosis is typically established when neurological symptoms are paralleled by characteristic inflammatory white matter lesions on brain and spinal cord MRI, and when the cerebrospinal fluid displays increased intrathecal immunoglobulin synthesis or oligoclonal bands suggestive of an immune reaction in the CNS. Diagnostic criteria (the so-called McDonald criteria) were established to enable MS diagnosis as early as possible in the context of a typical clinical presentation [13]. Exclusion of relevant differential diagnosis is paramount given there is no clinical or paraclinical feature unique to MS, which is reflected by the high number of false diagnoses. Important differential diagnoses to be considered are migraine, vascular diseases/comorbidities, neuromyelitis optica spectrum disorders, rheumatologic diseases, Susac syndrome, and other less prevalent diseases (for details, see [14]).

Treatment of MS

Several immunotherapies for the treatment of MS have been developed over the past decades [15]. These drugs are predominantly approved for RMS and reduced the attack frequency by an order of magnitude of between 30 and 70% compared to placebo or active comparator in phase III clinical trials. Some of these medications may slow down progression of disability. Post authorization studies posit that these immunotherapies have resulted in a better prognosis of MS and that time to reduced walking distance or wheelchair dependency can be prolonged. In addition, most drugs reduce the number of new white matter lesions, and some medications may slow down progression of brain atrophy. The approved drugs failed to clearly demonstrate neuroprotective properties, which underscores the urgent need for regenerative therapies [16]. Several attempts to target potentially modifiable environmental factors in MS (for example, diet, obesity, smoking, vitamin D supplementation) have yielded inconclusive results or remain underway [17–19]. Besides efforts to target the dysregulated immune system and hereby reduce relapse rates and disability progression, therapeutic efforts to alleviate burdensome and disabling fatigue, depression, sleep disorders, cognitive dysfunction, and pain are equally important, although evidence-based treatment recommendations on “symptomatic therapy” are scant [20].

How could and MS and FS be interrelated?

Brain and retinal vasculature are involved in MS, and studies of cerebral perfusion in MS have occasionally been performed. Decreased cerebral blood flow has been found in both the gray and white matter of MS patients as well as in the so-called “normal-appearing white matter” (NAWM) [21]. Interestingly, one study reported decreased CBF in the NAWM of patients with CIS, and another study found reduced venous density in the periventricular areas of patients with short disease duration [22]. This suggests altered perfusion to be a phenomenon occurring in early disease, although the underpinnings of cerebral hypoperfusion in MS are incompletely understood. Initially, cerebral hypoperfusion was believed to occur subsequent to axonal and neuronal degeneration, reflected by brain volume loss and resulting from reduced metabolic demand. However, this hypothesis has recently been questioned, while an alternative directionality is also conceivable. In MS, impaired astrocytic energy production might contribute to diminished arteriolar vasodilation through lower K+ recovery in the perivascular space. Preliminary OCT angiography and functional retinal imaging studies reported reduced arteriolar and venular retinal blood flow velocities in MS, and rarefaction of retinal vessels in eyes with optic neuritis. Interestingly, rigidity of retinal vessels is a common observation in both MS and FS. Another commonality of these conditions are increased concentrations of the vasoconstrictive endothelin-1.

Do MS and Flammer syndrome coincide?

Association between FS [23] and MS has been investigated [24]. A questionnaire with 15 typical symptoms and signs of FS was given to 58 people with MS who were hospitalized in a Rehabilitation Centre and to 259 control subjects in shopping malls. MS patients were on average 44.7 years old and had relatively high neurological disability (above 5 on the Expanded Disability Status Scale). MS patients reported six of the 15 FS signs and symptoms significantly more often than controls: dizziness, low body mass index, cold hand/feet, tendency toward perfectionism, reduced thirst, feeling cold. Additional symptoms (tinnitus, headaches, increased pain sensation, long sleep-onset time, migraines increased response to certain drugs, low blood pressure) also occurred more frequently in MS than in controls, although not significantly. Some of these symptoms are known to occur in MS and can be related to the polyfocal affection of multiple areas of the brain and spinal cord. The study is limited by a highly selected MS cohort (inpatients with advanced disease). To further substantiate a possible association of FS and MS, newly diagnosed MS patients in the CIS or early RRMS stage will have to be investigated. Moreover, causality of the reported co-occurrence in either direction cannot be inferred. If FS increases the risk of MS through vascular dysregulation or if MS causes FS-like symptoms and signs through secondary vascular dysregulation (see above) remains to be clarified in subsequent studies.

**Susac syndrome (SuS)**

Susac syndrome is a very rare (several hundred published cases), T cell mediated CNS disorder affecting small arteries and arterioles of the brain, retina, and inner ear, hereby causing a clinical triad of visual disturbances, hearing deficits, and encephalopathy. Abnormal neurologic symptoms and findings such as vertigo, ataxia, gait abnormalities, upper motor neuron signs, and sensory disturbances are found only in a subset of patients. Diagnostic procedures include CSF analysis (with typically elevated protein but rarely oligoclonal bands), MRI (supratentorial white matter lesions, affection of the corpus callosum (CC) with “snowball lesions” in acute stage and pronounced atrophy of the CC in chronic stages), and fluorescein angiography (FAG) and OCT to detect retinal affection (branch retinal artery occlusion and arterial wall hyperfluorescence). Establishment of SuS diagnosis may be delayed because the clinical triad is rarely complete at onset [25]. SuS is amenable to treatment with high dose methylprednisolone and eventually plasmapheresis in acute phases and prednisolone, intravenous immunoglobulins, azathioprine, methotrexate, rituximab, and others as maintenance therapy, while many MS immunomodulators seem to be inefficacious or even harmful. Some authors propose the use of antiplatelet or anticoagulant therapy to reduce the risk of thrombosis in small arterioles. Flammer and colleagues reported increased ET-1 levels in SuS and proposed that patients with primary vascular dysregulation/FS have an increased risk for SuS. However, as Flammer et al. emphasize that SuS through its involvement of microvessels may also cause secondary vascular dysregulation, and rightly so call for further studies that help clarify the presumed causality of the association between SuS and vascular dysregulation and the temporal evolution of pathogenic events.

**Concluding remarks and expert recommendations**

FS and MS seem to exhibit some overlap in clinical presentation and presumably share pathophysiological commonalities. However, an increased risk of MS in subjects with FS and vice versa cannot be inferred due to the scarcity of the data. Studies on symptoms of FS in patients with newly diagnosed MS need to be carried out, and with regard to FS symptoms, these patients will have to be followed longitudinally over the course of the disease. Structural and functional changes of the cerebral and retinal vasculature should be investigated in people with MS with and without additional FS symptoms. These studies will hopefully help to clarify mutual pathogenetic and clinical interrelations and leverage predictive diagnostics for both conditions, thus identifying people at risk to develop an overlap syndrome. This is a prerequisite for targeted prevention that will be built upon better understood clinical phenotypes and biomarker studies, which will ultimately pave the way to a personalization of medical services for patients with neuroinflammatory disorders.

**References**

1. Borisow N, Döring A, Pfueller CF, Paul F, Dörr J, Hellwig K. Expert recommendations to personalization of medical approaches in treatment of multiple sclerosis: an overview of family planning and pregnancy.EPMA J. 2012;3(1):9. 10.1186/1878-5085-3-9.
2. Veauthier C, Hasselmann H, Gold SM, Paul F. The Berlin Treatment Algorithm: recommendations for tailored innovative therapeutic strategies for multiple sclerosis-related fatigue. EPMA J. 2016;7:25.eC
3. Urbanek C, Weinges-Evers N, Bellmann-Strobl J, et al. Attention network test reveals alerting network dysfunction in multiple sclerosis. Mult Scler. 2010;16:93–99.
4. Paul F. Pathology and MRI: exploring cognitive impairment in MS. Acta Neurol Scand. 2016;134 Suppl 200:24–33.
5. Koduah P, Paul F, Dörr JM. Vitamin D in the prevention, prediction and treatment of neurodegenerative and neuroinflammatory diseases. EPMA J. 2017;8(4):313–325. 10.1007/s13167-017-0120-8.
6. Sinnecker T, Kuchling J, Dusek P, Dörr J, Niendorf T, Paul F, et al. Ultrahigh field MRI in clinical neuroimmunology: a potential contribution to improved diagnostics and personalised disease management. EPMA J. 2015;6(1):16. 10.1186/s13167-015-0038-y.
7. Sinnecker T, Clarke MA, Meier D, et al. Evaluation of the central vein sign as a diagnostic imaging biomarker in multiple sclerosis. JAMA Neurol 2019; https://doi.org/10.1001/jamaneurol.2019.2478
8. Oberwahrenbrock T, Traber GL, Lukas S, Gabilondo I, Nolan R, Songster C, et al. Multicenter reliability of semiautomatic retinal layer segmentation using OCT. Neurol Neuroimmunol Neuroinflamm. 2018;5(3):e449. 10.1212/NXI.0000000000000449. eCollection 2018 May.
9. Zimmermann HG, Knier B, Oberwahrenbrock T, Behrens J, Pfuhl C, Aly L, Kaminski M, Hoshi MM, Specovius S, Giess RM, Scheel M, Mühlau M, Bellmann-Strobl J, Ruprecht K, Hemmer B, Korn T, Paul F, Brandt AU. Association of retinal ganglion cell layer thickness with future disease activity in patients with clinically isolated syndrome. JAMA Neurol. 2018;75(9):1071–1079. 10.1001/jamaneurol.2018.1011.
10. Ayadi N, Dörr J, Motamedi S, Gawlik K, Bellmann-Strobl J, Mikolajczak J, et al. Temporal visual resolution and disease severity in MS. Neurol Neuroimmunol Neuroinflamm. 2018;5(5):e492. 10.1212/NXI.0000000000000492.
11. Sinnecker T, Oberwahrenbrock T, Metz I, Zimmermann H, Pfueller CF, Harms L, et al. Optic radiation damage in multiple sclerosis is associated with visual dysfunction and retinal thinning--an ultrahigh-field MR pilot study. Eur Radiol 2015;25:122–31.
12. Dendrou CA, Fugger L, Friese MA. Immunopathology of multiple sclerosis. Nat Rev Immunol. 2015;15(9):545–58. doi: 10.1038/nri3871.
13. Thompson AJ, Banwell BL, Barkhof F, Carroll WM, Coetzee T, Comi G, et al. Diagnosis of multiple sclerosis: 2017 revisions of the McDonald criteria. Lancet Neurol. 2018;17(2):162–173. 10.1016/S1474-4422(17)30470-2.
14. Geraldes R, Ciccarelli O, Barkhof F, De Stefano N, Enzinger C, Filippi M, et al. MAGNIMS study group. The current role of MRI in differentiating multiple sclerosis from its imaging mimics. Nat Rev Neurol. 2018;14(4):199–213. 10.1038/nrneurol.2018.14. Erratum in: Nat Rev Neurol. 2018 Mar 20;14 (4):213.
15. Gehr S, Kaiser T, Kreutz R, Ludwig WD, Paul F. Suggestions for improving the design of clinical trials in multiple sclerosis-results of a systematic analysis of completed phase III trials. EPMA J 2019;10:425–436.
16. Starossom SC, Campo Garcia J, Woelfle T, et al. Chi3l3 induces oligodendrogenesis in an experimental model of autoimmune neuroinflammation. Nat Commun. 2019;10:217
17. Camu W, Lehert P, Pierrot-Deseilligny C, et al. Cholecalciferol in relapsing-remitting MS: a randomized clinical trials (CHOLINE). Neurol Neuroimmunol Neuroinflamm. 2019;6:e597.
18. Dörr J, Bäcker-Koduah P, Wernecke KD, et al. High-dose vitamin D supplementation in multiple sclerosis – results from the randomized EVIDIMS (efficacy of vitamin D supplementation in in multiple sclerosis) trial. Mult Scler J Exp Transl Clin 2020;6: 2055217320903474.
19. Brenton JN, Banwell B, Bergqvist AGC, et al. Pilot study of a ketogenic diet in relapsing-remitting MS. Neurol Neuroimmunol Neuroinflamm. 2019;6:e565.
20. Gaede G, Tiede M, Lorenz I, Brandt AU, Pfueller C, Dörr J, et al. Safety and preliminary efficacy of deep transcranial magnetic stimulation in MS-related fatigue. Neurol Neuroimmunol Neuroinflamm. 2017;5(1):e423. 10.1212/NXI.0000000000000423. eCollection 2018 Jan.
21. D’haeseleer M, Cambron M, Vanopdenbosch L, De Keyser J. Vascular aspects of multiple sclerosis. Lancet Neurol. 2011 Jul;10(7):657–66. 10.1016/S1474-4422(11)70105-3. Review.
22. Sinnecker T, Bozin I, Dörr J, Pfueller CF, Harms L, Niendorf T, et al. Periventricular venous density in multiple sclerosis is inversely associated with T2 lesion count: a 7 Tesla MRI study. Mult Scler. 2013 Mar;19(3):316–25. 10.1177/1352458512451941.
23. Flammer J, Konieczka K, Flammer AJ. The primary vascular dysregulation syndrome: implications for eye diseases. EPMA J. 2013 Jun 7;4(1):14. 10.1186/1878-5085-4-14.
24. Konieczka K, Koch S, Binggeli T, Schoetzau A, Kesselring J. Multiple sclerosis and primary vascular dysregulation (Flammer syndrome). EPMA J. 2016 Jun 15;7:13. 10.1186/s13167-016-0062-6. eColl
25. Kleffner I, Dörr J, Ringelstein M, Gross CC, Böckenfeld Y, Schwindt W, et al. European Susac Consortium (EuSaC). Diagnostic criteria for Susac syndrome. J Neurol Neurosurg Psychiatry. 2016 Dec;87(12):1287–1295. 10.1136/jnnp-2016-314,295.

**Possibilities of implementing the paradigm of predictive, preventive and personalized medicine into psychotherapeutic practice**

Malakhovskiy V^1^, Monasipova L*^1^, Lim V^1^

^1^The First Moscow State Medical University named after I.M. Sechenov (Sechenov University) Moscow, Russia

*Corresponding author: Liliya Monasipova, The First Moscow State Medical University named after I.M. Sechenov, Trubeckaya str., 8, Moscow, 119991; e.mail: monasipova-aria@mail.ru

**Keywords:** predictive preventive personalized medicine, personalized psychotherapy, targeted personalized treatment, prevent programs, Ayurveda, dominant representative system, anxiety disorders

**Introduction**

The high appealability of patients with anxiety disorders to private clinics, including repeated ones, indicates the need to look for effective treatment regimens.

Issues of effective correction of mental disorders based on a personalized approach remain insufficiently studied in theoretical and practical terms.

The system of prevention, diagnosis and treatment in Ayurveda is based on a constitutional approach. It can provide prediction, personalized treatment, and effective preventive programs.

The possibilities of using the potential of Ayurveda to develop personalized medicine ideas have been discussed by researchers [1–3].

Research confirms the relationship between the imbalance of doshas and the results of psychopathological Western questionnaires. Thus, for example, there is a connection between the imbalance of Vata and Pitta doshas and a high level of anxiety [4].

Several clinical studies focus on the effectiveness of a diet based on Ayurveda’s personalized approach in the treatment of anxiety disorders [5].

The aim of the study is to assess the potential of Ayurveda for the selection of personalized treatment regimens for anxiety disorders.

**Materials and methods**

Specialists at the Scientific and Practical Center for Traditional Medical Systems of the Sechenov University have developed a protocol for the diagnosis and treatment of anxiety disorders, based on a personalized approach of Ayurveda in combination with an assessment of the patient’s dominant representative system (auditory, visual, kinesthetic).

The study involved 43 patients with an established diagnosis of generalized anxiety disorder according to ICD 10.

The choice of a psychotherapeutic technique was determined by the outcome of the Prakriti and Vikriti questionnaires developed in the Scientific and Practical Center for Traditional Medical Systems and F. Puselik’s representative system questionnaire. The choice of diet was determined by the test results of the Vikriti questionnaire. The effectiveness of the intervention was evaluated on a Hamilton anxiety scale (HARS).

**Results**

According to the results of the Vikriti questionnaire, Vata dosha prevailed in 26 patients, and Pitta dosha prevailed in 17 patients.

In each of these groups, indicators of a representative system were evaluated. Among patients with Vikriti Vata, the auditory system predominated (15 patients), there were also 11 patient with the kinesthetic system. Among patients with Vikriti Pitta visuals prevailed (11 patients), there were also 6 patients with a predominant kinesthetic system.

The treatment algorithm was developed on the results of the Vikriti questionnaire and the dominant representative system.

Patients with a predominance of the Vata dosha and the auditory system had sessions of cognitive-behavioral therapy and existential psychotherapy, during which the cognitive component of anxiety was corrected. In addition, patients were trained in breathing techniques. In this group, the HARS results decreased from 25.3 ± 2.7 to 5.8 ± 0.21.

In work with patients with a predominance of the Vata dosha and kinesthetic system, the emphasis was on the somato-vegetative and anxiety components. The techniques of body-oriented psychotherapy, Jacobson’s progressive muscle relaxation were used. In this group, the HARS results decreased from 23.1 ± 2.7 to 8.2 ± 0.7.

A group of patients with a predominance of the Pitta dosha and the visual system underwent art therapy, patients with a predominance of the Pitta dosha and the kinesthetic system were trained in Jacobson’s progressive muscle relaxation. The somato-vegetative and behavioral components of anxiety were corrected using these methods. The level of anxiety decreased from 22.3 ± 2.4 to 8.5 ± 0.8 and from 24.4 ± 2.6 to 7.3 ± 0.6, respectively.

The most persistent result was observed in patients with an auditory representative system, which is consistent with selected methods of therapeutic exposure. A significant number of patients in this category maintained a stable therapeutic effect, despite the impact of stressful factors in the follow-up.

Additionally, dietary and life style recommendations were included in the treatment protocol; they were different for patients with a predominance of Vata and Pitt doshas.

The next stage, the follow-up study, was conducted 6 months after the intervention. None of the patients had clinically significant relapses.

**Conclusions**

1. Effective treatment of anxiety disorders requires approaches of predictive, personalized, preventive medicine (PPPM). Ayurveda’s constitutional approach can provide targeted treatment and prevention programs. Thus, when selecting methods of psychotherapy, we can take into account the dominant dosha. For example, it was noted that in patients with a predominance of the Vata dosha, the cognitive component of anxiety and respiratory disturbances were expressed, and therefore breathing exercises and cognitive therapy techniques were effective. In patients with a predominance of Pitta dosha, the behavioral component of anxiety, muscle tension prevailed, and progressive muscle relaxation training and behavioral techniques were effective.
2. The use of personalized secondary prevention programs, including diet and lifestyle recommendations, is important for the secondary prevention of anxiety disorders.
3. The study demonstrated the relationship between the Prakriti–Vikriti Ayurveda concept and the representative system. The identification of the auditory representative system as dominant can be considered as a predictor of effectiveness of psychotherapeutic intervention.

**Outlook**

A follow-up large-scale study is necessary to confirm the presented results or to optimize the results for individual patients.

**References**

1. Bhalerao S, Patwardhan K. Prakriti-based research: Good reporting practices. J Ayurveda Integr Med. 2016;7(1):69–72. 10.1016/j.jaim.2015.08.002
2. Safonicheva O. New international education project in predictive, preventive and personalized medicine. EPMA J. 2014;5(S1):20. 10.1186/1878-5085-5-S1-A20
3. Sarsina PR, et al. Traditional, complementary and alternative medical systems and their contribution to personalisation, prediction and prevention in medicine—person-centred medicine. EPMA J. 2012;3(1):15. 10.1186/1878-5085-3-15
4. Mills PJ et al. Relationships among classifications of ayurvedic medicine diagnostics for imbalances and western measures of psychological states: an exploratory study. J Ayurveda Integr Med. 2019;10(3):198–202. 10.1016/j.jaim.2018.02.001
5. Cheong MS. Effect of Ayurveda diet on anxiety disorder: narrative study. Asia Life Sci. 2018;15(2):1321–1329.

**Predictive, preventive and personalized approach in the field of neurogenic feet deformities**

Frolov V^1^, Akopyan M*^1^

^1^Federal State Autonomous Educational Institution of Higher Education I.M. Sechenov First Moscow State Medical University of the Ministry of Health of the Russian Federation (Sechenov University), Department of sports medicine and medical rehabilitation.

*Corresponding author: Prof. Dr. V. Frolov, I.M. Sechenov First Moscow State Medical University, Russian Federation; e.mail: vafrolovva@yandex.ru, marianna.akopian@yandex.ru

**Keywords:** Predictive preventive personalized medicine, mononeuropathy, piriformis syndrome (PS), meralgiaparaesthetica (Bernhardt–Roth syndrome), spina bifida, neurology, orthopedics, electroneuromyography, stabilometry, personalized approach, neurogenic feet deformities, pressure point

**Introduction** Strain redistribution and pressure points displacement [1] of the feet are important points for detecting and personalizing treatment of the neurogenic feet deformities. Predictive, preventive and personalized medicine predicts the disease development and prevents its progression at all stages of the disease.

Foot deformities can be caused by changes in the skeleton, muscle function disturbances [6], central and peripheral nervous system diseases, etc.

The comprehensive examination, including electroneuromyography (ENMG), stabilometry and functional scale of the lower limb (LEFS) allows identifing the foot disturbances.

The predictive, preventive and personalized algorithms allow choosing the most optimal and effective approach taking into account the individual characteristics of the patient [7].

**The foot pathology—an important neurogenic diagnostic criterion**

Feet deformities are often the result of central and peripheral nervous system diseases [4], such as Charcot–Marie–Tooth disease (CMT), spina bifida [3], compression neuropathies of the lower extremities [5], Parkinson’s disease, multifocal dystonia, multiple sclerosis (MS) and mononeuropathy.

**Mononeuropathy—the most common disease of the peripheral nervous system**

According to statistics, mononeuropathies are quite common and account for approximately 50% of all peripheral nervous system diseases and occupy 2nd place for the entire disability [2]. The study involves 39 patients with mononeuropathies of the sciatic nerve compression (PS), meralgia paraesthetica (Bernhardt–Roth syndrome), common peroneal and tibial nerve compressions.

**The personalized approach in the framework of feet deformities diagnosis**

The personalized approach was used for the primary diagnosis of neurogenic feet deformities. The obtained diagnostic data significantly enhanced the therapeutic effect and decreased further symptoms manifestation, including muscle strength and tone descension, muscle hypo- and atrophy, lower extremity joints and lumbar intervertebral discs overload. The diagnosis included electroneuromyography (ENMG), stabilometry and functional scale of the lower limb (LEFS). The feet deformities and impaired function presented in 55% of cases as paralytic (equinopolar, equinovarus) deformity, among which a hollow foot presented in 29% of cases. The results were directly dependent on the affected peripheral nerve and impaired muscles involved in the pathology. The paralytic feet deformities developed due to the muscle imbalance, leading to the foot points support displacement (calcaneus, distal phalanges of the 1st and 5th toes). The LEFS scale indicated a lower limb function depression on the affected side.

**The pathological stereotype of movement consequences**

The patients had changes in motor status, sensitivity and reflexes decrease on the damaged side. A number of patients, due to the early stage of the disease, had unmanifested neurogenic feet deformities. The impulse average values on ENMG were below normal, corresponding with the clinical diagnosis. Patients with moderate neurological deficit showed a gait decrease according to the foot contact time changes on the stabilometry. In addition, the load reduction on the calcaneus and distal phalanges of the 1st and 5th toes was detected.

**PPPM—an effective method of rehabilitation and prevention of foot deformities**

Predictive, preventive and personalized medicine enables us to diagnose and timely treat the compression symptoms, foot deformities, which prevents further manifestations of the disease, such as muscle hypo- and atrophy, joints overload with dystrophic changes development and intervertebral hernias development. The rehabilitation course includes manual therapy of the feet and lower legs; the production of individual orthopedic insoles, physical exercises affecting certain muscle groups and specific drug therapy.

**Conclusions** The strain redistribution and pressure points displacement assessment enables estimating the deformation foot degree, taking into account the disease symptoms severity in each patient. This algorithm fits into the foundation of PPPM. The muscle and neural status assessment helps to prevent further disease progression.

The treatment showed significant improvement of the impulse velocity rate of motor fibers by ENMG in 88% of cases, alignment of the foot points is supported by the stabilometry and improvement of the LEFS scale characteristics. Finally, PPPM is essential to improve individual outcomes at any stage and treat manifested symptoms of the disease.

**References**

1. Leonchuk SS, Evreinova YV, Sazonova NV. Modern referent lines and angles in diagnostic and treatment of foot and ankle pathology; Rejr. 2018;8(4):143–154. 10.21569/2222-7415-2018-8-4-143-154
2. Yevtushenko SK, Yevtushevskaia AN, Marusichenko VV. Tunnel neuropathies. Difficulties in the diagnosis and therapy. Int Neurol J. 2016. 10.22141/2224-0713.1.71.2015.78417
3. Gunay H, Sozbilen MC, Gurbuz Y, Altinisik M, Buyukata B. Incidence and type of foot deformities in patients with spina bifida according to level of lesion. Childs Nerv Syst. 2016;32(2):315–9. 10.1007/s00381-015-2944-7.
4. Mandel S, Willis J. Handbook of lower extremity neurology. Publication date 29 Oct 1999; ISBN10 0443075484; ISBN13 9780443075483.
5. Mozolevsky YV, Barinov AN. Combination treatment for tunnel neuropathies of the lower extremities. Neurol Neuropsychiatry, Psychosomat. 2013;(4):10–20. 10.14412/2074-2711-2013-2449
6. Stinus H, Weber F. Inserts for foot deformities. Orthopedist. 2005;34:776–781. 10.1007/s00132-005-0829-2
7. Golubnitschaja O, Baban B, Boniolo G, et al. Medicine in the early twenty-first century: paradigm and anticipation - EPMA position paper 2016. EPMA J. 2016;7:23; 10.1186/s13167-016-0072-4

**Risk-based screening strategies for personalized prevention of neurodegenerative diseases**

Safonicheva OG*^1^, Smekalkina LV^2^, Naprienko MV^3^

^1^Department of Sports Medicine and medical rehabilitation, Institute of Clinical Medicine, First Moscow State Medical University (I.M. Sechenov University), Russian Federation; ^2^¬^3^Department of Integrative Medicine, Institute of Post-graduate Education for Doctors, First Moscow State Medical University (I.M. Sechenov University), Russian Federation

*Corresponding author: Prof. Dr. O. Safonicheva, I.M. Sechenov First Moscow State Medical University, Russian Federation; e.mail: safonicheva.o@mail.ru

**Keywords**: preventive predictive personalized medicine (PPPM), neurodegenerative diseases, screening strategies, cerebral metabolism, new markers, risk factors, patients stratification.

**Introduction**

The increase in life expectancy of the European population has led to an increase in the burden of diseases occurring with cognitive deficits (dementia, Alzheimer’s Disease). Brain-related disorders are expected to affect at least one in three persons at the age of 75, costing approximately 800 billion euro every year. The cost is comparable to that of cardiovascular diseases, cancer and diabetes put together [1]. To date, there is no generally accepted classification of pre-dementia cognitive disorders (PDCDs): more commonly, the term “mild cognitive impairment” (MCI) is used for assessing the prodromal stage of neurodegenerative diseases to refer to the reversible disorders [2]. PDCOs are heterogeneous in the mechanisms of their occurrence, involving genetic predisposition but also a number of modifiable factors, the synergic combination of which potentiates the risks. Thus, the search for new markers for the reversible disorders at the stage of MCI, stratification of patients and development of personalized treatment approach are the main ideas of predictive preventive and personalized medicine (PPPM).

**The aim** of the pilot research was to develop risk-based screening strategy for MCI and study the interconnections between brain hypoxia, MCI and postural muscle stress.

**The study outlines**

The study included 118 patients (38 men and 80 women), average age 65 ± 4.6 years, with “office syndrome” - combined myofascial pain in the cervical part of the vertebral column, chronic fatigue syndrome (CFS) and MCI. Neuropsychological tests—Mini-Mental State Examination (MMSE) and Montreal cognitive scale (MoCA)—were used to detect the cognitive and emotional status. Neurological examination and magnetic resonance imaging conducted were to identify the role of the biomechanical disturbances in the cervical region of the vertebral column in affecting the state of cerebral blood flow. Genetic tests used for assessment of tendency to depression, obsessive-compulsive disorders, dementia, addictions (smoking, alcoholism, overeating), mood disorders, and anxiety. The neuro-energo-cartography (NEC) method of visualization was used to study cerebral metabolism and measure the degree of acidosis and hypoxia in the brain tissues.

**Results**

The main complaints in patients were divided into two groups: disorders in the cognitive, personal, emotional status, including headache, fatigue, memory, and sleep problems; somatic disorders, including persistent muscle pain, a sense of “stiffness”, frequent pain (thorax, cervical spine), various joint pain (without redness or swelling), and shortness of breath. Neuro-vertebral examination revealed biomechanical markers, including muscular discoordination, rigidity in the shoulder girdle, myotonic “tunnel-syndromes” in cervical spine, and postural displacement in all patients. Muscle spasms, the fascia, and cranial membranes kinetics restriction contributed to slowing down of the cerebral blood flow and obstruction of venous outflow. The NEC analysis revealed signs of functional inter-hemispheric asymmetry and different levels of cerebral acidosis as biomarkers for brain hypoxia.

The main treatment goals were creation of personalized integrative programs of rehabilitation for patients with different postural, neurovascular disturbances to remove the brain hypoxia and return them into “corridor of self-regulation”.

Complex therapy included cognitive and behavioral therapy; complementary therapies included connective tissue techniques, methods of osteopathy for spasmodic muscles releasing, and the neuro-vascular “tunnel syndromes” removal. Intermitted hypoxia therapy and medicines (neuro-protectors, vitamins, anti-oxidants) were used for patients with cerebral acidosis for normalization of the brain metabolism and reduction of oxidative stress. Coordination gymnastics was used for patients with postural problems. Recommendations to healthy lifestyle and working place with ergonomic equipment were given to all patients. A comprehensive personalized rehabilitation program improved patient’s clinical and emotional background, memory and cognitive functions. NEC examination marked the trend in recovery of inter-hemispheric connections and interactions; normalization of the bioelectrical activity in the brain, improvement of cerebral metabolism, and plasticity (Fig. 1a & b).
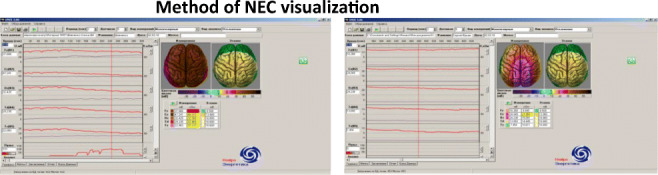


**Fig. 1** **a** Cerebral acidosis in a 45 y.o. patient. **b** Decreasing of cerebral acidosis after treatment in patient

**Conclusions and outlook**

In this pilot study, we compared the prognostic manifestations of postural muscle stress, cerebral metabolism, and mild cognitive impairment in patients. A new protocol for personalized profiling of working-aged individuals was developed. Neuropsychological, neuro-vertebral, and instrumental NEC express-visualization technologies for diagnosis of stress-induced conditions (disturbed body posture, neuro-vascular “tunnel syndromes”, cerebral acidosis) helped indicate the early stages of neurodegenerative diseases and include the patients with MCI into the risk group.

Conventional methods in PPPM programs (modification of life style, diet, physical activity) can decrease side-effects of drugs and motivate the patients to take personal responsibility of their own health.

Effective screening strategies may result in earlier disease detection, which provides possibilities for more effective treatments, to diminish the disease burden, and help transition the healthcare system to the preventive, predictive, and personalized medicine concept.

**References**

1. Golubnitschaja O, Costigliola V, EPMA. General report and recommendations in predictive, preventive and personalised medicine 2012: white paper of the European association for predictive, preventive and personalised medicine. EPMA J. 2012; 10.1186/1878-5085-3-14
2. Vasenina EE, Gutorova DA, Smirnova IM, Levin OS. Predementia cognitive disorders: modern approaches to terminology, diagnosis and treatment. Farmateka. 2018. 10.18565/harmateca.
3. Polivka J Jr., Polivka J, Pesta M, Rohan V, Celedova L, Mahajani S, Topolcan O, Golubnitschaja O. Risks associated with the stroke predisposition at young age: facts and hypotheses in light of individualized predictive and preventive approach. EPMA J. 2019;10(1):81–99. 10.1007/s13167-019-00162-5.
4. Safonicheva OG, Martynchik SA. Comprehensive screening program on the platform “Neurology” on the basis of personalized medicine innovative technologies. Int J Appl Fund Res. 2015;2(2):246–250.

**Piriformis muscle syndrome (PMS): diagnosis and treatment in the framework of predictive, preventive, and personalized medicine**

Frolov VA*, Akopyan MS

*Corresponding author

 Federal State Autonomous Educational Institution of Higher Education I.M. Sechenov First Moscow State Medical University of the Ministry of Health of the Russian Federation (Sechenov University), Department of sports medicine and medical rehabilitation

e.mail: vafrolovva@yandex.ru, marianna.akopian@yandex.ru.

**Keywords:** Predictive preventive personalized medicine, piriformis muscle, myofascial syndrome, biomechanical dysfunction, neurology medicine, orthopedic medicine, surgery

**Introduction** The piriformis muscle syndrome (PSM) patients’ treatment is difficult due to the late stage of diagnosis [2] and misjudgment of the magnitude of the disease. Piriformis syndrome is accompanied by biomechanical dysfunctions at the pelvis and lower extremities and tension of the myofascial components of the musculoskeletal system around the affected area. Predictive, preventive and personalized medicine includes exact diagnosis and timely treatment of the disease and is effective in the later stage.

**Risk factors can be fixed**

Piriformis syndrome occurs quite often in the population. According to statistics, 6% of all patients presenting with low back pain had PS [2]. Several risk factors cause the disease such as anatomic variation of sciatic nerve, overloading the piriformis muscle with prolonged walking, running or prolonged sitting [6].

**PS is frequently diagnosed at later stages**

Usually, PS is confirmed in the late stages of the disease, accompanied with the obvious symptoms, including intensive pain in the gluteal region, while siting more than 10–15 min, pain irradiation along the sciatic nerve and impaired gait. Periodic examination could promote early prevention of the disease in the population.

**Early diagnoses play a key role in PS manifestation**

The physiotherapist’s knowledge of myofascial syndrome affecting the piriformis may help in early diagnosis and treatment [1]. In addition, PS can be detected with the aid of MRI and rheovasography (RVG) [2]. The study involves 60 patients, with periodic non-intensive myofascial pain in the gluteal area, aged from 30 to 40. All participants passed MRI, stabilometry, and rheovasography (RVG) of the lower extremities. The MRI showed the type B anatomic variation of the sciatic nerve [4] in 42% and pyriformis muscle changes in 86%. In 95% of cases, there were changes in stabilometry, including displacement of the pressure center [5] with a twisted pelvis, and 82% of patients had changes in RVG, including blood flow disturbance (spasm of arterioles and expansion of venules) [3]. In 9% of cases, there were no changes in the piriformis muscle and the sciatic nerve.

**Short rehabilitation period**

The remaining patients underwent the early rehabilitation course, including pharmacotherapy, physiotherapy, and acupuncture within 1.5–2 weeks. The effectiveness of rehabilitation was evaluated with MRI, stabilometry, and rheovasography (RVG). After the rehabilitation course, the piriformis muscle changes decreased down to 0.9%. Student’s t test proved the 3-P algorithm for rehabilitation to be the most effective. The average value of t-emp applicability evaluating the proposed hypothesis is >2.042 of t-critical values (*p* = 0.05).

**PS management requires a new diagnostic approach in the framework of predictive, preventive, and personalized medicine**

In the end, the main problem is undiagnosed PS, which requires a new diagnostic approach in the framework of the predictive, preventive, and personalized medicine approach based on the targeted preventive measures correction of physical activity, early multi-level diagnostics, improved patient stratification, and treatment algorithms, taking into account their individual health characteristics and manifestations of the disease.

**Conclusions** Thus, predictive, preventive, and personalized medicine is effective in patients suffering from PS. A population study at the initial stages of the disease is necessary to prevent syndrome progression and enable rapid recovery. Identifying the abnormal variation of the sciatic nerve makes it possible to prevent future disease development. Finally, 3PPPM is essential to improve individual outcomes at any stage and treat the manifested disorder.

**References**

1. Thomas EN, Cyteval C, Remy A, Herisson C; Blotman F. Syndrome myofascial du muscle piriforme et sciatalgie persistante chez le lombalgique en reeducation. Lett Méd Phys Réadapt. 2009;25:158–161. 10.1007/s11659-009-0127-1
2. Fishman LM, Polesin A, Sampson S. Piriformis syndrome - a diagnosis comes into its own. Muscle Nerve. 2019;59(4 Suppl). 10.1002/mus.26417.
3. Zuev DS, Kostenko EV, Petrova YA. The effectiveness of complex application of osteopathy and shock wave therapy in the rehabilitation treatment of myofascial pain syndrome. 2019;10(2):36–45. 10.17816/clinpract10236-45
4. Ro TH, Edmonds L. Diagnosis and management of piriformis syndrome: a rare anatomic variant analyzed by magnetic resonance imaging. J Clin Imaging Sci. 2018;8:6. 10.4103/jcis.JCIS_58_17
5. Gagey PM et Weber B Avec la collaboration de Bonnier L, J. Boquet j; J.-Y. Curnu J-Y; G. Rerrey G, C. Marucchi C, Pichon J, Scheibel A, Toupet M, Villeneuve Ph et Zamfiresco F. Posturologie-regulation et dereglements de la station debout. Paris Masson 1995, 1999, 2004; ISBN 9785980371234
6. Ugrenović S, Jovanović I, Krstić V, Stojanović V, Vasović L, Antić S, Pavlović S. The level of the sciatic nerve division and its relations to the piriform muscle. Vojnosanit Pregl. 2005 Jan;62(1):45–9. 10.2298/vsp0501045u

## 3PM in cardio-vascular disease

**The syndrome of early vascular aging and health protection system in youth**

Evsevyeva M*^1,2^, Eremin M^1^, Fursova E^1^, Rusidi M^1^, Koshel V^1^

^1^University Health Centre, Stavropol State Medical University, Russian Federation

^2^ Department of Internal Deseases, Stavropol State Medical University, Russian Federation

*Corresponding author: Prof. Dr. Maria Evsevyeva, University Health Centre, Department of Internal Deseases, Stavropol State Medical University, Mira Str. 310, 355017, Stavropol, Russian Federation; e.mail: evsevieva@mail.ru

**Keywords**: predictive preventive personalized medicine, early vascular aging, preclinical atherosclerosis, young people, risk factors, tissue biomarker, cardio-ankle vascular index

**Background**

Despite the success achieved in treatment and prevention of atherosclerotic cardiovascular (СV) diseases, they remain in leading positions in the overall structure of morbidity and mortality worldwide [1]. This situation is largely due to the lack of an effective system of mass predictive diagnostics in active age individuals [2], which until recently was limited to assessing the main risk factor (RF) profile [3,4]. Meanwhile, young people (YP) may be carriers of preclinical atherosclerosis [5]. It has already been established that atherosclerotic diseases begin to develop on average 30 years before their clinical manifestation. The modern lifestyle predisposes persons to their rapid rejuvenation. Recommended riskmetric scales were studied primarily in persons of more mature age. Therefore, hopes are pinned on the timely diagnosis of original vascular phenotype, estimated by vascular age (VA), which, in turn, is predetermined by an indicator of arterial wall stiffness. Obtaining such data is necessary to address the issues of individualized prevention of these diseases and their further personalized treatment. Therefore, some authors have recently proposed the concept of early vascular aging or EVA syndrome [6]. Its detection indicates that the patient has the most unfavorable vascular phenotype, in which his biological age exceeds passport (chronological) age [2]. A high level of predictive potential in such phenotype has already been proven [5]. Some authors believe that using vascular age is associate with more effectively predicting features of the atherosclerotic pathology course in comparison with traditional riskometric systems.

**Aim**

To compare predictive instrumental screening data to detect EVA cases among YP with results of common riskometric scales, as well as to evaluate the possibility of correcting their vascular stiffness in the course of preventive intervention.

**Material and methods**

Within the framework of the University’s preventive project, angiological screening was performed in 243 YP 18–25 y.o. using the VaSera-1500 device (Fukuda Denshi, Japan), which allows evaluating such measure of vascular stiffness as cardio-ankle vascular index (CAVI). Quartile analysis of CAVI was performed. According to experts, the fourth CAVI-quartile corresponds to EVA-syndrome. The profile of main RF was also assessed. We used European Society of Cardiology (ESC) and Framingham riskometric scales. CAVI was evaluated before and after an 8-month preventive intervention that included correction of identified RF. The preventive program was carried out under guidance of an interdisciplinary team of specialists from the University Health Center using real and Internet technologies to monitor implementation of the program by each participant. Data was processed using the “Statistica 10.0” software package (StatSoft Inc., USA).

**Results**

In this YP sample, the CAVI minimum was 3.3 and maximum was 8.1. The CAVI value corresponding to EVA-syndrome was higher than 6.5 in boys and higher than 6.3 in girls. According to results obtained using the accepted risk-measuring scales ECS and Framingham, all young carriers of EVA-syndrome had low CV risk. This suggests that, on one hand, these scales have no diagnostic value for YP, and, on other hand, the assessment of VA is reclassifying potential for CV risk in this age group. Due to this instrumental technology, YP have an understanding of the difference between their passport and vascular age. It also influenced the motivation of YP in terms of their participation in preventive educational activities. The coverage of healthy lifestyle education was significantly higher among those who passed this survey—95.4% compared to 37.8% in unexplored persons (*p* < 0.05). This YP expressed a desire to participate in a preventive program for correcting behavioral RF. For eight months of such preventive intervention, the CAVI-level among boys decreased by 9.3% (n/s), and among girls it decreased by 14.2% (*p* < 0.05). The presented data are consistent with the opinion of other authors on the feasibility of early predictive diagnosis of preclinical atherosclerosis in YP [6]. Angiological screening is optimally suited for this purpose. The predictive diagnostic advantages of vascular stiffness compared with other RF are due to its nature as a “tissue” biomarker rather than a “circulating” biomarker such as lipids and glucose [3]. Arterial stiffness integrates the long-term effect of all other RF.

**PPPM-associated conclusion and prospects**

Thus, angiological screening is a more informative method compared to riskometric scales for predictive diagnostics in YP. Detection of unfavorable vascular phenotype makes it possible to transfer young carriers of EVA-syndrome from the low- to high-risk CV group. The timely start of behavioral RF-correction within preventive intervention with the aim of maximum individualization is important. In the case of further development of clinical manifestations of atherosclerotic CV disease, the availability of the premorbid dynamics of previous vascular phenotype will contribute to more personalized therapy of detected pathology. This approach to individualized long-term management of persons starting from their young age is fully consistent with the principles of predictive, preventive, and personalized medicine [7].

**References**

1. Polivka J Jr., Polivka J, Pesta M, Rohan V, Celedova L, Mahajani S, et al. Risks associated with the stroke predisposition at young age: facts and hypotheses in light of individualized predictive and preventive approach. EPMA J. 2019;10:81–99. 10.1007/s13167-019-00162-5
2. Nilsson P et al. Early vascular ageing in translation: from laboratory investigations to clinical applications in cardiovascular prevention. J Hypertens. 2013;31(8):1517–1526. 10.1097/HJH.0b013e328361e4bd.
3. Evsevyeva M, Rostovtseva M, Gal’kova I, Rusydi A. Аbout correlation of socio-psychological status and factors of cardio-vascular risk at young contingent. In: Breaking down the barriers (EUSUHM 2013), poster abstracts. 2013;37. 10.29296/25877305-2018-10-17
4. Evsevyeva M, Kumukova Z. Features of the psychological status of young people with signs of arterial hypertension. Russian Psychiatric J. 2007:3:53–57. eLIBRARY ID: 21130981.
5. van de Laar R, Stehouwer C, Prins M, van Mechelen W, Twisk J, Ferreira I. Self-reported time spent watching television is associated with arterial stiffness in young adults: the Аmsterdam Growth and Health Longitudinal Study. Br J Sports Med. 2014;48:256–264. 10.1136/bjsports-2013-092555.
6. Laurent S, Boutouyrie P, Cunha P, Lacolley P, Nilsson P. Concept of extremes in vascular aging from early vascular aging to supernormal vascular aging. Hypertension. 2019;74:218–228. 10.1161/HYPERTENSIONAHA.119.12655
7. Chaari L (Ed.). Digital health approach for predictive, preventive, personalised and participatory medicine. Series: Advances in predictive, preventive and personalised medicinе. 2019; Vol. 10. ISBN 978-3-030-11800-6

**The Influence of Vitamin K Status on the Individual Course of Age-dependent Arterial Stiffening**

Mayer O*^1,2^, Gelzinsky J^1,2^, Seidlerova J^1,2^, Kucera R^3^, Materankova M^1^, Mares S^1^, Svobodova V^1^, Topolcan O^3^, Cifkova R^4^, Filipovsky J^1,2^, Vermeer C^5^

^1^2nd Department of Internal Medicine, Medical Faculty of Charles University and University Hospital, Pilsen, Czech Republic;

^2^Biomedical Center, Medical Faculty of Charles University, Pilsen, Czech Republic;

^3^Department of Immunochemistry Diagnostics, Medical Faculty of Charles University and University Hospital, Pilsen, Czech Republic

^4^Centre for Cardiovascular Prevention of the First Faculty of Medicine, Charles University and Thomayer Hospital, Prague, Czech Republic;

^5^Cardiovascular Research Institute CARIM, Maastricht University, The Netherlands

*Corresponding author: Otto Mayer, University Hospital, 2nd Dept. of Internal Medicine, Pilsen CZ

e.mail: mayero@fnplzen.cz

**Keywords:** Predictive preventive personalized medicine, cardiovascular, matrix γ-carboxyglutamate MGP, pulse wave velocity, vascular aging, Vitamin K

**Background**

The progressive loss of elastic properties of large arteries is a typical phenotype of vascular aging. The underlying mechanisms (collectively known as “arteriosclerosis”) involve several natural (age-dependent) pathophysiological processes, such as the fragmentation and thinning of elastin lamellae due to its “material fatigue” from cyclic loading, the cross-linking of collagen fibers by advanced glycation end-products as well as several others [1]. Arteriosclerosis coexists with atherosclerosis (the development of intimal atheromatous plaques) and both processes jointly contribute to individual morbidity risk. Numerous studies have demonstrated that increased stiffness of large central arteries (quantified as aortic pulse wave velocity, PWV) is an independent predictor of morbidity and mortality [2].

One of the most important mechanisms of arteriosclerosis is the calcification of elastic elements in the vessel wall. Vascular or tissue calcification represents a highly regulated and potentially reversible process. On a cellular level, it shares many pathways involved in bone mineralization [3]. The matrix γ-carboxyglutamate protein (matrix Gla protein, MGP) represents the natural factor of the “anti-calcification defense” of human tissues (allowing even a reversal of the calcification process) and vitamin K is the essential co-factor of its maturation to biologically active forms [4]. Circulating desphospho-uncarboxylated isoform of MGP (dp-ucMGP), a biomarker of both vitamin K status and biological activity of MGP, was associated with the extent of vascular calcification, as well as the incidence risk of cardiovascular events [4,5].

Predictive, preventive, personalized medicine (PPPM) strategies for cardiovascular diseases are receiving increasingly more attention of late [6]. The immediate effort should be focused on screening methods and the indication of suitable interventions for particular individuals based on their cardiovascular risk stratification. In concordance with PPPM, new biomarkers and multivariate models are frequently used and the promising results achieved [6].

In line with the physiological role of vitamin K and MGP, we have investigated the association between baseline dp-ucMGP concentrations and the following natural course of arterial stiffening in a prospective study based on the general population setting.

**Methods**

The study cohort consisted of 541 individuals with a median follow-up of 8.0 years, all examined as a part of the Czech post-MONICA study in 2008 and 2016/17 [7].

Aortic PWV was obtained after 15 min of rest in a supine position using a Sphygmocor MM3 device ((AtCor Medical Ltd., Sydney, NSW, Australia). Intra-individual PWV difference (follow-up *minus* baseline value) was divided by the exact time between these two visits and a parameter obtained (∆PWV/year, i.e. individual change of PWV per year of follow-up) to be used as the primary estimate of individual age-dependent arterial stiffening. Accelerated arterial stiffening was arbitrarily set as ∆PWV/year ≥0.2 m/s. In addition to the basic parameters (lipids, creatinine, glucose, etc.), the concentration of the soluble receptor for advanced glycation end products (sRAGE) was quantified using ELISA methods (R&D Systems Inc., Minneapolis, MN, USA). Dp-ucMGP was quantified in citrate plasma samples using the Ina*K*tif MGP iSYS kit (IDS, Boldon, UK), a pre-commercial automated assay based on the sandwich (dual antibody) ELISA kits developed by VitaK (Maastricht University, The Netherlands).

**Results**

Detailed characteristics of the study sample at baseline are shown in Table 1. The univariate association between ∆PWV/year and other clinical parameters are shown in Table 2. Age, male gender, history of vascular disease, treatment with antihypertensives, with RAAS blockers and statins all significantly positively correlated with ∆PWV, while a negative association was found between sRAGE and baseline PWV. Both baseline PWV and ∆PWV per year increased across dp-ucMGP quintiles (Fig. 1) and these trends in both dependent variables remained significant after adjustment for potential covariates, i.e. age, gender, mean arterial pressure, sRAGE, treatment with antihypertensive drugs and with antidiabetics, *plus* baseline PWV if ∆PWV per year was compared (Fig. 1).

**Table 1** Baseline characteristics of study sample mean (SD) or *factor proportion*)

**Table:** 

*n*	530
age[years]	53.9 (12.3)
*male gender [%]*	*45.2*
*manifest vascular disease [%]*	*5.3*
*current smoking [%]*	*30.6*
body mass index[kg/m^2^]	26.9 (4.4)
systolic blood pressure [mmHg]	127.3 (16.0)
diastolic blood pressure [mmHg]	80.9 (9.0)
mean arterial pressure[mmHg]	96.4 (10.3)
*any antihypertensives [%]*	*29.1*
*RAAS blockers [%]*	*18.9*
*arterial hypertension*^#^	*42.5*
LDL cholesterol[mmol/L]	3.11 (0.89)
*statins [%]*	*12.6*
fasting glycemia[mmol/L]	5.21 (0.82)
*antidiabetics [%]*	*1.9*
*overt diabetes*^*$*^*[%]*	*4.2*
estimated glomerular filtration (CKD-EPI) [mL/min]	86.0 (15.3)
sRAGE [pg/mL]	1349 (758)
dp-ucMGP [pmol/L]	536 (273)
*warfarin[%]*	2.0
baseline PWV [m/s]	7.6 (2.0)
follow-up PWV[m/s]	9.1 (2.3)
∆PWV/year^§^ [m/s]	0.18 (0.24)

RAAS, renin-angiotensin system; LDL, low-density cholesterol; sRAGE, soluble receptor for advanced glycation end products; dp-ucMGP, desphospho-uncarboxylated matrix Gla protein; PWV, aortic pulse wave velocity

^#^systolic ≥140 or diastolic blood pressure ≥ 90 mmHg, or treatment with antihypertensives; ^*$*^fasting glycemia ≥7 mmol/L or treatment with antidiabetics; ^§^ *see* Methods for definition; italicized items are highly relevant for conclusions

**Table 2** Association between the individual progression of arterial stiffening (∆PWV per year) and its covariates (Spearman’s rank correlation and multiple linear stepwise regression)

**Table:** 

	Univariate model		Multivariate step-wise model	
	Spearman’s R	*p value*	β coeff. (SE)	*p value*
age	0.154	0.0003	0.0073 (0.0009)	<0.0001
male gender	0.127	0.0032	0.0515 (0.0177)	0.004
manifest vascular disease	0.094	0.028		
current smoking	0.033	0.45		
body mass index	0.069	0.11		
mean arterial pressure	−0.052	0.23		
LDL cholesterol	−0.036	0.41		
fasting glycemia	0.047	0.28		
estimated glomerular filtration	−0.046	0.28		
any antihypertensives	0.133	0.0019	−0.1279 (0.0445)	0.004
RAAS blockers	0.125	0.0036	0.0466 (0.0209)	0.026
statins	0.106	0.014		
antidiabetics	0.094	0.067		
warfarin	0.023	0.60		
sRAGE	−0.192	<0.0001	0.3498 (0.0674)	<0.0001
baseline PWV	−0.310	<0.0001	−0.0801 (0.0051)	<0.0001
dp-ucMGP	0.128	0.0032	0.1150 (0.0474)	0.016
*const.*			*0.4406 (0.1847)*	*0.017*

log-transformations were done in sRAGE and dp-ucMGP

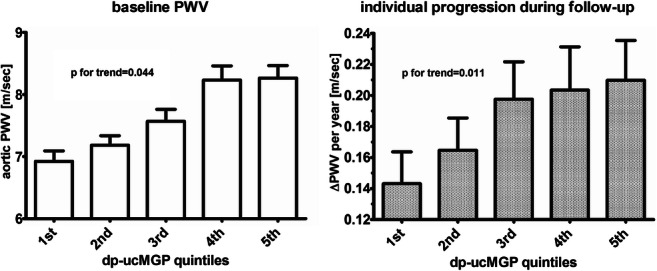



**Fig. 1** Baseline pulse wave velocity (left panel) and its progression during follow-up (∆PWV per year, right panel) among dp-ucMGP quintiles [mean (standard deviation)]; limits of dp-ucMGP quintiles are as follows: ≤356, 357–468, 469–568, 569–690 and ≥ 691 pmol/L; *p* value fully adjusted for potential covariates (*see* Results for details)

Furthermore, arbitrary ∆PWV per year ≥0.2 m/s was used as a dependent variable (reflecting the accelerated individual progression of aPWV during follow-up) and confirmatory results were observed. After adjustment for potential covariates, an increase in dp-ucMGP amounting to one quintile was associated with approximately a 24% higher relative risk of accelerated aortic stiffening (Table 3, model A). A confirmatory finding was found by comparing the 1st quintile of dp-ucMGP with the higher ones (2nd to 5th; Table 3, model B).

**Table 3** Predictors of accelerated individual progression of arterial stiffness (∆PWV per year ≥0.2 m/s) using multivariate logistic regression models

**Table:** 

	Model A		Model B	
	Odds ratio	*p value*	Odds ratio	*p value*
age ≥ 65 years	2.12 (1.28–3.51)	0.004	2.11 (1.27–3.50)	0.004
male gender	1.59 (1.07–2.37)	0.022	1.63 (1.10–2.42)	0.016
manifest vascular disease	1.40 (0.57–3.43)	0.47	1.47 (0.60–3.59)	0.40
current smoking	1.43 (0.95–2.14)	0.10	1.40 (0.93–2.10)	0.10
body mass index ≥30 kg/m^2^	1.25 (0.78–2.00)	0.37	1.31 (0.82–2.10)	0.26
arterial hypertension	0.95 (0.58–1.55)	0.75	0.93 (0.57–1.50)	0.75
LDL cholesterol ≥2.5 mmol/L	1.49 (0.94–2.38)	0.093	1.52 (0.95–2.41)	0.75
overt diabetes	1.04 (0.38–2.83)	0.87	1.09 (0.41–2.93)	0.87
estimated glomerular filtration≤90 mL/min	1.04 (0.68–1.58)	0.86	1.11 (0.73–1.68)	0.63
RAAS blockers	1.56 (0.86–2.82)	0.11	1.61 (0.89–2.90)	0.11
statins	1.51 (0.79–2.88)	0.21	1.61 (0.84–3.06)	0.15
warfarin	0.38 (0.09–1.61)	0.19	0.50 (0.12–3.00)	0.34
sRAGE<881 pg/mL	1.85 (1.13–3.02)	0.015	1.84 (1.12–3.00)	0.016
baseline PWV ≥ 9 m/s	0.35 (0.21–0.59)	<0.0001	0.37 (0.22–0.61)	<0.0001
increase of dp-ucMGP by one quintile	1.24 (1.07–1.43)	0.003	–	
dp-ucMGP in 1st quintile (< 357 pmol/L)	–		0.57 (0.35–0.92)	0.021

**Data interpretation**

The key finding of this prospective, general population-based study is that vitamin K status plays an important pathophysiological role in the natural, age-dependent progression of arterial stiffening. High vitamin K status (low dp-ucMGP) was independently associated with approximately a 43% lower risk of accelerated individual progression of PWV during 8 years of follow-up (defined as 0.2 m/s per year). It is generally accepted that the “natural course” of vascular aging corresponds to approximately a 1 m/s increase in PWV per decade of life [8]. We might say that during the 8-year follow-up, high vitamin K status “slowed” the aging of arteries by about 5 years compared to the subjects with low vitamin K status.

So far, at least 11 smaller or medium-sized interventional studies have shown that supplementation of iso-vitamin K_2_ (menaquinone, MK-7) leads to a decrease in circulating dp-ucMGP [9]. Moreover, another intervention trial with MK-7 supplementation also demonstrates the effect on arterial stiffness [10]. In line with our observations, the sub-study of the ASTRONOMER trial also indicated high dephosphorylated MGP concentrations independently associated with a higher risk of progression of aortic stenosis [11].

**Conclusions and expert recommendations**

- Vitamin K status was identified as an independent predictor of the individual course of arterial stiffening. In theory, it can also be used as a therapeutic target to slow-down vascular aging.
- dp-ucMGP was identified as a promising tool for risk prediction and personalization of cardiovascular prevention.
- In concordance with PPPM principles, we support the creation of new multiparameter algorithms and multivariate models for risk assessment and patient stratification.

**Acknowledgments**

Supported by the Health Development Agency of Czech Ministry of Health (project 15-27109), Charles University Research Fund (PROGRES, project Q39), by IDS Plc Boldon, UK, and by FNPl, 00669806.

**References**

1. O’Rourke MF, Mancia G. Arterial stiffness. J Hypertens. 1999;17:1–4.
2. Vlachopoulos C, Aznaouridis K, Stefanadis C. Prediction of cardiovascular events and all-cause mortality with arterial stiffness: a systematic review and meta-analysis. J Am Coll Cardiol. 2010; 10.1016/j.jacc.2009.10.061.
3. Keeley FW, Partridge SM. Amino acid composition and calcification of human aortic elastin. Atherosclerosis. 1974;19:287–96.
4. Dalmeijer GW, van der Schouw YT, Vermeer C, Magdeleyns EJ, Schurgers LJ, Beulens JW. Circulating matrix Gla protein is associated with coronary artery calcification and vitamin K status in healthy women. J Nutr Biochem. 2013; 10.1016/j.jnutbio.2012.02.012.10.
5. Mayer O Jr., Seidlerová J, Bruthans J, Filipovský J, Timoracká K, Vaněk J, et al. Desphospho-uncarboxylated matrix Gla-protein is associated with mortality risk in patients with chronic stable vascular disease. Atherosclerosis. 2014; 10.1016/j.atherosclerosis.2014.04.027.
6. Golubnitschaja O, Costigliola V, EPMA. General report & recommendations in predictive, preventive, and personalized medicine 2012: white paper of the European Association for Predictive, Preventive, and Personalised Medicine. EPMA J. 2012; 10.1186/1878-5085-3-14.
7. Barrett M, Boyne J, Brandts J, Brunner-La Rocca HP, De Maesschalck L, et al. Artificial intelligence supported patient self-care in chronic heart failure: a paradigm shift from reactive to predictive, preventive and personalized care. EPMA J. 2019 Nov 22;10(4):445–464. 10.1007/s13167-019-00188-9.
8. Herbert A, Cruickshank JK, Laurent S, Boutouyrie P; Reference Values for Arterial Measurements Collaboration. Establishing reference values for central blood pressure and its amplification in a general healthy population and according to cardiovascular risk factors. Eur Heart J. 2014; 10.1093/eurheartj/ehu293.
9. Roumeliotis S, Dounousi E, Eleftheriadis T, Liakopoulos V. Association of the Inactive Circulating Matrix Gla Protein with Vitamin K Intake, Calcification, Mortality, and Cardiovascular Disease: A Review. Int J Mol Sci. 2019; 10.3390/ijms20030628.
10. Knapen MH, Braam LA, Drummen NE, Bekers O, Hoeks AP, Vermeer C. Menaquinone-7 supplementation improves arterial stiffness in healthy postmenopausal women. A double-blind randomized clinical trial. Thromb Haemost. 2015; 10.1160/TH14-08-0675.
11. Capoulade R, Côté N, Mathieu P, Chan KL, Clavel MA, Dumesnil JG, et al. Circulating levels of matrix Gla protein and progression of aortic stenosis: a substudy of the Aortic Stenosis Progression Observation: Measuring Effects of rosuvastatin (ASTRONOMER) trial. Can J Cardiol. 2014; 10.1016/j.cjca.2014.03.025.

**Relevance of Lp-PLA**_**2**_ **blood concentration for morphological structure of atherosclerotic plaque: a shift from reactive to predictive, preventive and personalized medicine to predict stroke in patients with asymptomatic internal carotid artery stenosis**

Kopolovets I^1,2^, Berek P*^1^, Stefanic P^1^, Lotnyk D^3^, Mucha R^4^, Hertelyova Z^5^, Toth S^6^, Boyko N^2,7^, Sihotsky V^1^

^1^Clinic of Vascular Surgery, Eastern Slovak Institute of Cardiovascular Diseases and Faculty of Medicine, Pavol Jozef Safarik University, Kosice, Ondavska 8, Kosice 04001, Slovak Republic

^2^Uzhhorod National University, Research Development and Educational Center of Molecular Microbiology and Mucosal Immunology, Narodna Square 1, Uzhhorod 88000, Ukraine

^3^Department of Physics Cornell University Clark Hall, 142 Sciences Drive Ithaca 14853, New York, United States

^4^Institute of Neurobiology, Biomedical Research Center of the Slovak Academy of Sciences, Soltesovej 4, Kosice, 04001, Slovak Republic

^5^Institute of Experimental Medicine, Faculty of Medicine, Pavol Jozef Safarik University, Kosice, Tr. SNP 1, Kosice, 04001, Slovak Republic

^6^Clinic of Cardiology, Eastern Slovak Institute of Cardiovascular Diseases and Faculty of Medicine, Pavol Jozef Safarik University, Kosice, Ondavska 8, Kosice 04001, Slovak Republic

^7^Uzhhorod National University, Department of Clinical Laboratory Diagnostics and Pharmacology, Narodna Square 3, Uzhhorod 88000, Ukraine

^*^Corresponding author: Peter Berek, Clinic of Vascular Surgery, Eastern Slovak Institute of Cardiovascular Diseases and Faculty of Medicine, Pavol Jozef Safarik University, Kosice, Ondavska 8, Kosice 04001, Slovak Republic; e.mail: berekp67@gmail.com

**Keywords:** predictive preventive personalized medicine (3PM), ischemic stroke, atherosclerosis, carotid artery stenosis, vulnerable plaque, vascular markers, carotid endarterectomy, inflammation, Lp-PLA_2_ concentration, blood, homocystein, risk assessement, paradigm shift, disease management

**Background**

Over one million Europeans are stroke-diagnosed annually [1]. Manifested atherosclerotic lesions of the carotid arteries account for approximately 40% of all ischemic strokes, and genetic predisposition, hypertension, among others are considered the best acknowledged risk factors [2]. However, the etiology of many stroke cases remains unclear, particularly in young patients below 50 years of age [2]. To this end, one of the most promising research areas involves the risks linked to the vascular inflammation and the follow-up cascade of pathological changes which altogether may lead to the clinical manifestation of stroke [3].

According to the EPMA criteria, clinically relevant biomarker-sets should serve for predictive diagnosis, targeted prevention and personalization of medical services, in order to contribute to the paradigm shift from reactive to 3PM [4]. Contextually, consideration of multi-factorial risks and individualized patient profiling provide comprehensive information for the clinically relevent biomarker-sets and multi-parametric analysis as tools for the 3PM implementation. A prospective pilot research project was carried out at the Clinic of Vascular Surgery of the Eastern Slovak Institute of Cardiovascular Diseases and the Faculty of Medicine of Pavol Jozef Safarik University, Kosice. The main criteria evaluated in our study included the degree of ICA stenosis, the morphological structure of the atherosclerotic plaque and the level of lipoprotein-associated phospholipase A_2_ (Lp-PLA_2_) concentration.

**Working hypothesis**

This project tested the hypothesis that the risk of stroke in patients with internal carotid artery (ICA) stenosis cannot be evaluated based on the determination of the degree of ICA stenosis only. In addition to stenosis, the morphological structure of the atherosclerotic plaque can serve as an important risk factor as well, while ulceration and instability of atherosclerotic plaques can be characterized by biomarkers.

**Materials and methods**

The study included 70 (27 females and 43 males) patients, who were hospitalized for carotid endarterectomy. The patients were divided into two groups depending on their symptoms: Group I included 30 patients with symptomatic ICA stenosis >50%; Group II comprised 70 patients with asymptomatic ICA stenosis >70%. There was no statistically significant difference in age and sex between the groups. Ultrasound scans were used to assess ICA stenosis, as well as the structure of the atherosclerotic plaque. Before surgery, blood samples were taken to determine the levels of the following biomarkers: Lp-PLA_2_, IL-4, hemopexin, and homocysteine.

**Results**

When assessing the morphological structure of the atherosclerotic plaque using ultrasound, soft and mixed atherosclerotic plaques were detected in 73% of the patients in group I and 55% of the patients in group II. The comparison of Lp-PLA_2_ concentration ratio revealed a statistically significant correlation (*p* < 0.001) between the increase in Lp-PLA_2_ concentration in the patients with symptomatic ICA stenosis (285.30 ± 2.05 μg/l) compared to the patients with asymptomatic ICA stenosis (274.35 ± 3.38 μg/l). In 15% of asymptomatic patients with soft atherosclerotic plaque, Lp-PLA_2_ level was higher (293.90 ± 1.5 μg/l) than that in symptomatic patients with hard atherosclerotic plaque (261.40 ± 1.3 μg/l). The concentration of Lp-PLA_2_ correlated with atherosclerotic plaque structure rather than its size.

The comparison of serum hemopexin levels in symptomatic (0.38 ± 0.01 ng/l) and asymptomatic (0.351 ± 0.012 ng/l) patients revealed no statistically significant difference between both groups.

In the patients with symptomatic ICA stenosis, the serum concentration of IL-4 (65.77 ± 3.78 ng/l) was found to be significantly higher compared to the patients with asymptomatic ICA stenosis (42.69 ± 1.73 ng/l).

The analysis of the homocysteine test revealed a statistically significant difference between the patients with symptomatic ICA stenosis and those with asymptomatic ICA stenosis. At the same time, homocysteine as a marker of atherosclerotic complications was inferior to Lp-PLA_2_ when comparing the following risk factors, namely the degree of ICA stenosis, the morphological structure of the atherosclerotic plaque, and the concentration of the “optimal” marker.

**Data interpretation**

There is a wide range of vascular biomarkers; however, each biomarker has its own specificity and changes in its concentration may depend on different factors [3]. Among vascular markers analyzed, a statistically significant correlation between the degree of ICA stenosis, the structure of the atherosclerotic plaque and the clinical course were observed only in the case of Lp-PLA_2_. The dominant factor for the increase in Lp-PLA_2_ was the structure of the atherosclerotic plaque. According to the results of our study, unstable atherosclerotic plaques are typical for symptomatic patients, as well as for the patients with asymptomatic ICA stenosis. Our results are consistent with the results obtained by other authors who observed the patients with asymptomatic carotid stenosis and found that the patients with unstable echolucent plaques were 2.31 times more likely to develop stroke compared to the patients with stable plaque based on ultrasound findings [5]. Consideration of Lp-PLA_2_ concentration in the patients with ICA stenosis may serve as an auxiliary criterion at the stage of determining and selecting treatment tactics for patients with ICA stenosis greater than 70%, and finally to apply a predictive and prognostic patient-specific treatment of atherosclerosis promoting the change from reactive medicine to 3PM. In addition, the proposed vascular biomarker Lp-PLA_2_, which depends on the individual lipid profile, can be applied alternatively to the typical post-symptomatic treatment of atherosclerosis. HDL-associated Lp-PLA_2_ may substantially contribute to the HDL antiatherogenic activity and could be additionally used to predict the efficacy of medication prescribed.

**Conclusions**

Our research demonstrated a statistically significant correlation between Lp-PLA_2_ and the degree of ICA stenosis, the structure of the atherosclerotic plaque, and the clinical course. The increase in Lp-PLA_2_ level (285.30 ng/ml ± 2.05) and ultrasound findings of soft atherosclerotic plaque in combination with stenosis degree may indicate a high embolic potential in the patients with ICA stenosis.

Consideration of the morphological structure of the atherosclerotic plaque and Lp-PLA_2_ concentration in the patients with ICA stenosis is recommended for the implementation of a personalized approach to therapies that meet the criteria of 3PM.

The implementation of the 3PM strategy and personalized approach is one of the optimal directions to find the additional criteria for early identification of risk factors for stroke.

It is anticipated that, ultimately, this change in diagnosis and therapy will help in the future design and development of new, more selective, and effective therapies for each individual patient.

**References**

1. Polivka J, Polivka J Jr, Rohan V. Predictive and individualized management of stroke-success story in Czech Republic. EPMA J. 2018;9(4):393–401. 10.1007/s13167-018-0150-x.
2. Polivka J Jr., Polivka J, Pesta M, Rohan V, Celedova L, Mahajani S, et al. Risks associated with the stroke predisposition at young age: facts and hypotheses in light of individualized predictive and preventive approach. EPMA J. 2019;10: 81–99. 10.1007/s13167-019-00162-5.
3. Poredos P, Jezovnik MK. The Role of Inflammatory Biomarkers in the Detection and Therapy of Atherosclerotic Disease. Curr Vasc Pharmacol. 2016;14(6):534–546.
4. Golubnitschaja O, Baban B, Boniolo G, Wang W, Bubnov R, Kapalla M, et al. Medicine in the early twenty-first century: paradigm and anticipation - EPMA position paper 2016. EPMA J. 2016 Oct;7:23. eCollection 2016. 10.1186/s13167-016-0072-4.
5. Li D, Wei W, Ran X, Yu J, Li H, Zhao L, et al. Lipoprotein-associated phospholipase A2 and risks of coronary heart disease and ischemic stroke in the general population: A systematic review and meta-analysis. Clinica Chimica Acta. 2017;471:38–45. 10.1016/j.cca.2017.05.017.

**Contrast-enhanced ultrasound in detection of the endoleak: a possible role in a personalized approach to the follow-up after endovascular repair of the abdominal aneurysm**

Mirka H*^1,3^, Korcakova E^1^, Duras P^1^, Houdek K^2^, Molacek J^2^, Hosek P^3,4^

^1^Department of Medical Imaging, ^2^Department of Surgery, University Hospital and Faculty of Medicine in Pilsen, Charles University, Czech Republic; ^3^Biomedical Centre, ^4^Department of Histology and Embryology, Faculty of Medicine in Plzen, Charles University, Czech Republic.

*Corresponding author: Hynek Mirka, assoc. prof., MD, PhD, Department of Medical Imaging, University Hospital in Pilsen, Czech Republic; e.mail: mirka@fnplzen.cz, ORCID ID: 0000-0002-7546-5625

**Keywords:** Preventive predictive and personalized medicine (PPPM), abdominal aorta, aneurysm, endovascular repair, follow-up, endoleak, diagnostic imaging, ultrasound, contrast-enhanced ultrasound, computed tomography

**Introduction**

The prevalence of abdominal aortic aneurysms (AAA) is 4–8%. In Western countries it is responsible for 4 to 5% of sudden deaths [1]. Endovascular aneurysm repair (EVAR) was introduced in 1991. EVAR is less invasive than open surgery; however, it requires life-long monitoring due to potential complication [2].

Endoleak is the most frequently occurring complication associated with EVAR. It occurs in up to 45% of patients. Treatment decisions regarding this complication are made individually based on the detection and determination of the type of endoleak. The most frequently occurring endoleak is type II ensuing from the superior mesenteric or lumbar artery (Fig. 1). In approximately 50% of cases, the endoleak can close spontaneously, and the initial treatment is conservative [3]. CT angiography (CTA) is the most frequently used method of monitoring the patients with EVAR. The alternatives of CTA are, primarily, MR angiography (MRA) and contrast-enhanced ultrasonography (CEUS).
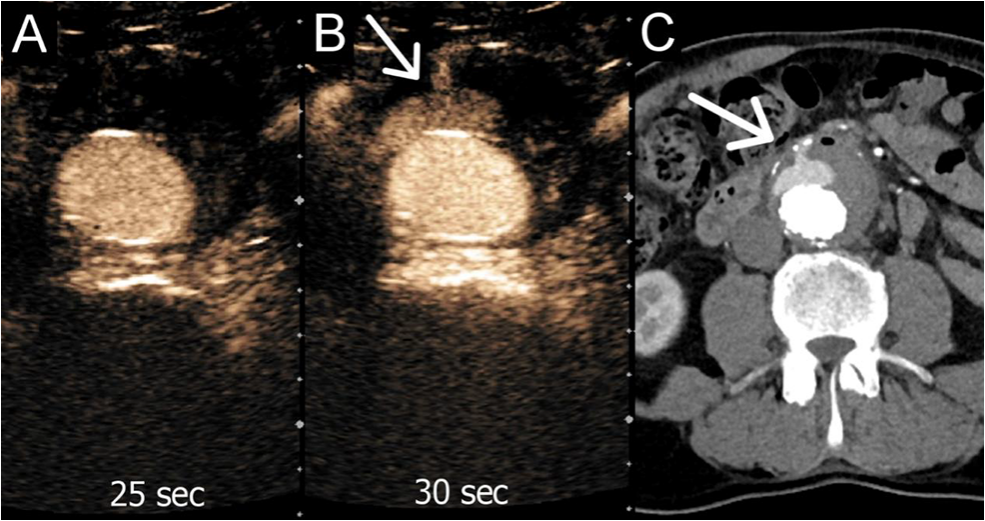


**Fig. 1** Type II endoleak. The endoleak (**b**) occurs with delay over contrast filling of the stengraft (**a**). Endoleak is indicated by the arrow in image **b**. CT image correlates with US image (**c**), endoleak is highlighted by the arrow

Our study is aimed at verifying the use of contrast-enhanced ultrasonography (CEUS) in monitoring patients after an implantation of aortic stent-graft.

**Materials and methods**

CEUS examinations were performed by two radiologists with more than 5 and 7 years of experience. They were blinded to the CTA results. The sonography result was determined by way of consensus of both readers. The examinations were performed using Acuson Antares and Acuson S 2000 scanners (Siemens, Erlangen, Germany) and a low-frequency convex probe (3.5 MHz). We administered one or two doses of 2.4 ml of a 2nd-generation contrast medium (CM) (SonoVue, Bracco, Milano, Italy) with a subsequent 10 ml saline flush into the cubital vein using 20 G cannula. We evaluated endoleak occurrence and type on the postcontrast images.

CTA examinations were performed by Somatom Definition and Somatom Definition Flash scaners (Siemens, Erlangen, Germany) in arterial a venous phase after application of 80 ml of CM (Iomeron 350, Bracco, Milan, Italy). The same parameters as the ones used for CEUS were then evaluated by a radiologist blinded to the CEUS finding.

**Results**

Endoleaks were found in 32 examinations (3 endoleaks type I and 29 endoleaks type II). CEUS detected endoleaks in 29 cases, and 3 examinations were false negative. Using CTA, we identified endoleaks in 33 cases. In one patient with false positivity, newly occurring fine calcification in a thrombosed AAA sac was determined as a potential endoleak. In this case, CEUS provided a correct (i.e. negative) result. The established sensitivity was 90.9% and the established specificity was 100% for endoleak detection. The overall share of correct decisions on endoleak occurrence made using CEUS was 95.2%. Endoleak types were correctly determined in 27 cases, and incorrectly in two cases. Thus, the achieved reliability percentage was 93.3% and sensitivity 84.9%. CEUS failed to correctly determine endoleak occurrence and type recognition in 57 cases; its overall accuracy being 91.94%.

**Data interpretation and PPPM-related expert recommendations**

The study demonstrated the suitability of CEUS in the detection of endoleaks after EVAR. Nevertheless, the results show slightly lower accuracy for this method versus standardly used CTA. Issues associated with endoleak detection occurred in three patients; in all cases, these were small type II endoleaks from the lumbar artery behind the stent graft bifurcation or proximal section of one of the iliac limbs. Issues with detection can be caused by the metal artifacts or stentgraft frame as well as proximity of the stent-graft lumen filled with contrast medium [4–9]. In two cases, the endoleak was correctly detected but incorrectly interpreted (overestimated) as type I instead of type II. Overestimation of endoleaks leads to change in treatment—anticipated interventional treatment need not have been be implemented.

Endoleak types are evaluated with respect to the location of CM leakage, direction of the bubble flow and time span between filling of the stent-graft lumen and inflow of bubbles into the aneurysmal sac. In type I, III and IV endoleaks, the CM leakage appears simultaneously with stent graft filling. In type II endoleaks, there is a period of several seconds between stent-graft filling and the occurrence of bubbles in the sac (Fig. 1). The information on blood flow direction in the endoleak—which cannot be evaluated using CT—is also essential. Undoubtedly, the potential advantage of CEUS consists in the possibility of continual monitoring of CM flow during longer periods, thus improving the visualization of slowly occurring endoleaks that result only in a small change of density in CTA [10].

Evaluation of repeated examinations that were made in identical persons is considered a limitation factor in this study. However, the time span between the examinations of all patients was long enough to avoid the possibility of retaining the preceding medical finding in the memory of the radiologist. CEUS accuracy in evaluation of endoleak types can be distorted by the prevailing occurrence of endoleaks of types III—V.

We recommend performing follow-up examinations immediately after EVAR, and subsequently after 1, 6 and 12 months during the first year. Afterwards, patients without complications are monitored once a year. CEUS can be used as a replacement for CTA follow-up examinations, where suitable. In addition, CEUS examinations can be performed at shorter intervals than CT without significantly increasing the burden on the investigated person.

In conclusion, the results of our study support use of CEUS in the secondary prevention of endoleak-related complications in patients after EVAR. The advantages of this method over CTA are that of reducing the burden of patients and the financial costs of monitoring. Overall, it may be said that CEUS can be used as part of a personalized diagnostic algorithm that contributes to the early prediction of outcomes following EVAR.

**Acknowledgements**

Supported by the Ministry of Health - Conceptual Development of research institutions 00669806, by the project CZ.1.05/2.1.00/03.0076 from European Regional Development Fund and Program Progress Q39 of the Charles University Prague.

**References**

1. Ferda J, Baxa J, Ferdova E, Kucera R, Topolcan O, Molacek J. Abdominal aortic aneurysm in prostate cancer patients: the “road map” from incidental detection to advanced predictive, preventive, and personalized approach utilizing common follow-up for both pathologies. EPMA J. 2019 Nov 26;10(4):415–423. 10.1007/s13167-019-00193-y.
2. Molacek J, Treska V, Zeithaml J, Hollan I, Topolcan O, Pecen L, et al. Blood biomarker panel recommended for personalized prediction, prognosis, and prevention of complications associated with abdominal aortic aneurysm. EPMA J. 2019; 10.1007/s13167-019-00173-2.
3. White SB, Stavropoulos SW. Management of endoleaks following endovascular aneurysm repair. Semin Intervent Radiol. 2009;26:33–8.
4. Gilabert R, Buñesch L, Real MI, García-Criado Á, Burrel M et al. Evaluation of abdominal aortic aneurysm after endovascular repair: prospective validation of contrast-enhanced US with a second-generation US contrast agent. Radiology. 2012;264:269–77.
5. Giannoni MF, Citone M, Rossini M, Speziale F, David V. Role of contrast-enhanced ultrasound in the follow-up of endo-vascular aortic aneurysm repair: an effective and safe surveillance method. Curr Pharm Des. 2012;18:2214–22.
6. Millen A, Canavati R, Harrison G, McWilliams RG, Wallace S, Vallabhaneni SR, et al. Defining a role for contrast-enhanced ultrasound in endovascular aneurysm repair surveillance. J Vasc Surg. 2013;58:18–23.
7. Von Tengg-Kobligk H, Correa Londono M, Von Allmen R, Heverhagen JT, Van Den Berg JC. State-of-the-art of imaging detecting endoleaks post-EVAR with special focus on low-flow endoleaks. J Cardiovasc Surg. 2014;55:563–79.
8. Rübenthaler J, Reiser M, Cantisani V, Rjosk-Dendorfer D, Clevert DA. The value of contrast-enhanced ultrasound (CEUS) using a high-end ultrasound system in the characterization of endoleaks after endovascular aortic repair (EVAR). Clin Hemorheol Microcirc. 2017;66(4):283–92.
9. Chung J, Kordzadeh A, Prionidis I, Panayiotopoulos Y, Browne T. Contrast-enhanced ultrasound (CEUS) versus computed tomography angiography (CTA) in detection of endoleaks in post-EVAR patients. Are delayed type II endoleaks being missed? A systematic review and meta-analysis. J Ultrasound. 2015;18:91–99.
10. Partovi S, Kaspar M, Aschwanden M, Lopresti C, Shivanshu M, Uthoff H et al. Contrast-enhanced ultrasound after endovascular aortic repair—current status and future perspectives. Cardiovasc Diagn Ther. 2015;5:454–63.

## 3PM in dentistry

**Personalized approach to the dental caries prevention based on the electromagnetic field (EMF) exposure on polymer filling materials**

Moiseeva NS*^1^, Kunin AA^2^

^1^ Department of Oral and Maxillofacial surgery,

^2^ Department of Hospital Dentistry,

Voronezh State Medical University named after N.N. Burdenko

*Corresponding author: Natalia S. Moiseeva; e.mail: natazarova@yandex.ru

**Keywords**: predictive preventive personalized medicine, dentistry, European Dentistry Department, European Association for predictive, preventive and personalized medicine EPMA, dental caries, polymer-based filling materials, electromagnetic field, innovative technologies, tailored treatment, prognosis, enamel electrical conductivity, patient profiling and stratification

**Background**

The incidence rate of dental caries remains high at the population level reaching 98% globally [1]. A large number of medical technologies do not provide high quality and effective treatment and prevention, which can lead to the development of recurrent caries and the resulting complications of caries sometimes lead to disability in middle-aged and elderly people [2–3].

Comprehensive patient examination, using the patient’s profile, including their age, gender, presence/absence of general pathology and a whole rank of dental diagnostic methods, is a crucial link for effective individual prevention and treatment of dental caries. Statistical analysis of these parameters contributes to their stratification allowing picking out groups of patients with compensated and non-compensated caries forms with its substantial progression in the near future. The new method based on the microstructure modification of polymer-based filling materials using electromagnetic field exposure provides a comprehensive treatment approach allowing to enhance the ion-exchange processes in hard dental tissues and increase the level of enamel mineralization after the materials application, which will contribute to the prevention of recurrent caries [4–7]. Such prognosis approach to the diagnostics and treatment of dental caries correlates with the main concept of predictive, preventive and personalized medicine (PPPM) and has a high potential for clinical implementation.

The purpose of our scientific work is to study the preventive ability of dental filling materials with modified microstructure in dental caries progression.

**Materials and methods**

A personalized approach was applied to select patients with medium caries and without any general pathology for further investigations. The inclusion criteria were enamel resistance level, tooth plaque cariogenicity, and absence of periodontal disease. The study subjects are a contingent of 204 people aged 18–45 with a diagnosis of medium caries. Further, the contingent was examined to treat the teeth with filling material Charisma with EMF exposure (I – study group) and without EMF exposure (II – control group). Each study group material was placed in the working interpolar space for electromagnetic processing with the strength of 22 × 10^4^ A/m for 20 min [7].

This investigation was approved by the Ethical Committee for research of the State Funded Educational Institution of Higher Education «Voronezh State Medical University named after N.N. Burdenko» of the Ministry of Health of the Russian Federation (Protocol No. 1, February 25, 2016).

Dental examinations of patient’s health status for their inclusion to our investigation provide integral data concerning their age, gender, presence/absence of bad habits, quality of nutrition, level of enamel resistance, tooth plaque cariogenicity, etc. These parameters help us to create a specification rank among patients allowing picking out groups of patients with compensated and non-compensated medium caries. The inclusion and excludion criteria have been detailed by Moiseeva et al. [5].

Furthermore, those patients underwent dental caries treatment. At follow-up visits, all patients underwent complete dental examinations including the assessment of filling quality using the following methods: vital staining of enamel at the border of filling, quality of filling according to D.M. Karalnik and determination of the electrical conductivity of enamel in accordance with the R.G. Buyankina scale.

**Statistical analyses**

For statistical processing, a standard package of STATISTICA 8.0, Statsoft was used. The following parameters were calculated: mean and deviation, median and the standard error of the mean, asymmetry, minimum and maximum values of the data series, quantile value and quantile range. To compare the groups, we used a non-parametric Mann–Whitney test, Kruskal–Wallis test and a median test; the effect of multiple comparisons was taken into account. The differences were considered significant at *p* < 0.05.

**Results**

After 1 year in the study group, the quality of filling is maintained at an appropriate satisfactory level, in contrast to the control group, where clinical changes in the quality of the filling border are recorded in 7.8–9.8% of cases. The results of the electrical enamel conductivity are presented in Table 1 and in Fig. 1.

**Table 1** The results of progression and non-progression of recurrent caries by the electrical enamel conductivity data

**Table:** 

Observation stage	Group I with EMF exposure	Group II control without EMF exposure	*p* level
Mean ± S.E. 95% CI			
Before treatment	37.549 ± 0.467 (28.0/47.0)	39.167 ± 0.472 (29.0/48.0)	>0.05
After treatment	0.842 ± 0.058 (0.23/2.8)	1.183 ± 0.073 (0.35/3.10)	<0.05
After 1 month	0.924 ± 0.053 (0.3/2.7)	1.362 ± 0.068 (0.45/2.8)	<0.001
After 6 months	1.096 ± 0.052 (0.4/2.8)	1.897 ± 0.065 (0.78/3.2)	<0.001
After 1 year	1.208 ± 0.051 (0.51/2.87)	2.601 ± 0.051 (1.56/3.72)	<0.001

Abbreviations: Mean – average sample value; CI – confidence interval, S.E. – standard error


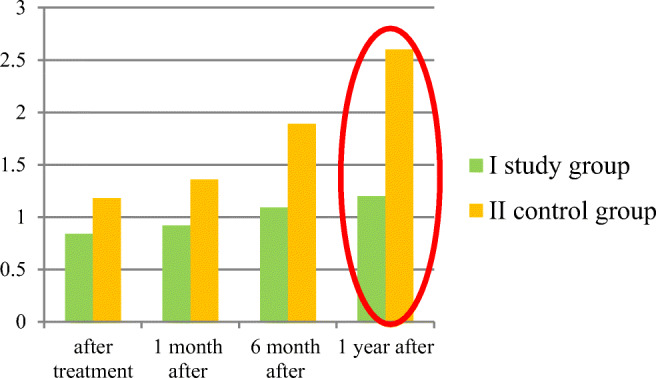


**Fig. 1** The graph of the electrical enamel conductivity

During the observation period, the electrical conductivity parameters of the enamel stabilized only in the study group, which indicates compensatory processes in the enamel and a tighter filling. However, in the control group, a significant increase in the digital values of electrical conductivity indicates an increasing violation of the marginal fit of filling material and may cause risks of recurrent caries in future (*p* < 0.01).

**PPPM related conclusion and expert recommendation**

The results of studies contributed to an increase in the biocompatibility of the material and increased strength properties with the structure of hard dental tissues, which brought us closer to creating filling material with improved adhesion and strength characteristics and increase the efficiency of caries treatment and contribute to dental caries prevention in a long-term period. Such a comprehensive approach to the diagnosis of the patient’s profiling status and further individualized caries treatment correlates with the main postulate of the European Association of Predictive, Preventive and Personalized Medicine (ЕРМА) [8]. Received results allows improving the quality of treatment and prevention of dental caries due to the activation and normalization of the ion-exchange processes in the enamel when using polymer filling materials with the electromagnetic field-modified microstructure.

The proposed concept allows creating an individual approach to the implementation of high quality treatment of caries in practice, which ensures maximum use of the principles of prediction and prevention. From the results, it is possible to recommend the use of a portable electromagnetic installation at a specialized company for practical dentistry to influence filling materials using an electromagnetic field. Thus, the developed method of changing the microstructure of polymer-based filling materials using an electromagnetic field is a fundamentally new approach protected by the patents and confirmed by statistics [4–7] that contributes to filling quality improvement and caries prevention.

Thus, better patient’s profile justification at the initial survey study promotes targeted treatment algorithms tailored to the person. Tailored treatment may lead to significantly improved results in the long-term. All these aspects provide the advanced technology of predictive, preventive and personalized dentistry in the framework of caries treatment.

**References**

1. Frencken J. Caries epidemiology and its challenges. Monogr Oral Sci. 2018;27:11–23. 10.1159/000487827.
2. Golubnitschaja O, Costigliola V. Dental health: EPMA recommendations for innovative strategies. EPMA J. 2014;5(Suppl 1):A119.
3. Golubnitschaja O, Costigliola V, Grech G. EPMA World Congress: traditional forum in predictive, preventive and personalised medicine for multi-professional consideration and consolidation. EPMA J. 2017;8(Suppl 1):1. 10.1007/s13167-017-0108-4.
4. Moiseeva NS, Kunin AA, Shabanov RA, Aliev NT. Electromagnetic influence on microstructural changes in dental filling materials: improvement in physical and mechanical properties. EPMA J. 2017;8(Suppl 1):S49.
5. Moiseeva NS, Kunin AA. Clinical and laboratory evaluation of microstructural changes in the physical, mechanical and chemical properties of dental filling materials under the influence of an electromagnetic field. EPMA J. 2018;9:47. 10.1007/s13167-018-0126-x.
6. Moiseeva NS, Kunin AA, Haytac CM. Efficiency of dental caries prevention with the use of polymer-based toothpastes modified by the electromagnetic field. EPMA J. 2018;9:319. 10.1007/s13167-018-0140-z.
7. Moiseeva NS, Kunin AA. Method of improving the adhesion and strength properties of dental filling materials and bonds. 2016. RF patent 2,594,255.
8. Golubnitschaja O, Baban B, Boniolo G, Wang W, Bubnov R, Kapalla M, et al. Medicine in the early twenty-first century: paradigm and anticipation – EPMA position paper 2016. EPMA J. 2016;7:23. 10.1186/s13167-016-0072-4.

**Toothache management utilizing an innovative approach according to predictive, preventive and personalized medicine**

Belenova I*^1^, Koretskaya I ^2^, Shabanov R^1^, Azarova O^1^, Belenov I^1^

**1** Department of Hospital Dentistry, Voronezh State Medical University named after N.N. Burdenko, Russian Federation

**2** Department of Propaedeutic Dentistry, Voronezh State Medical University named after N.N. Burdenko, Russian Federation

*Corresponding author: Prof. Dr. Irina Belenova

**Keywords:** predictive preventive personalized medicine, patient stratification, therapy prediction, prognosis, toothache prevention, repair enamel, proteinogenic acids, hyperesthesia management

Toothache is a strong, unpleasant sensation in a tooth, in a certain area of ​​the gum or in the entire jaw. It refers to those pains that do not allow you to sleep, eat food, speak and perform normal actions. In its strength and intensity, toothache can only be compared with the pain from the exit of stones from the kidneys. It will not be a mistake to say that the prevalence of dental diseases in the human population exceeds all other types of diseases. A large number of dental clinics and surgeries in any, even a small, city evidences this fact [1, 3].

“Teeth ache! Why?”—any person has faced a similar problem at least once in his life. Pain is never without a reason—this is a signal that some kind of malfunction has occurred in the organ. If the pain did not occur, it would be impossible to recognize the onset of the disease, and patients would seek help only in a very severe condition. Toothache can be classified based on a number of signs. 1. According to the pain dynamics. 2. According to the pain intensity. In the acute form, the pain is so strong that a person hurries to the dentist without hesitation. 3. According to the pain localization. Additionally, the causes of toothache are diverse: tooth decay, pulpitis, periodontitis, periodontitis, etc. In our research, we sought to understand why intact, “Hollywood white”, teeth ache after professional whitening. In pursuit of a Hollywood smile, some patients notice that they experience increased tooth sensitivity (hyperesthesia). This means that, together with the removed bacterial plaque, the mineralization of the hard dental coating, enamel, was disrupted.

In our work, we sought to understand what is happening with hard tooth tissues after professional whitening and how to prevent the negative consequences of this fashionable and sought-after procedure [2].

**The aim of the study** was to identify the effectiveness of amino acids application in order to restore the physiology of the tooth.

**Materials and methods** The study included 47 patients aged 19 to 45 years without severe dental and somatic pathology after professional whitening of the tooth enamel. The methods used in the study were:

- Clinical: survey, examination, determination of the index prevalence of hyperesthesia teeth (IRGZ), the index of intensity of hyperesthesia teeth (IIHS); test of enamel resistance (TER); clinical evaluation of the enamel remineralization rate (KOSRE test – Russian abbreviation).
- Clinical and laboratory: acid enamel biopsy. Performing the acid enamel biopsy in the groups of patients.
- Laboratory: scanning electron microscopy (SEM). For microscopic examination, teeth removed according to orthopedic and orthodontic indications were taken. The enamel was whitened in accordance with the instructions for use of the whitening system. The chips were prepared and investigated under 450- and 1000-fold magnification using a “Camscan S4” scanning microscope.

**Research results** The results of laboratory investigations. Changes in the structure of the tooth were traced when performing a comparative analysis of the spectrogram data of the enamel that was not exposed to whitening and after the procedure. After the exposure to peroxide compounds, enamel roughness and cavities appeared; usuras of tooth enamel, entrances to “enamel tunnels” became wider; this may be considered as a reason for the occurrence of teeth hyperesthesia in almost 100% of cases.

The entrances to the dentinal tubules became “open” and wider (Fig. 1).
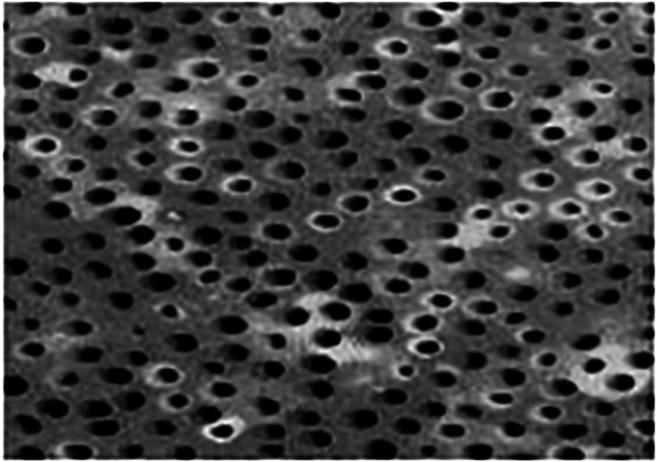


**Fig. 1** Dentinal tubules after application of whitening systems.

After whitening, the organic matrix of the tooth also suffered. Important components of the protection of the tooth glycoproteins and “cationic” protein were destroyed; this is clearly demonstrated on the slide.

**The clinical result after whitening in all patients was 6–8 tones, which was a demonstration of the effectiveness of the whitening system itself (Fig. 2).** However, after the procedure, almost all patients complained of tooth sensitivity.
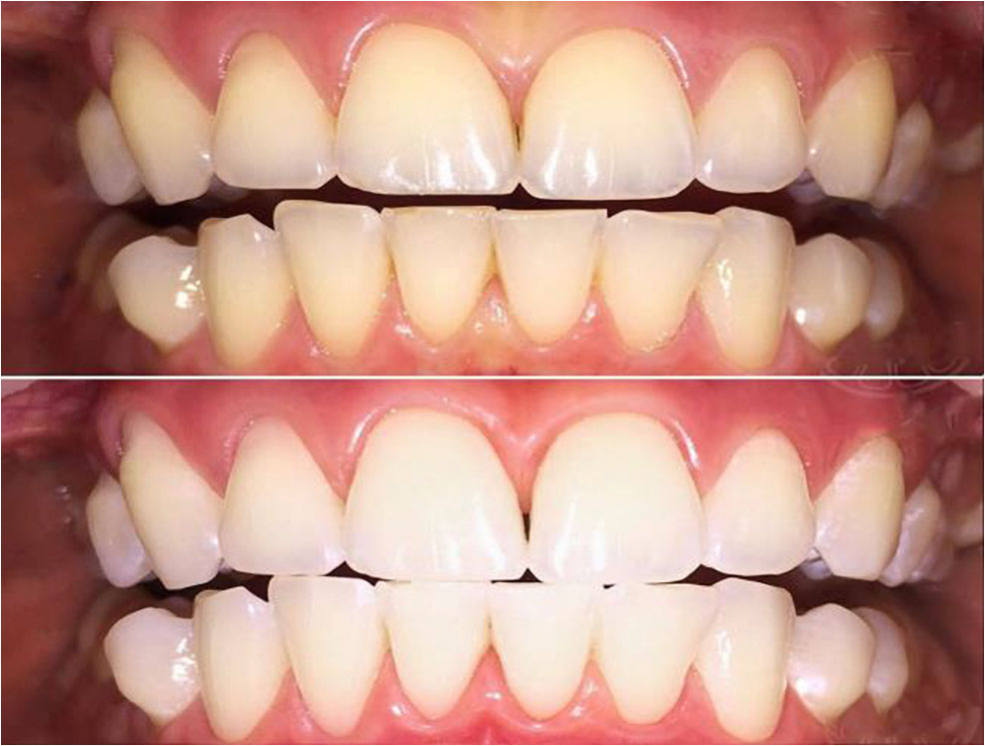
**Fig. 2** Clinical result after whitening

The enamel resistance according to the results of TER investigation, as well as the remineralization rate—according to the KOSRE-test results—decreased. The outcome of calcium and phosphorus ions increased significantly. Thus, immediately after the enamel whitening procedure, morphochemical changes appear: the enamel resistance decreases and the enamel acid compliance increases; this appears to be a prerequisite for the occurrence of carious and non-carious dental diseases. To restore the structure and function of the hard tissues of the teeth, we applied three preparations: 1. based on amino acids and calcium; 2. based on calcium and fluorine; 3. based on fluorine only.

We have obtained positive results in all study groups. Hyperesthesia of the teeth was eliminated. The enamel has become less acid compliant, i.e. its resistance has increased according to the results of the TER investigation and KOSRE test. The outcome of calcium ions and phosphorus ions decreased in the studied groups. All the drugs involved in the study are relevant for use immediately after the whitening of the enamel and are effective for reducing tooth sensitivity, increasing acid resistance and resistance of the enamel. The best results in a shorter period were obtained in the group using the preparation based on the amino acid complex.

**PPPM related conclusion and outlook** Toothache treatment is possible only within the framework of predictive, preventive and personalized medicine. To solve this problem, it is necessary to stratify patients by type of toothache. After professional whitening, patients with hyperesthesia should receive mandatory targeted prevention of enamel destruction and dental hyperesthesia. The best effect of the restoration of hard tissues of teeth was registered with the combined action of minerals and amino acids.

**References**

1. Kunin A, Polivka J Jr., Moiseeva N, Golubnitschaja O. "Dry mouth" and "Flammer" syndromes-neglected risks in adolescents and new concepts by predictive, preventive and personalised approach. EPMA J. 2018;9(3):307–317. 10.1007/s13167-018-0145-7.
2. Mozaffari MS, Emami G, Khodadadi H, Baban B. Stem cells and tooth regeneration: prospects for personalized dentistry. EPMA J. 2019;10(1):31–42. 10.1007/s13167-018-0156-4.
3. Tachalov VV, Orekhova LY, Isaeva ER, Kudryavtseva TV, Loboda ES, Sitkina EV. Characteristics of dental patients determining their compliance level in dentistry: relevance for predictive, preventive, and personalized medicine. EPMA J. 2018;9(4):379–385. 10.1007/s13167-018-0152-8.

**Detection of primary demineralization of tooth enamel as prevention of carious and non-carious diseases**

Kunin AA^1^, Ippolitov YuA^1^, Gavrish AV*^1^, Seredin P^2^, Kunin DA^3^

^1^Voronezh State Medical University, Studetcheskaya st. 10, Voronezh, Russia, 39400

^2^Voronezh State University, Voronezh, University sq. 1, 394018, Russia

^3^Family Medicine Center “Olympus Health”, Teatralnaya st. 23/1, 394036, Voronezh, Russia

*Corresponding author: Dr. Artem Gavrish, Department of Pediatric Dentistry with Orthodontics, Voronezh State Medical University, Studentcheskaya st. 10, Voronezh, Russia, 39,400; e.mail: artemgawrish@mail.ru

**Keywords**: predictive preventive personalized medicine, personalized diagnostics, demineralization, hypomineralization, CT-densitometry, personalized prevention

**Differential diagnosis of primary pathologies of hard tooth tissues as a problematic issue in modern dentistry**

Primary demineralization of the hard tissues of teeth is one of the most common groups of both dental pathologies and the general morbidity system, distinguished by an extensive list of etiological factors. However, the basis of these conditions is primarily a violation of enamel metabolism (the predominance of demineralization over remineralization) [1,2].

This group of nosologies includes primary subsurface demineralization (82.4%) [3], primary superficial demineralization (82%) [4] and primary superficial hypomineralization (15.1%) [5]. Although the onset of caries worldwide is associated with direct exposure to cariogenic microflora, often primary enamel damage is associated with chemical, mechanical or physical damage, as well as with other factors [1].

Reliable diagnostics include the key stages of primary prevention of pathologies of hard tissues of teeth. Many authors often turn to the fundamental aspects of the diagnostic problem of these nosologies [6], rarely addressing the problem of the clinical implementation of these methods in wide clinical practice.

In our work, we propose to optimize and deepen the diagnostic paradigms by introducing the previously unused CT densitometry technique into them.

The aim of the study was to identify early diagnostic criteria for the primary enamel demineralization necessary for the formation of preventive and diagnostic methods.

**Description of the study**

For the selection of patients and their distribution into groups, the methods of electrometric diagnostics of enamel (0–0.2 μA for intact enamel) and light-induced fluorescence (no spots on intact enamel) were used.

The investigated materials were obtained from 528 patients and divided into 4 groups according to the criterion of detected signs above indicated pathologies: control (135 persons, 25.57%), persons with primary hypermineralization (124 persons, 23.48%), persons with primary surface demineralization (121 persons, 22.92%) and persons with primary subsurface demineralization (148 persons, 28.03%).

The study was conducted according to analysis of the teeth images obtained by densitometry using cone-beam computed tomography.

**Results**

The study determined the normative values of clinically healthy enamel, corresponding to the range from 2800 to 4000 Hounsfields (HU) with a uniform distribution of mineral density.

In primary congenital hypomineralization of permanent teeth, CT densitometry values correspond to 1600–2000 HU with a uniform decrease in the mineral density of tooth enamel. With subsurface demineralization, the values go into the range from 1500 to 2600 HU with a characteristic bend of the CT densitometry section in the prismatic enamel section in the direction of decreasing values by more than 30%. In the case of surface demineralization, the values are in the range from 1600 to 2900 HU with a thinning of the enamel layer less than 1 mm.

The data presented are in the field of statistically significant values (*p* < 0.05).

**Data interpretation in the context of PPPM**

Densitometry results allow the differential diagnosis of pathologies of tooth hard tissues in the early, preclinical stages and maximize the effectiveness of preventive solutions. Ultimately, an integrated approach to diagnosis enables one to individualize the tactics of prevention for each individual patient, not only in the context of primary demineralization but also in the general dental status. This method enables one to more finely differentiate pathologies, personifying them for certain groups of patients. The CT densitometry method will enable creating an individual clinical picture and therapeutic plan based on the specific clinical conditions.

**Conclusions**

Based on the data obtained, it is possible to conduct a clear differential diagnosis of tooth enamel pathologies, since each of them corresponds to a clear range of radiological values. According to the severity of CT changes, one can put forward a prognosis of the severity and control the effectiveness of remineralizing therapy. A clear diagnosis makes it possible to conduct targeted therapy and prevention of each nosology, personalizing the approach to each patient. Such an algorithm for detailed preclinical diagnostics will allow moving away from generalized treatment regimens that do not take into account the individual characteristics of the clinical course of this group of pathologies [7].

*This work was supported by the grant 16-15-00003 of the Russian Science Foundation.*

**References**

1. EPMA World Congress: traditional forum in predictive, preventive and personalised medicine for multi-professional consideration and consolidation. EPMA J. 2017;8(Suppl 1):1–54. 10.1007/s13167-017-0108-4
2. Seredin P, Goloshchapov D, Ippolitov Y, Vongsvivut P. Pathology-specific molecular profiles of saliva in patients with multiple dental caries-potential application for predictive, preventive and personalised medical services. EPMA J. 2018;9(2):195–203. 10.1007/s13167-018-0135-9
3. Nor MAN, Chadwick BL, Farnell D, Chestnutt IG. The prevalence of enamel and dentine caries lesions and their determinant factors among children living in fluoridated and non-fluoridated areas. Commun Dental Health 2019;36(3):229–236. 10.1922/CDH_4522Nor08.
4. Kreulen CM, Van ‘t Spijker A, Rodriguez JM, Bronkhorst EM, Creugers NHJ, Bartlett DW. Systematic review of the prevalence of tooth wear in children and adolescents. Caries Res 2010;44:151–159. 10.1159/000308567
5. Zhao D, Dong B, Ren Q, Sun Y. The prevalence of molar incisor hypomineralization: evidence from 70 studies. Int J Paediatr Dent. 2017;28(2):170–9.
6. Rahul M, Ashima G, Krishan G, Veenu S. Analysis of bone mineral density and content in children with molar incisor hypomineralization using dual-energy X-ray absorptiometry scan: a pilot study. J Indian Soc Pedod Prev Dent 2019;37:282–5.
7. Tachalov VV, Orekhova LY, Isaeva ER, Kudryavtseva TV, Loboda ES, Sitkina EV. Characteristics of dental patients determining their compliance level in dentistry: relevance for predictive, preventive, and personalized medicine. EPMA J. 2018;9(4):379–385. 10.1007/s13167-018-0152-8.

**Preventive, Predictive and Personalized Medicine in Pediatric Dentistry: Caries Risk Assessment**

Merglova V*^1^, Hauer L^1^, Koberova-Ivancakova R^2^

^1^Department of Dentistry, University Hospital and Faculty of Medicine in Pilsen, Charles University, Czech Republic

^2^Department of Dentistry, University Hospital and Faculty of Medicine in Hradec Kralove, Charles University, Czech Republic

*Corresponding author: Vlasta Merglova, M.D., Ph.D., Associate Professor, Department of Dentistry, University Hospital in Pilsen; e.mail: merglovav@fnplzen.cz

**Keywords**: predictive, preventive and personalized medicine, pediatric dentistry, early childhood caries, caries risk assessment, risk factors, protective factors, dental caries prevention, dental recalls, Streptococcus mutans, nutritional habits

**Introduction**

Preventive, preventive and personalized medicine (PPPM) is an approach to medicine that proposes the customization of healthcare in which decisions and treatment are personalized based on the patient’s needs [1, 2]. The PPPM concept has an important impact on dental and oral health care [3].

Dental caries is the most common chronic childhood disease. Early childhood caries (ECC) is defined as tooth decay in preschool children. ECC is a biofilm-mediated, sugar-driven, multifactorial, dynamic disease that results in demineralization of dental tissues. Its development is determined by biological, behavioural and psychosocial factors linked to an individual’s environment [4]. The mean caries prevalence for 5-year-olds is 63%. Approaches to the reduction of ECC include early prediction, prevention and personalized dental care.

Caries risk assessment (CRA) is the process of establishing the probability of a child or group of children developing caries over a certain period, or the likelihood that there will be a change in size or activity of lesions already present. Caries risk factors are variables that are thought to cause the disease directly, or whose prediction has been shown to be useful.

The aim of the study was to identify caries risk factors among one-year-old infants.

**Materials and methods**

Our cross-sectional study is part of a long-term research and was performed at the Department of Dentistry and Obstetrics, University Hospital in Pilsen, Czech Republic. The study comprised Caucasian women who were attending the Obstetric Department for an antenatal check-up. The pregnant women were subsequently referred to the Dentistry Department in February, March and April 2018. The pregnant women were informed of the study; 142 of them agreed to participate in the study. During the dental visit all women were educated regarding the prevention of ECC. Six months after birth they received repeated information.

The research cohort comprised 116 infants: 54 boys (46.6%) and 62 girls (53.4%). At the age of one year, the infants and their mothers were invited to the CRA. The following data were recorded: diseases, fevers, antibiotics administration, nutrition and nutritional habits. The oral examination comprised an evaluation of enamel quality and collection of a saliva sample. The presence and amount of *Streptococcus mutans* (SM) in the saliva were detected by Dentocult Strip Mutans test, Orion Diagnostica™.

**Results**

The presence of caries risk factors is mentioned in Table 1.

**Table 1** The presence of caries risk factors

**Table:** 

Risk factor	Number of infants	%
Fevers	39	33.6
Frequent use of baby-bottle containing sweet liquids	29	25
Antibiotics treatment	28	24.1
Risk salivary SM levels	26	22.4
Nocturnal breastfeeding	25	21.6
Absence of oral hygiene	17	14.7
Presence of systemic diseases	15	12.9
Oral pathology	10	8.6

**Conclusions and PPPM-related expert recommendations**

The most obvious predictive factor for caries in the current study was the presence of fevers during the first year of age. This condition is connected with oral medicaments containing sugar and hyposalivation. Several studies have been conducted with the purpose of investigating the relationship between feeding practices and ECC [4]. The majority of studies have confirmed a high risk of ECC in cases of prolonged nocturnal breastfeeding and bottle feeding—this is knowledge that can be used for targeted prevention. Nocturnal breastfeeding was present in 21.6% of infants and bad habits connected with baby bottle in 25% of infants in the current study. An early colonization of the oral cavity with SM and high amounts of SM in saliva are associated with the development of ECC [4]. The finding that regular oral hygiene had not yet started in 14.7% of the given cohort of infants shows that not all previously educated mothers followed the recommendation to begin with oral hygiene when the first primary tooth appears in the oral cavity. In countries with advanced primary prevention, only 5% of one-year-old infants remain deprived of oral hygiene [5]. Of the infants, 24.1% were taking antibiotics during the first year of life and 12.9% of infants have a history of respiratory tract and ear infection and asthma. Being as asthmatic children may have an especially high risk of ECC due to the effect of their pharmacotherapy, mouth breathing and frequent intake of sugary drinks, it is important to pay attention to the patients’ individual profiles. Pathological oral conditions were described in 10 (8.6%) infants.

Targeted prevention of caries should take into account that ECC shares common risk factors with other non-communicable diseases associated with excessive sugar consumption, such as cardiovascular diseases, diabetes and obesity. A CRA should be carried out during the child’s first dental visit. Children with an individual profile that includes risk factors that are of predictive value for the development of caries should be targeted for appropriate preventive care with dental recalls based on individual need.

**Acknowledgements** Supported by a grant from the Ministry of Health of the CR – Conceptual Development of Research Organization Faculty Hospital in Pilsen – FNPI, 00669806.

**References**

1. Golubnitschaja O, Baban B, Boniolo G, Wang W, Bubnov R, Kapalla M, et al. Medicine in the early twenty-first century: paradigm and anticipation - EPMA position paper 2016. EPMA J. 2016; 10.1186/s13167-016-0072-4.
2. Golubnitschaja O, Watson ID, Topic E, Sandberg S, Ferrari M, Costigliola V. Position paper of the EPMA and EFLM: a global vision of the consolidated promotion of an integrative medical approach to advance health care. EPMA J. 2013; 10.1186/1878-5085-4-12.
3. Golubnitschaja O. Time for new guidelines in advanced healthcare: the mission of The EPMA Journal to promote an integrative view in predictive, preventive and personalized medicine. EPMA J. 2012; 10.1186/1878-5085-3-5.
4. Tinanoff N, Baez RJ, Guillory CD, Donly KJ, Feldens CA, McGrath, et al. Early childhood caries epidemiology, aetiology, risk assessment, societal burden, management, education and policy: global perspective. Int J Paediatr Dent. 2019; 10.1111/ipd.12484.
5. Hultquist AI, Bägesund M. Dentin caries risk indicators in 1-year-olds. A two year follow-up study. Acta Odontol Scand. 2016; 10.1080/00016.357.2016.1227085.

**Emotional self-regulation and its relation to quality of individual oral care, application of 3PM concepts in dentistry**

Tachalov VV*^2^, Orekhova LYu^1^,^2^, Kuchumova ED^2^, Sitkina EV^3^, Iamanidze NA^1,2^, Grinenko EE^1,2^

1. City Periodontal Center “PAKS”, St. Petersburg, Russia

2. Therapeutic Dentistry and Periodontology Department, Pavlov First Saint Petersburg State Medical University, St. Petersburg, Russia

3. Department of General and Clinical Psychology, Pavlov First Saint Petersburg State Medical University, St. Petersburg, Russia

*Corresponding author: Vadim V. Tachalov, Ph.D., Associate Professor, Therapeutic Dentistry and Periodontology Department, Pavlov First Saint Petersburg State Medical University, 6/8 Lva Tolstogo Street, 197022, St. Petersburg, Russia; e.mail: tachalov@mail.ru

**Keywords:** Compliance, treatment adherence, individual oral hygiene, psychological specifics of patients, relevance of treatment, predictive preventive personalized medicine, personalized hygiene, 3PM, individualized patient profile, improved outcome, preventive predictive periodontology, preventive dentistry, dental disease prevention, personalized oral care, pshylogical approach in periodontology

**Introduction**

3PM concepts. Oral diseases such as dental caries and periodontal diseases can lead to eventual tooth loss. Worldwide severe periodontal disease in upward of 20% of adults, results in tooth loss and about 30% of people aged 65–74 have no natural teeth [1, 2].

In Europe and the USA, dental caries remains a major problem [3, 4].

The novel PPPM concepts have been suggested by EPMA to benefit the impacts on the oral and dental health [5–7].

For many acute and chronic disorders, the current health care outcomes are considered inadequate [7, 8].

The purpose of the study was to investigate the impact of emotional self regulation upon the quality of individual oral care to establish a personalized approach in dental prophylaxis.

**Methods**

A total of 99 persons (62 women and 37 men) 19 to 67 years of age participated in the study which was conducted with the use of the following psychodiagnostics methods: (1) Leonhard-Smishek Characterological Questionnaire (version for adults); (2) Individual Typological Questionnaire by L.N. Sobchick (ITQ). The Green—Vermillion index was defined at the beginning of the study and upon its completion (in a month). After the 1st examination, professional oral hygiene was performed on each patient. Using this index, the examined patients were divided into two groups by the dynamics of oral care performance during the treatment period. The former group patients demonstrated negative dynamics—deterioration of their oral cavity status (increase of the Green—Vermillion index by the end of the study). The latter group patients demonstrated positive dynamics—improvement of their oral hygiene status (decrease of the Green—Vermillion index by the end of the study).

**Results**

The study revealed statistically significant differences between Groups 1 and 2 in terms of frequency of tooth brush replacements. Group 1 patients replaced their tooth brushes less often (0.94 ± 0.42) than Group 2 patients (1.34 ± 0.66).

Statistically significant differences between Groups 1 and 2 were also defined by the level of patients’ emotional lability (disequilibrium): in Group 1, affective-exalted type of a person was registered more often (19.05 ± 4.85) than in Group 2 (13.30 ± 6.07).

Statistically significant differences between Groups 1 and 2 were also defined by the parameter “hyperthymic type of a person”: the value of this indicator was higher in Group 1 (16.05 ± 3.17) than in Group 2 (12.44 ± 6.0).

In Group 1 there were more patients of a cyclothymic type of a person (16.4 ± 6.19) than in Group 2 (12.3 ± 4.9).

Statistically significant differences between Groups 1 and 2 were also assessed according to the scale “Extraversion”: the highest value was registered in Group 1 (6.5 ± 1.8), and the lowest was registered in Group 2 (4.7 ± 2.4), i.e. more sociable and extroverted patients were observed in Group 1 than in Group 2.

**3PM related conclusions and outlook**

The emotional lability, introversion, increased emotional self-regulation and anxiety (concern) about the treatment results are linked to the quality of individual oral care.

This knowledge could help doctors in predicting the result of the treatment and making it most effective by increasing patients’ compliance level and attitude towards their health and treatment through a psychological approach [9]. Additionally, it is very important that a disease can be predicted by self-control methods and prevented or treated in the early stage with positive therapeutic effect [10].

**References**

1. World Health Organization. Oral disease burdens and common risk factors. 2018. http://www.who.int/oral_health/disease_burden/global/en/.
2. Mozaffari MS, Emami G, Khodadadi H, et al. Stem cells and tooth regeneration: prospects for personalized dentistry. EPMA J. 2019;10, 31–42. 10.1007/s13167-018-0156-43.
3. World Dental Federation. Promoting oral health through fluoride. Int Dent J. 2018;68:16–7.
4. Centers for Disease Control and Prevention. Health, United States, 2016. https://www.cdc.gov/nchs/data/hus/hus16.pdf#060.
5. Moiseeva NS, Kunin AA, Haytac CM. Efficiency of dental caries prevention with the use of polymer-based toothpastes modified by the electromagnetic field. EPMA J. 2018;9:319–329. 10.1007/s13167-018-0140-z
6. Tachalov VV, Orekhova LY, Kudryavtseva TV, Isaeva ER, Loboda ES. Manifestations of personal characteristics in individual oral care. EPMA J. 2016;7:8. 10.1186/s13167-016-0058-2. CrossRefPubMedPubMedCentralGoogle Scholar
7. Almendra Mattos RM, de Mendonça RMH, dos Santos Aguiar S. Adherence to dental treatment reduces oral complications related to cancer treatment in pediatric and adolescent patients. Support Care Cancer 2020;28, 661–670. 10.1007/s00520-019-04857-3
8. EPMA World Congress: Traditional Forum in Predictive, Preventive and Personalised Medicine for Multi-Professional Consideration and Consolidation. EPMA J. 2017;8, 1–54. 10.1007/s13167-017-0108-4
9. Tachalov VV, Orekhova, LYu, Isaeva ER, et al. Characteristics of dental patients determining their compliance level in dentistry: relevance for predictive, preventive, and personalized medicine. EPMA J. 2018;9, 379–385. 10.1007/s13167-018-0152-8
10. Kunin A, Polivka J Jr., Moiseeva N, Golubnitschaja O. “Dry mouth” and “Flammer” syndromes - neglected risks in adolescents and new concepts by predictive, preventive and personalised approach. EPMA J. 2018;9(3):307–17.

**The effects of changes in the microstructure of polymer-based toothpastes after the electromagnetic field exposure on predictive caries prevention**

Kunin AA^1^, Moiseeva NS*

^1^ Department of Hospital Dentistry,

^2^ Department of Oral and Maxillofacial surgery,

Voronezh N.N. Burdenko State Medical University

*Corresponding author: Associate Prof. Dr. Natalia S. Moiseeva; e.mail: natazarova@yandex.ru

**Keywords:** predictive preventive personalized medicine, dentistry, European Dentistry Department, European Association for predictive, preventive and personalized medicine EPMA, dental caries prevention, polymer-based toothpastes, electromagnetic field, innovative technologies, therapy effectiveness, progression, ion-exchange enamel processes

**Background**

Despite the obvious success in caries prevention worldwide the incidence of caries rate remains high and results not only in a medical but also in a global social problem that requires an immediate solution.

Determining the predictor factors of initial caries progression is a key point of its successful prevention and for a personalized treatment approach which fits with the main concept of predictive preventive personalized medicine (PPPM). Therefore, a promising area of preventive dentistry is the development of new highly effective methods of enhancing the remineralizing activity of prophylactic means and the search for more effective ways for dental caries prevention [1–4]. Those methods may significantly contribute to the paradigm change from reactive to predictive, preventive and personalized dentistry as well as healthcare systems at large. A prospective research study performed at the Voronezh State Medical University named after N.N. Burdenko included a comprehensive patient’s dental status profiling and further applied modified and non-modified polymer-based toothpastes for early caries prevention [5, 6]. It was concluded that improving the microstructure of polymer-based toothpastes using an electromagnetic field could provide a highly effective caries prevention by the targeted enamel remineralization and promote a new quality level of dental treatment oriented on dental caries prediction [5–8].

**Materials and methods**

Study subjects represented a randomly selected contingent of 62 patients aged from 18 to 35 years old with intact teeth and without any general pathology, divided into 2 equivalent groups, who underwent 1-month controlled tooth brushing with (group 1) and without (group 2) electromagnetic field exposure.

The scientific study was approved by the local committee for ethics in research of the State Funded Educational Institution of Higher Education «Voronezh N.N. Burdenko State Medical University» of the Ministry of Health of the Russian Federation (Protocol N 1, February 25, 2016).

Dental examinations of patient’s health status for their inclusion to our investigation provided integral data concerning their age, gender, presence/absence of bad habits, quality of nutrition, level of enamel resistance, tooth plaque cariogenicity, etc. Those parameters helped us to create a specification rank among young people allowing picking out groups of patients with potential risk factors to caries appearance and its progression in the near future. The inclusion and excludion criteria have been detailed by Moiseeva et al. [5].

Furthermore, those patients underwent controlled tooth brushing. To assess the effectiveness of the use of toothpaste with the electromagnetic field in terms of predictive caries prevention at follow-up visits, all the patients underwent complete dental examinations and several functional tests: Functional Enamel Resistance (FER-test), Cariogenicity of Dental Plaque by Hardwick J.L., Manly E.B. and the Electrical Enamel Conductivity using the DentEst device (Geosoft Dent).

**Statistical analyses**

For statistical processing of the study results, a standard package of STATISTICA 8.0 software programs by Statsoft was used. The following parameters were calculated: mean and the standard error of the mean. To compare the groups, we used a non-parametric Mann–Whitney and Kruskal–Wallis test. The differences were considered significant at *p* < 0.05.

**Results**

After the controlled tooth brushing, the level of caries-resistance in group 1 significantly increased from 21.8 ± 3.0% to 5.8 ± 0.6% (*p* < 0.01) and in group 2 from 20.8 ± 2.8% to 13.2 ± 2.1% (*p* < 0.05), which indicated a more significant remineralizing effect of the toothpaste with the electromagnetic field compared with group 2 (Fig. 1).

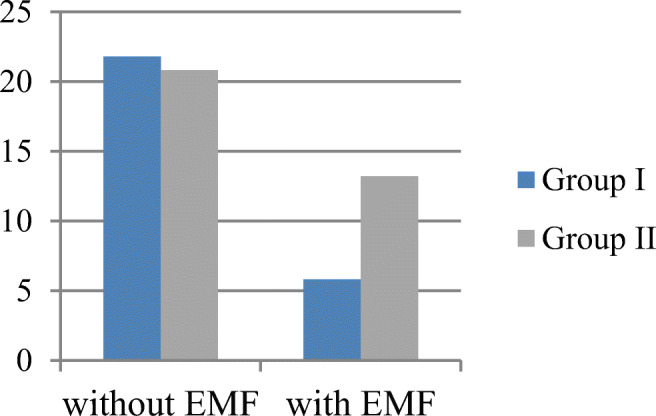



**Fig. 1** The functional enamel resistance before and after the electromagnetic field exposure

The tooth plaque cariogenicity in group 1 significantly decreased after the controlled brushing of teeth from 21.8 ± 2.3% to 9.2 ± 0.5% (*p* < 0.01), and in group 2 from 21.6 ± 2.2% to 15.0 ± 1.2% (*p* < 0.05), which is due to the prolonged remineralizing action of toothpaste with the electromagnetic field (Fig. 2).

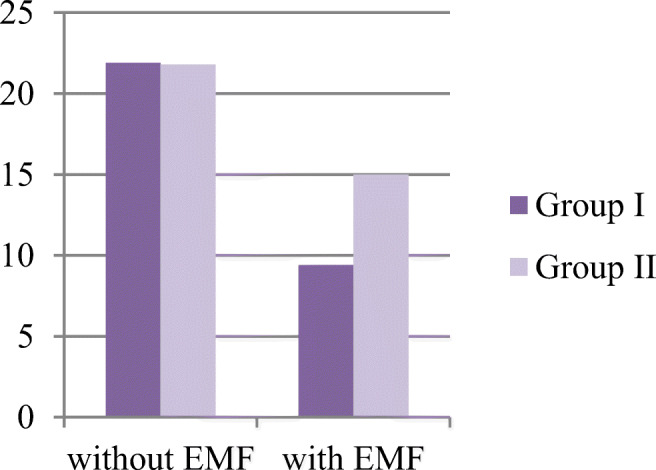



**Fig. 2** The tooth plaque cariogenicity before and after the electromagnetic field exposure

The electrical enamel conductivity decreased significantly in group 1 from 0.8 ± 0.1 μA to 0.1 ± 0.01 μA (*p* < 0.01), and in group 2 from 0.7 ± 0.1 μA to 0.4 ± 0.05 μA (*p* < 0.05), which indicated a more effective caries prediction in group 1 (Fig. 3).

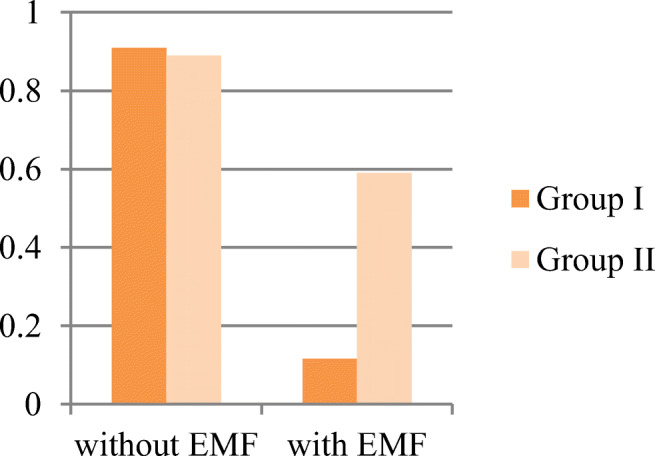



**Fig. 3** The electrical enamel conductivity

The positive preventive effect of the controlled tooth brushing are due to the fact that its mineral and polymer components structured under the influence of an electromagnetic field provide for better penetration into the enamel ensuring a prolonged remineralizing effect in terms of highly effective caries prediction (р = 0.01).

Thus, the results obtained for electrical conductivity confirmed a significant decrease in the parameters to 0–0.2 μA and the retention of such an effect for three months, which indicates the effectiveness of preventive measures and enables us to recommend the method of improving the microstructure of polymer toothpastes for practical dentistry.

**PPPM related conclusion and recommendation**

Thus, the obtained results indicate a positive remineralizing effect of the toothpastes modified by the electromagnetic field microstructure, both on the enamel resistance level and on the dental plaque micro flora, including the elimination of its cariogenic activity, promoting highly effective preclinical caries prevention and prediction of its progression at the early stage in a long-term period which correlate with the main concepts of predictive, preventive and personalized (3P) medicine (PPPM) and will have a positive effect on the healthcare economy in terms of cost-effective targeted prevention and reducing financial costs for the treatment of dental caries complications. Moreover, such integral dental health status investigation allows distributing the contingent according to the level of caries severity and risk factors, which will promote early individualized preventive therapy.

The results of the present study become the basis for the formation of recommendations on the use of electromagnetic field-modified polymer-based toothpastes as effective preventive technology for practical healthcare as well as for their inclusion in dental diseases prevention programs of Medical Universities worldwide.

In conclusion, the present study has introduced a new highly effective method of dental caries prevention that enables changing the generally accepted paradigm for dental caries treatment to early caries prediction and prevention.

**References**

1. Nor NAM, Chadwick BL, Farnell D, Chestnutt IG. The prevalence of enamel and dentine caries lesions and their determinant factors among children living in fluoridated and non-fluoridated areas. Community Dent Health. 2019 Aug 20. 10.1922/CDH_4522Nor08.
2. Golubnitschaja O, Costigliola V. Dental health: EPMA recommendations for innovative strategies. EPMA J. 2014;5(Suppl 1):A119.
3. Golubnitschaja O, Baban B, Boniolo G, Wang W, Bubnov R, Kapalla M, et al. Medicine in the early twenty-first century: paradigm and anticipation – EPMA position paper 2016. EPMA J. 2016;7:23. 10.1186/s13167-016-0072-4.
4. Golubnitschaja O, Costigliola V, Grech G. EPMA World Congress: traditional forum in predictive, preventive and personalised medicine for multi-professional consideration and consolidation. EPMA J. 2017;8(Suppl 1):1. 10.1007/s13167-017-0108-4.
5. Moiseeva NS, Kunin AA. Clinical and laboratory evaluation of microstructural changes in the physical, mechanical and chemical properties of dental filling materials under the influence of an electromagnetic field. EPMA J. 2018;9:47. 10.1007/s13167-018-0126-x.
6. Moiseeva NS, Kunin AA, Haytac CM. Efficiency of dental caries prevention with the use of polymer-based toothpastes modified by the electromagnetic field. EPMA J. 2018;9:319. 10.1007/s13167-018-0140-z.
7. Kunin AA, Moiseeva NS, Mekhantieva LE. Improving the effectiveness of dental caries prevention using therapeutic toothpastes. EPMA J. 2017;8(Suppl 1):S50.
8. Moiseeva NS, Kunin AA. Method of improving the adhesion and strength properties of dental filling materials and bonds. 2016. RF patent 2594255.

**Modified diode red light for caries prevention**

Kunin AA*^1^, Lukuanovich PA^2^, Kunin DA^3^, Yakunina LA^3^

^1^Voronezh, Russia N.N. Burdenko State Medical Academy

^2^Department of Mathematical Physics, Voronezh State University, Voronezh, Russia

^3^Olymp Zdorovia Clinic, Voronezh, Russia

*Corresponding author: Prof. Dr. Anatolij Kunin, Dentistry Faculty, Departments of Maxillofacial Surgery and Hospital Dentistry, Voronezh N.N. Burdenko State Medical University, Voronezh, Russia; e.mail: natazarova@yandex.ru

**Keywords:** predictive preventive personalized medicine, individualized prevention, caries prevention, light-induced fluorescence, caries media, light treatment, laser, personalized therapy, diode red light, dentine decay, enamel remineralization

**Evaluation of the value and organization of the study light factors for the prevention of caries**

It is impossible to overestimate the influence of light factors on metabolic processes in the hard tooth tissues. An integral effect of using fundamental physical and mathematical research and their practical application predicts not only a positive effect of prevention and treatment of caries but also contributes to the achievement of a positive integral clinical result [1,2,3,4,5]. Cooperation of the Department of therapeutic Dentistry of the Voronezh State Medical University named after N. N. Burdenko and the Physical Department of Voronezh State University allowed conducting a series of research that determined the influence of various parameters of light, its induction, pulsation and its combinations on the level of metabolic processes in hard tooth tissues and the effectiveness of caries prevention.

**What was the scientific basis of the study?**

Previously, in order to activate the metabolic processes of the tooth, helium–neon lasers were used and later the effectiveness of light modulated light sources was proved with parameters from 625 nm to 760 nm. Analysis of the preliminary results on 22 patients with intact teeth and dentine decay (caries media) showed an advantage of using the wave length 760 nm. Determining the most optimal light parameters to achieve maximum effectiveness of caries prevention, is the basis for understanding predictive, preventive and personalized medicine [1,6].

**Methods**

For this research, 152 patients were selected; 78 (51.3%) were men and 74 (48.7%) were women, aged between 20 and 25; 36 (23.7%) of them had intact teeth and 116 (76.3%) were diagnosed with dentine decay (caries media) after filling.

The method of primary prevention for intact teeth and secondary prevention (for elimination of reccurent caries) consisted of optical emission at 760 nm for 76 patients (50%) with the effect of light and 76 patients (50%) without it.

Positive results of the prevention of caries (the level of metabolism of the teeth was not reduced in the tooth and appearance of active enamel remineralization was detected) were evaluated in terms of its electrical conductivity and light-induced fluorescence. Light exposure was performed in 1.5 min daily sessions for 3 days.

**Results**

In the light therapy group, electrical conductivity indicators and light-induced fluorescence indicators did not show the effect of enamel demineralization in both groups for the duration of one year. The start indicators of enamel conductivity were from 0 to 25, and when dentine decay (caries media) was diagnosed, enamel conductivity was from 3.85 to 4.5. Light-induced fluorescence of intact enamel did not show the appearance of stain. Teeth, diagnosed with caries media bordering with the caries cavity showed as a shadow stain. This indicator did not change after a year. Furthermore, in the teeth diagnosed with dentine decay, the spots disappeared, and the conductivity of enamel was 0.2 ± 0.05.

Caries cavities did not occur throughout this period, and the 100% effectiveness of primary and secondary caries prevention was proved.

**What was observed in the control group?**

Over a period of one year, out of 76 patients diagnosed with dentin decay and not exposed to the effect of light on the enamel of the teeth, six (7.9%) patients were found to have developed new caries cavities. Two of those (33.3%) six cases of newly developed cavities were in the group of intact teeth, and four (66.7%) cases were in the group of the patients diagnosed with moderate caries.

**Conclusions**

The optimal effect of the prevention in the group of patients with intact teeth exposed to light should be viewed as a new major preventive program in dentistry. According to academic sources, as of today, such results had been impossible to achieve. In the group of patients diagnosed with dentine decay, the absence of recurrence of caries, due to the use of light in the course of treatment, showed the adequacy of this type of treatment in normalizing the metabolic processes in the hard teeth tissues that were destroyed by the carious process and the trauma caused by teeth preparation during the main method of caries treatment, which was proved in early research. It was impossible to eliminate these negative factors without the light treatment.

Using clinical, physical and mathematical methods to study the influence of the modified red light on the hard tissues of the teeth showed the great effect of normalization of the metabolic process in the teeth. Additionally, as a result, it showed us the optimization of caries prevention at the initial stage of enamel demineralization, which is the most important indicator being studied in predictive, preventive and personalized medicine [6].

**How to use these findings in the future?**

This apparatus (“STOMASvet”), developed by our team and certificated in Russia, should be certified in other countries and an international patent should be obtained. Furthermore, we view it as necessary to train specialists to give master classes, organized with EPMA. Such training sessions have already been held in cities such as Voronezh and Lipetsk (Russia).

Implementation of the developed light characteristics for prevention and prophylactic treatment is based on 10–15 years of preliminary physico-mathematical digital research. Thus, the breach between medicine and fundamental research is eliminated with the help of modern methods, such as (atomic-force microscopy, scanning microscopy, plane microscopy), which led not only to new results but also verified their relevance for caries prevention.

**References**

1. Kunin AA, Belenova IA, Ippolitov YA, Moiseeva NS. Predictive research methods of enamel and dentine for initial caries detection. EPMA J. 2013;4(1):19. 10.1186/1878-5085-4-19
2. Kunin AA, Moiseeva NS. A novel approach for detection of primary tooth caries based on the light influence foundation EPMA J. 2014;5(Suppl 1):A123. 10.1186/1878-5085-5-S1-A123
3. Kunin DA. Fundamental basis of patient-specific caries prevention. EPMA J. 2014;5(Suppl 1):A118. 10.1186/1878-5085-5-S1-A118
4. Kunin AA, Belenova IA, Kupets TV. Evaluating the effectiveness of structural and metabolic tooth enamel reparation by magnesium-calcium remineralizing complex. EPMA J. 2014;5(Suppl 1):A122. 10.1186/1878-5085-5-S1-A122
5. Kunin AA, Belenova IA. Innovative aspects of tooth ultrastructure and ultra-chemistry: unraveling of caries mechanisms development prevention strategies. EPMA J. 2014;5(Suppl 1):A124. 10.1186/1878-5085-5-S1-A124
6. Kunin A, Polivka J Jr., Moiseeva N, Golubnitschaja O. “Dry mouth” and “Flammer” syndromes-neglected risks in adolescents and new concepts by predictive, preventive and personalised approach. EPMA J. 2018;9(3):307–317. 10.1007/s13167-018-0145-7.

## 3PM in inflammatory diseases

**Mechanosensing and mechanotransduction as overlooked biological features potentially instrumental for advanced diagnostics and treatment of inflammation in the context of 3PM**

Nardini C*^1,2,3^, Manni L^4^

^1^Department of Laboratory Medicine, Division of Clinical Chemistry Karolinska Institute, Sweden

^2^Bio Unit, Scientific and Medical Direction, SOL Group, Italy

^3^IAC “Mauro Picone”, Consiglio Nazionale delle Ricerche CNR Italy

^4^Institute of translational pharmacology, Consiglio Nazionale delle Ricerche (CNR), Italy

*Corresponding author: Dr. Christine Nardini; e.mail: christine.nardini.rsrc@gmail.com, luigi.manni@ift.cnr.it

**Keywords**: predictive, preventive and personalized medicine, patient stratification; individualized patient profiles; risk, modifiable and preventable factors; multi-omics, genetics; epigenetics; big data analysis; phenotyping, gut-intestinal microbiome, autonomic nervous system, computational approaches, network theory, inflammation, wound healing, inflammatory reflex, mechanotransduction, non-communicable diseases

**Background**

Predictive, preventive and personalized medicine (3PM/PPPM [1]) represents a necessary change in paradigm in medicine, to enable and grant efficient health services to all citizens, in a society where a greatly extended lifespan is not accompanied (yet) by a corresponding health span. Among the many changes in mentality, which this new paradigm entails, is the urgent duty to move away from the current reactive medical paradigm, offering -viceversa- cost-effective prevention, precise prediction and sufficient personalization. Among the areas of medicine that most crucially need this change is the vast and fragmented area of specialties that are concerned with non-communicable diseases (NCDs), characterized by chronic low-grade inflammation and wound healing gone awry [2]. Fragmentation is among the issues that force patients classification into categories (diabetes, rheumatoid arthritis -, inflammatory bowel disease -IBD, to name a few) that may also benefit from a more holistic approach to the low grade inflammatory and impaired wound healing, going beyond specialization, taking advantage of subtle phenotypic characteristics able to preventively identify non-physiological mechanisms at work, following the exemplar of Flammer syndrome [3] an approach promising to benefit a number of related syndromes.

**Results**

Mechanosensing and mechanotransduction are likely to represent overlooked biological features in inflammation, particularly in the potential they have to address chronic inflammation. Given the overwhelming number of individuals affected by chronic inflammation (roughly 40 million deaths per year, corresponding to 71% of all deaths of individuals between the age of 30 and 69 [4]), all approaches susceptible to contribute to the control of this pandemic are of interest in 3PM.

We here present results on rheumatoid arthritis (RA), a well-known model for NCD, with a global incidence ranging from 1 to 4% worldwide, affecting mostly women (70%) and with unclear etiology, although genetic and epigenetic alterations, as well as modifications of the gut-intestinal microbiome composition (dysbiosis) are known characteristics of the disease [5].

In addition to chronic inflammation, impaired wound healing (also known as epithelial mesenchymal transition type 2) is an accompanying marker of such diseases. Wound healing physiological progression is characterized by four main phases [2], the first, very early consists of non-transcriptional signalling, quickly followed by blood clotting and transient acute inflammation, later replaced by longer lasting regeneration and remodelling phases. Wound healing is elicited in response to stimuli perceived as injuries that can be mechanical, among others. In such cases, the early phases of wound healing overlap/are preceded by mechanosensing and mechanotransduction phenomena that convert the mechanical stimulus into a biochemical signal.

Mainstream treatments of RA consist of disease modifying anti rheumatic drugs (DMARDs) divided into conventional (cDMARDs), including methotrexate (MTX) a low cost, anticancer drug given in low doses, with immunodepressive effects and toxicity, effective in 60% of patients, and biologics (bDMARDs) more targeted therapies, highly expensive, still immunodepressive, and whose efficacy must be empirically tested on patients that are non-responders to MTX [6].

Therefore, with the ambition to better treat more RA patients, in addition to the design and/or repurposing of drugs, non-pharmacological approaches are also being investigated. A surge of non-pharmacological therapies is now investing medicine; these include, for example, fecal microbiota transplantation (FMT, [7]), mechanical subcutaneous stimulations, mechanically induced regeneration [8] and electrical stimulation [9].

In this context, we report and frame within 3PM the results of our studies conducted to achieve a better, molecular understanding of the effects elicited by a mechanical subcutaneous stimulation, making use of unbiased screenings (omics) collected at different time points and from different tissues in order to offer a systemic view on a systemic disease that also have the potential to enable technological development for the continuous real-time monitoring of biological therapeutic effects of mechanical stimulations [10].

The results [11], conducted on animal models, completed by control arms and duplicated experiments, produced a series of relevant information for the implementation of 3PM. Frist of all, after validation of the effectiveness of the approach by standard phenotypic traits assessment (i.e. paws’ thickness), it has been possible to show that mechanical subcutaneous stimulations released in the dorsal area of animals (local) can elicit wound healing responses that are systemic, i.e. measurable in blood (peripheral blood mononuclear cells, PBMC), stably over time after a transitory phase where inflammation was dampened. Second, not only can blood be a mirror of the return to homeostasis lead by reduced inflammation and wound healing activity but the composition of the gut-intestinal (GI) microbiome also undergoes a significant modification towards a more eubiotic composition and increased abundance of genera (such as Roseburia) producing anti-inflammatory metabolites (butyrate).

Indeed, we have shown [12] that computational approaches can enhance and ease such type of analysis, demonstrating that the algorithmically joint analysis of two easily accessible bodily compartments (blood and feces) can provide a better insight into the mechanisms at work under these conditions (RA as a model of NCDs), showing that in addition to the inflammatory and wound healing functions, mechanical stimulation affects the sphingolipid metabolism, known to be altered in RA.

**Conclusions and expert recommendations**

The complexity of the mechanisms revealed, and in particular the overlooked connections between mechanotransduction, wound healing, inflammation and associated dysbiosis, reinforces the necessity of a holistic, systemic approach to malady. In particular, the known connection between the autonomic nervous system control of inflammation (including the inflammatory reflex [13]) as well as the connection between the gut-intestinal microbiome and the central nervous system (gut-brain axis [14]), ask for a revision of our understanding of inflammation, accepting that the biological functions involved—and possibly manipulable in view of a return to homeostasis—are more numerous and complex than acknowledged so far, and better described by a greater inflammatory pathway [15]. In short, we argue that an ample revision of all mechanisms known to impact directly on the control of inflammation, collected with the usage of tools typical of computational biology—including, in particular, network theory—will enable not only a better understanding of chronic inflammation and wound healing gone awry in NCDs but will also offer operational cues to design new therapies. This has been the case, for example, in biolectronic medicine [9], which requires the implant of a device, with the associated economical and psychological costs; therefore, it is useful to explore all means (mechanical stimulation, gut microbiome manipulation, etc.) to access and modify the inflammatory system, owing in particular to all technological advances (including NGS and mass spectrometry-based technologies) enabling efficient multiomic approaches in easy-to-access body fluids [16].

Understanding the greater inflammatory pathway in detail [15] automatically enables 3PM, as expliciting the static and dynamic factors affecting proper wound healing implies to devote attention to the following aspects of patients life: (i) contingent biochemical factors: high glucose levels, hypoxia, pre-existing infection, macrophage activity (impaired by corticosteroids), bisphosphates, denosumab and biologicals; (ii) contingent environmental factors: moisture, diabetes mellitus, oedema, ethanol abuse, smoking, under nutrition, omega-3 fatty acids intake and lack of vitamins; (iii) genetics and epigenetic: causes for impaired WH.

As recalled above, the abundance and complexity of this information can be leveraged with adapted computational tools and mathematical theory, namely network approaches. Having such a systemic knowledge of individuals, automatically enables personalized therapy with respect to the return to homeostasis of inflammation, via the study of patients personal history, granting personalized acts to enable the return to homeostasis (diet, lifestyle, drug therapy revision); better patients stratification, granted by a deeper knowledge of the specific pathways gone awry; and last but not least, preventive approaches that enable physicians to choose more adapted therapies in cases of comorbidities, and continuous monitoring of patients propension towards impairment of wound healing and inflammation via adapted questionnaires and tests, carefully designed to assess personalized risk.

Given the numbers at stake and the difficulties that the current medical paradigm shows in addressing the NCD pandemic, all cues susceptible to enhance prevention (questionnaires) and prediction (phenotyping and multi-omics) are to be exploited. This includes the opening towards non-pharmacological approaches and physical therapies that take advantage of our body’s ability to manage mechanical signals, an ability whose integrity has a direct impact on the control of wound healing, a hallmark of NCDs.

**References**

1. Hood L. A personal journey of discovery: developing technology and changing biology. Annu Rev Anal Chem. 2008;1:1–43, 2008. 10.1146/annurev.anchem.1.031207.113113.
2. Stolzenburg-Veeser L, Golubnitschaja O. Mini-encyclopaedia of the wound healing - opportunities for integrating multi-omic approaches into medical practice. J Proteomics. 2018;188:71–84. 10.1016/j.jprot.2017.07.017.
3. Konieczka et al. Flammer syndrome. EPMA J 2014;5(1):11. 10.1186/1878-5085-5-11.
4. GBD 2015 Risk Factors Collaborators. Global, regional, and national comparative risk assessment of 79 behavioural, environmental and occupational, and metabolic risks or clusters of risks, 1990–-2015: a systematic analysis for the Global Burden of Disease Study 2015. Lancet Lond Engl. 2016;388(10053):1659–1724. 10.1016/S0140-6736(16)31679-8.
5. Lerner A, Jeremias P, Matthias T. The World Incidence and Prevalence of Autoimmune Diseases is Increasing. Int j Celiac Dis. 2015;3:151–155
6. Sullivan SD, Alfonso-Cristancho R, Carlson J, Mallya U, Ringold S. Economic consequences of sequencing biologics in rheumatoid arthritis: a systematic review. J Med Econ. 2013;16(3):391–6. 10.3111/13696998.2013.763812.
7. Choi HH, Cho Y-S. Fecal microbiota transplantation: current applications, effectiveness, and future perspectives. Clin Endosc. 2016;49(3)3:257–265. 10.5946/ce.2015.117.
8. Cezar CA, Roche ET, Vandenburgh HH, Duda GN, Walsh CJ, Mooney DJ. Biologic-free mechanically induced muscle regeneration. Proc Natl Acad Sci U A. 2016;113(6):1534–9. 10.1073/pnas.1517517113.
9. Koopman FA et al. Vagus nerve stimulation inhibits cytokine production and attenuates disease severity in rheumatoid arthritis. Proc Natl Acad Sci U A. 2016;113(29):8284–9. 10.1073/pnas.1605635113.
10. Nardini C, Carrara S, Liu Y, Devescovi V, Lu Y, Zhou X. I-needle: detecting the biological mechanisms of acupuncture. Science. 2014;346(6216 Suppl)S21–S22.
11. Nardini C, Carrara S, Liu Y, Devescovi V, Lu Y, Dent JE. Systemic wound healing associated with local sub-cutaneous mechanical stimulation. Sci Rep. 2016;6(39043). 10.1038/srep39043.
12. Xiaoyuan Z, Devescovi V, Yuanhua L, Dent JE, Nardini C. Host-microbiome synergistic control on sphingolipid metabolism by mechanotransduction in model arthritis. Biomolecules. 2019;9(4):144.
13. Andersson U, Tracey KJ. Reflex principles of immunological homeostasis. Annu Rev Immunol. 2012;30:313–35. 10.1146/annurev-immunol-020711-075015.
14. Carabotti M, Scirocco A, Maselli MA, Severi C. The gut-brain axis: interactions between enteric microbiota, central and enteric nervous systems. Ann Gastroenterol. 2015;28(2):203–209.
15. Maturo MG, Soligo M, Gibson G, Manni L, Nardini C. The greater inflammatory pathway-high clinical potential by innovative predictive, preventive, and personalized medical approach. EPMA J. 2019;11(1):1–16. 10.1007/s13167-019-00195-w.

**A Blood biomarker panel recommended for personalized prediction, prognosis and prevention of rheumatoid arthritis**

Suchy D^1^, Fuchsova R*^2^, Topolcan O^2^, Kucera R^2^

^1^Department of Clinical Pharmacology, ^2^Department of Immunochemistry Diagnostics, University Hospital and Faculty of Medicine in Pilsen, Charles University, Czech Republic

*Corresponding author: Radka Fuchsova, MD, Department of Immunochemistry Diagnostics, University Hospital in Pilsen, Czech Republic; e.mail: fuchsovar@fnplzen.cz,

**Keywords:** rheumatoid arthritis, inflammation, synovial tissue, preventive predictive and personalized medicine (PPPM), early diagnosis, biomarker panel, prognostic factors, monitoring disease activity, predictive biomarkers, disease management

**Introduction**

Rheumatoid arthritis (RA) is the most common chronic autoimmune joint disease affecting synovial tissue in multiple joints. Early detection of RA and the availability of biological agents have markedly improved outcomes in patients [1]. At present, composite indices (mostly DAS28, Disease Activity Index) are used to monitor the activity of the disease. They include clinical parameters (tender and swollen joints), and two laboratory parameters of inflammation: C reactive protein (CRP) and erythrocyte sedimentation rate parameter (ESR). The disadvantage of these scores is the degree of subjectivity of some of the criteria. Moreover, a significant proportion of the patients with negative inflammatory tests still have active disease.

**Rheumatoid arthritis and PPPM**

RA is a typical example of a disease that requires new early predictive diagnostics measures. The principles of predictive, preventive and personalized medicine (PPPM) [2] and their appropriate application can be a good starting point for changing the general approach to RA management [3]. There is a clear need to find efficient diagnostic and prognostic biomarkers for this disease in order to prevent its rapid progression and subsequent functional decline [4]. PPPM enables the prediction of individual predisposition to the disease, can help with targeted preventive measures and dictates the design of personalized treatment algorithms tailored to the individual. Furthermore, PPPM contributes to the cost-effective management of the disease [5]. PPPM principles are, at present, slowly entering clinical practice. The effort to implement this new approach in the field is most visible in the search for new biomarkers: starting with genetic biomarkers, followed by single blood biomarkers and finalized by the multi-omics approach [6].

**The role of biomarkers in RA diagnostics and treatment**

The use of biomarkers in RA diagnostics began many years ago in other fields of medicine; namely oncology [7]. PPPM, as well as biomarkers, are making important contributions to other fields of medicine [8]. There are several key stages of RA, the proper management of which may influence prognosis, progression of disease and management (Table 1).

**Table 1** Biomarkers in the PPPM approach

**Table:** 

Early diagnosis	Starting treatment in the early stages prevents osteoarticular destruction
Prognostic factors	Identifying patients at high risk of an aggressive form of RA
Monitoring disease activity	Evaluating treatment efficacy
Predictive factors for therapy	Biomarkers predictive of the response to treatment

Predictive diagnostics and immediate preventive therapy are crucial for the halting of joint deterioration, functional disability and unfavourable disease outcome. The 2010 RA classification criteria from the American College of Rheumatology/European League Against Rheumatism (ACR/EULAR) consists of symptoms and laboratory findings within 4 domains (total score 10 points). 40% of this total possible score relies on laboratory tests. Up to 3 points are generated by the presence of rheumatoid factor (RF) and/or anti-citrullinated peptide antibodies (ACPA). Equal weight is given to RA and ACPA. Among ACPAs, anti-CCP, the anti-cyclic citrullinated peptide assay, has a superior diagnostic and prognostic value. The sensitivity of the anti-CCP assay in RA is 60–80%, with a very high specificity of 95–99%. The assay has a high predictive value and the autoantibody can be found early, even in the preclinical phase of RA.

**Anti-mutated citrullinated vimentin (anti-MCV)**

Vimentin is a protein that can be citrullinated, a reaction mediated by peptidyl arginine deiminase. A meta-analysis from 2010 that included 14 studies in which anti MCV and anti-CCP were tested, concluded that there is no substantial difference between the two tests. Thus, determination of anti-MCV seems to be useful as a line 2 test in patients suspected of having RA but is negative for both RF and anti-CCP [9].

**14-3-3η protein**

The 14-3-3 family of proteins is a family of chaperone proteins. The combination of 14-3-3η protein, RF and anti-CCP will help increase the sensitivity and specificity for patients with early active disease, help stratify RA patients and enable us to individualize treatments for patients in early stages. It may predict both clinical and radiographic progression, as well as treatment response [10]. Lower 14-3-3η levels were found in patients who achieved remission during biologic therapy with anti-IL 6 monoclonal antibody (tocilizumab). Post-treatment 14-3-3η expression is significantly different between stages.

**The multi-biomarker disease activity index (MBDA index)**

During the development of the MBDA score, 96 candidate biomarkers were reduced to 12 (CRP, leptin, resistin, serum amyloid A, IL-6, TNF-RI, VEGF-A, MMP-1, YKL-40, MMP-3, EGF, VCAM-1), that run as a single test and result in a score of 0–100. Depending on the values of this score, the disease activity can be classified into mild (1–28), moderate (29–43) or severe (>44). The MBDA score is a very good determinant of subclinical disease activity and progressive disease with structural progression. In addition, the MBDA score may help individualize treatment by determining which patients should receive more aggressive/expensive therapy and which patients may do well with conventional therapy [11].

**Calprotectin**

Calprotectin (S100 protein) might be a valuable marker of RA disease activity. Calprotectin differs from other laboratory markers in its local production from activated synovial cells and release from inflamed synovium. As a consequence, calprotectin directly reflects the amount of activated macrophages and the extent of inflammation. Calprotectin might be superior to CRP for the detection of patients in clinical remission with subclinical disease activity. Several authors have reported increased calprotectin serum levels in RA patients, its association with disease activity, its dynamic decrease after initiation of effective treatment and its role in predicting response to treatment [12].

**Conclusions and PPPM-related expert recommendations**

The goal of current and future biomarker use in rheumatic diseases is to enable early detection, effective monitoring and the individualization of treatment.

- Anti MCV, 14-3-3η protein, MBDA score and calprotectin were identified as important parameters in predictive diagnostics, as well as in the assessment of patients’ response to treatment
- The MBDA index is a panel of biomarkers that correlates with disease activity both clinically and radiographically.
- Serum biomarkers had stronger associations with joint damage than did clinical assessment.
- A multi-biomarker score for structural damage has the potential to aid the personalized assessment of joint damage risk and identify patients who most need joint sparing therapy, as well as track disease changes in response to therapy.
- New biomarkers, alongside suitably applied PPPM rules, help in the cost-effective management of diseases, including RA.

**References**

1. Nakken B, Papp G, Bosnes V, Zeher M, Nagy G, Szodoray P. Biomarkers for rheumatoid arthritis: From molecular processes to diagnostic applications-current concepts and future perspectives. Immunol Lett. 2017; 10.1016/j.imlet.2017.05.010
2. Golubnitschaja O, Costigliola V, EPMA. General report & recommendations in predictive, preventive and personalised medicine 2012: white paper of the European Association for Predictive, Preventive and Personalised Medicine. EPMA J. 2012; 10.1186/1878-5085-3-14.
3. Golubnitschaja O, Baban B, Boniolo G, Wang W, Bubnov R, Kapalla M, et al. Medicine in the early twenty-first century: paradigm and anticipation-EPMA position paper 2016. EPMA J. 2016;7:23.
4. Gavrilă BI, Ciofu C, Stoica V. Biomarkers in rheumatoid arthritis, what is new? J Med Life. 2016;9:144–148.
5. Lemke HU, Golubnitschaja O. Towards personal health care with model-guided medicine: long-term PPPM-related strategies and realisation opportunities within ‘Horizon 2020’. EPMA J. 2014; 10.1186/1878-5085-5-8.
6. Lu M, Zhan X. The crucial role of multiomic approach in cancer research and clinically relevant outcomes. EPMA J. 2018; 10.1007/s13167-018-0128-8.
7. Hu R, Wang X, Zhan X. Multi-Parameter systematics trategies for predictive, preventive and personalized medicine in cancer. EPMA J. 2013; 10.1186/1878-5085-4-2.
8. Molacek J, Treska V, Zeithaml J, Hollan I, Topolcan O, Pecen L, et al. Blood biomarker panel recommended for personalized prediction, prognosis, and prevention of complications associated with abdominal aortic aneurysm. EPMA J. 2019; 10.1007/s13167-019-00173-2.
9. Syversen SW, Goll GL, van der Heijde D, Landewe R, Lie BA, Odegard S, et al. Prediction of radiographic progression in rheumatoid arthritis and the role of antibodies against mutated citrullinated vimentin: results from a 10-year prospective study. Ann Rheum Dis. 2010; 10.1136/ard.2009.11392.
10. Maksymowych WP, Naides SJ, Bykerk V, Siminovitch KA, van Schaardenburg D, Boers M, et al. Serum 14-3-3η is a novel marker that complements current serological measurements to enhance detection of patients with rheumatoid arthritis. J Rheumatol. 2014; 10.3899/jrheum.131446.
11. Centola M, Cavet G, Shen Y, Ramanujan S, Knowlton N, Swan KA, et al. Development of a multi-biomarker disease activity test for rheumatoid arthritis. PLoSOne. 2013; 10.1371/journal.pone.0060635.
12. Hurnakova J, Hulejova H, Zavada J, Hanova P, Komarc M, Mann H, et al. Relationship between serum calprotectin (S100A8/9) and clinical, laboratory and ultrasound parameters of disease activity in rheumatoid arthritis: A large cohort study. PLoS One. 2017; 10.1371/journal.pone.0183420.

**Soluble adhesion molecules in prognosis of the rheumatoid arthritis therapy**

Sarithala VJ*, Yagoda AV, Koroy PV

*Corresponding author

Department of hospital therapy, Stavropol State Medical University, Stavropol, Russia

The aim of this study was to assess the relationship between blood levels of adhesion molecules and results of biological therapy in rheumatoid arthritis (RA); 35 patients with RA (29 women, 6 men) aged from 40 to 66 years, who were undergoing biological therapy (tocilizumab – 20 patients, etanercept – 15 patients) in combination with methotrexate were examined.

Positive response on treatment was seen in 74.3% of patients, and in 25.7% of cases treatment was ineffective. The control group consisted of 70 healthy individuals. Blood concentration of intercellular adhesion molecule-1 (ICAM-1), vascular cell adhesion molecule-1 (VCAM-1), platelet-endothelial cell adhesion molecule-1 (PECAM-1) Е-, Р- and L-selectins was carried out using ELISA before and after 12 weeks of treatment. The diagnostic value of parameters were defined by their accuracy (Ac). In RA, an increase in levels of all molecules of immunoglobulin superfamily, P-selectin was observed. In patients with positive results for therapy, normalization of ICAM-1, P-selectin and decrease of VCAM-1, PECAM-1 were observed. In patients with no effect from treatment, only concentration of ICAM-1 was reduced. Concentrations of ICAM-1 above 886 ng/ml, PECAM-1 above 101 ng/ml, Е-selectin below 40 ng/ml, Р-selectin below 176 ng/ml were associated with an increased chance of responding to biological therapy. These values of ICAM-1 (Ac 88.6%), Р-selectin (Ac 82.9%) and PECAM-1 (Ac 71.4%) were characterized with high accuracy in delineation of treatment results. Therefore, we conclude that increased blood concentration of adhesion molecules in RA was decreased in dynamics of biological therapy. Initial adhesins levels can be used as predictors of treatment outcomes in RA.

**DEFa1 and DEFb1 in the role of biomarkers of predisposition to the activation of infectious process in inflammatory diseases of the pelvic organs in women**

Baturin VA^1,2^, Boshyan RO*^1,2^

^1^Stavropol State Medical University, Russia

^2^Center for Clinical Pharmacology and Pharmacotherapy, Stavropol, Russia

*Corresponsing author: Dr. R.O. Boshyan, Stavropol State Medical University, Russia

DEFa1 and DEFb1 are one of the first lines of defense against the entry of bacteria, fungi and viruses. DEFa1 is involved in oxygen-independent destruction of phagocytosed microorganisms, in the systemic immune response. DEFb1 is released on the mucosal surface and provides anti-infective protection. The lack of defensins synthesis leads to the development of infectious and inflammatory complications. We studied the levels of DEFa1 and DEFb1 in the blood and studied the species composition of the microflora of the urogenital tract in 153 women of reproductive age in the city of Stavropol of the Russian Federation. The subjects were divided into groups: with minimal clinical symptoms of the infectious process, with moderate, with severe. The concentration of DEFa1 was in the group with minimal symptoms of 21.1 ± 6.5 ng / ml, and DEFb1 was 75.6 ± 14.4 ng / ml (*p* < 0.05). The level of DEFa1 was 46.5 ± 12.5 ng / ml, and DEFb1 was 27.3 ± 7.8 ng / ml (*p* < 0.05) in the group of women with severe symptoms. Concentration of DEFb1 had more high values 90.2 ± 35.7 ng / ml in women at the end of the second menstrual cycle, and a very high correlation was found between the number of leukocytes in flora smears and the expression of DEFb1(r = 0.97, *p* < 0.05). The authors recommend taking into account the concentration of DEFa1 and DEFb1 in the blood of women as a predisposition to the activation of the infection process during inflammatory diseases of the pelvic organs.

## Application of microbiome, immune-, pre- and probiotics – 3PM concepts

**Microbiome in leanness: potential impacts for stratifying suboptimal health conditions and preventive treatments tailored to the person**

Bubnov R^1,2^, Radchenko D^3^, Golubnitschaja O*^4^

^1^Zabolotny Institute of Microbiology and Virology, National Academy of Sciences of Ukraine

^2^Clinical hospital‘Pheophania’of State Affairs Department, Ukraine

^3^Center of Molecular Biotechnology, Friedrich-Wilhelms-University of Bonn, Germany

^4^Predictive, Preventive and Personalised (3P) Medicine, Department of Radiation Oncology, University Hospital Bonn, Rheinische Friedrich-Wilhelms-Universität Bonn, Germany

*Corresponding author: Prof. Dr. Olga Golubnitschaja, Predictive, Preventive and Personalised (3P) Medicine, Department of Radiation Oncology, University Hospital Bonn, Rheinische Friedrich-Wilhelms-Universität Bonn, Venusberg-Campus 1,53127 Bonn, Germany; e.mail: Olga.Golubnitschaja@ukbonn.de

**Keywords:** predictive preventive personalised medicine; patient stratification, individualised patient profile; microbiome; metabolic health; leanness; low BMI; hypoxia; probiotics; prebiotics; gut-brain axis; microbial; diversity; butyrate; hypoxia; mesenteric ischemia

**Introduction**

Microbiome research is amongst the most innovative fields in biomedical sciences being referred to as a second genome [1–3]. The epidemic of obesity and associated comorbidities, which apart from being a healthcare issue of modern society, is a tremendous economic burden, although currently less explored, the microbiota specific for lean people demonstrates its clear particularities and deviations compared to people with both normal and increased BMI [2]. To date, the obesity specific microbiome has been prioritised for extensive studies demonstrating human microbiota as affecting the thermogenesis, energy balance and even food consuming behaviour, amongst others.

Specifically, in the context of predictive, preventive and personalised medicine, the EPMA expert group has strongly contributed to the recent knowledge accumulated towards leanness specific microbiota [3,4]. The most comprehensive updates to the topic are provided in the EPMA/Springer book “Flammer Syndrome - From Phenotype to Associated Pathologies, Prediction, Prevention and Personalisation” [5].

The current study is designed to correlate the leanness and particularly the Flammer Syndrome (FS) phenotype (anorexia nervosa as an extreme case of FS) with particularities of their microbiome profiles.

**FS phenotype** associated symptoms and complications are considered by the patient stratification, namely:

- primary vascular dysregulation;
- altered regulation of senses (thirst, pain, smell, etc.) and sensitivity towards medications altered circadian rhythms and sleep patterns;
- psychological and psychiatric aspects (e.g. tendency to meticulous personality and depression)
- increased incidence of dry mouth and sicca syndrome;
- more prevalent impaired wound healing as well as a predisposition to young stroke and aggressive metastatic cancer subtypes in the family, amongst others [6–10].

The concept of **the brain-gut axis** suggests that modulating the gut microbiome might be a strategy to prevent stress-related disorders, such as depression, anxiety and CNS disorders [11,12].

Sleep loss or disturbance, common for people having FS, may act as a stressor changing the microbial composition, which leads to metabolic dysfunction. Altered circadian rhythm increases the permeability of the intestinal barrier increasing risks of inflammation [11]. Bifidobacteria amongst others are often underrepresented in depression [13].

The microbial composition of lean people is characterised by high diversity and increased presence of bacteria responsible for health beneficial short-chain fatty acids (e.g butyrate), which is linked to a reduced risk of cardiovascular diseases [14]. Prevotella has been outlined to exist in increased amounts in obese compared to lean people due to its ability to enhance the extraction of energy from starch and oligosaccharides [15]. The “lean enterotype”, abundant mostly by phyla Bacteroidetes and Firmicutes [16] although considered as ‘healthier’ requires careful reconsidering and validation of a patient-specific microbiome profiling for targeted treatments and prevention.

Multiple approaches are being developed to prevent and treat metabolic disorders by **modulating gut microbiome**, these include the use of pre- and probiotics, altering the diet, and fecal microbiota transplantation (FMT) [3,4]. Studies confirm that a **modified diet** positively influences the gut of patients with metabolic syndrome, which lean people can possess [17].

Bifidobacteria and Lactobacteria are the most studied bacteria for the role of **probiotics** that demonstrate efficacy to reduce obesity, dyslipidemia, and improve metabolic health [3,4].

Akkermansia muciniphila is a rather new application as a microbiome marker in patients with non-obese metabolic disorders and potentially most relevant probiotic strain for Flammer phenotype, and hypoxia-associated conditions. A. muciniphila is Gram-negative, strictly anaerobic, able to use mucin as its sole source of carbon and nitrogen, enhancing gut barrier function [18]. A. muciniphila abundance negatively correlates with BMI in adults [19].

Using novel substances demonstrating **prebiotic** qualities open new perspectives for combined application with probiotics benefiting the host health [20]. A very recent study has presented that nanoceria together with L.casei ІМV В-7280 demonstrates a great clinical potential to treat hypercholesterolemia in affected individuals [21].

**Hypoxia** results in an altered diversity of gut microbial communities, with a noticeable decrease of Lactobacilli. Mesenteric ischemia (MI) can be a relevant marker to stratify patients with metabolic disorders, in particular, in underweight being a source of hypoxic signalling [22].

However, Lactobacillus supplementation could recover the function of deficient bone marrow mesenchymal stem cells in the hypoxic rat model [23]. Modulating gut microbiome via administration of Bifidobacteria animalis probiotic strains can alleviate MI, as detected by Doppler ultrasound [24]; short-term probiotic therapy is effective if prescribed individualised.

A severe and common metabolic disorder such as gout leading to kidney failure may develop in lean individuals, research suggests that individualised probiotic treatments decrease inflammation in the body and improve metabolism [25]. Nevertheless, it remains challenging to establish a causal link (cause-effect relation) between microbial composition and a certain phenotype or disease.

**Conclusions and PPPM related expert recommendations**

Microbiology has been often considered as retrospective. However, microbiome research together with the novel, computational methods have great potential to lead the way to predicted and personalised medicine of the future.

Considering metabolic and microbiome as targets in lean individuals is an important task and requires a focused approach, including individualised prediction and treatments tailored to the person.

Individualised patient profiles might be used to optimise the pre- and probiotic composition for treatment modalities; for this the significance of stratification and pre-treatment questionnaires should be emphasised. It is crucial to pick the most appropriate strain and consider utilising a synergistic effect between pre- and probiotics. Follow-up translational measures to bring new knowledge from the lab to the patient are strongly recommended. If validated in a large-scale clinical study, this approach might be instrumental for primary and secondary prevention in affected underweight individuals and patients with metabolic disorders diagnosed with Flammer syndrome.

**References**

1. Integrative HMP (iHMP) Research Network Consortium. The Integrative Human Microbiome Project: dynamic analysis of microbiome-host omics profiles during periods of human health and disease. Cell Host Microbe. 2014;16(3):276–289. 10.1016/j.chom.2014.08.0142.
2. Turnbaugh PJ, et al. A core gut microbiome in obese and lean twins. Nature. 2009;457(7228):480–4. 10.1038/nature07540.
3. Bubnov RV, Spivak MY, Lazarenko LM, Bomba A, Boyko NV. Probiotics and immunity: provisional role for personalized diets and disease prevention. EPMA J. 2015;6:14.
4. Bubnov RV, Babenko LP, Lazarenko LM, Mokrozub VV, Spivak MY. Specific properties of probiotic strains: relevance and benefits for the host. EPMA J. 2018 Jun; 9(2): 205–223.
5. Bubnov R, Golubnitschaja O. Flammer syndrome, disordered eating and microbiome: interrelations, complexity of risks and individual outcomes. IIn: Golubnitschaja O (Ed.) Flammer syndrome - from phenotype to associated pathologies, prediction, prevention and personalization. Springer, Cham. 2019; ISBN 978-3-030-13552-2.
6. Avishai E, Yeghiazaryan K, Golubnitschaja O. Impaired wound healing: facts and hypotheses for multi-professional considerations in predictive, preventive and personalised medicine. EPMA J. 2017;8(1), 23–33. 10.1007/s13167-017-0081-y.
7. Kunin A, Polivka J Jr., Moiseeva N, Golubnitschaja O. “Dry Mouth” and “Flammer” syndromes - neglected risks in adolescents and new concepts by predictive, preventive and personalised approach. EPMA J. 2018;9(3):307–317. 10.1007/s13167-018-0145-7
8. Bubnov R, Polivka J Jr., Zubor P, Konieczka K, Golubnitschaja O. "Pre-metastatic niches" in breast cancer: are they created by or prior to the tumour onset? "Flammer syndrome" relevance to address the question. EPMA J. 2017;8(2):141–57. 10.1007/s13167-017-0092-8.
9. Goncharenko V, Bubnov R, Polivka J Jr., Zubor P, Biringer K, Bielik T, Kuhn W, Golubnitschaja O. Vaginal dryness: individualised patient profiles, risks and mitigating measures. EPMA J.2019;10(1):73–79. 10.1007/s13167-019-00164-3.
10. Polivka J Jr., Polivka J, Pesta M, Rohan V, Celedova L, Mahajani S, Topolcan O, Golubnitschaja O. Risks associated with the stroke predisposition at young age: facts and hypotheses in light of individualized predictive and preventive approach. EPMA J. 2019;10(1):81–99. doi: 10.1007/s13167-019-00162-5.
11. Reynolds AC, et al. The shift work and health research agenda: considering changes in gut microbiota as a pathway linking shift work, sleep loss and circadian misalignment, and metabolic disease. Sleep Med Rev. 2017;34:3–9.
12. Foster JA, Rinaman L, Cryan JF. Stress & the gut-brain axis: regulation by the microbiome. Neurobiol Stress. 2017;7:124–136. 10.1016/j.ynstr.2017.03.001
13. Kuo PH, Chung YE. Moody microbiome: Challenges and chances. J Formos Med Assoc. 2019;118 Suppl 1:S42-S54. 10.1016/j.jfma.2018.09.004.
14. Gomes AC, Hoffmann C, Mota JF. Gut microbiota is associated with adiposity markers and probiotics may impact specific genera. Eur J Nutr. 2019. 10.1007/s00394-019-02034-0.
15. Hu HJ, Park SG, Jang HB, Choi MK, Park KH, Kang JH et al. Obesity alters the microbial community profile in Korean adolescents. PLoS One. 2015;10:e0134333. 10.1371/journal.pone.0134333.
16. Turnbaugh PJ, Hamady M, Yatsunenko T, Cantarel BL, Duncan A, Ley RE, Sogin ML, Jones WJ, Roe BA, Affourtit JP, et al. Nature. 2009;457:480–484.
17. Kim MS, Hwang SS, Park EJ, Bae JW. Strict vegetarian diet improves the risk factors associated with metabolic diseases by modulating gut microbiota and reducing intestinal inflammation. Environ Microbiol Rep 2013;5:765–775. 10.1111/1758-2229.12079
18. Qixiao Zhai, et al. A next generation probiotic, Akkermansia muciniphila. Crit Rev Food Sci Nutr. 2019;59(19):3227–3236. 10.1080/10408398.2018.1517725.
19. Zhang T, Li Q, Cheng L, Buch H, Zhang F. Akkermansia muciniphila is a promising probiotic. Microb Biotechnol. 2019;12(6):1109–1125. 10.1111/1751-7915.13410
20. Gibson GR, et al. Expert consensus document: the International Scientific Association for Probiotics and Prebiotics (ISAPP) consensus statement on the definition and scope of prebiotics. Nat Rev Gastroenterol Hepatol. 2017;14(8):491–502. 10.1038/nrgastro.2017.75.
21. Bubnov R, Babenko L, Lazarenko L, et al. Can tailored nanoceria act as a prebiotic? Report on improved lipid profile and gut microbiota in obese mice. EPMA J. 2019; 10.1007/s13167-019-00190-1
22. Friedman ES, et al. Microbes vs. chemistry in the origin of the anaerobic gut lumen. Proc Natl Acad Sci U S A. 2018;115(16):4170–4175. 10.1073/pnas.1718635115
23. Xing J, et al. Hypoxia induces senescence of bone marrow mesenchymal stem cells via altered gut microbiota. Nat Commun. 2018;9:2020.
24. Bubnov R, Spivak M. Bifidobacteria animalis VKL and VKB probiotic strains are effective for alleviating mesenteric ischemia in patients with metabolic syndrome Turk J Gastroenterol 2019;30(Suppl. 3):S313–S314. 10.5152/tjg.2019.050919
25. American Physiological Society. Short-term probiotics regimen may help treat gout, kidney disease. 2019; https://www.the-aps.org/detail/news/2019/10/04/short-term-probiotics-regimen-may-help-treat-gout-kidney-disease?SSO=Y (Accessed 26.11.2019).

**Individualized probiotic therapy of metabolic syndrome: preliminary clinical results**

Bubnov R*^1,2^, Spivak M^1^

^1^Zabolotny Institute of Microbiology and Virology, National Academy of Sciences of Ukraine, Zabolotny Str., 154, Kyiv, 03143 Ukraine

^2^Clinical Hospital “Pheophania” of State Affairs Department, Zabolotny Str., 21, Kyiv, 03143 Ukraine

*Corresponding author: Dr. Rostyslav Bubnov, Zabolotny Institute of Microbiology and Virology, National Academy of Sciences of Ukraine; e.mail: dr.rbubnov@gmail.com

**Introduction**

The definition of metabolic syndrome (MetS) requires detection of central obesity +2/4 factors (hyperglycemia, dyslipidemia, cardiovascular disease, hypertension). Ultrasound (US) can accurately detect visceral obesity and a number of markers for stratification of patients with MetS.

**The Aim:** was to study the efficacy of individualized probiotic therapy of MetS according to the host’s phenotype using multiparameter ultrasound markers.

**Material and methods**

We included 116 overweight subjects (age 23–74 years; 67 females), BMI > 30, waist circumference (WC) > 110, or constituents of MetS and normal / low BMI, who underwent general clinical, lab tests; radiology tests for stratification according to the developed MetS biomarker panel, which included:

- Anthropometric (weight, BMI, WC);
- Metabolic markers (NAFLD liver size and stiffness) portal hypertension;
- Pro-inflammatory (visceral fat (VF), microsplenia, intestinal wall thickness);
- Vascular (mesenteric, renal blood flow Doppler), atherosclerosis plaques;
- Hypoxia markers;
- Microbiota markers;
- ‘Mechanistical’ – posture parameters, muscle signaling, trigger points, nerves.

After careful examination we selected 10 patients for probiotic therapy with patterns of MetS as follows: DMT2; liver fibrosis, NAFLD; hyperuricemia, gout; atherosclerosis; hypertension (early age); and 3 patients with normal / low BMI with detected increased VF. Probiotic strains were selectively given according to [1,2]: at a dose 10^8^ CFU daily for 10 days: *L. acidophilus* ІМV В-7279, *L. casei* ІМV В-7280, *L. delbrueckii* subsp. *bulgaricus* ІМV В-7281, *L. rhamnosus* LB-3 VK6, *L. delbrueckii* LE VK8, *L. plantarum* LM VK7), *Bifidobacterium* genus (*B. animalis* VKL, *B. animalis* VKB.

**Results**

Weight, BMI, WC and VF decreased after probiotic administration. All studied parameters improved in all cases—US markers of visceral obesity, gut motility, colonic wall thickness, liver size and stiffness, mesenteric lymph nodes, spleen size and Doppler markers of mesenteric blood flow.

**Conclusion**

Short-term probiotic therapy is effective for various metabolic disorders in obese and lean individuals if prescribed individualized (see Fig. 1).

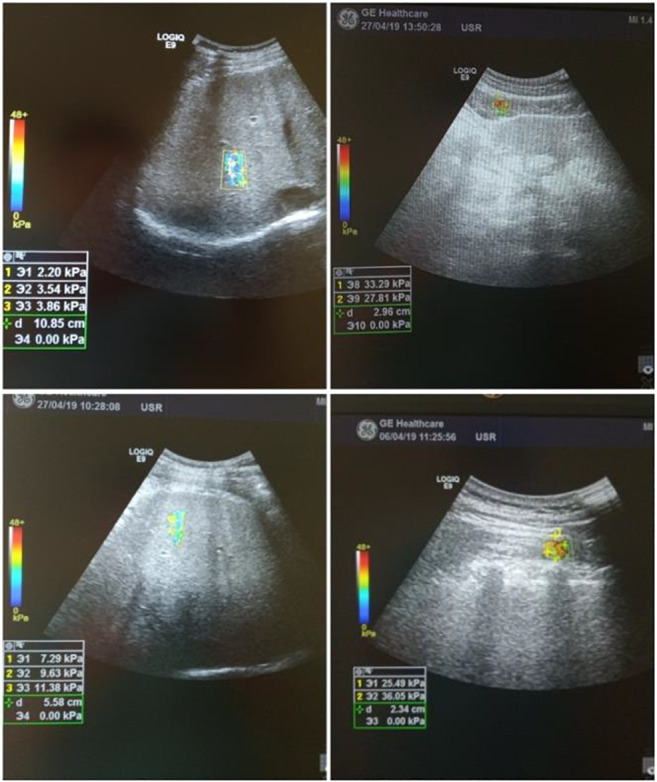



**Fig. 1** Positive progress of liver (left) and visceral fat (right) structure and stiffness (upper –after therapy)

**References**

1. Bubnov RV, Babenko LP, Lazarenko LM, Mokrozub VV, Spivak MY. Specific properties of probiotic strains: relevance and benefits for the host. EPMA J. 2018 Jun; 9(2): 205–223.
2. Bubnov RV, Babenko LP, Lazarenko LM, Mokrozub VV, Demchenko OA, Nechypurenko OV, Spivak MY. Comparative study of probiotic effects of Lactobacillus and Bifidobacteria strains on holesterol levels, liver morphology and the gut microbiota ino bese mice. EPMA J. 2017;8(4):357–376.

**Personalized pharmabiotics and individual nutrition for nosology specific correction of microbiota and local immune system biomarkers**

Meleshko T^1^, Rukavchuk R^1^, Drobnych V^1^, Boyko N*^1^

^1^RDE Centre of Molecular Microbiology and Mucosal Immunology, Uzhhorod National University, Uzhhorod, Ukraine

^2^Ediens LTD, Uzhhorod, Ukraine

*Corresponsing author: Prof. Dr. N. Boyko, ^1^RDE Centre of Molecular Microbiology and Mucosal Immunology, Uzhhorod National University, Uzhhorod, Ukraine; e.mail: nadiya.boyko@gmail.com

**Keywords**: personalized pharmabiotics, individual biomarkers, information system (IS), algorithm, individual nutritional needs, predictive preventive personalized medicine (3PM)

**Individual human microbiome as a potential tool in precise diagnostic and nosology-specific prevention and treatment of noncommunicable diseases**

The human microbiome determines our personal health, thus prognostic correction of detected composition to expected functional activity by pharmabiotics and/or individual nutrition is a promising approach for the prevention and treatment of noncommunicable diseases (NCD) triggered by low-grade inflammation [1]. These facts along with the assessment of individual behavior and lifestyle to determine personalized nutrition or individual biotics appointment mean that the use of biotics (pre-, pro-, syn-, immune, and pharmabiotics) should be individually designed to specific nosology.

**Addressed nosologies of dominant noncommunicable diseases and used methodology**

The most typical and reported NCD, associated with “unhealthy” microbiome changes were addressed in the current study. The first targeted nosology was caries in children and periodontitis in the elderly population connected with microbiome changes. Both diseases were supplemented with enterocolitis. The second nosology group was formed by women and men with so-called urogenital inflammation symptoms, and the third group consisted of “metabolic disorders” patients, ranging from overweight persons (obese), patients with detected diabetes type 2 (DT2), and individuals with various stages of cardiovascular diseases (CVD). The number of patients in the first group was 40 (20 for each oral nosology), 40 for urogenital chronic inflammation processes (20 for both genders), and 35 for each group of metabolic diseases. The exclusion and inclusion criteria for these selected nosologies were previously reported [2, 3].

Microbiome composition of secrets from oral cavity (saliva), vaginal and genital washes, and gut content of patients were detected by qRT-PCR (ParadontoScreen, Femoflor®-16, Androflor®), and 16S rRNA sequencing techniques for the gut specimens. In addition, the levels of pro- and anti-inflammatory cytokines: TNF-α, IFN-γ, IL- 6, 10, 12, 17, IL-1β, SIgA, IgA, were determined by ELISA, and biochemical biomarkers (for obese, DT2 and CVD) relevant to early inflammation were routinely detected. Individual profiles of patients were developed, and, in order to recommend the individual treatment either with pharmabiotics or nutrition for these different nosologies, the information system (IS) has been built.

**Information system was built and applied for the individual pharmabiotics creation and testing**

The information system for personalized pharmabiotics creation and prescriptions aimed to regulate the saliva/gut microbiota ratio, biodiversity, and functionality had been developed.

The proposed IS operates as an algorithm for the selection of individually required contents from a large amount of data (databases, DBs), in particular: i) patients’ anamnesis, medical examination results, ii) biomarkers; iii) defined microbiome representatives/ratio for the majority of NCDs, iv) biologically active molecules (BAM) of edible plants, v) clinically proven data available for the registered influence of BAM, fibers, other food components, fermented products starters, and other connected microbiota on human microbiome representatives diagnostic ratio, vi) food composition DBs, etc. Further development of the IS tool is oriented on precise diagnostic at earlier stages of diseases and improvement of individual recommendations by machine learning techniques.

Using this system and the DBs, we designed and clinically probed the efficacy of pharmabiotics of new generation branded as ProPhyLactOR™ for the oral “caries” and “periodontitis” types microbiome correction, with two separate treatment forms, and FlexiLactUR™ with individually adjusted pre- and probiotic components aimed to correct the microbiome composition of the urogenital tract. The initial composition contains synergistically acting commensal microbiota representatives and prebiotic ingredients from edible plants [4]. The content of pharmabiotics could be slightly modified and adjusted to the patients’ individual microbiome-immune profile and to the detected nosology. This developed and successfully clinically tested algorithm for personalized nutrition (Fig. 1) for prevention and treatment of 1) childhood and adult obesity; 2) DT2, 3) CVD, enabled the designing of the novel ethnic fermented traditional foods and beverages branded as Ediens™.

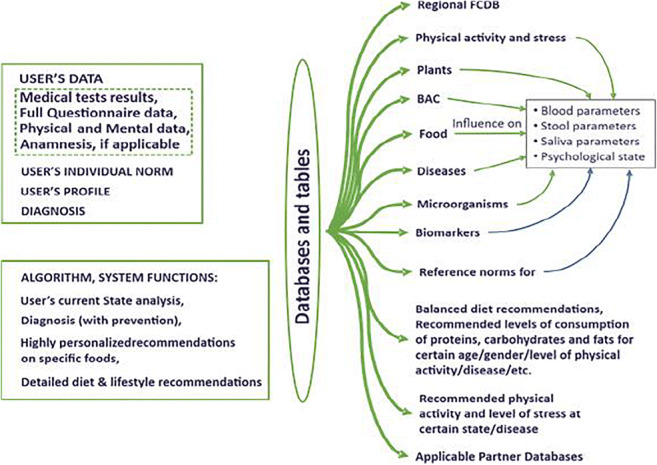



**Fig. 1** Algorithm for personalized nutrition calculation

The limited controlled clinical diet study, where we apply a two week individual nutrition plan by using food ingredients, rich in BAM (local plants) in combination with prepared fermented products with unique starters, showed a significant decrease in insulin resistance.

**Limited control diet study for the approval of accuracy of the algorithm applied for prognostic microbiome correction by individual nutrition needs calculation**

Trends to the normalization of glucose level and a statistically important decrease of very low-density lipids (VLDL), triglycerides, glycosylated hemoglobin (HbA1c), uric acid and creatinine had been detected, while the level of high-density lipids (HDL) increased in a non-statistically significant manner. Similarly to biochemical indices, the physical parameters in the experimental group of patients had been changed. The implementation of a control diet led to the normalization of the majority of commensal gut microbiome representatives, including beneficial anaerobes. The increasing number of *Lactobacillus casei*, but not of *L. acidophilus*, was accompanied by the reduced levels of HbA1c. The reduction of cholesterol and triglycerides in the blood of patients had been correlated with the decreased amount of *Enterococcus faecalis*, but not of *E. faecium*, in the gut microbiota content. The data of the clinical trial (63 parameters of each patient) was used for the development of a new methodology for the detection of personalized treatment process biomarkers. These biomarkers are essential for the development of new machine learning models, which could predict the success of a patient’s personalized treatment.

**3PM related conclusion and outlook**

The CVD are subject to prevention by correction of the individual profile including each person’s unique microbiome dependent on individual lifestyle, nutrition and behaviour. To recommend prognostic treatment for the patients with a diagnosed stage of “metabolic disorder”, IT tools (IS) need to be applied in order to calculate the complex individual biomarkers in the context of individual variations.

The algorithm under testing is a potential tool to estimate (in addition to the detected genetic) lifestyle-dependent, epigenetic, environmental and nutritional factors in an individual to provide the level of recommendations and adequate prognostication that is crucial to implement 3PM [5].

To prove and suggest a provisional role of individual nutrition or pharmabiotics treatment in 3PM, the cohort studies with precise individual measurements of the biomarkers, prioritized with correlation analysis of the initial data, analysis of the main components, cluster analysis and a well-known machine learning procedure “feature selection”, need to be performed. The strong demands for the improving of the design of cohort studies are highly recommended by 3PM experts [6].

**References**

1. Dietert R, Dietert J. The microbiome and sustainable healthcare. Healthcare. 2015:3:1:100–129. 10.3390/healthcare3010100
2. Chendey T, Rishko M, Boyko N, Kroon P. Six weeks ingestion of polyphenol-rich *Urtica dioica* and *Sideritis scardica* does not influence endothelial function, blood pressure or lipid profile in patients with coronary artery disease or at high cardiovascular risk: a randomised controlled trial. Ukrainian Med J. 2014;99:132–133.
3. Petrov V, Boyko N. Early diagnostic markers of obesity, diabetes and metabolic syndrome. Ukraine patent 90788. 2014.
4. Melnyk V, Dyachuk E, Bati V, Levchuk O, Boyko N. Composite biological product for the treatment of inflammation of periodontal tissues and correction of associated gastroduodenal disorders of the intestine in children. Ukraine patent 93301. 2014.
5. Golubnitschaja O, Watson ID, Topic E, Sandberg S, Ferrari M, Costigliola V. Position paper of the EPMA and EFLM: a global vision of the consolidated promotion of an integrative medical approach to advance health care. EPMA J. 2013;4:12. 10.1186/1878-5085-4-12
6. Gehr S, Kaiser T, Kreutz R, Ludwig WD, Paul F. Suggestions for improving the design of clinical trials in multiple sclerosis-results of a systematic analysis of completed phase III trials. EPMA J. 2019;10:425–436. 10.1007/s13167-019-00192-z

**Creation of lactobacilli based pharmabiotics for individual prevention and treatment of infectious-inflammatory diseases of the human urogenital system**

Meleshko T^1^, Babenko L^2^, Lazarenko L^2^, Boyko N*^1^

^1^RDE Centre of Molecular Microbiology and Mucosal Immunology, Uzhhorod National University, Uzhhorod, Ukraine

^2^Department of Problems of Interferon and Immunomodulators, Zabolotny Institute of Microbiology and Virology of NAS of Ukraine, Kyiv, Ukraine

*Corresponsing author: Prof. Dr. N. Boyko, ^1^RDE Centre of Molecular Microbiology and Mucosal Immunology, Uzhhorod National University, Uzhhorod, Ukraine; e.mail: nadiya.boyko@gmail.com

**Keywords**: personalized pharmabiotics, microbiome correction, lactobacilli, nosology-specific treatment, strain-dependent efficacy, predictive preventive personalized medicine (3PM)

**The principles for individual immunobiotics developments and testing of their efficacy on the urogenital tract infectious-inflammation diseases**

Personalized use of immunobiotics is a promising direction in the prevention of various infectious and inflammatory diseases associated with microbiome changes of the genitourinary system in humans.

Recently, the strain-dependent properties (adhesive ability, resistance to antibiotics, and gut biological fluids) of probiotic strains (lactobacilli and bifidobacteria) were reported, and their potential application for most effective individualized treatment for gut and distant sites microbiome modulation was preliminary discussed [1].

The objective of our study was to determine in a limited randomized clinical trial the patient- and nosology-specific efficacy of immunobiotics, developed from different original strains of lactobacillus (and their combinations), demonstrating both antimicrobial and immune-modulating functions preliminary examined on animal and cellular models [2].

Patients with infectious-inflammatory diseases of the genitourinary system were recruited from a regional hospital. Eligibility requirements and enrolment procedures were performed in accordance with the EU Regulation No 536/2014. The inclusion criteria for women were age over 18 years, urogenital pathologies of different nosology, and exclusion criteria were detected antibodies to hepatitis B or C; alcohol or drug abuse; HIV infection or antiretroviral therapy; diagnosed diseases of the cervix (dysplasia); menstrual disorders; cancer; pregnancy.

Collected specimens were plated on chromogenic media for detection of cultured urinary tract microbiota representatives and were additionally genetically defined by qRT-PCR (Femoflor®-16, Androflor® REAL-TIME PCR Detection Kit, DNA technologies, Russia). Isolated microorganisms were identified by using the Lachema biochemical tests. Local immune response (IL-10, 17, 1b; TNF-a, SIgA) before and after applied LAB treatment was measured by ELISA. Lactobacilli strains in this study were sequenced and identified as *Lactobacillus casei* (2 strains) and *Lactobacillus acidophilus.*

**Individual gender and nosology specific microbiome changes of infectious-inflammatory diseases of the genitourinary system by application of different lactobacilli strains composition**

The typical agents (nosologies) of infectious-inflammatory diseases of the genitourinary system were identified. The female bacterial vaginitis was characterized mostly by the predominance of *Escherichia coli*, *Staphylococcus aureus*, and *Candida albicans* with or without the presence of *Gardnerella, Atopobium, Ureaplasma* or *Mycoplasma.* Different nosologies of the male urogenital tract diseases were characterized mostly by the prevalence of such opportunistic microorganisms as *Streptococcus* and *Staphylococcus* with or without *Ureaplasma*, *Haemophilus*, Enterobacteriaceae, and *Candida.*

Thus, new effective probiotics and their compositions were designed taking into account individual features of the commensal microbiota of the patients and the dominating agents of opportunistic infections of male and female urogenital systems.

It has been proved that the use of the *L. casei* IMV B-7412 probiotic strain was the most effective in mixed infections classified as a significant anaerobic/aerobic microbial ratio of microbiome, a combination of strains *L. casei* IMV-B 7280 / *L. casei* IMV B-7412 enhances the antifungal effect, while the composition of strains *L. casei* IMV B-7280 and *L. acidophilus* IMV B-7279 promotes the restoration of healthy microbiome, providing complete elimination of coliform bacteria and microscopic fungi, and reducing the number of pathogenic staphylococci.

All LAB strains and their compositions were characterized by strain and compositionally specific ability to restore the local and systemic immune response by decreasing the level of IL-17 and SIgA, and increasing the IL-10 level.

**Proper combination of LAB strains pragmatically designed is crucial to get prognostic healthy microbiome changes**

Efficacy of the application of different pharmabiotics is the subject of great concern since the immunomodulatory properties of its LAB components are mainly strain-dependent and connected to cell walls structure, as was recently demonstrated [3]. There is also an open discussion about oral vs. intra-urogenital administration of various LAB based biopreparation. Their efficacy is dependent on the proper combination of LAB strains, correspondingly pragmatically designed content of pharmabiotics is crucial. The combination of commensal strains that are defining the efficacy by producing specific metabolites or preventing adhesion or even physiological competition with strains should also be considered.

Previously, we were able to demonstrate that following the oral administration of synbiotic Bifiten™, the vagina mucosal sites were settled by both commensal and transit microbiota, which in turns led to a reduction of the perinatal complications for the mother and newborn [4]. At the same time, we were able to observe side effects after application of such complex synbiotics to healthy mothers, who did not require any treatment and were initially characterized by a significant number of their own lactobacilli strains.

**3PM related conclusion and outlook**

Prevention of infection-inflammatory diseases needs to be based on monitoring of individual urogenital system microbiome ratio, since this ratio determines the resistance of the local immune system and initiation of low-grade inflammation.

This approach is in line with proposed 3PM solutions to improve sensitivity and specificity of early stage biomarkers and currently well-discussed [5].

Infants and young people’s urogenital health is dependent significantly on women/mothers’ health encoding due to early programming self-resistance to potentially pathogenic bacteria, candida, but also viruses, mycoplasma, and chlamydia. This preventive approach is crucial for specific person microbiome-based prophylactics.

Another issue is a specific mixture of etiologic agents causing the infectious-inflammatory urogenital disease—“pathogenic formula”, combined with the individual microbiome, connected to specific local immune response, modulation of which is strain-dependent [1, 4]—this issue could be the limitation of recommendation for application of any pharmabiotics strains separately or in combination.

The predictive treatment effect of using any pharmabiotics should be based on the microbiome phenotype of the urogenital system. Then the individual pharmabiotic content selection based on IS models together with ML and databases usage is the way for really prognostic individual efficacy.

3PM requires the understanding of individual differences, thus the “same” nosology needs to be treated differently, based on at least each person’s microbial and immune profile.

**References**

1. Bubnov R, Babenko L, Lazarenko L, Mokrozub V, Spivak M. Specific properties of probiotic strains: relevance and benefits for the host. EPMA J. 2018;9(2):205–223. 10.1007/s13167-018-0132-z.
2. Lazarenko L, Babenko L, Shynkarenko-Sichel L, Pidgorskyi V, Mokrozub V, Voronkova O, Spivak M. Antagonistic action of Lactobacilli and Bifidobacteria in relation to *Staphylococcus aureus* and their influence on the immune response in cases of intravaginal staphylococcosis in mice. Probiotics Antimicro Prot. 2012;84(30):78–89. 10.1007/s12602-012-9093-z
3. Мokrozub V, Lazarenko L, Sichel L, Babenko L, Lytvyn P, Demchenko O, Melnichenko Yu, Boyko N, Biavati B, DiGioia D, Bubnov R, Spivak M. The role of beneficial bacteria wall elasticity in regulating innate immune response. EPMA J. 2015;6:13. 10.1186/s13167-015-0035-1
4. Tsmur O, Levchuk O, Liashyna K, Boyko N. Results of using the domestic synbiotic Bifiten for treatment of bacterial vaginosis of pregnant women. Women’s Health. 2016;6.12: 70–5.
5. Drucker E, Krapfenbauer K. Pitfalls and limitations in translation from biomarker discovery to clinical utility in predictive and personalised medicine. EPMA J. 2013;4:7. 10.1186/1878-5085-4-7

## Biobanking in 3PM

**BRoTHER – a Regional Biobank Network in the Centre of Europe Aimed at Promoting Personalized Medicine Through Digitalization in Biobanking**

Brochhausen C*^1^, Babel M^1^, Kinkorova J^2^, Karlikova M^2^, Becker KF^3^, Niedermair T^1^, Topolcan O^2^, Weichert W^3^, Evert M^1^, Kucera R^2^

^1^Institute of Pathology, University Regensburg, Germany

^2^Department of Immunochemistry Diagnostics, University Hospital in Pilsen, Czech Republic

^3^Institute of Pathology, Technical University Munich, Germany

*Corresponding author: Prof. Dr. Christoph Brochhausen, Institute of Pathology

Franz-Josef-Strauss Allee 11, University Regensburg; e.mail: christoph.brochhausen@ukr.de

**Keywords:** Predictive preventive personalized medicine (PPPM), Biobank, Horizon 2020, Research infrastructures, European Commission, Excellence, Ethical legal, Social issues, Education, Innovation, Digital services, Big data

**Introduction**

Biobanking has been a fast-growing field in basic-, clinical- and translational research over the past 30 years. Biobanks collect, store and share biological material alongside associated data and provide a fundamental scientific infrastructure for personalized medicine [1]. Biobanks represent a key resource for predictive diagnostics, research and experimental therapies [2] and follow the whole chain of medical interventions. By placing an emphasis on prevention, prediction and a healthy lifestyle, biobanks have contributed to the current paradigm shift within medicine from “one-size-fits-all” to “individualized medicine” [3, 4]. Biobanking plays a crucial role in the introduction and further optimization of personalized medicine. Samples stored in biobanks provide information about risk factors of diseases. They assist in the discovery of biomarkers that aid in early diagnosis and a personalized treatment choice, as well as biomarkers for the prediction of initial responses to treatment. Biobank samples also serve as unique sources in the search for drugs [5].

**A Prerequisite for the Optimal Linking of Biobanks and 3P Medicine in Two Neighbouring Regions**

***International collaboration***

Since personalized medicine works with highly specific and well characterized cohorts, national and international cooperation between biobanks as repositories of patient samples and data is key to their optimal utilization. The European Commission strongly supports biobanking as part of the Horizon 2020 research projects and encourages international collaboration and coordination [6, 7].

The BRoTHER (Biobank Research on Telemedical Approaches for Human Biobanks in a European Region) project is a bilateral, cross border collaboration between two regions: German-Bavaria and the Czech Republic. The project is supported by a grant from the Bavarian-Czech University Agency (BTHA) with funding coming from the Bavarian State Ministry of Finance. The aim of the project is to define and analyze the obstacles that must be overcome in order for two clinically related biobanks from two different national health care systems to set up collaboration. The BRoTHER consortium consists of four partners: two in Germany—the Institute of Pathology of the University of Regensburg and the Institute of Pathology at Munich’s Technical University, and two in the Czech Republic—the Department of Immunochemistry at the Faculty Hospital in Pilsen and the Masaryk Memorial Cancer Institute of Masaryk University in Brno (Fig. 1).

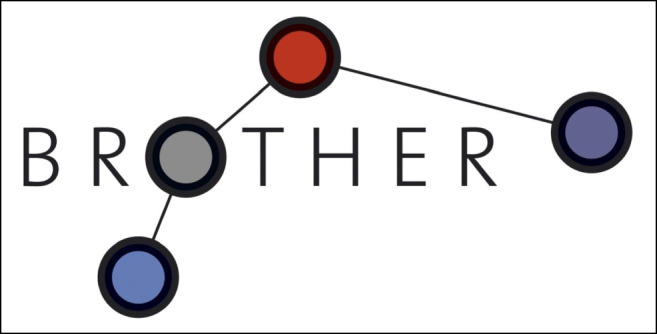



**Fig. 1** The BRoTHER logo

***Innovation***

Biobanks use modern and innovative tools and technologies to make personalized medicine possible. Digitalization is a progressive technological tool and is an important issue in the cooperation between various biobanks. Therefore, whole slide imaging (WSI) and virtual microscopy (VM) technologies are being developed alongside modern immunoanalytical techniques for personalized medicine, cryotechnology and robotics. They will also help resolve IT-related issues in biobanking [8]. The interaction of biobanks from different regions and health care systems is a cornerstone of the acceleration of biomedical research. Despite the harmonizing process in biobanking within the European Union, special challenges remain that must be overcome to facilitate effective and fruitful international collaboration [9].

***Education***

Biobanking as a science is interdisciplinary due to its dual nature involving both biological samples and data. Educated medical staff, specialists for data management and other support services are all cooperating as a consortium with a full range of expertise required for biobanking. There is, as yet, no comprehensive education or training for new biobank staff in Europe and this is urgently needed [10].

Two initiatives were set up for the education of future generations of students: A “Summer School” and an “Intensive course in immunochemistry, biobanking and personalized medicine, students learnt the practical work behind different aspects of biobanking such as cryotechnology, immunochemistry, data-management, WSI, VW and pre-analytics as well as ethical, legal, social issues (ELSI). The lessons were conducted in English with the participation of students from both Germany and the Czech Republic. These two courses are the basis for the preparation of common curricula in the future.

**Conclusions and PPPM-related expert recommendations:**

- Biobanking is a pillar of personalized medicine and contributes to the preventive, predictive and personalized medicine (PPPM) paradigm.
- The BRoTHER project supports education and the dissemination of the ideas behind biobanks to the public, as well as the education of young professionals in the field of biobanking in order to train both current and future biobank staff.
- A new generation of well-educated biobankers is promising for the future development of quality assurance in biobanking. A better quality of biobanks means a better quality of samples as well as the speeding up of biobanks’ operations and an improvement in the standards of subsequent research.
- BRoTHER represents an innovative example of how a young generation of professionals could be systematically acquainted with the principles of PPPM through the platform of biobanks.

**References**

1. Liu A, Pollard K. Biobanking for personalized medicine. Adv Exp Med Biol. 2015;864:55–68.
2. Botti G, Franco R, Cantile M, Ciliberto G, Ascierto PA. Tumor biobanks in translational medicine. J Translat Med. 2012;10:204.
3. Jose R, Rooney R, Nagisetty N, Davis R, Hains D. Biorepository and integrative genomics initiative: designing and implementing a preliminary platform for predictive, preventive and personalized medicine at a pediatric hospital in a historically disadvantaged community in the USA. EPMA J. 2018; 10.1007/s13167-018-0141-y.
4. Kinkorová J, Topolčan O. Biobanks in Horizon 2020: sustainability and attractive perspectives. EPMA J. 2018; 10.1007/s13167-018-0153-7.
5. Hewitt RE. Biobanking: the foundation of personalized medicine. Curr Opin Oncol. 2011;23:112–119
6. Shabo A. Meaningful use of patient-centric health records for personalized medicine. EPMA J. 2011;2:184–185.
7. Golubitschaja O, Kinkorova J, Costigliola V. Predictive, preventive and personalised medicine as the hardcore of “Horizon 2020”: EPMA position paper. EPMA J. 2014;5:6.
8. Golubnitschaja O, Baban B, Boniolo G, Wang W, Bubnov R, Kapalla M, et al. Medicine in the early twenty-first century: paradigm and anticipation - EPMA position paper 2016. EPMA J. 2016;7:23.
9. Golubnitschaja O. Time for new guidelines in advanced healthcare: the mission of The EPMA Journal to promote an integrative view in predictive, preventive and personalized medicine. EPMA J. 2012;3:5.
10. Kinkorová J. Biobanks in the era of personalized medicine: objectives, challenges, and innovation. EPMA J. 2016;7:4.

BRoTHER is an acronym for Biobank Research on Telemedical Approaches for Human Biobanks in a European Region. The round dots represent the biobank locations in the colour of the respective corporate identities (blue for Munich, grey for Regensburg, red for Pilsen and dark blue for Brno).

**BBMRI-ERIC and ISBER: two approaches to biobanking**

Kinkorova J*, Simanek V, Kucera R, Karlikova M, Topolcan O

Department of Immunochemistry Diagnostics, University Hospital in Pilsen, Czech Republic

*Corresponding author: Judita Kinkorova, Department of Immunochemistry Diagnostics, University Hospital in Pilsen, CZ; e.mail: kinkorovaj@fnplzen.cz

**Keywords:** biobanks, preventive predictive personalized medicine (PPPM), biomarkers, innovation, predictive diagnostics, BBMRI-ERIC, ISBER, Horizon 2020, ELSI

**Introduction**

During the past three decades biobanking has grown dramatically and has become an important source for biomedical research. Biobanks are considered one of the pillars of personalized medicine. Biobanks are special facilities containing biological samples - usually - human samples and related data and information [1]. Samples stored in biobanks serve researchers all over the world, helping solve both local and widely international health problems. As some diseases (cancers, cardiovascular diseases, diabetes, neurodegenerative, etc.) have a global dimension, biobanking - the cornerstone for biomarker discovery, has gained validation and is being used in prevention, prediction, diagnosis and therapy as part of a personalized approach to the patient [2]. International collaboration and communication between biobanks worldwide is one of the basic characteristics of biobanking.

**Biobanking: European and global strategies**

Biobanking is strongly supported by the European Commission and by a wide range of research and collaborative projects that are part of the biggest EU research and innovation program ever, Horizon 2020 [3]. The societal challenge of Horizon 2020 is ‘Health, demographic change and wellbeing (SC1)’. It is a call for - better Health and care, economic growth and sustainable health systems, with one of the priorities being ‘personalized medicine’ [4, 5, 6]. As biomedical research moves forward, there is a paradigm shift in medicine from a reactive to a proactive approach that is predictive, preventive, personalized and participatory [7]. Biobanking and major chronic illnesses have a global dimension. Globalization raises new ethical, legal and social (ELSI) questions as well as regulatory, technical and managerial questions [2, 3]. Currently, two international biobank entities: BBMRI-ERIC (Biobanking and BioMolecular resource Research Infrastructure - European Research Infrastructure Consortium) and ISBER (International Society for Biological and Environmental Repositories) are leading bodies in biobanking.

**BBMRI-ERIC**

BBMRI is a European research infrastructure for biobanking, whose purpose is to pool all the main actors in biobanking in order to boost biomedical research. BBMRI provides support and assistance for quality management services, ethical, legal and social issues. IT online tools to facilitate the exchange of samples, mainly in Europe. BBMRI-ERIC currently includes 20 countries, as well as one international organization, making it one of the largest European research infrastructures. BBMRI-ERIC was initiated by the European Commission as a research infrastructure in 2007–2013. During its preparatory phase (2008–2011), possible members - European biobanks - were identified, and in 2013 BBMRI-ERIC became an EU infrastructure. Current hot topics in the European biobank infrastructure are GDPR (General Data Protection Regulation) and its implementation via Code of Conduct, as well as two supportive IT tools: sample/data negotiator and sample/data locator. The Negotiator enables users to negotiate access to samples and datasets in biobanks. The Negotiator focuses on making communication between a high number of requesters and biobanks as efficient as possible. The Locator helps locate samples and datasets hosted by biobanks that are of interest to the requesters and will allow for detailed, privacy-preserving, multi-criteria search of samples and datasets, while also respecting the degree of control required by the biobank infrastructure operators [8]. The current goal of BBMRI-ERIC is to make the new treatment possible.

**ISBER**

ISBER is a global biobanking organization which creates opportunities for networking, education, and innovations and harmonizes approaches to evolving challenges in biological and environmental repositories. ISBER fosters collaboration; creates education and training opportunities; provides a forum for the dissemination of state-of-the-art policies, processes, and research findings; and provides an international showcase for innovative technologies, products, and services. Together, these activities promote the best practices that cut across the broad range of repositories that ISBER serves [9]. ISBER was initially planned in 1999, and the first regular annual meeting took place in 2001. ISBER provides services around the world and has four domains: Americas, Europe, Middle East and Africa, Indo-Pacific, and China. In comparison to BBMRI-ERIC, ISBER is global; reflecting a wider range of repository types and trying to formulate future trends and challenges in biobanking. Notwithstanding, human biobanks are the core of ISBER activities. The newly published Best Practices in 2018, 4th edition [10] pays special attention to the ELSI, thus bringing medicine closer to a patient-focused approach and promoting a personalized approach. BBMRI-ERIC on the other hand is mainly a European entity with a focus on enabling samples and data exchange between European BBMRI-ERIC partners.

**Conclusions and PPPM-related expert recommendations**

- Biobanking is a cornerstone of personalized medicine; samples and related data are of eminent importance in predictive diagnostics and targeted prevention. The main actors in the field of biobanking are BBMRI-ERIC, the biggest European Research Infrastructure, and ISBER. They determine the future developments in the field.
- Horizon 2020 and Horizon Europe are EU tools that reflect the current needs in personalized medicine and biobanking. EPMA, BBMRI-ERIC, and ISBER, all main actors in biobanking, have to contribute to identifying the optimal solutions for future research in order to reach full compliance with PPPM standards.

**Acknowledgements** Supported by Ministry of Health, Czech Republic - conceptual development of research organization (Faculty Hospital in Pilsen - FNPl, 00669806) and BBMRI-CZ: Biobank network - a versatile platform for the research of the etiopathogenesis of diseases CZ.02.1.01/0.0/0.0/16_013/0001674, Bank of the clinical samples LM2018125.

**References**

1. Kinkorová J. Biobanks in the era of personalized medicine: objectives, challenges, and innovation. EPMA J. 2016;7:4.
2. Hewitt R, Hainaut P. Biobanking in a fast moving world: an international perspective. J Nat Cancer Inst Monographs. 2011; 10.1093/jncimonographs/Igr005.
3. Golubnitschaja O, Baban B, Boniolo G, Wang W, Bubnov R, Kapalla M, et al. Medicine in the early twenty-first century: paradigm and anticipation - EPMA position paper 2016. EPMA J. 2016;7:23.
4. Kinkorová J, Topolčan O. Biobanks in Horizon 2020: sustainability and attractive perspectives. EPMA J. 2018; 10.1007/s13167-018-0153-7.
5. Horizon 2020 Work Programme 2018–2020 8. Health, demographic change and wellbeing. https://ec.europa.eu/research/participants/data/ref/h2020/wp/2018-2020/main/h2020-wp1820-health_en.pdf.
6. Golubitschaja O, Kinkorová J, Costigliola V. Predictive, preventive and personalised medicine as the hardcore of “Horizon 2020”: EPMA position paper. EPMA J. 2014;5:6.
7. Hood L, Friend SH. Predictive, personalized, preventive, participatory (P4) cancer medicine. Nat Rev ClinOncol. 2011;8:184–7.
8. BBMRI-ERIC IT supportive tools: http://www.bbmri-eric.eu/services-support/.
9. ISBER. https://www.isber.org/page/About
10. ISBER. Best practices: recommendations for repositories, fourth edition. https://www.isber.org/page/BPR.

